# Palladium-Catalyzed Site-Selective Regiodivergent Carbocyclization of Di- and Trienallenes: A Switch between Substituted Cyclohexene and Cyclobutene

**DOI:** 10.1021/jacs.5c00739

**Published:** 2025-03-06

**Authors:** Wei-Jun Kong, Haibo Wu, Jia-Yi Chen, Rong-Zhen Liao, Yaoyao Liu, Zhipu Luo, Ivo Pires Vilela, Pan Fang, Fahmi Himo, Jan-E. Bäckvall

**Affiliations:** † Department of Chemistry, Arrhenius Laboratory, Stockholm University, SE-106 91 Stockholm, Sweden; ‡ School of Chemical Science and Engineering, Tongji University, Shanghai 200092, China; § Key Laboratory of Material Chemistry for Energy Conversion and Storage, Ministry of Education, Hubei Key Laboratory of Bioinorganic Chemistry and Materia Medica, Hubei Key Laboratory of Materials Chemistry and Service Failure, School of Chemistry and Chemical Engineering, Huazhong University of Science and Technology, Wuhan 430074, China; ∥ MOE Key Laboratory of Geriatric Diseases and Immunology, Suzhou Key Laboratory of Pathogen Bioscience and Anti-infective Medicine, Institute of Molecular Enzymology, School of Life Sciences, Suzhou Medical College, Soochow University, Suzhou 215123, China

## Abstract

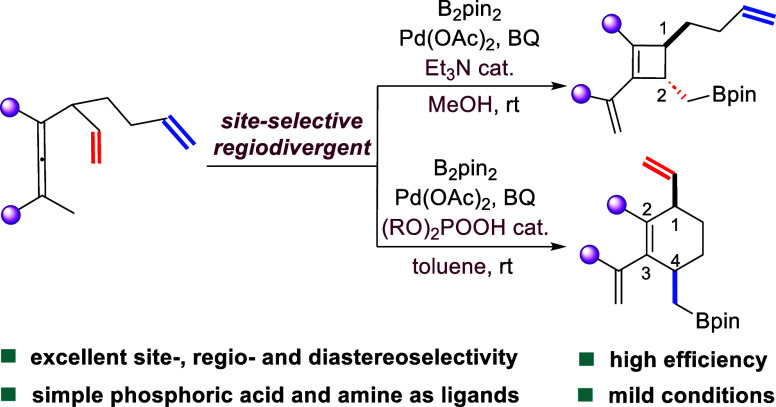

Nature efficiently produces a myriad of structurally diverse carbon ring frameworks from common linear precursors via cyclization reactions at specific olefinic sites in dienes or polyenes. In contrast, achieving the site-selective functionalization of diene or polyene substrates remains a formidable challenge in chemical synthesis. Herein, we report a pair of highly site-selective, regiodivergent carbocyclization reactions of dienallenes and trienallenes, enabling the efficient synthesis of *cis*-1,4-disubstituted cyclohexenes and *trans*-1,2-disubstituted cyclobutenes from a common precursor with high diastereoselectivity. Remarkably, simple achiral organophosphoric acids and amines are identified as powerful ligands for controlling these palladium-catalyzed regiodivergent carbocyclizations. This approach represents the first example of site-selective regiodivergent carbocyclization, providing a practical method for the stereospecific synthesis of thermodynamically disfavored *cis*-1,4-disubstituted cyclohexenes and fully substituted *trans*-1,2-cyclobutenes. Additionally, the methodology developed offers general insights into the development of metal-catalyzed site-selective, regiodivergent carbocyclizations of diene and polyene precursors, mimicking natural carbocyclization processes.

## Introduction

**Figure 1:**
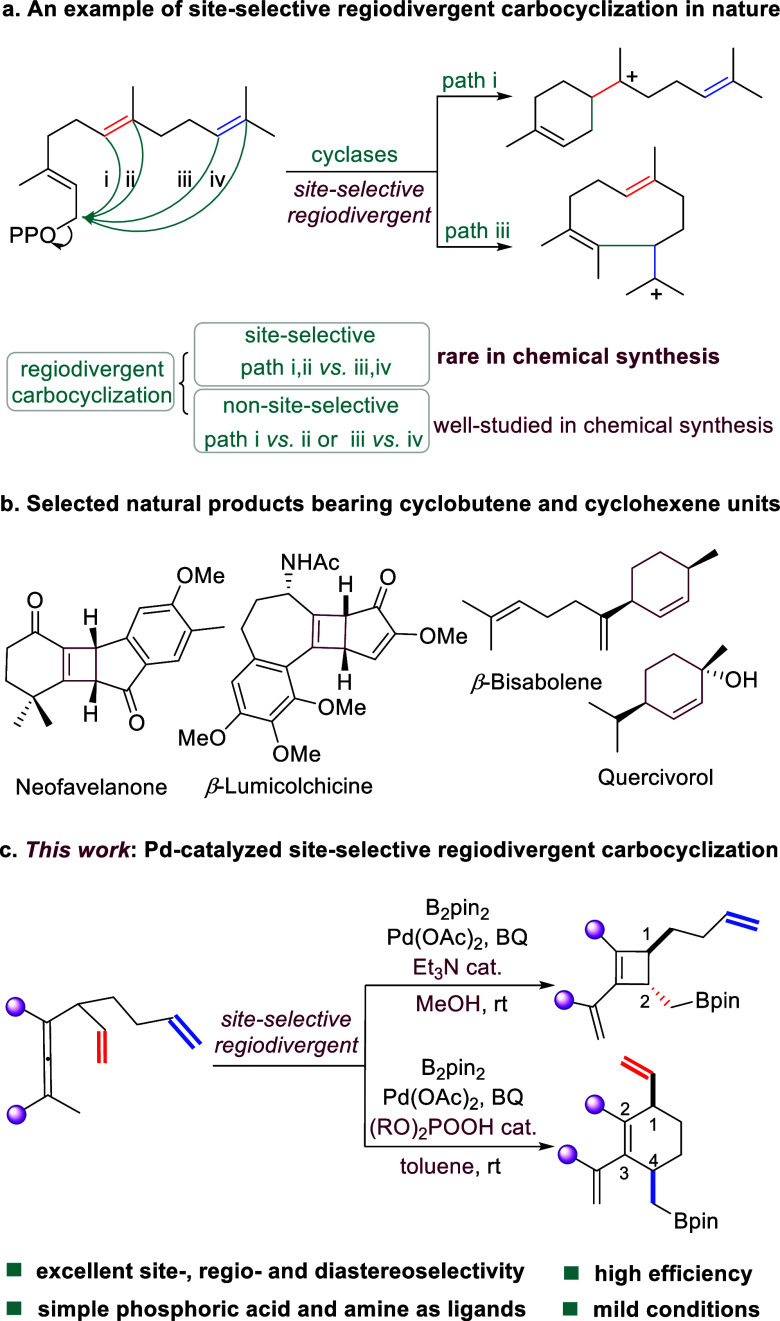
Introduction to the development of Pd-catalyzed site-selective regiodivergent carbocylization for the divergent access to cyclobutenes and cyclohexenes.

In the process of converting a series of simple C_5n_ linear precursors into thousands of terpenoid natural products, nature exhibits unparalleled synthetic efficiency in constructing structurally diverse carbon ring systems (Figure 1a).^1^ While modern chemical synthesis can replicate nearly all these naturally occurring carbon skeletons, it predominantly relies on distinct precursors for biogenetically homologous natural products.^2^ In this context, developing methods that enable divergent synthesis of carbon ring systems from common diene or polyene precursors represents an important strategy for enhancing synthetic efficiency and increasing chemodiversity in chemical synthesis. However, achieving site-selective functionalization or derivatization of dienes or polyenes remains a general formidable challenge in chemical synthesis.^3^

The construction of stereodefined saturated or partially saturated ring systems has always been an important goal in the synthetic community because of their prevalence in natural products and pharmaceuticals.^4^ Cyclobutenes^5^ and cyclohexenes^6^ constitute two common classes of carbon ring motifs present in many natural products and functional molecules (Figure 1b); therefore, their synthesis has attracted significant attentions. On the one hand, cyclobutene, as a typical highly strained ring system, has conventionally been synthesized primarily through photocatalytic or transition metal-catalyzed [2 + 2] cycloadditions,^7,8^ which often require electronically or sterically demanding alkyne or alkene substrates. In recent years, transition metal-catalyzed cyclization has emerged as a promising alternative to access cyclobutenes,^9^ but examples of synthesis of fully substituted cyclobutenes are still rare.^8c,8d^ On the other hand, even though the Diels–Alder reaction is a powerful tool for cyclohexene synthesis,^10^ it is slow with unsubstituted ethylene as dienophile, and furthermore, substitution on either ethylene or diene often leads to regioselectivity problems. Moreover, control of the stereochemistry toward the synthesis of thermodynamically disfavored isomers (e.g., *cis*-1,4-disubstituted cyclohexene) is still a general challenge for the constructions of disubstituted cyclohexenes and cyclohexanes.^11^ A recent work^12^ by the Yin group showcased a nickel-catalyzed migratory difunctionalization of methylenecyclohexane, providing a modular access to thermodynamically disfavored substituted saturated six-membered rings. In 2020, our group reported a Pd/ETM (ETM = electron transfer mediator)-catalyzed stereoselective carbocyclzation,^13^ which also furnished thermodynamically disfavored *cis*-1,4-disubstituted O- and N-heterocycles. Despite these advances, a reliable protocol for the synthesis of *cis*-1,4-disubstituted cyclohexenes is still exclusive. Given the prevalence of both cyclobutenes and cyclohexenes as well as the limitations of current synthetic methods, the development of efficient strategies, particularly divergent ones, for accessing both classes of ring systems is highly desirable.

Transition metal-catalyzed carbocylization reactions are fundamental tools for the construction of diverse carbon ring systems in organic synthesis.^14^ Catalyst-controlled regiodivergent transformations using a single substrate to access two distinct products constitute an appealing strategy, especially for small molecule library construction.^15^ However, the current state-of-the-art in this field is largely limited to the regioselectivity concerning two different positions of an alkene (nonsite-selective, Figure 1a) or an alkyne.^16,17^ Ligand-controlled site-selective regiodivergent carbocyclization reactions that form differently sized rings (Figure 1a) are rarely studied. Inspired by our previous work on palladium-catalyzed oxidative transformations of allenes^18^ and the finding that a proximal olefin is an effective directing group for allenic C–H bond cleavage,^19^ we speculated that the installation of a second distal olefin group would enable the possible access to both 4-*exo*-trig and 6-*exo*-trig cyclization products. Herein, we report the discovery of a pair of palladium-catalyzed site-selective regiodivergent carbocyclization/borylation reactions of dienallenes and trienallenes for the divergent synthesis of *cis*-1,4-disubstituted cyclohexenes and *trans*-1,2-disubstituted cyclobutenes with simple organophosphoric acids and amines as controlling additives, respectively (Figure 1c).

## Results and Discussion

We commenced the borylative cyclization with dienallene **1a** as the model substrate and bis(pinacolato)diboron (B_2_pin_2_) as the borylation reagent. The initial trial was run with 1,4-benzoqunone (BQ) as oxidant (1.1 equiv) in the presence of catalyst precursor palladium acetate (Pd(OAc)_2_) in toluene at room temperature (rt) (entry 1, Table 1). Interestingly, the envisioned cyclohexene was formed as the *cis* isomer (**2a**) in 17% yield with a 10:1 diastereomeric ratio (*dr*). The major product was identified as *trans*-1,2-disubstituted cyclobutene **3a** (67% yield with 3:1 *dr*), which was formed through the cyclization of the proximal olefin. To increase the regio- and diastereoselectivity, reaction conditions were screened including the use of various additives. Phosphoric acids were tested because they are versatile ligands/counterions for transition metal-catalyzed asymmetric transformations, including palladium-catalyzed reactions.^20^ In addition to imparting chirality, phosphoric acids also affect the reactivity and selectivity at the same time. The addition of 6 mol % BINOL phosphoric acids **L1** and **L2** increased the yield of **2a** to 95% (11:1 *dr*) and 85% (13:1 *dr*), respectively, with the complete suppression of **3a** formation (entries 2 and 3, Table 1). The spiro-phosphoric acid **L4** gave **2a** in 74% yield with 11:1 *dr*, along with **3a** in 20% yield with 2:1 *dr* (entry 4, Table 1). The 2,2′-diphenol- and VAPOL-phosphoric acids **L4** and **L5** both afforded **2a** in good yields (75% and 76%) with excellent regioselectivity but with low diasteroselectivity (3.3:1 and 4:1 *dr*, respectively, (entries 5 and 6, Table 1),). The ratio **2a**:**3a** deteriorated to about 1:1 when *bis*-BINOL based phosphoric acids **L6** and **L7** were applied (entries 7 and 8, Table 1). However, the same backbone-based phosphoric acid **L8** delivered **2a** exclusively in 97% yield with 18:1 *dr* (entry 9, Table 1). Di-isopropyl and di-*tert*-butyl phosphoric acids led to about a 1:1 ratio of **2a** to **3a** (entries 10 and 11, Table 1). To our delight, simple diaryl phosphoric acids **L11**-**L15** could also produce **2a** in a regiospecific manner and excellent yields with good to high *dr* values (entries 12–16, Table 1). When catalytic amounts of triethylamine (Et_3_N) were added to the reaction in toluene, **3a** was formed as the major product in 78% yield (3:1 *dr*), along with 10% of **2a** (entry 17, Table 1). Changing the solvent to methanol (MeOH) in the presence of Et_3_N as an additive further improved the yield and *dr* of **3a** to 86% and 9:1, respectively (entry 18, Table 1). The reaction in MeOH without Et_3_N gave inferior performance in terms of regioselectivity, yield, and *dr* (entry 19, Table 1). Replacing Et_3_N with DABCO (1,4-diazabicyclo 2.2.2 octane) improved the diastereoselectivity of **3a** to 12:1 and gave the highest regioselectivity with a slightly decreased yield (79%) (entry 20, Table 1). Interestingly, when Pd(TFA)_2_ was used instead of Pd(OAc)_2_, the reaction yielded the cyclohexene product in excellent yield (96%) but with a moderate *dr* of 5:1 (entry 21, Table 1). When another precatalyst, PdCl_2_, was tested, no desired cyclization products were obtained (entry 22, Table 1). A control experiment was conducted with 6 mol % of **L15** and 2.0 equiv of NaOAc to elucidate the effect by added acetate (entry 23, Table 1). Interestingly, the reaction afforded cyclobutene **3a** in 60% yield (3:1 *dr*) and cyclohexene **2a** in 17% yield (10:1 *dr*), showing that the added acetate suppresses the effect by the phosphoric acid. The outcome of this experiment is similar to that when only Pd(OAc)_2_ is used without phosphoric acid (entry 1, Table 1).

**Table 1:**
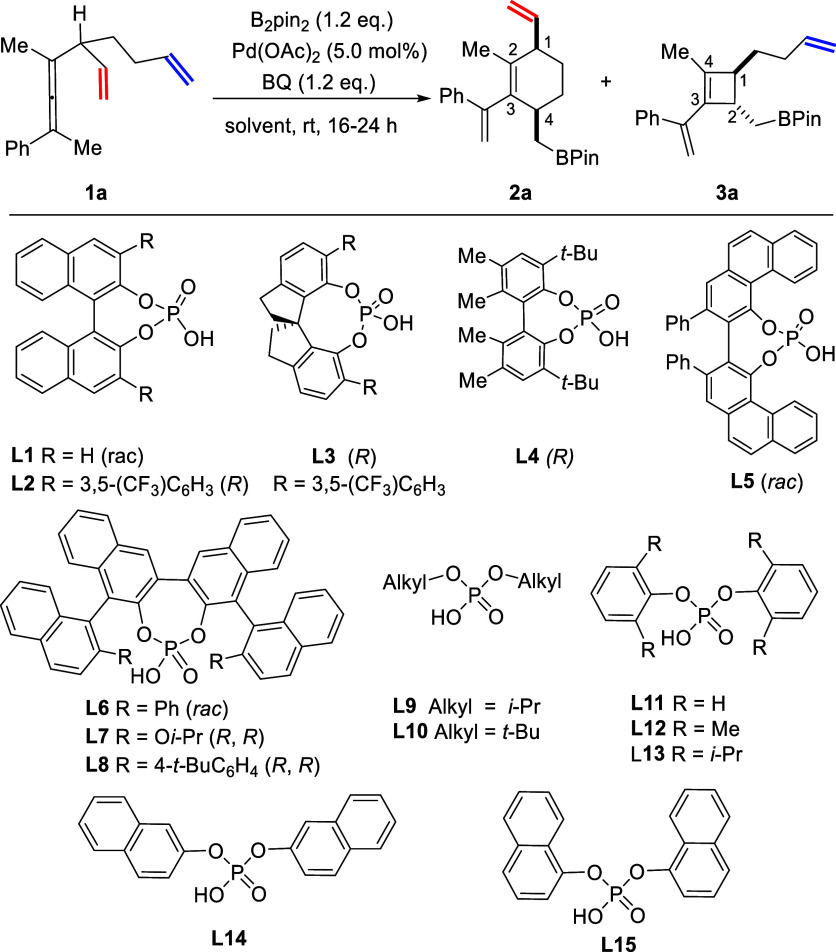
Optimization of the Regiodivergent Synthesis of Cyclobutene and Cyclohexene^a^

entry	additive	solvent	**2a** [%](*dr*)	**3a** [%](*dr*)
1		toluene	17 (10:1)	67 (3:1)
2	**L1**	toluene	95 (11:1)	0
**3**	**L2**	toluene	85 (13:1)	0
4	**L3**	toluene	74 (11:1)	20 (2:1)
5	**L4**	toluene	75 (3.3:1)	0
6	**L5**	toluene	76 (4.0:1)	0
7	**L6**	toluene	57 (10:1)	43 (2:1)
8	**L7**	toluene	51 (12:1)	40 (1.4:1)
9	**L8**	toluene	97 (18:1)	0
10	**L9**	toluene	45 (10:1)	43 (2.3:1)
11	**L10**	toluene	45 (10:1)	49 (2.5:1)
12	**L11**	toluene	93 (13:1)	0
13	**L12**	toluene	92 (10:1)	0
14	**L13**	toluene	94 (7.5:1)	0
15	**L14**	toluene	98 (15:1)	0
16	**L15**	toluene	98 (15:1)	0
17^b^	**Et**_**3**_**N**	toluene	10 (ND)	78 (3:1)
18^b^	**Et**_**3**_**N**	MeOH	5 (ND)	86 (9:1)
19		MeOH	20 (9:1)	71 (8.6:1)
20^c^	**DABCO**	MeOH	3 (ND)	79 (12:1)
21^d^		toluene	96 (5:1)	0
22^e^		toluene	0	0
23^f^	**L15**	toluene	17 (10:1)	60 (3:1)

a Reaction conditions: **1a** (0.1 mmol), B_2_pin_2_ (0.12 mmol), Pd(OAc)_2_ (5.0 mol %), additive (6.0 mol %), solvent (1.0 mL) under air, 18 h, rt, yields and diastereoselectivity ratios (*dr*) were determined by ^1^H NMR using CH_3_NO_2_ as internal standard in crude mixture. ND: *dr* value was not determined.

b 20 mol % of triethylamine (Et_3_N).

c 10 mol % of 1,4-diazabicyclo 2.2.2 octane (DABCO).

d Pd(TFA)_2_ instead of Pd(OAc)_2_.

e PdCl_2_ instead of Pd(OAc)_2_.

f 2.0 equiv NaOAc was added together with **L15**.

**Scheme 1:**
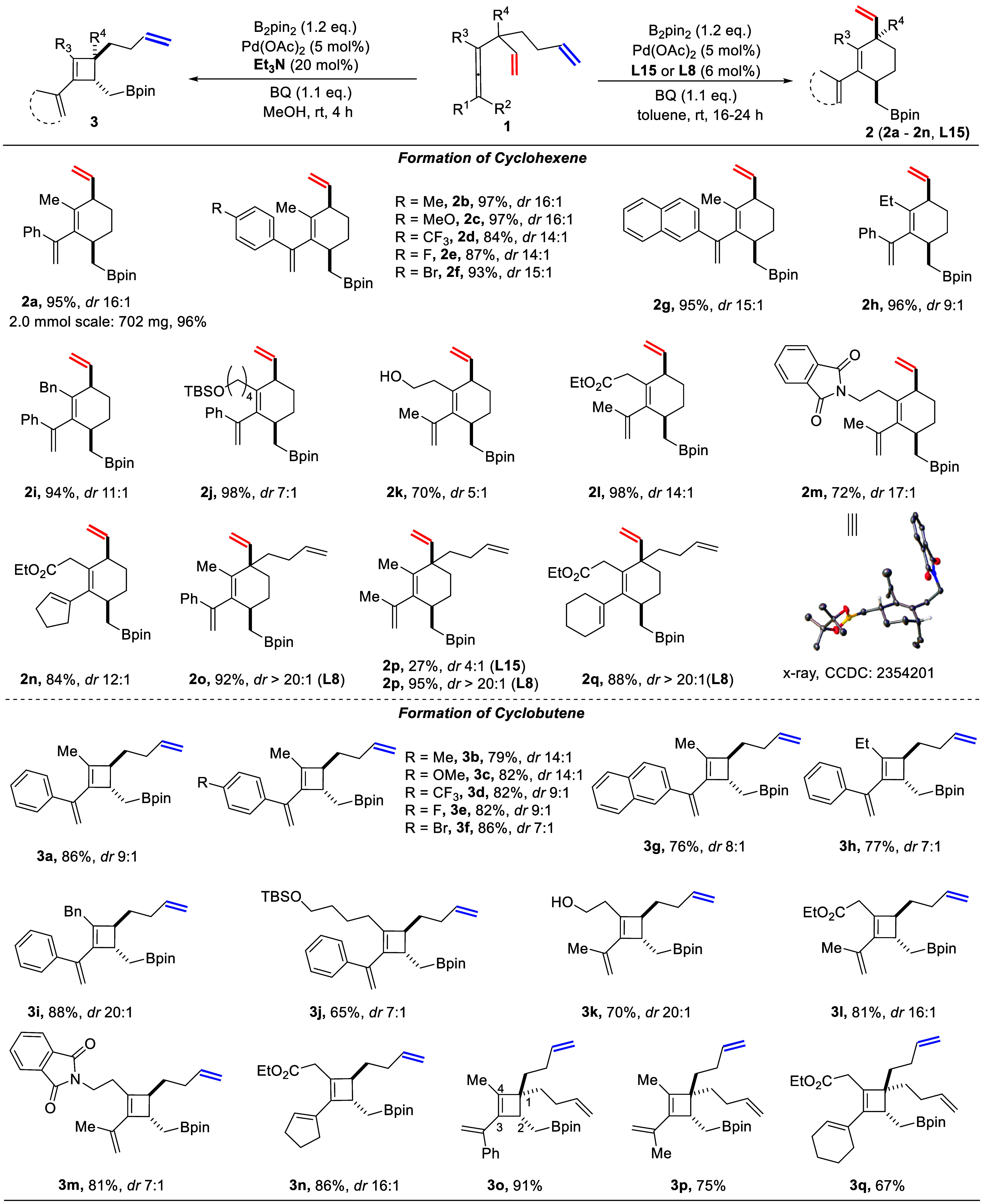
Scope of Di- and Trienallenes for Pd(II)-Catalyzed Regiodivergent Synthesis of Cyclohexenes and Cyclobutenes Reaction conditions: **1** (0.2 mmol, 1.0 equiv), B_2_pin_2_ (0.24 mmol, 1.2 equiv), solvent (2.0 mL) under air. Isolated yields. Diastereoselectivity ratios (*dr*) were determined by ^1^H NMR of the crude reaction mixture.

With the optimal reaction conditions for the switchable synthesis of cyclohexene and cyclobutene in hand (entries 16 and 18, Table 1), a wide range of dienallene derivatives **1** were investigated. **L15** was preferred over **L8** for the synthesis of *cis*-1,4-disubstituted cyclohexenes **2** because of its ready accessibility compared to that of **L8**. The reaction generally afforded **2** exclusively (Scheme 1, upper part). Substrates with electron donating methyl (Me, **1b**) and methoxy (MeO, **1c**) groups on the phenyl ring afforded the corresponding products **2b** and **2c** in excellent yields and diastereoselectivities (97% yield, 16:1 *dr*). Electron-withdrawing groups trifluoromethyl (CF_3_) and fluoro (F) in **1d** and **1e** delivered the fluorinated cyclohexenes **2d** and **2e** in 84% yield with 14:1 *dr* and 87% yield with 14:1 *dr*, respectively. This palladium-catalyzed reaction showed good tolerance to a bromo substituent as **2f** was produced in 93% yield with 14:1 *dr*. Naphthyl substituted dienallene gave the desired product **2g** in 95% yield with 15:1 dr. When the R^3^ group was changed from methyl to ethyl or benzyl groups, the reaction still ran smoothly, affording the corresponding products **2h** and **2i** in excellent yields and in both cases with a slightly decreased *dr* (9:1 and 11:1). Substrates with protected or unprotected hydroxyl groups (**1j** and **1k**) were both compatible with this protocol, and the corresponding cyclohexenes **2j** and **2k** were obtained in good yields. However, the diastereomeric ratio of **2k** with a free hydroxyl group was decreased to 5:1. The ester and imide groups were also well tolerated (**2l** and **2m**). The single-crystal X-ray diffraction structure of **2m** confirmed the *cis* isomer configuration. The allylic C–H bond on a cyclopentene ring could also be activated, furnishing the cyclopentenyl *cis*-1,4-disubstituted cyclohexene **2n** in 84% yield with 12:1 *dr*. The trienallenes (R^4^ = butenyl group) **1o**, **1p,** and **1q** afforded the corresponding products **2o**, **2p,** and **2q**, respectively, in 88–95% yield with *dr* > 20:1 using **L8** as ligand in place of **L15** due to its better performance in improving the reactivity and controlling the regioselectivity. For example, **L15** afforded **2p** in only 27% yield (4:1 *dr*), whereas **L8** gave **2p** in 95% yield (>20:1 *dr*). It is noteworthy that the allylic C–H bond on a cyclohexane ring was also activated (**2q**).

**Scheme 2:**
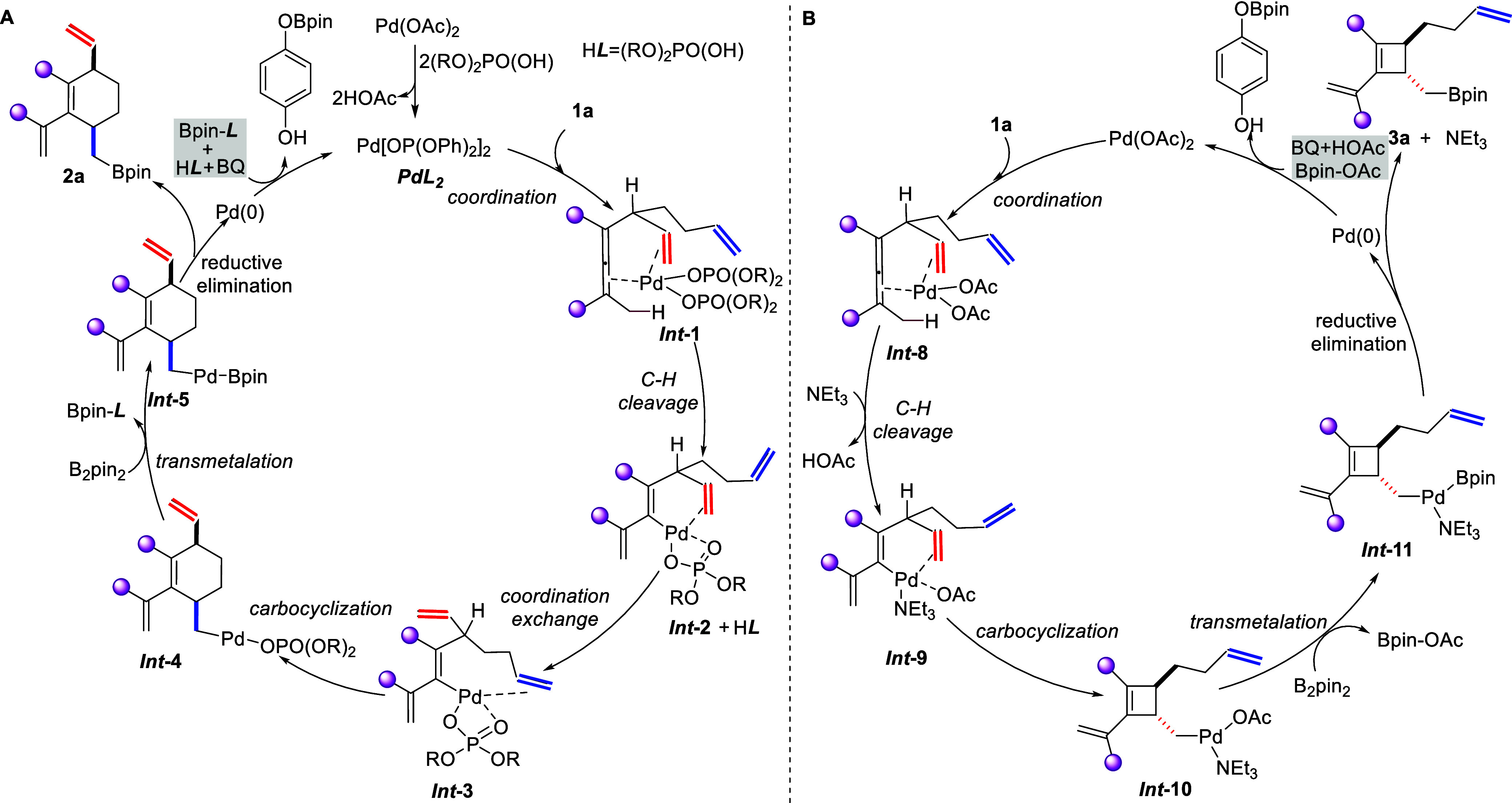
Proposed Catalytic Cycle for Regiodivergent Synthesis of Cyclohexenes (A) and Cyclobutenes (B)

Next, the optimized reaction conditions for the synthesis of *trans*-1,2-disubstituted cyclobut-3-ene **3** were tested for the carbocyclization of the same substrates (**1a**–**1q**) using Et_3_N as additive in MeOH (Scheme 1, lower part). Generally, all the substrates for the synthesis of cyclohexenes **2** were applicable for the synthesis of cyclobutenes **3** with comparable yields and good to excellent diastereoselectivities. The corresponding *trans*-tetrasubstituted cyclobutenes **3a**–**3n** were formed in 65 to 88% yield with *trans*/*cis* ratios from 7:1 to 20:1 (Scheme 2, lower part). Substrates **1b** and **1c** with electron-donating groups Me and MeO on the phenyl rings gave the corresponding cyclobutenes **3b** and **3c** in 79% and 84% yield, respectively, with the same 14:1 *dr*. Substrates with electron-withdrawing groups CF_3_ and F on the phenyl ring showed comparable reactivities as well, delivering the products **3d** (82%) and **3e** (82%) with a 9:1 *dr* for both. Notably, a bromo substituent on the aryl ring was also well tolerated under these reaction conditions, and **3f** was obtained in 86% yield with a dr of 7:1. When changing R^3^ on the allene structure to an ethyl (**1h**) or a benzyl (**1j**) group, the corresponding products were formed in 96% yield (*dr* = 9:1) and 94% yield (*dr* = 11:1), respectively. The functional groups including protected hydroxyl, free hydroxyl, ester, and protected amine were also well tolerated (**3j**–**3n**). When trienallenes (R^4^ = butenyl) **1o**–**1q** were subjected to the typical reaction conditions, tetrasubstituted cyclobutenes were obtained in good yields. These three products (**3o**–**3q**) have no *trans/cis* isomers, because two butenyl groups are connected to the same carbon atom.

A possible mechanism for the regiodivergent carbocyclization is given in Scheme 2. In the presence of phosphoric acid, a palladium phosphate complex is formed. Coordination of substrate **1a**, where the olefin in the shorter chain (marked in red) triggers palladium for a C–H bond cleavage (*Int***-1**),^19a^ leads to dienyl complex *Int***-2**. Ligand exchange leads to an olefin switch with the formation of *Int***-3**. Carbocyclization produces *Int***-4**, which is trapped by B_2_pin_2_ to give product **2a**.

In the absence of the phosphoric acid, palladium acetate reacts with substrate **1a** to give *Int***-8**. The presence of Et_3_N may facilitate the C–H bond cleavage, which releases one molecule of acetic acid and results in the formation of *Int***-9**. Carbocyclization can now occur with the formation of cyclobutene intermediate *Int***-10**, which is trapped by B_2_pin_2_ to give the cyclobutene product **3a**. In contrast to phosphoric acid, Et_3_N could favor formation of intermediate *Int***-9** and avoid interception by the long chain olefin group.

**Figure 2:**
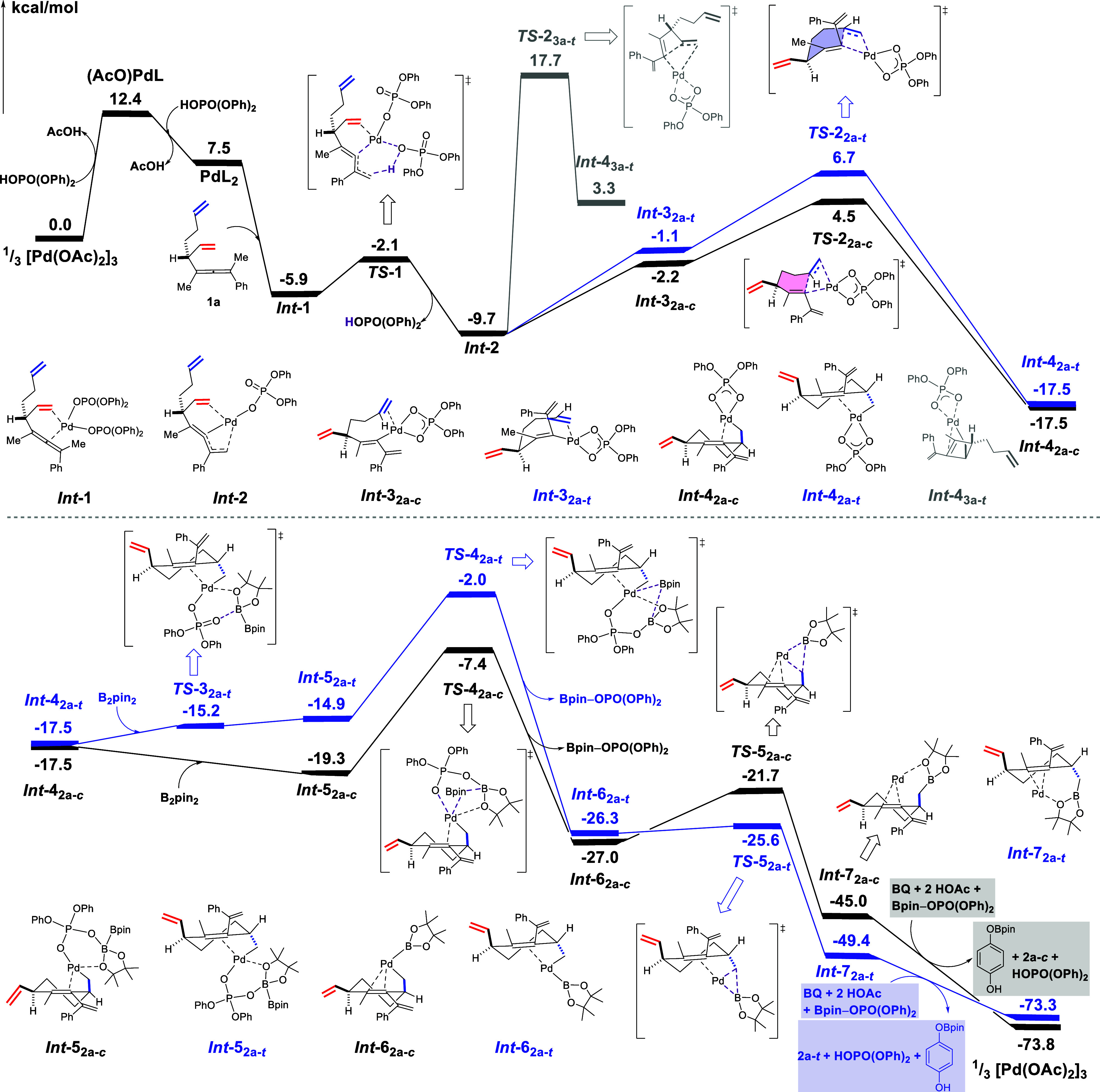
Calculated energy profile (in kcal/mol) for the formation of **2a**. In the calculations, “H**L**” represents ligand **L11**.

To gain further insight into the reaction mechanism, we performed density functional theory calculations (Supporting Information for computational details) using substrate **1a** and ligand **L11** as the model case (Table 1, entry 12). The calculations show that the mechanism in Scheme 2A has feasible energy barriers that are compatible with the reaction conditions. The obtained overall energy profile is shown in Figure 2, while optimized geometries of all intermediates and transition states are provided in the Supporting Information.

**Figure 3:**
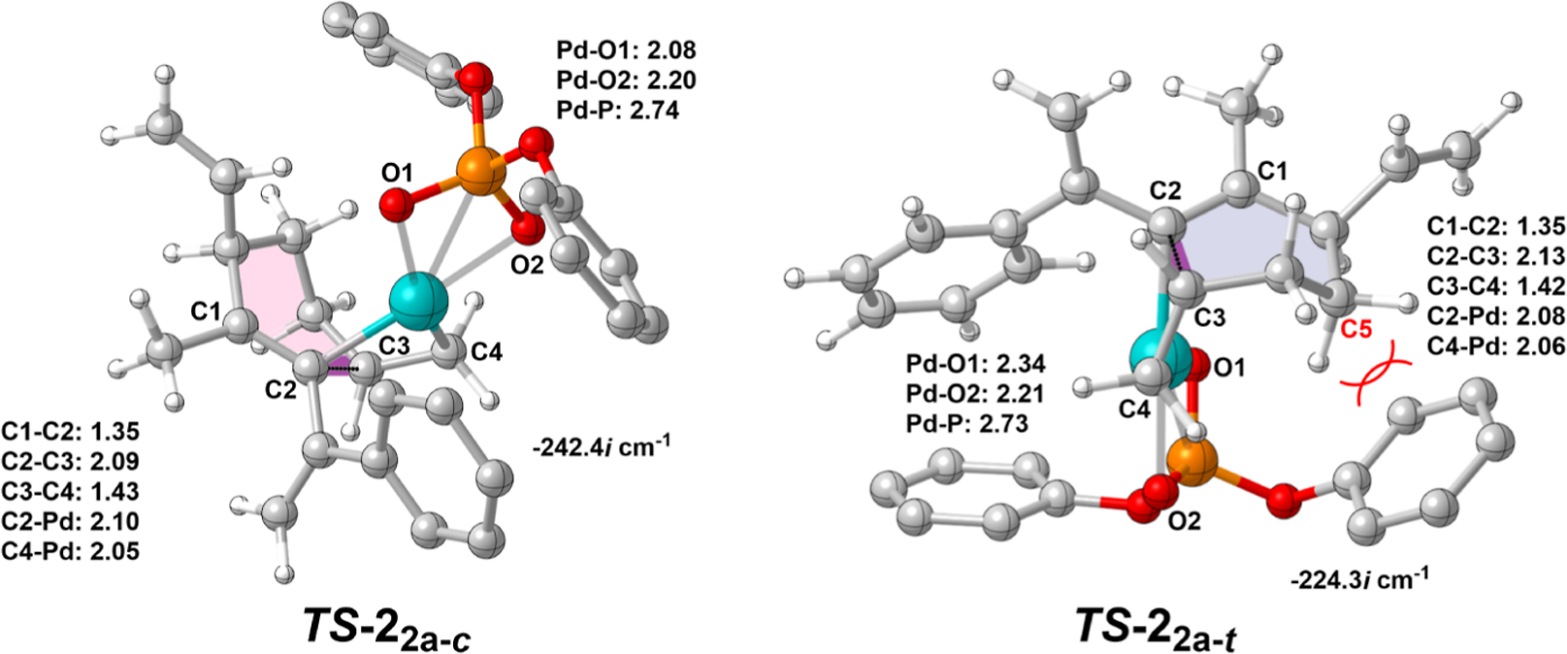
Optimized transition states for carbocyclization step leading to the different isomers of product **2a**. Distances are given in Å. The hydrogen atoms of the phenyl groups are omitted for clarity.

Palladium acetate exists as a trimer, and in the presence of phosphoric acid, the calculations show that palladium diphosphate can form with a penalty of 7.5 kcal/mol. The mixed palladium acetate phosphate complex has a 4.9 kcal/mol higher energy. Substrate **1a** then coordinates to the metal to form ***Int-1***, which is calculated to be 13.4 kcal/mol more stable than palladium-diphosphate. Next, proton transfer from the methyl group of **1a** to the phosphate ligand takes place, with a very low barrier of 3.8 kcal/mol, leading to the dissociation of a phosphoric acid ligand and the formation of the dienyl complex *Int***-2**. Then, upon a coordination change of the metal from the pendent olefin to the distant one, *Int***-3**_**2a-***c*_ is formed, which is 7.5 kcal/mol higher than *Int***-2**. This is followed by the carbocyclization step via *TS***-2**_**2a-***c*_ (shown in Figure 3), with a calculated barrier of 14.2 kcal/mol relative to that of *Int***-2**, to yield the six-membered ring intermediate *Int***-4**_**2a-***c*_. From *Int***-4**_**2a-***c*_, B_2_pin_2_ adds to the oxygen atom of the phosphate ligand leading to *Int***-5**_**2a-***c*_, which is then followed by a transmetalation step through transition state *TS***-4**_**2a-***c*_, with a barrier of 11.9 kcal/mol, to form the Pd–B bond in *Int***-6**_**2a-***c*_. The next step involves the reductive elimination (*TS***-5**_**2a-***c*_), which is associated with a barrier of 5.3 kcal/mol, producing *Int***-7**_**2a-***c*_ in which the Pd(0) coordinates to the C=C double bond of product **2a**. Finally, to close the catalytic cycle, Pd(0) is oxidized to Pd(II) by BQ. The cyclization transition state *TS***-2**_**2a-***c*_ produces the *cis*-isomer of the product. We also calculated the full energy profile for the formation of the *trans*-isomer (also shown in Figure 2), which proceeds via *TS***-2**_**2a-***t*_ (Figure 3). The repulsion between C5 methylene and the phenyl ring on the diphosphate ligand results in an energy of 2.2 kcal/mol higher than *TS***-2**_**2a-***c*_, which is in good agreement with the experimental findings. Interestingly, from *Int***-2** the barrier for the formation of the four-membered ring, which would lead to product **3a** was calculated to be +27.4 kcal/mol (*TS***-2**_**3a-***t*_ in Figure 2), which is as much as 11.0 kcal/mol higher than *TS***-2**_**2a-***c*_, in agreement with the fact that product **3a** is not observed when **L11** is used as ligand.

Regarding the mechanism for the formation of the four-membered ring corresponding to Scheme 2B, we could not obtain reasonable energy barriers in the DFT calculations despite examining many options, indicating that perhaps some element is missing in this mechanism.

**Scheme 3:**
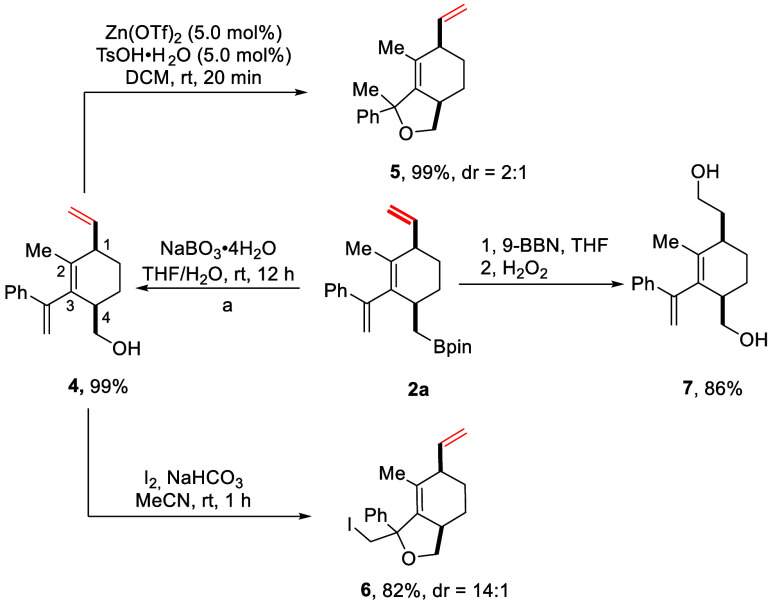
Representative Transformations of Cyclohexene **2a**

The olefin and boronate ester groups on *cis*-1,4-disubstituted cyclohexene **2** and *trans*-1,2-disubstituted cyclobutene **3** are both versatile, transformable functional groups. To further demonstrate the synthetic utility of the cyclohexene protocol, representative transformations of **2a** were conducted, as shown in Scheme 3. The oxidation of **2a** with sodium perborate (NaBO_3_·4H_2_O) afforded the corresponding alcohol **4** in a nearly quantitative yield. The intramolecular hydroalkoxylation of the vinyl group bound to C3 in **4** in the presence of catalytic amounts of zinc triflate (Zn(OTf)_2_) and *p*-toluenesulfonic acid monohydrate (TsOH·H_2_O) gave the bicyclic hexahydrobenzofuran **5** in 99% yield and diastereoselectivity ratio of 2:1. Similarly, iodoalkoxylation of **4** with stochiometric iodine and sodium bicarbonate as base gave the bicyclic iodide **6** in 82% yield and 14:1 *dr*. Hydroboration-oxidation of **2a** transformed the olefin group and boronate group in one pot to give diol **7** in 86% yield.

## Conclusions

In summary, we achieved a palladium-catalyzed switchable synthesis of *cis*-1,4-disubstituted cyclohexenes and *trans*-1,2-disubstituted cyclobutenes, which was enabled by simple binaphthyl phosphoric acid and triethyl amine additives. This protocol features excellent regioselectivity, diastereoselectivity, and mild reaction conditions. The transformations of olefin and boronate ester groups on the products provide expedient access to representative derivatives and further demonstrate the synthetic utility of the developed methodology.

## Supporting Information Available

Experimental procedures; characterization data for all new compounds; NMR spectra; computational studies; and additional relevant references (PDF)
