# Supplementary material for: Palladium-Catalyzed Site-Selective Regiodivergent Carbocyclization of Di- and Trienallenes: A Switch between Substituted Cyclohexene and Cyclobutene
Source: J Am Chem Soc. 2025 Mar 6;147(11):9909–18. doi: 10.1021/jacs.5c00739 (PMC11926882; doi:10.1021/jacs.5c00739)
Supplement: Supplementary file 1 — ja5c00739_si_001.pdf [file ja5c00739_si_001.pdf]

Supporting Information

Palladium-Catalyzed Site-Selective Regiodivergent Carbocyclization of Di- and Trienallenes: a Switch between Substituted Cyclohexene and Cyclobutene

Wei-Jun Kong,[1,2 ‡,*] Haibo Wu,[1,‡,*] Jia-Yi Chen,[1,3,‡] Rong-Zhen Liao,[3] Yaoyao Liu,[4] Zhipu Luo,[4] Ivo Pires Vilela,[1] Pan Fang,[4] Fahmi Himo,[1,*] and Jan-E. Bäckvall[1,*]

[1]Department of Chemistry, Arrhenius Laboratory, Stockholm University, SE-106 91 Stockholm, Sweden;

[2]School of Chemical Science and Engineering, Tongji University, Siping Road 1239, Shanghai, China;

[3]Key Laboratory of Material Chemistry for Energy Conversion and Storage, Ministry of Education, Hubei Key Laboratory of Bioinorganic Chemistry and Materia Medica, Hubei Key Laboratory of Materials Chemistry and Service Failure, School of Chemistry and Chemical Engineering, Huazhong University of Science and Technology, Wuhan 430074, China

[4]MOE Key Laboratory of Geriatric Diseases and Immunology, Suzhou Key Laboratory of Pathogen Bioscience and Anti-infective Medicine, Institute of Molecular Enzymology, School of Life Sciences, Suzhou Medical College, Soochow University, China.

Corresponding authors: Wei-Jun Kong: kongwj@tongji.edu.cn ; Haibo Wu: haibo.wu@su.se ; Fahmi Himo: fahmi.himo@su.se ;Jan-E. Bäckvall: jeb@organ.su.se.

‡These authors contributed equally (WK, HW and JC).

Contents

1. General Information S2
2. Optimization of Reaction Conditions S2
3. General Procedures for the Synthesis of Cyclohexenes and Cyclobutenes S3
   3.1 General Procedure A: Synthesis of Cyclohexene **2** S3
   3.2 General Procedure B: Synthesis of Cyclobutene **3** S12
3. Procedures for the Synthesis of Allenes S20
   3.1 General Procedure C for the Synthesis of Allene **1a-1j** S20
   3.2 Procedure for the Synthesis of Allene **1k** and **1m** S26
   3.3 Procedure for the Synthesis of Allene **1l** and **1n** S27
   3.4 Procedure for the Synthesis of Allene **1o** S30
   3.5 Procedure for the Synthesis of Allene **1p** and **1q** S32
4. Transformation of Products S36
5. X-Ray Crystallographic Analysis S39
6. Determination of the Relative Configuration of **3k** S55
7. Computational Details S60
8. References S83
9. ^{1}H NMR and ^{13}C NMR Spectra S84

1. General Information

Unless otherwise noted, all reagents were used as received from commercial suppliers. Dry solvents were obtained from commercial sources, from a VAC™ drying system or dried over molecular sieves. Palladium acetate ($Pd(OAc)_2$), bis(pinacolato)diboron (B_2pin_2), phosphoric acids **L1-L5**, **L9**, **L10**, and **L11** were purchased from Sigma-Aldrich. **L6**,[1] **L7**,[2] **L8**,[1] **L12**,[3] **L13**,[4] **L14**[5] and **L15**[6] were synthesized according to reports in the literature. Reactions were monitored using Merck silica gel 60 F254 plates (TLC analysis) or $KMnO_4$ stain. Flash column chromatography was carried out with 60Å (particle size 35-70 μm) silica gel. ^{1}H/^{13}C NMR experiments were performed on a Bruker NMR (400/101 MHz) or (500/125 MHz) at room temperature. ^{19}F-NMR experiments were performed on a 400 MHz Bruker NMR (377 MHz). The diastereomeric ratios were determined by crude ^{1}H NMR. Chemical shifts (δ) are reported in parts per million (ppm) relative to the $CDCl_3$ peaks (δ(H) = 7.26 and δ(C) = 77.0 ppm). Coupling constants (*J*) are reported in Hertz (Hz). The following abbreviations were used to explain multiplicities: s = singlet, d = doublet, t = triplet, q = quartet, m = multiplet, and br = broad. HRMS were recorded on a Bruker MicroTOF spectrometer equipped with an ESI as ion sources.

2. Optimization of Reaction Conditions

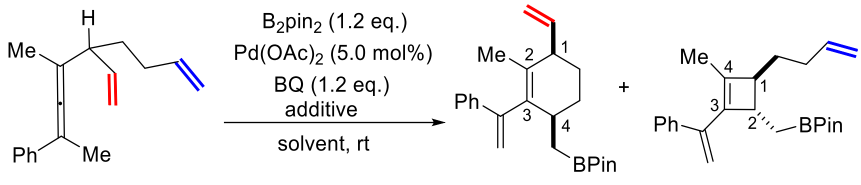

1a **2a** **3a**

To an 8 mL vial equipped with a stirring bar were added $Pd(OAc)_2$ (1.1 mg, 5 mol%), additive (6 mol% phosphoric acid, 20 mol% Et_3N, or 10 mol% DABCO), and solvent (1.0 mL). When phosphoric acid was used as the additive, the mixture was stirred at room temperature for 10 minutes before **1a** (23.8 mg, 0.1 mmol), B_2pin_2 (30.5 mg, 1.2 equiv.), and BQ (13.0 mg, 1.2 equiv.) were added. The reaction mixture was then stirred at room temperature for 16 h. For the cases using Et_3N or DABCO as additives, **1a** (23.8 mg, 0.1 mmol), B_2pin_2 (30.5 mg, 1.2 equiv.), and BQ (13.0 mg, 1.2 equiv.) were added directly to the reaction mixture, which was then stirred at room temperature for

4 h. After completion of the reaction, the solvent was removed under reduced pressure. CH_3NO_2 (16 μL, 0.3 mmol) was then added to the residue, and the yield and diastereomeric ratio were determined by crude ^{1}H NMR analysis

3. General Procedures for the Synthesis of Cyclohexenes and Cyclobutenes

3.1 General Procedure A: Synthesis of Cyclohexene 2

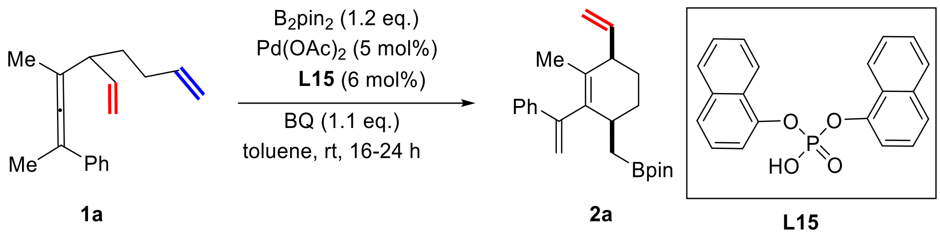

To an 8 mL vial charged with a stirring bar were added $Pd(OAc)_2$ (2.2 mg, 5 mol%), **L15** (4.4 mg, 6 mol%) and 2.0 mL toluene. The mixture was stirred at room temperature for 10 min before **1a** (47. 6 mg, 0.2 mmol), B_2pin_2 (61.0 mg, 1.2 equiv.) and BQ (26.0 mg, 1.1 equiv.) were added. TLC analysis was conducted for each reaction, conforming complete conversion of the starting materials. After being stirred at room temperature for 16 h, the solvent was removed under vacuum. Crude ^{1}H NMR of the residue was recorded to determine the diastereomeric ratio. The recovered NMR sample and the remaining residue were combined and purified by column chromatography on silica gel (Pentane: EtOAc = 20:1) to give **2a** as a colorless oil in 95% yield with a 15:1 diastereomeric ratio.

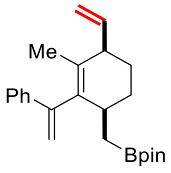

2a

4,4,5,5-Tetramethyl-2-((3-methyl-2-(1-phenylvinyl)-4-vinylcyclohex-2-en-1-yl)methyl)-1,3,2-dioxaborolane (**2a**). Following the general procedure A, **2a** was isolated as a colorless oil (69 mg, 95% yield, 15:1 dr). When the reaction was run in 2.0 mmol scale, 702 mg (96%) of **2a** was isolated. **^{1}H NMR** (400 MHz, $CDCl_3$) major isomer δ 7.44 (d, J = 6.3, 2H), 7.35 – 7.22 (m, 3H), 5.85 (ddd, J = 16.6, 10.3, 8.0 Hz,

1H), 5.66 (d, *J* = 1.8 Hz, 1H), 5.14 – 5.05 (m, 2H), 5.00 (d, *J* = 1.8 Hz, 1H), 2.76 (q, *J* = 6.1 Hz, 1H), 2.41 – 2.28 (m, 1H), 1.87 – 1.50 (m, 7H), 1.21 (s, 12H), 1.10 (dd, *J* = 15.7, 4.0 Hz, 1H), 0.69 (dd, *J* = 15.8, 11.1 Hz, 1H). **^{13}C NMR** (101 MHz, $CDCl_3$) *δ* 148.3, 142.0, 139.8, 139.4, 130.3, 128.2, 127.2, 126.2, 126.1, 114.7, 113.8, 82.7, 45.5, 34.2, 27.4, 27.3, 24.9, 24.6, 20.0. **HRMS** (ESI-TOF) m/z Calc. for $C_{24}H_{33}BO_2Na$ $(M+Na)^+$: 387.2470, found: 387.2480.

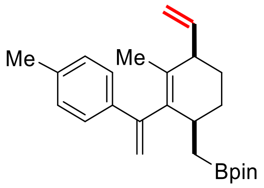

2b

4,4,5,5-Tetramethyl-2-((3-methyl-2-(1-(p-tolyl)vinyl)-4-vinylcyclohex-2-en-1-yl)methyl)-1,3,2-dioxaborolane (**2b**). Following the general procedure A, **2b** was isolated as a colorless oil (73 mg, 97% yield, 16:1 dr). **^{1}H NMR** (400 MHz, $CDCl_3$) major isomer δ 7.35 – 7.27 (m, 2H), 7.10 (d, *J* = 7.9 Hz, 2H), 5.90 – 5.75 (m, 1H), 5.60 (d, *J* = 1.8 Hz, 1H), 5.12 – 5.00 (m, 2H), 4.91 (d, *J* = 1.8 Hz, 1H), 2.73 (q, *J* = 6.1 Hz, 1H), 2.33 (s, 4H), 1.85 – 1.70 (m, 2H), 1.71 – 1.59 (m, 1H), 1.58 – 1.48 (m, 3H), 1.19 (s, 12H), 1.12 – 1.02 (m, 1H), 0.71 – 0.59 (m, 1H). **^{13}C NMR** (101 MHz, $CDCl_3$) δ 148.2, 142.2, 139.7, 137.1, 130.2, 129.1, 126.3, 114.8, 113.0, 82.9, 45.6, 34.5, 27.6, 27.5, 25.1, 24.8, 21.3, 20.1. **HRMS** (ESI-TOF) m/z Calc. for $C_{25}H_{35}BO_2Na$ $(M+Na)^+$: 401.2627, found: 401.2621.

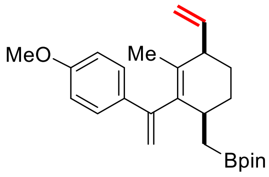

2c

2-((2-(1-(4-Methoxyphenyl)vinyl)-3-methyl-4-vinylcyclohex-2-en-1-yl)methyl)-4,4,5,5-tetramethyl-1,3,2-dioxaborolane (**2c**). Following the general procedure A, **2c** was isolated as a colorless oil (76 mg, 97% yield, 16:1 dr). **^{1}H NMR** (400 MHz, $CDCl_3$) major isomer δ 7.35 (d, *J* = 8.5 Hz, 2H), 6.83 (d, *J* = 8.4 Hz, 2H), 5.90 – 5.71 (m, 1H),

5.53 (d, J = 1.7 Hz, 1H), 5.14 – 5.00 (m, 2H), 4.86 (d, J = 1.7 Hz, 1H), 3.80 (s, 3H), 2.72 (q, J = 6.3 Hz, 1H), 2.38 – 2.22 (m, 1H), 1.85 – 1.70 (m, 2H), 1.68 – 1.61 (m, 1H), 1.58 – 1.49 (m, 4H), 1.19 (s, 12H), 1.10 – 1.03 (m, 1H), 0.68 – 0.60 (m, 1H). **^{13}C NMR** (101 MHz, $CDCl_3$) δ 159.2, 147.8, 142.2, 139.7, 132.5, 127.5, 114.8, 113.8, 112.0, 82.9, 55.4, 45.6, 34.5, 27.6, 27.5, 25.1, 24.8, 20.1. **HRMS** (ESI-TOF) m/z Calc. for $C_{25}H_{35}BO_3Na$ $(M+Na)^+$: 417.2576, found: 417.2548.

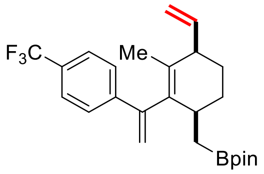

2d

4,4,5,5-Tetramethyl-2-((3-methyl-2-(1-(4-(trifluoromethyl)phenyl)vinyl)-4-vinylcyclohex-2-en-1-yl)methyl)-1,3,2-dioxaborolane (**2d**). Following the general procedure A, **2d** was isolated as a colorless oil (73 mg, 84% yield, 14:1 dr). **^{1}H NMR** (400 MHz, $CDCl_3$) major isomer δ 7.57 – 7.48 (m, 4H), 5.88 – 5.74 (m, 1H), 5.71 (d, J = 1.5 Hz, 1H), 5.11 – 5.03 (m, 3H), 2.74 (q, J = 7.0, 6.5 Hz, 1H), 2.34 – 2.24 (m, 1H), 1.84 – 1.72 (m, 2H), 1.70 – 1.61 (m, 1H), 1.58 – 1.50 (m, 4H), 1.18 (s, 12H), 1.05 – 0.98 (m, 1H), 0.68 – 0.59 (m, 1H). **^{19}F NMR** (377 MHz, $CDCl_3$) δ -62.43. **^{13}C NMR** (101 MHz, $CDCl_3$) δ 147.5, 143.6, 141.9, 138.9, 131.4, 129.5, 129.2, 126.6, 125.5 (t, J = 3.9 Hz), 116.2, 115.1, 83.0, 45.6, 34.5, 27.5, 27.4, 25.1, 24.8, 20.2. **HRMS** (ESI-TOF) m/z Calc. for $C_{25}H_{32}BF_3O_2Na$ $(M+Na)^+$: 455.2344, found: 455.2346.

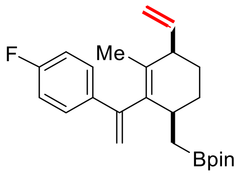

2e

2-((2-(1-(4-Fluorophenyl)vinyl)-3-methyl-4-vinylcyclohex-2-en-1-yl)methyl)-4,4,5,5-tetramethyl-1,3,2-dioxaborolane (**2e**). Following the general procedure A, **2e** was isolated as a colorless oil (67 mg, 87% yield, 14:1 dr). **^{1}H NMR** (400 MHz, $CDCl_3$) major isomer δ 7.40 – 7.33 (m, 2H), 7.01 – 6.93 (m, 2H), 5.86 – 5.76 (m, 1H), 5.56 (d, J = 1.6 Hz, 1H), 5.09 – 5.02 (m, 2H), 4.96 – 4.93 (m, 1H), 2.72 (q, J = 6.1 Hz, 1H), 2.34 – 2.24 (m, 1H), 1.82 – 1.71 (m, 2H), 1.69 – 1.58 (m, 1H), 1.58 – 1.47 (m, 4H), 1.19 (s, 12H), 1.09 – 1.00 (m, 1H), 0.68 – 0.57 (m, 1H). **^{19}F NMR** (377 MHz, $CDCl_3$)

δ -115.66. **^{13}C NMR** (101 MHz, $CDCl_3$) δ 162.5 (d, *J* = 246.1 Hz), 147.4, 142.0, 139.4, 136.04 (d, *J* = 3.2 Hz), 130.7, 128.0, 127.9, 115.3, 115.1, 115.0, 113.7 (d, *J* = 1.7 Hz), 82.9, 45.6, 34.4, 27.6, 27.4, 25.1, 24.8, 20.1. **HRMS** (ESI-TOF) m/z Calc. for $C_{24}H_{32}BFO_2Na$ $(M+Na)^+$: 405.2376, found: 405.2375.

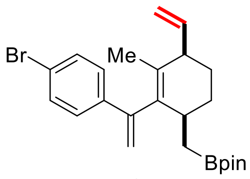

2f

2-((2-(1-(4-Bromophenyl)vinyl)-3-methyl-4-vinylcyclohex-2-en-1-yl)methyl)-4,4,5,5-tetramethyl-1,3,2-dioxaborolane (**2f**). Following the general procedure A, **2f** was isolated as a colorless oil (82 mg, 93% yield, 15:1 dr). **^{1}H NMR** (400 MHz, $CDCl_3$) major isomer δ 7.44 – 7.37 (m, 2H), 7.30 – 7.24 (m, 2H), 5.85 – 5.75 (m, 1H), 5.62 (d, *J* = 1.6 Hz, 1H), 5.08 – 5.02 (m, 2H), 4.99 (d, *J* = 1.5 Hz, 1H), 2.72 (q, *J* = 6.1 Hz, 1H), 2.33 – 2.22 (m, 1H), 1.82 – 1.71 (m, 2H), 1.68 – 1.59 (m, 1H), 1.57 – 1.48 (m, 4H), 1.19 (s, 12H), 1.06 – 0.98 (m, 1H), 0.68 – 0.56 (m, 1H). **^{13}C NMR** (101 MHz, $CDCl_3$) δ 147.5, 142.0, 139.0, 138.9, 131.5, 131.0, 128.0, 121.4, 115.0, 114.5, 83.0, 45.6, 34.5, 27.5, 27.4, 25.1, 24.8, 20.1. **HRMS** (ESI-TOF) m/z Calc. for $C_{24}H_{32}BBrO_2Na$ $(M+Na)^+$: 465.1575, found: 465.1565.

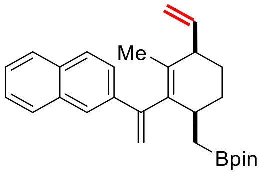

2g

4,4,5,5-tetramethyl-2-((3-methyl-2-(1-(naphthalen-2-yl)vinyl)-4-vinylcyclohex-2-en-1-yl)methyl)-1,3,2-dioxaborolane (**2g**). Following the general procedure A, **2g** was isolated as a colorless oil (79 mg, 95% yield, 15:1 dr). **^{1}H NMR** (400 MHz, $CDCl_3$) major isomer δ 7.84 – 7.75 (m, 4H), 7.64 (dt, *J* = 8.5, 1.3 Hz, 1H), 7.48 – 7.39 (m, 2H), 5.92 – 5.82 (m, 1H), 5.79 (d, *J* = 1.3 Hz, 1H), 5.15 – 5.05 (m, 3H), 2.84 – 2.75 (m, 1H), 2.46 – 2.34 (m, 1H), 1.90 – 1.77 (m, 2H), 1.74 – 1.65 (m, 1H), 1.63 – 1.52 (m, 4H), 1.17 (s, 12H), 1.14 – 1.06 (m, 1H), 0.75 – 0.63 (m, 1H). **^{13}C NMR** (101 MHz, $CDCl_3$) δ 148.4, 142.4, 139.5, 137.2, 133.7, 133.0, 130.7, 128.5, 127.9, 127.6, 126.0, 125.8,

125.5, 124.5, 114.9, 114.4, 82.9, 45.6, 34.7, 27.6, 27.5, 25.11, 24.76, 20.23. **HRMS** (ESI-TOF) m/z Calc. for $C_{28}H_{35}BO_2Na$ $(M+Na)^+$: 437.2627, found: 437.2619.

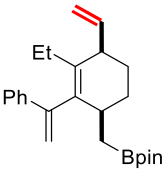

2h

2-((3-ethyl-2-(1-phenylvinyl)-4-vinylcyclohex-2-en-1-yl)methyl)-4,4,5,5-tetramethyl-1,3,2-dioxaborolane (**2h**). Following the general procedure A, **2h** was isolated as a colorless oil (73 mg, 96% yield, 9:1 dr). ^{1}**H NMR** (400 MHz, $CDCl_3$) major isomer δ 7.48 – 7.41 (m, 2H), 7.32 – 7.19 (m, 3H), 5.85 (ddd, *J* = 17.0, 10.1, 8.1 Hz, 1H), 5.66 (d, *J* = 1.8 Hz, 1H), 5.11 – 5.02 (m, 2H), 4.98 (d, *J* = 1.7 Hz, 1H), 2.92 (q, *J* = 6.4, 5.9 Hz, 1H), 2.38 – 2.27 (m, 1H), 2.22 – 2.11 (m, 1H), 1.91 – 1.62 (m, 4H), 1.59 – 1.46 (m, 1H), 1.18 (s, 12H), 1.08 – 1.01 (m, 1H), 0.86 (t, *J* = 7.5 Hz, 3H), 0.67 – 0.56 (m, 1H). ^{13}**C NMR** (101 MHz, $CDCl_3$) δ 148.0, 142.5, 139.9, 138.9, 136.3, 128.4, 127.4, 126.3, 114.8, 113.6, 82.9, 41.7, 28.0, 27.5, 25.8, 25.1, 24.8, 13.5. **HRMS** (ESI-TOF) m/z Calc. for $C_{25}H_{35}BO_2Na$ $(M+Na)^+$: 401.2627, found: 401.2628.

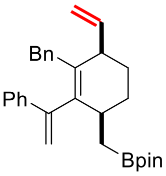

2i

2-((3-benzyl-2-(1-phenylvinyl)-4-vinylcyclohex-2-en-1-yl)methyl)-4,4,5,5-tetramethyl-1,3,2-dioxaborolane (**2i**). Following the general procedure A, **2i** was isolated as a colorless oil (83 mg, 94% yield, 11:1 dr). ^{1}**H NMR** (400 MHz, $CDCl_3$) major isomer δ 7.54 – 7.49 (m, 2H), 7.34 – 7.28 (m, 2H), 7.28 – 7.24 (m, 1H), 7.24 – 7.07 (m, 5H), 5.87 (ddd, *J* = 17.1, 10.1, 8.0 Hz, 1H), 5.76 (d, *J* = 1.5 Hz, 1H), 5.14 (d, *J* = 1.5 Hz, 1H), 5.08 (dd, *J* = 10.0, 2.0 Hz, 1H), 5.00 (ddd, *J* = 17.1, 2.0, 1.0 Hz, 1H), 3.61 (d, *J* = 14.8 Hz, 1H), 3.12 – 3.02 (m, 1H), 2.77 – 2.68 (m, 1H), 2.60 – 2.50 (m, 1H), 1.86 – 1.76 (m, 1H), 1.74 – 1.50 (m, 3H), 1.21 (s, 12H), 1.15 – 1.08 (m, 1H), 0.72 – 0.63 (m, 1H). ^{13}**C NMR** (101 MHz, $CDCl_3$) δ 148.3, 141.9, 141.2, 140.9, 140.1, 133.8, 129.08, 128.4, 128.2, 127.6, 126.3, 125.6, 115.4, 114.0, 83.0, 40.8, 38.6, 28.0, 27.3,

25.1, 24.8. **HRMS** (ESI-TOF) m/z Calc. for $C_{30}H_{37}BO_2Na$ $(M+Na)^+$: 463.2784, found: 463.2776.

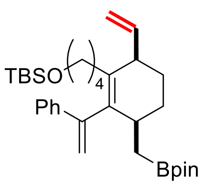

2j

***tert*-Butyldimethyl(4-(2-(1-phenylvinyl)-3-((4,4,5,5-tetramethyl-1,3,2-dioxaborolan-2-yl)methyl)-6-vinylcyclohex-1-en-1-yl)butoxy)silane** (**2j**). Following the general procedure A, **2j** was isolated as a colorless oil (105 mg, 98% yield, 7:1 dr). **^{1}H NMR** (400 MHz, $CDCl_3$) major isomer δ 7.47 – 7.37 (m, 2H), 7.32 – 7.18 (m, 3H), 5.83 (ddd, *J* = 17.1, 10.3, 8.1 Hz, 1H), 5.64 (d, *J* = 1.7 Hz, 1H), 5.10 – 5.02 (m, 1H), 5.05 – 4.94 (m, 2H), 3.52 – 3.42 (m, 2H), 2.87 (t, *J* = 6.6 Hz, 1H), 2.34 (d, *J* = 10.7 Hz, 1H), 2.10 (d, *J* = 13.4 Hz, 1H), 1.85 – 1.69 (m, 3H), 1.66 (dd, *J* = 8.3, 4.1 Hz, 1H), 1.55 (s, 3H), 1.53 – 1.46 (m, 1H), 1.39 – 1.23 (m, 4H), 1.18 (s, 12H), 1.07 – 1.00 (m, 1H), 0.85 (s, 9H), 0.66 – 0.56 (m, 1H), -0.01 (s, 6H). **^{13}C NMR** (101 MHz, $CDCl_3$) δ 148.1, 142.5, 140.0, 139.5, 134.9, 128.3, 127.4, 126.3, 114.9, 113.7, 82.9, 63.3, 42.2, 33.2, 32.7, 27.9, 27.4, 26.1, 25.1, 25.0, 24.8, 18.5, -5.1. **HRMS** (ESI-TOF) m/z Calc. for $C_{33}H_{53}BO_3SiNa$ $(M+Na)^+$: 559.3755, found: 559.3757.

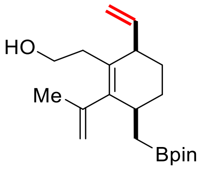

2k

2-(2-(Prop-1-en-2-yl)-3-((4,4,5,5-tetramethyl-1,3,2-dioxaborolan-2-yl)methyl)-6-vinylcyclohex-1-en-1-yl)ethan-1-ol (**2k**). Following the general procedure A, **2k** was isolated as a colorless oil (23.1mg, 70% yield, 5:1 dr) **^{1}H NMR** (400 MHz, $CDCl_3$) major isomer δ = 5.84 – 5.73 (m, 1H), 5.06 – 4.96 (m, 3H), 4.65 (dd, *J* = 2.5, 1.1 Hz, 1H), 3.59 (dd, *J* = 7.8, 6.1 Hz, 2H), 2.74 (dq, *J* = 9.5, 5.1 Hz, 1H), 2.57 – 2.36 (m, 2H), 2.16 (dt, *J* = 12.8, 6.1 Hz, 1H), 1.81 (d, *J* = 1.3 Hz, 3H), 1.76 – 1.38 (m, 4H), 1.23 (d, *J* = 2.4 Hz, 12H), 1.11 (dd, *J* = 15.7, 4.4 Hz, 1H), 0.65 (dd, *J* = 15.7, 10.1 Hz, 1H); minor isomer (distinct peaks) δ = 5.68 – 4.67 (m, 1H), 1.78 (d, *J* = 1.1 Hz, 3H), 0.85 (dd, *J* = 16.0, 8.8 Hz, 1H). **^{13}C NMR** (101 MHz, $CDCl_3$) major isomers δ = 145.4, 145.2, 142.1,

127.0, 115.0, 114.3, 82.9, 61.5, 42.3, 35.9, 33.7, 27.7, 27.2, 25.0, 24.7, 23.9; minor isomers (distinct peaks) δ = 145.0, 144.9, 141.9, 126.6, 114.8, 114.3 (overlapped), 82.9, 61.4, 41.6, 34.8, 32.3, 26.9, 26.3, 24.8, 24.7, 23.9. **HRMS** (ESI-TOF) m/z Cal. for $C_{20}H_{33}BNaO_4$ $(M+Na)^+$: 355.2419, found: 355.2419.

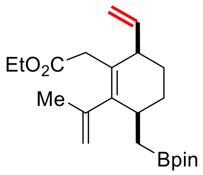

2l

Ethyl 2-(2-(prop-1-en-2-yl)-3-((4,4,5,5-tetramethyl-1,3,2-dioxaborolan-2-yl)methyl)-6-vinylcyclohex-1-en-1-yl)acetate (**2l**). Following the general procedure A, **2l** was isolated as a colorless oil (36.6mg, 98% yield, 14:1 dr) **^{1}H NMR** (400 MHz, $CDCl_3$) major isomer δ = 5.78 – 5.65 (m, 1H), 5.03 – 4.93 (m, 3H), 4.63 (d, J = 2.6 Hz, 1H), 4.05 (q, J = 7.1 Hz, 2H), 3.30 (d, J = 15.9 Hz, 1H), 2.88 (d, J = 15.9 Hz, 1H), 2.76 (q, J = 6.1 Hz, 1H), 2.44 (tt, J = 10.5, 5.3 Hz, 1H), 1.81 – 1.67 (m, 5H), 1.62 – 1.34 (m, 2H), 1.25 – 1.18 (m, 15H), 1.09 (dd, J = 15.8, 4.5 Hz, 1H), 0.64 (dd, J = 15.8, 10.1 Hz, 1H); minor isomer (distinct peaks) δ = 3.11 (d, J = 16.2 Hz, 1H), 2.94 (d, J = 16.2, 1H), 0.73 (dd, J = 15.9, 10.4 Hz, 1H). **^{13}C NMR** (101 MHz, $CDCl_3$) δ = 172.6, 146.0, 145.0, 141.5, 124.3, 115.3, 114.4, 82.9, 60.2, 43.2, 38.0, 32.9, 27.3, 27.2, 25.0, 24.7, 23.0, 14.2. **HRMS** (ESI-TOF) m/z Cal. for $C_{22}H_{36}BO_4$ $(M+H)^+$: 375.2705, found: 375.2696.

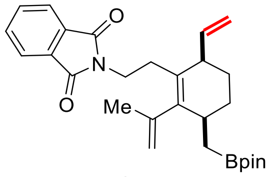

2m

2-(2-(2-(Prop-1-en-2-yl)-3-((4,4,5,5-tetramethyl-1,3,2-dioxaborolan-2-yl)methyl)-6-vinylcyclohex-1-en-1-yl)ethyl)isoindoline-1,3-dione (**2m**). Following the general procedure A, **2m** was isolated as a colorless oil (33.0 mg, 71% yield, 17:1 dr) **^{1}H NMR** (400 MHz, $CDCl_3$) δ = 7.80 (dd, J = 5.4, 3.0 Hz, 2H), 7.67 (dd, J = 5.4, 3.0 Hz, 2H), 5.77 (ddd, J = 17.0, 10.1, 8.0 Hz, 1H), 5.10 – 5.01 (m, 2H), 4.87 (dd, J = 2.5, 1.5 Hz, 1H), 4.41 (d, J = 2.4 Hz, 1H), 3.79 – 3.57 (m, 2H), 2.99 – 2.92 (m, 1H), 2.71 (dt, J = 13.3, 8.3 Hz, 1H), 2.35 – 2.25 (m, 1H), 2.15 – 2.06 (m, 1H), 1.75 – 1.64 (m, 5H), 1.60 – 1.53 (m, 1H), 1.47 – 1.35 (m, 1H), 1.21 (d, J = 2.1 Hz, 12H), 1.06 (dd, J = 15.7, 4.3 Hz, 1H), 0.58 (dd, J = 15.7, 10.5 Hz, 1H). **^{13}C NMR** (101 MHz, $CDCl_3$) δ = 168.2, 145.1, 145.0, 141.9, 133.7, 132.3, 127.4, 123.0, 115.2, 114.2, 82.9, 41.6, 36.8, 34.1,

31.2, 27.3, 27.2, 25.0, 24.7, 23.8. **HRMS** (ESI-TOF) m/z Cal. for $C_{28}H_{37}BNO_4$ $(M+H)^+$: 464.2815, found: 464.2809.

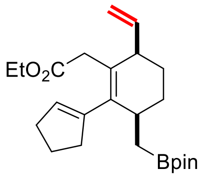

2n

Ethyl 2-(2-(cyclopent-1-en-1-yl)-3-((4,4,5,5-tetramethyl-1,3,2-dioxaborolan-2-yl)methyl)-6-vinylcyclohex-1-en-1-yl)acetate (**2n**). Following the general procedure A, **2n** was isolated as a colorless oil (33.6 mg, 84% yield, 12:1 dr) **^{1}H NMR** (400 MHz, $CDCl_3$) δ = 5.79 – 5.67 (m, 1H), 5.41 – 5.38 (m, 1H), 5.06 – 4.97 (m, 2H), 4.06 (q, J = 7.1 Hz, 2H), 3.21 (dd, J = 15.8, 0.8 Hz, 1H), 2.91 (d, J = 15.8 Hz, 1H), 2.82 – 2.74 (m, 1H), 2.51 – 2.29 (m, 4H), 2.25 – 2.16 (m, 1H), 1.92 – 1.66 (m, 4H), 1.63 – 1.41 (m, 2H), 1.27 – 1.19 (m, 15H), 1.05 (dd, J = 15.8, 3.7 Hz, 1H), 0.64 (dd, J = 15.8, 11.1 Hz, 1H). **^{13}C NMR** (101 MHz, $CDCl_3$) δ = 172.7, 143.5, 141.6 (overlapped, 2C), 128.1, 125.2, 115.1, 82.8, 60.1, 43.8, 38.3, 35.8, 33.5, 32.8, 27.1, 27.0, 25.0, 24.6, 23.4, 14.2. **HRMS** (ESI-TOF) m/z Cal. for $C_{24}H_{41}BNO_4$ $(M+H_4N)^+$: 418.3127, found: 418.3106.

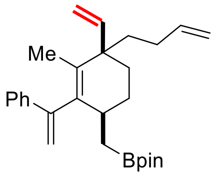

2o

2-((4-(But-3-en-1-yl)-3-methyl-2-(1-phenylvinyl)-4-vinylcyclohex-2-en-1-yl) methyl)-4,4,5,5-tetramethyl-1,3,2-dioxaborolane (**2o**). Following the general procedure A using **L8** as ligand, **2o** was isolated as a colorless oil (39.0 mg, 93% yield, >20:1dr) **^{1}H NMR** (400 MHz, $CDCl_3$) δ = 7.46 – 7.39 (m, 2H), 7.34 – 7.27 (m, 2H), 7.27 – 7.20 (m, 1H), 5.92 – 5.72 (m, 2H), 5.65 (d, J = 1.7 Hz, 1H), 5.09 – 5.00 (m, 2H), 4.98 – 4.95 (m, 2H), 4.94 – 4.91 (m, 1H), 2.32 – 2.12 (m, 2H), 1.98 – 1.81 (m, 3H), 1.76 – 1.66 (m, 1H), 1.58 – 1.41 (m, 5H), 1.36 – 1.28 (m, 1H), 1.25 – 1.11 (m, 13H), 0.84 (dd, J = 15.9, 11.5 Hz, 1H). **^{13}C NMR** (101 MHz, $CDCl_3$) δ = 148.8, 145.0, 142.8, 139.6, 139.4, 131.4, 128.3, 127.3, 126.2, 114.0, 114.0, 112.7, 82.8, 45.1, 36.8, 33.7, 28.5, 27.6, 25.0, 24.8, 24.5, 16.1. **HRMS** (ESI-TOF) m/z Cal. for $C_{24}H_{38}BO_4$ $(M+H_4N)^+$: 418.3127, found: 418.3106.

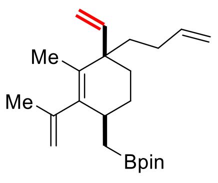

2p

2-((4-(But-3-en-1-yl)-3-methyl-2-(prop-1-en-2-yl)-4-vinylcyclohex-2-en-1-yl)methyl)-4,4,5,5-tetramethyl-1,3,2-dioxaborolane (**2p**). Following the general procedure A using **L8** as ligand, **2p** was isolated as a colorless oil (68 mg, 95% yield, >20:1 dr). **^{1}H NMR** (400 MHz, $CDCl_3$) major isomer δ 5.89 – 5.75 (m, 1H), 5.71 – 5.60 (m, 1H), 5.04 – 4.87 (m, 4H), 4.80 (dd, *J* = 17.4, 1.8 Hz, 1H), 4.58 (d, *J* = 2.5 Hz, 1H), 2.40 – 2.30 (m, 1H), 2.16 – 2.03 (m, 1H), 1.90 – 1.72 (m, 6H), 1.69 – 1.59 (m, 1H), 1.55 (s, 1H), 1.48 (s, 3H), 1.44 – 1.35 (m, 2H), 1.23 (d, *J* = 4.3 Hz, 12H), 1.15 – 1.06 (m, 1H), 0.79 – 0.69 (m, 1H). **^{13}C NMR** (101 MHz, $CDCl_3$) δ 146.5, 145.2, 145.0, 139.6, 127.4, 114.9, 114.9, 113.8, 112.7, 83.0, 44.8, 36.8, 32.5, 28.4, 27.6, 25.3, 25.1, 24.8, 23.1, 15.7. **HRMS** (ESI-TOF) m/z Calc. for $C_{23}H_{37}BO_2Na$ $(M+Na)^+$: 379.2783, found: 379.2782.

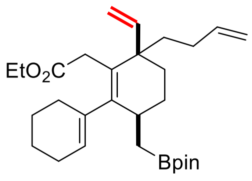

2q

Ethyl 2-(3-(but-3-en-1-yl)-6-((4,4,5,5-tetramethyl-1,3,2-dioxaborolan-2-yl)methyl)-3-vinyl-[1,1'-bi(cyclohexane)]-1,1'-dien-2-yl)acetate (**2q**). Following the general procedure A using **L8** as ligand, **2q** was isolated as a colorless oil (36.6 mg, 78% yield, >20:1 dr). **^{1}H NMR** (400 MHz, $CDCl_3$) *δ* = 5.87 – 5.67 (m, 2H), 5.39 – 5.35 (m, 1H), 5.06 – 4.83 (m, 5H), 4.02 (q, *J* = 7.1 Hz, 2H), 3.11 (d, *J* = 16.6, 1H), 2.81 (d, *J* = 16.6 Hz, 1H), 2.43 – 2.35 (m, 1H), 2.13 – 1.71 (m, 8H), 1.65 – 1.29 (m, 8H), 1.28 – 1.18 (m, 15H), 1.10 (dd, *J* = 16.0, 3.8 Hz, 1H), 0.74 (dd, *J* = 15.9, 11.5 Hz, 1H). **^{13}C NMR** (101 MHz, $CDCl_3$) *δ* = 172.9, 149.0, 144.2, 139.3, 138.3, 126.1, 125.8, 114.0, 113.0, 82.9, 60.0, 44.7, 36.4, 35.7, 28.5, 28.4, 27.6, 25.2, 25.0, 24.8, 24.6, 22.9, 22.1, 14.1. **HRMS** (ESI-TOF) m/z Cal. for $C_{29}H_{49}BNO_4$ $(M+H_4N)^+$: 486.3754, found: 486.3742.

3.2 General Procedure B: Synthesis of Cyclobutene 3

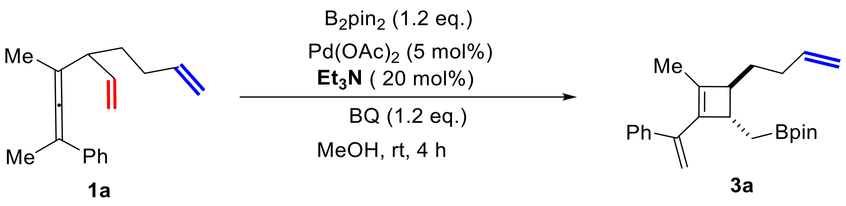

To an 8 mL vial charged with a stirring bar were added $Pd(OAc)_2$ (2.2 mg, 5 mol%), MeOH (2.0 mL) and Et_3N (5.6 uL, 20 mol%) sequentially. Then **1a** (47. 6 mg, 0.2 mmol) and B_2pin_2 (61.0 mg, 1.2 eq.) and BQ (26.0 mg, 1.1 equiv.) were added to the mixture. (TLC analysis was conducted for each reaction, conforming complete conversion of the starting materials.) After being stirred at room temperature for 4 h, the solvent was removed under vacuum. Crude ^1H NMR of the residue was recorded to determine the diastereomeric ratio. The recovered NMR sample and the remaining residue were combined and purified by column chromatography on silica gel (Pentane: EtOAc= 20:1) to give **3a** in 79% yield and with a 9:1 diastereomeric ratio.

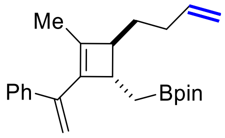

3a

2-((4-(But-3-en-1-yl)-3-methyl-2-(1-phenylvinyl)cyclobut-2-en-1-yl)methyl) 4,4,5,5-tetramethyl-1,3,2-dioxaborolane (**3a**). Following the general procedure B, **3a** was isolated as a colorless oil (63 mg, 86% yield, 9:1dr). **^{1}H NMR** (400 MHz, $CDCl_3$) δ = 7.41 – 7.23 (m, 5H), 5.95 – 5.82 (m, 1H), 5.18 – 4.90 (m, 4H), 2.72 – 2.60 (m, 1H), 2.28 – 2.09 (m, 3H), 1.77 – 1.48 (m, 2H), 1.36 – 1.23 (m, 16H), 0.96 (dd, J = 15.6, 9.3 Hz, 1H). **^{13}C NMR** (101 MHz, $CDCl_3$) δ = 143.7, 142.5, 141.9, 141.2, 139.4, 127.9, 127.8, 127.1, 114.0, 113.3, 82.9, 50.1, 41.7, 32.1, 31.7, 25.0, 24.9, 13.8. **HRMS** (ESI-TOF) m/z Cal. for $C_{24}H_{33}BO_2Na$ $(M+Na)^+$: 387.2470, found: 387.2463.

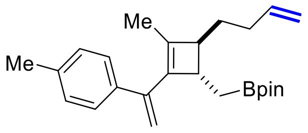

3b

2-((4-(But-3-en-1-yl)-3-methyl-2-(1-(p-tolyl)vinyl)cyclobut-2-en-1-yl)methyl)-

4,4,5,5-tetramethyl-1,3,2-dioxaborolane (**3b**). Following the general procedure B, **3b** was isolated as a colorless oil (60 mg, 79% yield, 14:1 dr). **^{1}H NMR** (400 MHz, $CDCl_3$) major isomer δ 7.26 – 7.22 (m, 2H), 7.15 – 7.07 (m, 2H), 5.92 – 5.79 (m, 1H), 5.13 – 4.89 (m, 4H), 2.68 – 2.57 (m, 1H), 2.35 (s, 3H), 2.24 – 2.10 (m, 3H), 1.72 – 1.62 (m, 1H), 1.58 – 1.49 (m, 1H), 1.34 (t, J = 1.7 Hz, 3H), 1.29 – 1.16 (m, 13H), 0.95 – 0.87 (m, 1H). **^{13}C NMR** (101 MHz, $CDCl_3$) δ 143.6, 142.5, 142.2, 139.6, 138.4, 136.9, 128.7, 128.0, 114.1, 113.0, 83.0, 50.2, 41.9, 32.3, 31.9, 25.2, 25.0, 21.3, 14.0. **HRMS** (ESI-TOF) m/z Calc. for $C_{25}H_{35}BO_2Na$ $(M+Na)^+$: 401.2627, found: 401.2615.

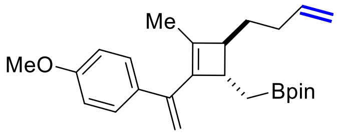

3c

2-((4-(But-3-en-1-yl)-2-(1-(4-methoxyphenyl)vinyl)-3-methylcyclobut-2-en-1-yl)methyl)-4,4,5,5-tetramethyl-1,3,2-dioxaborolane (**3c**). Following the general procedure B, **3c** was isolated as a colorless oil (64 mg, 82% yield, 14:1 dr). **^{1}H NMR** (400 MHz, $CDCl_3$) major isomer δ 7.30 – 7.26 (m, 2H), 6.87 – 6.82 (m, 2H), 5.92 – 5.79 (m, 1H), 5.09 – 4.89 (m, 4H), 3.81 (s, 3H), 2.67 – 2.57 (m, 1H), 2.24 – 2.10 (m, 3H), 1.73 – 1.61 (m, 1H), 1.57 – 1.47 (m, 1H), 1.35 (t, J = 1.7 Hz, 3H), 1.29 – 1.20 (m, 13H), 0.96 – 0.87 (m, 1H). **^{13}C NMR** (101 MHz, $CDCl_3$) δ 159.1, 143.2, 142.5, 142.3, 139.6, 133.8, 129.1, 114.1, 113.4, 112.5, 83.0, 55.4, 50.1, 41.9, 32.3, 31.9, 25.1, 25.0, 14.0. **HRMS** (ESI-TOF) m/z Calc. for $C_{25}H_{35}BO_3Na$ $(M+Na)^+$: 417.2576, found: 417.2559.

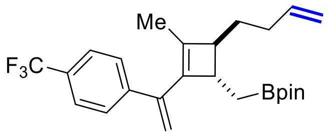

3d

2-((4-(But-3-en-1-yl)-3-methyl-2-(1-(4-(trifluoromethyl)phenyl)vinyl)cyclobut-2-en-1-yl)methyl)-4,4,5,5-tetramethyl-1,3,2-dioxaborolane (**3d**). Following the general procedure B, **3d** was isolated as a colorless oil (69 mg, 80% yield, 9:1 dr). **^{1}H NMR** (400 MHz, $CDCl_3$) major isomer δ 7.59 – 7.54 (m, 2H), 7.49 – 7.43 (m, 2H), 5.92 – 5.79 (m, 1H), 5.20 – 4.91 (m, 4H), 2.68 – 2.61 (m, 1H), 2.24 – 2.07 (m, 3H),

1.73 – 1.62 (m, 1H), 1.57 – 1.48 (m, 1H), 1.31 (t, *J* = 1.7 Hz, 3H), 1.28 – 1.12 (m, 13H), 0.98 – 0.90 (m, 1H). **^{19}F NMR** (101 MHz, $CDCl_3$) δ -62.36. **^{13}C NMR** (101 MHz, $CDCl_3$) δ 145.1, 144.6, 143.3, 142.8, 141.4, 139.4, 129.4 (q, *J* = 32.2 Hz), 128.4, 125.0 (q, *J* = 3.7 Hz), 114.8, 114.2, 83.1, 50.3, 41.9, 32.2, 31.8, 25.1, 25.0, 14.0. **HRMS** (ESI-TOF) m/z Calc. for $C_{25}H_{32}BF_3O_2Na$ $(M+Na)^+$: 455.2344, found: 455.2347.

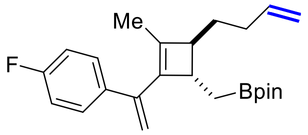

3e

2-((4-(But-3-en-1-yl)-2-(1-(4-fluorophenyl)vinyl)-3-methylcyclobut-2-en-1-yl)methyl)-4,4,5,5-tetramethyl-1,3,2-dioxaborolane (**3e**). Following the general procedure B, **3e** was isolated as a colorless oil (63 mg, 82% yield, 9:1 dr). **^{1}H NMR** (400 MHz, $CDCl_3$) major isomer δ 7.34 – 7.28 (m, 2H), 7.06 – 6.91 (m, 2H), 5.92 – 5.77 (m, 1H), 5.12 – 4.91 (m, 4H), 2.68 – 2.56 (m, 1H), 2.26 – 2.01 (m, 3H), 1.72 – 1.61 (m, 1H), 1.56 – 1.46 (m, 1H), 1.32 (t, *J* = 1.7 Hz, 3H), 1.28 – 1.10 (m, 13H), 0.96 – 0.87 (m, 1H). **^{19}F NMR** (101 MHz, $CDCl_3$) δ -115.70. **^{13}C NMR** (101 MHz, $CDCl_3$) δ 162.4 (d, *J* = 245.4 Hz), 142.8 (d, *J* = 2.5 Hz), 142.0, 139.5, 137.4 (d, *J* = 3.3 Hz), 129.6 (d, *J* = 7.9 Hz), 114.8 (d, *J* = 21.3 Hz), 114.2, 113.4, 83.1, 50.2, 41.9, 32.3, 31.8, 25.1, 25.0, 14.0. **HRMS** (ESI-TOF) m/z Calc. for $C_{24}H_{32}BFO_2Na$ $(M+Na)^+$: 405.2376, found: 405.2373.

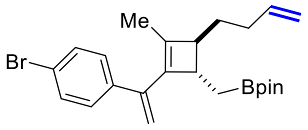

3f

2-((2-(1-(4-Bromophenyl)vinyl)-4-(but-3-en-1-yl)-3-methylcyclobut-2-en-1-yl)methyl)-4,4,5,5-tetramethyl-1,3,2-dioxaborolane (**3f**). Following the general procedure B, **3f** was isolated as a colorless oil (76 mg, 86% yield, 7:1 dr). **^{1}H NMR** (400 MHz, $CDCl_3$) major isomer δ 7.45 – 7.40 (m, 2H), 7.24 – 7.19 (m, 2H), 5.91 – 5.79 (m, 1H), 5.12 – 4.91 (m, 4H), 2.67 – 2.55 (m, 1H), 2.24 – 2.06 (m, 3H), 1.73 – 1.60 (m, 1H), 1.55 – 1.46 (m, 1H), 1.33 (t, *J* = 1.7 Hz, 3H), 1.28 – 1.12 (m, 13H), 0.95 – 0.87 (m, 1H). **^{13}C NMR** (101 MHz, $CDCl_3$) δ 143.1, 142.7, 141.6, 140.3, 139.4, 131.2,

129.8, 121.3, 114.2, 113.9, 83.1, 50.2, 41.9, 32.2, 31.8, 25.1, 25.0, 14.1. **HRMS** (ESI-TOF) m/z Calc. for $C_{24}H_{32}BBrO_2Na$ $(M+Na)^+$: 465.1575, found: 465.1580.

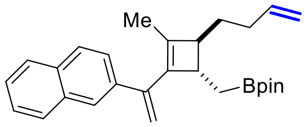

3g

2-((4-(But-3-en-1-yl)-3-methyl-2-(1-(naphthalen-2-yl)vinyl)cyclobut-2-en-1-yl)methyl)-4,4,5,5-tetramethyl-1,3,2-dioxaborolane (**3g**). Following the general procedure B, **3g** was isolated as a colorless oil (63 mg, 76% yield, 8:1 dr). **^{1}H NMR** (400 MHz, $CDCl_3$) major isomer δ 7.86 – 7.74 (m, 4H), 7.53 – 7.41 (m, 3H), 5.94 – 5.81 (m, 1H), 5.27 – 5.16 (m, 2H), 5.08 – 4.92 (m, 2H), 2.74 – 2.64 (m, 1H), 2.27 – 2.09 (m, 3H), 1.75 – 1.63 (m, 1H), 1.61 – 1.51 (m, 1H), 1.31 (s, 3H), 1.28 – 1.17 (m, 13H), 1.03 – 0.95 (m, 1H). **^{13}C NMR** (101 MHz, $CDCl_3$) δ 143.8, 142.9, 142.05, 139.5, 138.9, 133.5, 132.9, 128.2, 127.7, 127.4, 126.8, 126.6, 126.1, 125.8, 114.2, 113.9, 83.1, 50.3, 42.0, 32.3, 31.9, 25.2, 25.1, 14.1. **HRMS** (ESI-TOF) m/z Calc. for $C_{28}H_{35}BO_2Na$ $(M+Na)^+$: 437.2627, found: 437.2621.

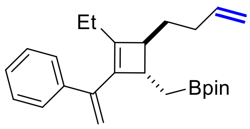

3h

2-((4-(But-3-en-1-yl)-3-ethyl-2-(1-phenylvinyl)cyclobut-2-en-1-yl)methyl)-4,4,5,5-tetramethyl-1,3,2-dioxaborolane (**3h**). Following the general procedure B, **3h** was isolated as a colorless oil (58 mg, 77% yield, 7:1 dr). **^{1}H NMR** (400 MHz, $CDCl_3$) major isomer δ 7.38 – 7.26 (m, 5H), 5.95 – 5.79 (m, 1H), 5.18 – 4.89 (m, 4H), 2.65 – 2.55 (m, 1H), 2.30 – 2.07 (m, 3H), 1.69 (dddd, J = 9.5, 8.3, 6.8, 3.6 Hz, 3H), 1.56 – 1.46 (m, 1H), 1.30 – 1.17 (m, 13H), 0.97 – 0.89 (m, 1H), 0.80 (t, J = 7.6 Hz, 3H). **^{13}C NMR** (101 MHz, $CDCl_3$) δ 148.0, 143.8, 141.6, 141.1, 139.6, 128.0, 127.9, 127.3, 114.1, 113.6, 83.1, 48.2, 41.5, 32.3, 32.1, 25.2, 25.1, 20.9, 11.7. **HRMS** (ESI-TOF) m/z Calc. for $C_{25}H_{35}BO_2Na$ $(M+Na)^+$: 401.2627, found: 401.2625.

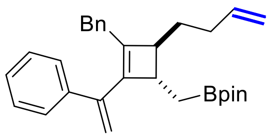

3i

2-((3-Benzyl-4-(but-3-en-1-yl)-2-(1-phenylvinyl)cyclobut-2-en-1-yl)methyl)-4,4,5,5-tetramethyl-1,3,2-dioxaborolane (**3i**). Following the general procedure B, **3i** was isolated as a colorless oil (77 mg, 88% yield, 20:1 dr). **^{1}H NMR** (400 MHz, $CDCl_3$) major isomer δ 7.44 – 7.37 (m, 2H), 7.31 – 7.19 (m, 5H), 7.18 – 7.12 (m, 1H), 7.07 – 7.02 (m, 2H), 5.83 – 5.71 (m, 1H), 5.21 – 5.15 (m, 2H), 4.96 – 4.84 (m, 2H), 3.15 – 3.00 (m, 2H), 2.74 – 2.64 (m, 1H), 2.30 – 2.20 (m, 1H), 2.15 – 1.95 (m, 2H), 1.65 – 1.56 (m, 1H), 1.48 – 1.36 (m, 1H), 1.32 – 1.26 (m, 1H), 1.23 (d, *J* = 1.9 Hz, 12H), 0.98 (dd, *J* = 15.7, 9.2 Hz, 1H). **^{13}C NMR** (101 MHz, $CDCl_3$) δ 144.0, 143.6, 143.6, 141.3, 139.4, 139.1, 128.9, 128.3, 128.1, 128.0, 127.4, 125.8, 114.4, 114.0, 83.1, 48.9, 42.0, 33.9, 32.1, 31.9, 25.1, 25.0. **HRMS** (ESI-TOF) m/z Calc. for $C_{30}H_{37}BO_2Na$ $(M+Na)^+$: 463.2784, found: 463.2781.

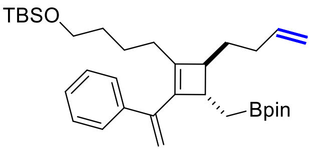

3j

(4-(4-(But-3-en-1-yl)-2-(1-phenylvinyl)-3-((4,4,5,5-tetramethyl-1,3,2-dioxaborolan-2-yl)methyl)cyclobut-1-en-1-yl)butoxy)(tert-butyl)dimethylsilane (**3j**). Following the general procedure B, **3j** was isolated as a colorless oil (70 mg, 65% yield, 7:1 dr). **^{1}H NMR** (400 MHz, $CDCl_3$) major isomer δ 7.37 – 7.19 (m, 5H), 5.94 – 5.79 (m, 1H), 5.16 – 4.90 (m, 4H), 3.53 – 3.38 (m, 2H), 2.65 – 2.57 (m, 1H), 2.29 – 2.03 (m, 3H), 1.78 – 1.46 (m, 4H), 1.36 – 1.11 (m, 17H), 0.96 – 0.82 (m, 10H), 0.02 (s, 6H). **^{13}C NMR** (101 MHz, $CDCl_3$) δ 146.7, 143.9, 141.9, 141.6, 139.5, 128.0, 127.3, 114.1, 113.6, 83.0, 63.1, 48.7, 41.7, 33.0, 32.4, 32.2, 27.5, 26.1, 25.2, 25.1, 23.8, 18.5, -5.1. **HRMS** (ESI-TOF) m/z Calc. for $C_{33}H_{53}BO_3SiNa$ $(M+Na)^+$: 559.3755, found: 559.3758.

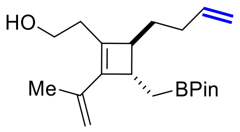

3k

2-(4-(But-3-en-1-yl)-2-(prop-1-en-2-yl)-3-((4,4,5,5-tetramethyl-1,3,2-dioxaborolan-2-yl)methyl)cyclobut-1-en-1-yl)ethan-1-ol (**3k**). Following the general procedure B using 20 mol% DABCO as ligand, **3k** was isolated as a colorless oil (19.4 mg, 58% yield, >20:1 dr). **^{1}H NMR** (400 MHz, $CDCl_3$) δ = 5.83 (ddt, J = 16.9, 10.2, 6.6 Hz, 1H), 5.01 (dq, J = 17.1, 1.8 Hz, 1H), 4.94 (dd, J = 10.2, 1.9 Hz, 1H), 4.80 (s, 2H), 3.78 – 3.63 (m, 2H), 2.72 (ddd, J = 14.9, 9.6, 5.8 Hz, 1H), 2.59 – 2.49 (m, 2H), 2.29 – 2.03 (m, 4H), 1.95 (s, 3H), 1.75 – 1.65 (m, 1H), 1.47 – 1.35 (m, 1H), 1.31 – 1.16 (m, 14H). **^{13}C NMR** (101 MHz, $CDCl_3$) δ = 145.8, 140.6, 139.1, 139.0, 114.2, 112.9, 83.0, 60.4, 46.7, 40.7, 32.1, 31.9, 31.4, 24.6, 24.5, 21.3. **HRMS** (ESI-TOF) m/z Cal. for $C_{20}H_{33}BNaO_3$ $(M+Na)^+$: 355.2419, found: 355.2414.

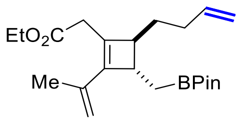

3l

Ethyl 2-(4-(but-3-en-1-yl)-2-(prop-1-en-2-yl)-3-((4,4,5,5-tetramethyl-1,3,2-dioxaborolan-2-yl)methyl)cyclobut-1-en-1-yl)acetate (**3l**). Following the general procedure B using 20 mol% DABCO as ligand, **3a** was isolated as a colorless oil (30.4 mg, 81% yield, 16:1 dr). **^{1}H NMR** (400 MHz, $CDCl_3$) δ = 5.84 (ddt, J = 16.9, 10.2, 6.6 Hz, 1H), 5.00 (dq, J = 17.1, 1.7 Hz, 1H), 4.92 (ddt, J = 10.2, 2.4, 1.3 Hz, 1H), 4.89 – 4.84 (m, 2H), 4.13 (q, J = 7.2 Hz, 2H), 3.30 (d, J = 15.2 Hz, 1H), 3.15 (d, J = 15.2, 1H), 2.55 – 2.45 (m, 1H), 2.27 – 2.05 (m, 3H), 1.95 – 1.89 (m, 3H), 1.83 – 1.63 (m, 1H), 1.54 – 1.43 (m, 1H), 1.39 – 1.18 (m, 16H), 0.84 (dd, J = 15.8, 9.9 Hz, 1H). **^{13}C NMR** (101 MHz, $CDCl_3$) δ = 170.4, 146.3, 139.3, 138.2, 134.8, 113.9, 113.7, 82.9, 60.6, 49.3, 41.2, 34.7, 32.0, 31.6, 24.9, 24.9, 21.06, 14.2. **HRMS** (ESI-TOF) m/z Cal. for $C_{22}H_{35}BNaO_4$ $(M+Na)^+$: 397.2525, found: 397.2530.

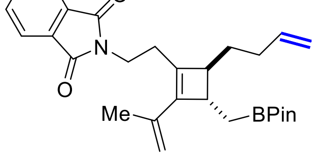

3m

2-(2-(4-(But-3-en-1-yl)-2-(prop-1-en-2-yl)-3-((4,4,5,5-tetramethyl-1,3,2-dioxaborolan-2-yl)methyl)cyclobut-1-en-1-yl)ethyl)isoindoline-1,3-dione (**3m**).

Following the general procedure B using 20 mol% DABCO as ligand, **3m** was isolated as a colorless oil 37.3 mg, 81% yield, 7:1 dr). **^{1}H NMR** (400 MHz, $CDCl_3$) δ = 7.85 – 7.81 (m, 2H), 7.73 – 7.67 (m, 2H), 5.87 (ddt, J = 16.9, 10.2, 6.6 Hz, 1H), 5.05 (dd, J = 17.1, 1.7 Hz, 1H), 4.94 (ddd, J = 10.1, 2.2, 1.2 Hz, 1H), 4.80 (dd, J = 6.2, 1.8 Hz, 2H), 3.79 – 3.72 (m, 2H), 2.71 (ddd, J = 13.9, 9.0, 7.0 Hz, 1H), 2.49 – 2.40 (m, 2H), 2.31 – 2.09 (m, 3H), 1.91 (s, 3H), 1.80 – 1.65 (m, 1H), 1.59 – 1.48 (m, 1H), 1.29 – 1.17 (m, 13H), 0.81 (dd, J = 15.8, 9.8 Hz, 1H). **^{13}C NMR** (101 MHz, $CDCl_3$) δ = 168.1, 145.7, 139.6, 139.3, 138.5, 133.9, 132.2, 123.2, 114.1, 113.2, 82.9, 48.3, 41.0, 36.5, 32.2, 32.0, 27.5, 25.0, 24.9, 24.9, 21.0. **HRMS** (ESI-TOF) m/z Cal. for $C_{28}H_{38}BNaO_4$ $(M+H)^+$: 462.2815, found: 462.2807.

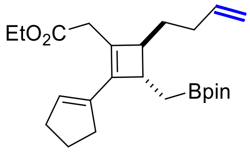

3n

Ethyl 2-(4-(but-3-en-1-yl)-2-(cyclopent-1-en-1-yl)-3-((4,4,5,5-tetramethyl-1,3,2-dioxaborolan-2-yl)methyl)cyclobut-1-en-1-yl)acetate (**3n**). Following the general procedure A using 20 mol% DABCO as ligand, **3n** was isolated as a colorless oil (34.6 mg, 86% yield, 16:1 dr) **^{1}H NMR** (400 MHz, $CDCl_3$) δ = 5.85 (ddt, J = 16.9, 10.0, 6.6 Hz, 1H), 5.76 – 5.68 (m, 1H), 5.05 – 4.97 (m, 1H), 4.96 – 4.89 (m, 1H), 4.14 (q, J = 7.2 Hz, 2H), 3.26 (d, J = 15.3 Hz, 1H), 3.16 – 3.11 (m, 1H), 2.63 – 2.46 (m, 1H), 2.62 – 2.27 (m, 6H), 2.23 – 2.03 (m, 2H), 1.97 – 1.79 (m, 2H), 1.76 – 1.66 (m, 1H), 1.61 – 1.46 (m, 1H), 1.30 – 1.20 (m, 15H), 0.87 (dd, J = 15.8, 10.0 Hz, 1H). minor isomer (distinct peaks): δ 4.08 (t, J = 7.1 Hz, 2H). **^{13}C NMR** (101 MHz, $CDCl_3$) δ = 170.6, 142.6, 139.4, 137.6, 133.4, 128.3, 113.9, 82.9, 60.5, 50.0, 41.7, 34.6, 33.1, 32.2, 32.1, 31.8, 24.9, 24.8, 23.3, 14.2. **HRMS** (ESI-TOF) m/z Cal. for $C_{24}H_{38}BO_4$ $(M+H)^+$: 401.2862, found: 401.2841.

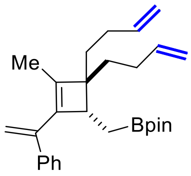

3o

2-((4,4-Di(but-3-en-1-yl)-3-methyl-2-(1-phenylvinyl)cyclobut-2-en-1-yl)methyl)-4,4,5,5-tetramethyl-1,3,2-dioxaborolane (**3o**). Following the general procedure B using 20 mol% as ligand, the reaction was run on a 1.0 mmol scale and **3o** was isolated as a colorless oil (380.6 mg, 91% yield) **^{1}H NMR** (400 MHz, $CDCl_3$) δ = 7.29 – 7.15

(m, 5H), 5.86 – 5.72 (m, 2H), 5.07 (d, J = 1.9 Hz, 1H), 5.03 (d, J = 1.8 Hz, 1H), 5.00 – 4.96 (m, 1H), 4.95 – 4.91 (m, 1H), 4.86 (ddd, J = 10.1, 6.6, 1.9 Hz, 2H), 2.81 (ddq, J = 10.0, 6.1, 2.1 Hz, 1H), 2.15 – 1.94 (m, 4H), 1.67 (ddd, J = 13.5, 12.0, 4.9 Hz, 1H), 1.57 – 1.48 (m, 2H), 1.31 (ddd, J = 13.5, 12.2, 4.7 Hz, 1H), 1.21 (d, J = 2.0 Hz, 3H), 1.17 – 1.14 (m, 12H), 1.03 (dd, J = 16.4, 5.9 Hz, 1H), 0.85 (dd, J = 16.4, 9.4 Hz, 1H). **^{13}C NMR** (101 MHz, $CDCl_3$) δ = 146.6, 143.2, 141.3, 141.2, 139.7, 139.6, 127.9, 127.7, 127.2, 113.8, 113.7, 113.3, 82.9, 49.6, 44.8, 35.0, 32.0, 29.7, 29.4, 24.9 (overlapped, 2C), 12.9. **HRMS** (ESI-TOF) m/z Cal. for $C_{28}H_{40}BO_2$ $(M+H)^+$: 419.3121, found: 419.3106.

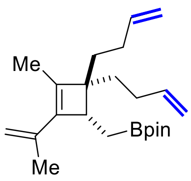

3p

2-((4,4-Di(but-3-en-1-yl)-3-methyl-2-(prop-1-en-2-yl)cyclobut-2-en-1-yl)methyl)-4,4,5,5-tetramethyl-1,3,2-dioxaborolane (**3p**). Following the general procedure B, **3p** was isolated as a colorless oil (54 mg, 75% yield). **^{1}H NMR** (400 MHz, $CDCl_3$) δ 5.93 – 5.75 (m, 2H), 5.09 – 4.74 (m, 6H), 2.80 – 2.65 (m, 1H), 2.21 – 1.96 (m, 4H), 1.91 (s, 3H), 1.76 (d, J = 2.1 Hz, 3H), 1.56 – 1.49 (m, 2H), 1.38 – 1.32 (m, 2H), 1.24 (s, 12H), 1.13 – 1.04 (m, 1H), 0.88 – 0.79 (m, 1H). **^{13}C NMR** (101 MHz, $CDCl_3$) δ 144.5, 142.3, 140.0, 139.8, 139.1, 113.9, 113.7, 111.9, 83.1, 49.3, 44.0, 35.2, 32.1, 29.9, 29.5, 25.1, 21.8, 13.3. **HRMS** (ESI-TOF) m/z Calc. for $C_{23}H_{37}BO_2Na$ $(M+Na)^+$: 379.2783, found: 379.2787.

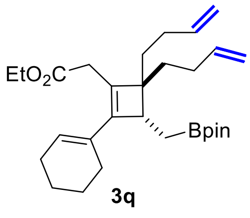

3q

Ethyl 2-(4,4-di(but-3-en-1-yl)-2-(cyclohex-1-en-1-yl)-3-((4,4,5,5-tetramethyl-1,3,2-dioxaborolan-2-yl)methyl)cyclobut-1-en-1-yl)acetate (**3q**). Following the general procedure A with 0.098 mmol of **1q**, **3q** was isolated as a colorless oil (30.6 mg, 67% yield) **^{1}H NMR** (400 MHz, $CDCl_3$) δ = 5.82 (ddq, J = 16.8, 10.2, 6.7 Hz, 2H), 5.69 (td, J = 4.0, 2.0 Hz, 1H), 4.99 (dt, J = 16.8, 1.8 Hz, 2H), 4.90 (dt, J = 10.2, 1.7 Hz, 2H),

4.11 (q, J = 7.1 Hz, 2H), 3.21 – 3.07 (m, 2H), 2.74 (dd, J = 9.6, 5.4 Hz, 1H), 2.35 – 2.22 (m, 1H), 2.20 – 1.93 (m, 7H), 1.77 – 1.45 (m, 7H), 1.45 – 1.17 (m, 16H), 1.06 (dd, J = 16.5, 5.4 Hz, 1H), 0.87 (dd, J = 16.5, 9.5 Hz, 1H). **^{13}C NMR** (101 MHz, $CDCl_3$) δ = 171.1, 146.7, 139.7, 139.5, 135.5, 132.3, 125.7, 113.7, 113.6, 82.9, 60.6, 49.6, 44.1, 35.5, 34.0, 31.7, 29.5, 29.5, 26.6, 25.3, 24.9, 24.9, 22.5, 22.1, 14.2. **HRMS** (ESI-TOF) m/z Cal. for $C_{29}H_{46}BO_4$ $(M+H)^+$: 469.3489, found: 469.3495.

3. Procedures for the Synthesis of Allenes

3.1 General Procedure C for the Synthesis of Allene 1a-1j

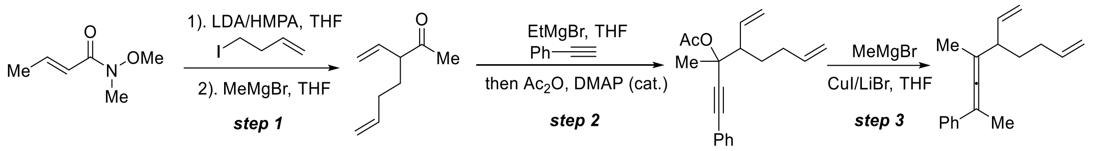

S1 **S2** **S3** **1a**

Step 1: Lithium diisopropylamide (LDA, 13.0 mmol, 1.0 M, 1.3 equiv.) was added to a 100 mL oven-dried Schlenk flask, followed by the slow addition of hexamethylphosphoramide (HMPA, 13.0 mmol, 1.3 equiv.) at -78 °C under N_2 atmosphere. After being stirred for 30 min, *N,O*-dimethyl crotonicamide **S1** (10.0 mmol, 1.0 equiv.) was added slowly to the solution at -78 °C and stirred for another 30 min before the addition of 3-butenyl iodide (1.1 equiv.). The reaction mixture was warmed up to 0 °C after being stirred at -78 °C for 6 h. Sat. aq. NH_4Cl was added to the mixture. The mixture was extracted with ethyl acetate (20 mL x 3), washed with sat. aq. NaCl and dried over Na_2SO_4. The residue was concentrated under vacuum and purified by column chromatography on silica gel to afford the α-alkylated amide in 61% yield (Petroleum ether: EtOAc = 5 : 1). Methylmagnesium bromide (1.5 equiv., 3.0 M in Et_2O) was added slowly to the prepared α-alkylated amide solution (1.0 M in Et_2O) at 0 °C. After the starting material has been consumed, sat. aq. NH_4Cl was added to the mixture. The mixture was extracted with ethyl acetate (20 mL x 3), washed with sat. aq. NaCl and dried over Na_2SO_4. The residue was concentrated in vacuo and purified by column chromatography on silica gel (Petroleum ether: EtOAc = 20 : 1) to give ketone **S2** (95%).

Step 2: To a solution of phenyl acetylene (715 mg, 7.0 mmol, 1.4 equiv.) in 10 mL THF was added 7.0 mL EtMgBr (1.0 M in THF, 7.0 mmol, 1.4 equiv.) slowly at 0 °C under N_2. The mixture was heated at 40 °C and stirred for 1 h to afford the (phenylethynyl)

magnesium bromide solution (caution: gas evolution). The prepared Grignard reagent was added to a solution of ketone **S2** (691 mg, 5.0 mmol, 1.0 equiv.) in 10 mL THF at 0 °C under N_2. After the consumption of ketone, Ac_2O (612 mg, 6.0 mmol, 1.2 equiv.) and DMAP (61 mg, 0.5 mmol, 0.1 equiv.) were added to the reaction mixture at 0 °C. The mixture was stirred at room temperature for 6 h. The reaction was quenched by the addition of 10 mL of saturated aq. NH_4Cl solution and extracted by EtOAc (20 mL x 3). The combined organic layers were dried over Na_2SO_4, concentrated under reduced pressure, and the residue purified by column chromatography on silica gel to afford propargyl ester **S3** (1.22 g, 87%). **^{1}H NMR** (400 MHz, $CDCl_3$) major isomer, δ 7.46 – 7.40 (m, 2H), 7.32 – 7.27 (m, 3H), 5.88 – 5.63 (m, 2H), 5.24 – 4.94 (m, 4H), 2.58 (ddd, *J* = 11.5, 9.4, 2.5 Hz, 1H), 2.24 – 2.12 (m, 1H), 2.07 – 1.84 (m, 5H), 1.76 (s, 3H), 1.61 – 1.49 (m, 1H). minor isomer (distinct peaks): δ 2.68 (ddd, *J* = 11.1, 9.7, 2.5 Hz, 1H), 2.04 (s, 3H), 1.71 (s, 3H). **^{13}C NMR** (101 MHz, $CDCl_3$) major isomer, δ 169.3, 138.5, 136.9, 131.8, 128.2, 122.6, 118.7, 114.8, 88.9, 85.8, 77.8, 53.4, 31.4, 28.3, 24.7, 22.0. minor isomer: δ 169.2, 138.5, 136.8, 131.8, 128.3, 122.6, 118.9, 114.7, 88.3, 86.0, 77.6, 53.0, 31.4, 28.3, 24.7, 22.0. **HRMS** (ESI-TOF) m/z Cal. for $C_{19}H_{22}NaO_2$ $(M+Na)^+$: 305.1512, found: 305.1513.

Step 3: Copper iodide (2.285 g, 12.0 mmol, 3.0 equiv.) and lithium bromide (dried with heat gun under vacuum) (1.041g, 12.0 mmol, 3.0 equiv.) were added to a 100 mL Schlenk flask under N_2, and then 20 mL THF was added to the flask. 4 mL of methylmagnesium bromide solution (3.0 M in THF, 12.0 mmol, 3.0 equiv.) was added slowly to the mixture at 0 °C and a bright yellow slurry was formed immediately. The mixture was stirred at 0 °C for another 1 h before the addition of a solution of **S3** (1.13 g, 4.0 mmol, 1.0 equiv.) in 4 mL THF. The resulting mixture was stirred for another 4 h before quenching with 2 mL saturated aq.NH_4Cl solution (dropwise addition at first due to gas evolution) and then 20 mL NH_4OH solution (25% aq.). The mixture was extracted by Et_2O (20 mL x 3), the combined organic layers were dried over Na_2SO_4, filtered and concentrated in vacuo. The residue was purified by column chromatography on silica gel to afford the allene **1a** as a colorless oil (905 mg, 95%, 58:42 dr). **^{1}H NMR** (400 MHz, $CDCl_3$) δ 7.44 – 7.37 (m, 2H), 7.34 – 7.28 (m, 2H), 7.22 – 7.15 (m, 1H), 5.89 – 5.62 (m, 2H), 5.15 – 4.89 (m, 4H), δ 2.59 (q, *J* = 7.7 Hz, 1H), 2.16 – 2.02 (m, 5H), 1.76 (s, 3H), 1.71 – 1.59 (m, 1H), 1.53 – 1.43 (m, 1H). **^{13}C NMR** (101 MHz, $CDCl_3$) major isomer δ 201.6, 141.3, 138.9, 138.5, 128.4, 126.3,

125.7, 115.2, 114.7, 103.6, 100.9, 48.6, 32.5, 31.8, 17.7, 17.4. Minor isomer δ 201.9, 141.1, 138.9, 138.4, 128.4, 126.3, 125.7, 115.1, 114.7, 103.5, 100.5, 48.4, 32.3, 31.8, 17.5, 17.2. **HRMS** (ESI-TOF) m/z Cal. for $C_{18}H_{22}Na$ $(M+Na)^+$: 261.1614, found: 261.1592.

For compound **1b**-**1j** similar yields were obtained for steps 1-3 as those were obtained for **1a**.

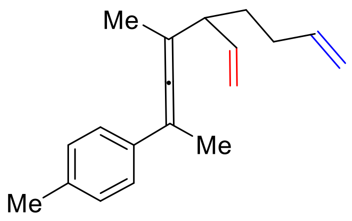

1b

1-Methyl-4-(4-methyl-5-vinylnona-2,3,8-trien-2-yl)benzene (**1b**). Isolated as a colorless oil, diastereomeric ratio (55:45 dr). **^{1}H NMR** (400 MHz, $CDCl_3$) δ 7.29 (d, *J* = 8.2 Hz, 2H), 7.12 (d, *J* = 8.0 Hz, 2H), 5.89 – 5.62 (m, 2H), 5.13 – 4.85 (m, 4H), 2.67 – 2.51 (m, 1H), 2.34 (s, 3H), 2.15 – 2.00 (m, 5H), 1.75 (s, 3H), 1.67 – 1.59 (m, 1H), 1.51 – 1.42 (m, 1H). **^{13}C NMR** (101 MHz, $CDCl_3$) major isomer δ 201.3, 141.4, 139.0, 136.0, 135.5, 129.1, 125.6, 115.1, 114.6, 103.4, 100.7, 48.6, 32.5, 31.8, 21.2, 17.8, 17.5. Minor isomer δ 201.6, 141.2, 138.9, 136.0, 135.5, 129.1, 125.6, 115.0, 114.7, 103.27, 100.3, 48.4, 32.3, 31.7, 21.2, 17.6, 17.2. **HRMS** (ESI-TOF) m/z Cal. for $C_{19}H_{24}Na$ $(M+Na)^+$: 275.1770, found: 275.1768.

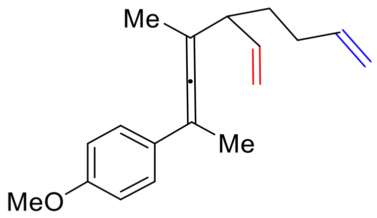

1c

1-Methoxy-4-(4-methyl-5-vinylnona-2,3,8-trien-2-yl)benzene (**1c**). Isolated as a colorless oil, diastereomeric ratio (58:42 dr). **^{1}H NMR** (400 MHz, $CDCl_3$) δ 7.35 – 7.28 (m, 2H), 6.89 – 6.83 (m, 2H), 5.87 – 5.61 (m, 2H), 5.13 – 4.89 (m, 4H), 3.81 (s, 3H), 2.68 – 2.52 (m, 1H), 2.14 – 2.00 (m, 5H), 1.75 (s, 3H), 1.68 – 1.58 (m, 1H), 1.52 – 1.42 (m, 1H). **^{13}C NMR** (101 MHz, $CDCl_3$) major isomer δ 201.0, 158.4, 141.4, 139.0, 130.9, 126.8, 115.1, 114.6, 113.8, 103.4, 100.4, 55.5, 48.6, 32.5, 31.8, 17.9, 17.6. Minor isomer

201.3, 158.4, 141.2, 138.9, 130.8, 126.8, 115.0, 114.7, 113.8, 103.3, 100.0, 55.5, 48.5, 32.4, 31.8, 17.7, 17.3. **HRMS** (ESI-TOF) m/z Cal. for $C_{19}H_{24}ONa$ $(M+Na)^+$: 291.1719, found: 291.1711.

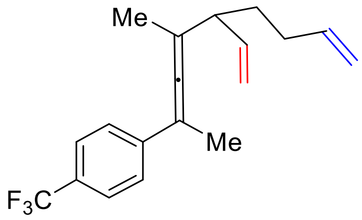

1d

1-(4-Methyl-5-vinylnona-2,3,8-trien-2-yl)-4-(trifluoromethyl)benzene (1d). Isolated as a colorless oil, diastereomeric ratio (55:45 dr). **^{1}H NMR** (400 MHz, $CDCl_3$) δ 7.57 – 7.52 (m, 2H), 7.50 – 7.43 (m, 2H), 5.86 – 5.60 (m, 2H), 5.15 – 4.88 (m, 4H), 2.72 – 2.55 (m, 1H), 2.18 – 1.97 (m, 5H), 1.78 (s, 3H), 1.69 – 1.57 (m, 1H), 1.54 – 1.43 (m, 1H). **^{19}F NMR** (377 MHz, Chloroform-*d*) major isomer δ -62.33. Minor isomer δ -62.32. **^{13}C NMR** (101 MHz, $CDCl_3$) major isomer δ 202.5, 142.4 (d, J = 1.5 Hz), 140.8, 138.7, 128.7 – 127.7 (m), 125.8, 125.8, 125.3, 115.6, 114.8, 104.5, 100.2, 48.4, 32.5, 31.7, 17.6, 17.3. Minor isomer δ 202.8, 142.3 (d, J = 1.5 Hz), 140.6, 138.6, 128.7 – 127.7 (m), 125.9, 125.8, 125.3, 115.5, 114.8, 104.3, 99.8, 48.3, 32.3, 31.7, 17.4, 17.0. **HRMS** (ESI-TOF) m/z Cal. for $C_{19}H_{21}F_3Na$ $(M+Na)^+$: 329.1560, found: 329.1546.

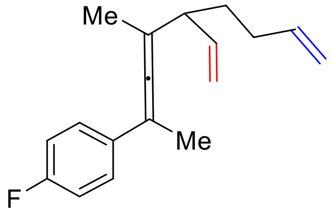

1e

1-fluoro-4-(4-methyl-5-vinylnona-2,3,8-trien-2-yl)benzene (1e). Isolated as a colorless oil, diastereomeric ratio (59:41 dr). **^{1}H NMR** (400 MHz, $CDCl_3$) δ 7.38 – 7.29 (m, 2H), 7.05 – 6.94 (m, 2H), 5.86 – 5.60 (m, 2H), 5.13 – 4.89 (m, 4H), 2.68 – 2.54 (m, 1H), 2.13 – 2.00 (m, 5H), 1.75 (s, 3H), 1.68 – 1.57 (m, 1H), 1.52 – 1.42 (m, 1H). **^{19}F NMR** (377 MHz, $CDCl_3$) major isomer δ -117.00. Minor isomer δ -117.03. **^{13}C NMR** (101 MHz, $CDCl_3$) major isomer δ 201.4 (d, J = 2.2 Hz), 161.7 (d, J = 245.3 Hz), 141.1, 138.9, 134.5 (d, J = 3.3 Hz), 127.1, 127.1, 115.0, 114.7, 103.9, 100.1, 48.5, 32.5, 31.7, 17.7, 17.2. Minor isomer δ 201.6 (d, J = 2.1 Hz), 161.7 (d, J = 245.1 Hz), 141.0, 138.8,

134.4 (d, J = 3.0 Hz), 127.2, 127.1, 115.2, 115.0, 114.7, 103.7, 99.7, 48.4, 32.3, 31.7, 17.6, 17.2. **HRMS** (ESI-TOF) m/z Cal. for $C_{18}H_{21}FNa$ $(M+Na)^+$: 279.1519, found: 279.1562.

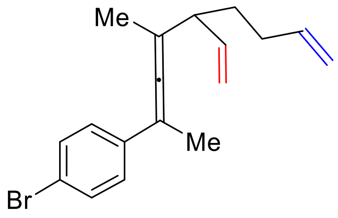

1f

1-Bromo-4-(4-methyl-5-vinylnona-2,3,8-trien-2-yl)benzene (**1f**). Isolated as a colorless oil, diastereomeric ratio (55:45 dr). **^{1}H NMR** (400 MHz, $CDCl_3$) δ 7.45 – 7.38 (m, 2H), 7.26 – 7.21 (m, 2H), 5.86 – 5.58 (m, 2H), 5.14 – 4.89 (m, 5H), 2.69 – 2.54 (m, 1H), 2.13 – 1.99 (m, 4H), 1.76 (s, 3H), 1.62 (ddq, J = 13.4, 9.0, 6.7 Hz, 1H), 1.52 – 1.42 (m, 1H). **^{13}C NMR** (101 MHz, $CDCl_3$) major isomer 201.7, 141.0, 138.8, 137.6, 131.4, 127.3, 120.1, 115.4, 114.8, 104.2, 100.2, 48.5, 32.5, 31.7, 17.6, 17.3. Minor isomer δ 201.9, 140.8, 138.7, 137.5, 131.4, 127.3, 120.1, 115.3, 114.8, 104.0, 99.8, 48.3, 32.3, 31.7, 17.4, 17.0. **HRMS** (ESI-TOF) m/z Cal. for $C_{18}H_{21}BrNa$ $(M+Na)^+$: 339.0719, found: 339.0715.

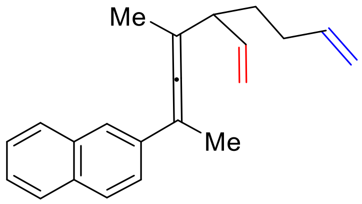

1g

2-(4-Methyl-5-vinylnona-2,3,8-trien-2-yl)naphthalene (**1g**). Isolated as a colorless oil, diastereomeric ratio (58:42 dr). **^{1}H NMR** (400 MHz, $CDCl_3$) δ 7.86 – 7.71 (m, 4H), 7.65 – 7.58 (m, 1H), 7.50 – 7.39 (m, 2H), 5.89 – 5.68 (m, 2H), 5.17 – 4.90 (m, 4H), 2.75 – 2.60 (m, 1H), 2.23 (s, 3H), 2.18 – 2.05 (m, 2H), 1.83 (s, 3H), 1.75 – 1.64 (m, 1H), 1.59 – 1.48 (m, 1H). **^{13}C NMR** (101 MHz, $CDCl_3$) major isomer δ 202.5, 141.2, 138.9, 135.9, 133.8, 132.4, 127.7, 126.1, 115.3, 114.7, 103.9, 101.24, 48.6, 32.5, 31.8, 17.8, 17.5. Minor isomer δ 202.7, 141.0, 138.8, 135.9, 133.8, 132.3, 128.1, 127.7, 125.5, 125.3, 115.2, 114.7, 103.7, 100.8, 48.4, 32.3, 31.7, 17.6, 17.2. **HRMS** (ESI-TOF) m/z Cal. for $C_{22}H_{24}Na$ $(M+Na)^+$: 311.1770, found: 311.1750.

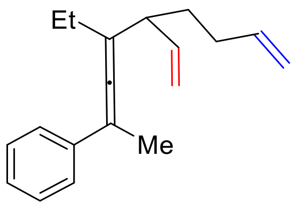

1h

(4-Ethyl-5-vinylnona-2,3,8-trien-2-yl)benzene (**1h**). Isolated as a colorless oil, diastereomeric ratio (54:46 dr). **^{1}H NMR** (400 MHz, $CDCl_3$) δ 7.48 – 7.39 (m, 2H), 7.36 – 7.28 (m, 2H), 7.23 – 7.15 (m, 1H), 5.90 – 5.63 (m, 2H), 5.17 – 4.87 (m, 4H), 2.74 – 2.54 (m, 1H), 2.26 – 1.96 (m, 7H), 1.72 – 1.60 (m, 1H), 1.56 – 1.44 (m, 1H), 1.08 – 0.98 (m, 3H). **^{13}C NMR** (101 MHz, $CDCl_3$) major isomer δ 201.0, 141.8, 139.0, 138.6, 128.3, 126.3, 125.5, 114.9, 114.6, 110.9, 103.3, 47.9, 32.9, 31.8, 24.9, 17.5, 12.4. Minor isomer δ 201.2, 141.5, 138.9, 138.5, 128.3, 126.3, 125.5, 114.8, 114.7, 110.9, 103.1, 47.8, 32.8, 31.8, 24.6, 17.6, 12.5. **HRMS** (ESI-TOF) m/z Cal. for $C_{19}H_{24}Na$ $(M+Na)^+$: 275.1770, found: 275.1740.

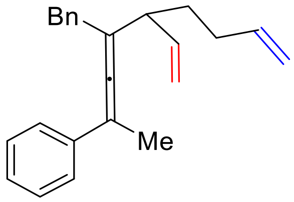

1i

(2-(Hepta-1,6-dien-3-yl)penta-2,3-diene-1,4-diyl)dibenzene (**1i**). Isolated as a colorless oil, diastereomeric ratio (64:36 dr). **^{1}H NMR** (400 MHz, $CDCl_3$) δ 7.41 – 7.15 (m, 10H), 5.80 – 5.62 (m, 2H), 5.14 – 5.03 (m, 2H), 4.95 – 4.82 (m, 2H), 3.55 – 3.30 (m, 2H), 2.71 – 2.53 (m, 1H), 2.12 – 1.92 (m, 5H), 1.70 – 1.58 (m, 1H), 1.55 – 1.42 (m, 1H). **^{13}C NMR** (101 MHz, $CDCl_3$) major isomer δ 202.8, 141.2, 139.9, 138.8, 138.0, 129.3, 128.3, 128.3, 126.4, 126.2, 125.6, 115.4, 114.6, 108.6, 102.7, 46.65, 38.7, 32.9, 31.7, 17.5. Minor isomer δ 202.6, 141.5, 139.9, 138.8, 138.1, 129.3, 128.3, 128.3, 126.5, 126.2, 125.6, 115.5, 114.6, 108.4, 102.7, 46.6, 38.7, 32.8, 31.7, 17.4. **HRMS** (ESI-TOF) m/z Cal. for $C_{24}H_{26}Na$ $(M+Na)^+$: 337.1927, found: 337.1927.

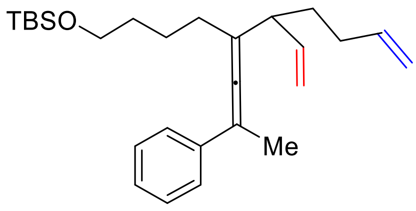

1j

tert-Butyldimethyl((5-(2-phenylprop-1-en-1-ylidene)-6-vinyldec-9-en-1-yl)oxy)silane (1j). Isolated as a colorless oil, diastereomeric ratio (61:39 dr). **^{1}H NMR** (400 MHz, $CDCl_3$) δ 7.43 – 7.36 (m, 2H), 7.35 – 7.27 (m, 2H), 7.22 – 7.14 (m, 1H), 5.86 – 5.60 (m, 2H), 5.12 – 4.88 (m, 4H), 3.57 (t, J = 6.3 Hz, 2H), 2.66 – 2.53 (m, 1H), 2.17 – 1.96 (m, 7H), 1.69 – 1.59 (m, 1H), 1.54 – 1.42 (m, 4H), 0.86 (s, 9H). **^{13}C NMR** (101 MHz, $CDCl_3$) major isomer δ 201.3, 141.7, 139.0, 138.4, 128.3, 126.3, 125.5, 115.0, 114.6, 109.1, 102.9, 63.25, 47.8, 32.8, 31.8, 31.6, 26.1, 24.2, 18.5, 17.5, -5.2. Minor isomer δ 201.4, 141.5, 138.9, 138.3, 128.3, 126.3, 125.6, 114.9, 114.7, 109.1, 102.7, 63.3, 47.8, 32.8, 31.8, 31.3, 26.1, 24.3, 18.5, 17.6, -5.2. **HRMS** (ESI-TOF) m/z Cal. for $C_{27}H_{42}OSiNa$ $(M+Na)^+$: 433.2897, found: 433.2898.

3.2 Procedure for the Synthesis of Allene 1k and 1m

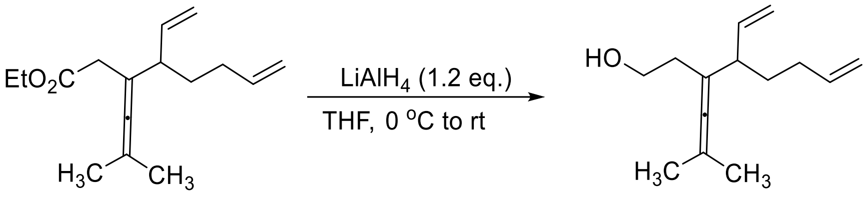

1l **1k**

To a 100 mL Schenck flask were added $LiAlH_4$ (1.8 mmol, 1.5 equiv.) and THF (10 mL) under N_2. The solution of **1l** (300 mg, 1.2 mmol) in 2 mL THF was added slowly to the mixture at 0 °C. The reaction was warmed to room temperature and stirred for 12 h. $NaSO_4 \cdot 10H_2O$ (500 mg) was added to the mixture to quench the reaction. The mixture was filtrated through a short pad of silica gel. The solvent was removed under vacuum and **1k** was obtained as a colorless oil (230 mg, 92%). **^{1}H NMR** (400 MHz, $CDCl_3$) δ = 5.84 – 5.74 (m, 1H), 5.59 (ddd, J = 17.0, 10.2, 8.5 Hz, 1H), 5.06 – 4.90 (m, 4H), 3.73 – 3.63 (m, 2H), 2.45 (q, J = 7.5 Hz, 1H), 2.26 – 1.95 (m, 4H), 1.72 (s, 3H), 1.70 (s, 3H), 1.64 (br, 1H), 1.59 – 1.52 (m, 1H), 1.49 – 1.37 (m, 1H). **^{13}C NMR** (100 MHz, $CDCl_3$) δ = 198.3, 141.4, 138.9, 114.6, 114.5, 101.2, 98.2, 61.3, 47.3, 34.6, 32.5, 31.5, 20.9, 20.8. **HRMS** (ESI-TOF) m/z Cal. for $C_{14}H_{23}O$ $(M+H)^+$: 207.1743, found: 207.1734.

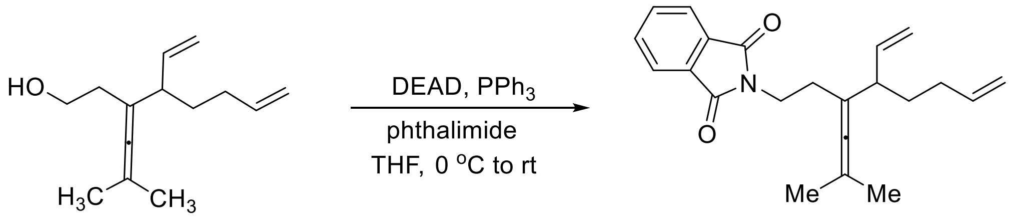

1k **1m**

To a 100 mL Schenck flask were added triphenylphosphine (314.8 mg, 1.2 mmol), phthalimide (176.4 mg, 1.2 mmol) and THF (10 mL) in sequence under N_2. Diethyl azodicarboxylate (DEAD, 209 mg, 1.2 mmol) was added dropwise to the mixture at 0 °C. The reaction was stirred at room temperature for 2 h followed by the addition of 10 mL sat. NH_4Cl (aq.). The mixture was extracted with ethyl acetate (10 mL x 3). The organic layers were combined, washed with brine, dried over Na_2SO_4, and concentrated in vacuo. The residue was purified by column chromatography on silica gel (Petroleum ether: EtOAc = 10:1) to afford **1m** as a colorless oil (200 mg, 85% yield). **^{1}H NMR** (400 MHz, $CDCl_3$) δ = 7.83 (dd, J = 5.5, 3.1 Hz, 2H), 7.70 (dd, J = 5.5, 3.1 Hz, 2H), 5.84 – 5.74 (m, 1H), 5.64 – 5.49 (m, 1H), 5.06 – 4.89 (m, 4H), 3.79 – 3.67 (m, 2H), 2.49 (q, J = 7.6 Hz, 1H), 2.38 – 2.17 (m, 2H), 2.13 – 1.95 (m, 2H), 1.63 (s, 3H), 1.58 (s, 3H), 1.56 – 1.45 (m, 1H), 1.45 – 1.36 (m, 1H). **^{13}C NMR** (100 MHz, $CDCl_3$) δ = 198.8, 168.3, 141.3, 138.9, 133.8, 132.3, 123.1, 114.7, 114.3, 100.7, 98.2, 47.1, 36.8, 32.4, 31.5, 29.8, 20.6, 20.7. **HRMS** (ESI-TOF) m/z Cal. for $C_{22}H_{25}NNaO_2$ $(M+Na)^+$: 358.1778, found: 358.1777.

3.3 Procedure for the Synthesis of Allene 1l and 1n.

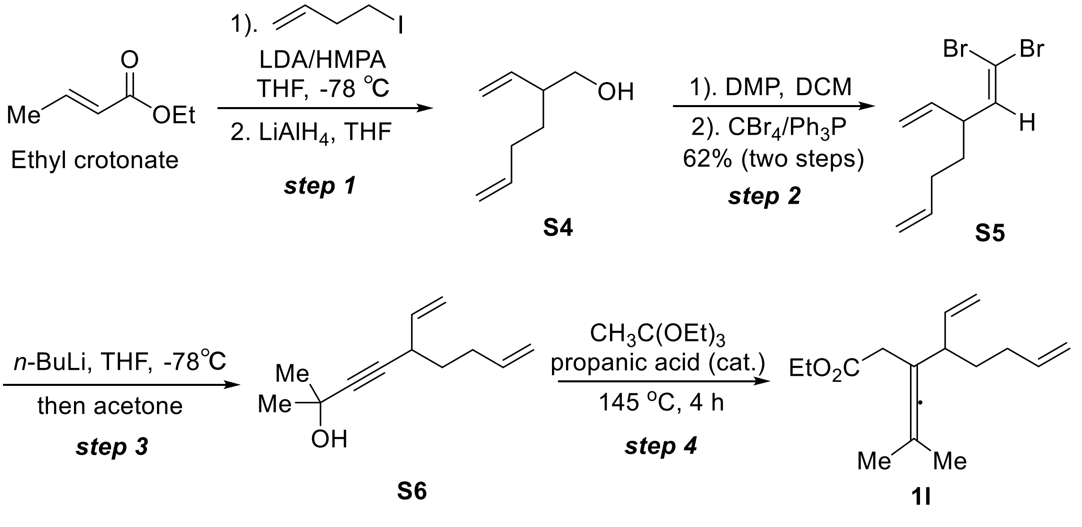

Step 1: According to a previously reported literature,[7] 22.0 mL of lithium diisopropylamide (LDA) (1.0 M in THF, 22.0 mmol, 1.1 equiv.) was added to a 100 Schlenk flask, followed by the slow addition of HMPA (3.8 mL 22.0 mmol, 1.1 equiv.) at -78 °C under N_2 atmosphere. After being stirred for 30 min, ethyl crotonate (2.28 g, 20.0 mmol, 1.0 equiv.) was added slowly to the solution and stirred at -78 °C for another 30 min before the addition of 3-butenyl iodide (4.0 g, 22 mmol, 1.1 equiv.). After the mixture was stirred at -78 °C for 6 h, the mixture was warmed up to 0 °C. Saturated aq. NH_4Cl was added and the mixture was extracted with EtOAc (20 mL x 3), washed with brine and dried over Na_2SO_4. The residue was concentrated in vacuo and purified by column chromatography on silica gel to afford the α-alkylated ester (Petroleum ether: EtOAc = 20:1 to 10:1) as a colorless oil (2.119 g, 63%). This prepared α-alkylated ester was added slowly to a suspension of with $LiAlH_4$ (622 mg, 16.4 mmol, 1.3 equiv.) in 50 mL THF at 0 °C under N_2. The mixture was allowed to warm to room temperature and stirred overnight. The reaction was quenched with $Na_2SO_4·10\ H_2O$ until no gas evolved. The mixture was filtrated through a short pad of silica to afford **S4** as a colorless oil (1.462g, 92%). **^{1}H NMR** (400 MHz, $CDCl_3$) δ 5.79 (ddt, *J* = 16.9, 10.2, 6.6 Hz, 1H), 5.59 (ddd, *J* = 17.0, 10.4, 8.7 Hz, 1H), 5.20 – 4.92 (m, 4H), 3.58 (dd, *J* = 10.7, 5.1 Hz, 1H), 3.43 (dd, *J* = 10.6, 8.1 Hz, 1H), 2.32 – 1.96 (m, 3H), 1.54 – 1.45 (m). **^{13}C NMR** (101 MHz, $CDCl_3$) δ 139.6, 138.4, 117.7, 114.7, 65.5, 46.4, 31.1, 29.8.

Step 2: Alcohol **S4** (505 mg, 4.0 mmol, 1.0 equiv.) was dissolved in 20 mL dichloromethane (DCM) under N_2 followed by the addition of Dess-Martin periodinane (DMP) (1.87 g, 4.4 mmol, 1.1 equiv.) at 0 °C. The mixture was stirred at room temperature for 2 h before quenching with 10 mL saturated aq. $Na_2S_2O_3$ solution. The mixture was extracted with Et_2O (10 mL x 3) and the combined organic layers were dried over Na_2SO_4. The extracts was then filtrated through a short pad of silica gel and concentrated under vacuum to give the corresponding aldehyde. In a 100 mL round-bottomed flask, a solution of CBr_4 (1.98 g, 6.0 mmol, 1.5 equiv.) and PPh_3 (3.15 g, 12.0 mmol, 3.0 equiv.) in DCM (25 mL) was stirred for 10 min under N_2 at 0 °C. The prepared aldehyde was added to the reaction and the resulting mixture was stirred at 0 °C for 2 h. The reaction mixture was concentrated under vacuum and the residue was purified by column chromatography on silica gel with pentane as eluent to give **S5** as a colorless oil (705 mg, 64%).

Step 3: *n*-Butyl lithium (3.0 mL, 2.5 M in hexane, 2.0 equiv.) was added dropwise to a

solution of **S5** (1.08 g, 3.7 mmol, 1.0 equiv.) in 30 mL THF at -78 °C under N_2. After the mixture had been stirred at -78 °C for 30 min, acetone (280 µL, 3.8 mmol, 1.02 equiv.) was added to the mixture and stirred for 3 h at -78 °C. The reaction was quenched by the addition of saturated aq. NH_4Cl and the mixture was extracted with EtOAc (20 mL x 3), washed with brine and dried over Na_2SO_4. After filtration the filtrate was concentrated under vacuum and purified by column chromatography on silica gel to afford **S6** (483 mg, 71%) as a colorless oil. **^{1}H NMR** (400 MHz, $CDCl_3$) δ 5.89 – 5.68 (m, 2H), 5.27 (dt, *J* = 16.9, 1.6 Hz, 1H), 5.12 – 4.94 (m, 3H), 3.14 – 3.04 (m, 1H), 2.26 – 2.10 (m, 2H), 1.88 (br, 1H), 1.66 – 1.55 (m, 2H), 1.53 (s, 6H). **^{13}C NMR** (101 MHz, $CDCl_3$) δ 138.0, 137.8, 115.1, 115.1, 88.6, 82.5, 65.3, 34.7, 34.4, 31.8, 31.1.

Step 4: A 50 mL round-bottomed flask was equipped with a Dean-Stark apparatus and a condenser. Propargylic alcohol **S6** (390 mg, 2.2 mmol, 1.0 equiv.), triethyl orthoacetate (10 mL), and propanoic acid (16.3 mg, 0.44 mmol, 0.2 equiv.) were added sequentially. After the reaction had been refluxed at 145 °C for 4 h, the mixture was cooled down to 0 °C in an ice bath. Et_2O (10 mL) and aq. HCl (1.0 M, 10 mL) were added. The organic layer was separated, and the aqueous layer was extracted with Et_2O (20 mL x 3). The combined organic layers were dried over Na_2SO_4, filtered, concentrated in vacuo, and purified by column chromatography on silica gel (Petroleum ether /Et_2O = 30/1) to afford the desired product **1l** (430 mg, 79%) as a colorless oil. **^{1}H NMR** (400 MHz, $CDCl_3$) *δ* = 5.86 – 5.74 (m, 1H), 5.56 (ddd, *J* = 17.0, 10.1, 8.5 Hz, 1H), 5.10 – 4.88 (m, 4H), 4.11 (q, *J* = 7.1 Hz, 2H), 2.95 – 2.84 (m, 2H), 2.54 (q, *J* = 7.5 Hz, 1H), 2.11 – 2.02 (m, 2H), 1.71 (s, 3H) 1.69 (s, 3H), 1.61 – 1.51 (m, 1H), 1.47 – 1.36 (m, 1H), 1.25 (t, *J* = 7.1 Hz, 3H). **^{13}C NMR** (101 MHz, $CDCl_3$) δ 199.9, 171.8, 140.9, 138.9, 115.0, 114.4, 98.1, 97.9, 60.4, 46.8, 38.1, 32.5, 31.4, 20.6, 20.4, 14.2. **HRMS** (ESI-TOF) m/z Cal. for $C_{16}H_{24}NaO_2$ $(M+Na)^+$: 271.1669, found: 271.1659.

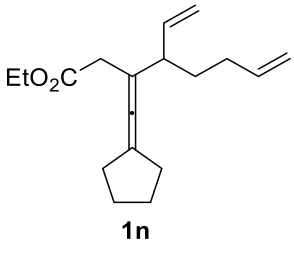

1n

Following the general procedure, **1n** was isolated as a colorless oil. **^{1}H NMR** (400 MHz, $CDCl_3$) *δ* = 5.80 (ddt, *J* = 16.9, 10.2, 6.7 Hz, 1H), 5.56 (ddd, *J* = 17.0, 10.0, 8.6 Hz, 1H), 5.08 – 4.89 (m, 4H), 4.10 (q, *J* = 7.1 Hz, 2H), 2.99 – 2.85 (m, 2H), 2.56 (q, *J* = 7.5

Hz, 1H), 2.40 – 2.24 (m, 4H), 2.11 – 2.01 (m, 2H), 1.72 – 1.51 (m, 5H), 1.47 – 1.38 (m, 1H), 1.24 (t, J = 7.1 Hz, 3H). **^{13}C NMR** (101 MHz, $CDCl_3$) δ = 195.4, 171.7, 141.0, 138.9, 115.0, 114.3, 106.8, 100.4, 60.4, 47.0, 38.2, 32.5, 31.5, 31.1, 30.9, 27.0, 27.0, 14.2. **HRMS** (ESI-TOF) m/z Cal. for $C_{18}H_{26}NaO_2$ $(M+Na)^+$: 297.1825, found: 297.1808.

3.4 Procedure for the Synthesis of Allene 1o.

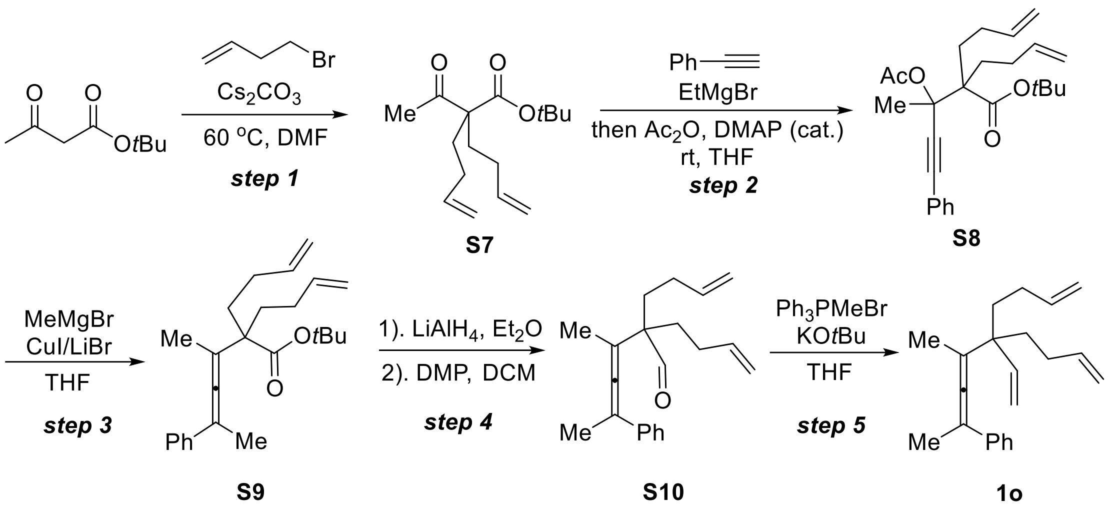

Step 1: To a 250 mL round-bottomed bottle were added *tert*-butyl acetoacetate (3.2 g, 20 mmol, 1.0 equiv.), Cs_2CO_3 (20 g, 60 mmol, 3.0 equiv.), 20 mL DMF and 4-bromo-1-butene (8.1 g, 60 mmol, 3.0 equiv.) sequentially at 0 °C. The resulting mixture was stirred at 60 °C for 12 h. After being cooled to room temperature, 100 mL of H_2O was added and the mixture was extracted with EtOAc (40 mL x 3). The organic layers were combined and washed with brine (30 mL x 3). The organic phase was dried over Na_2SO_4, filtered and concentrated under vacuum. The residue was purified by silica gel column chromatography (Petroleum ether: EtOAc = 30:1) to afford dialkylated ester **S7** as a colorless oil (3.8 g, 71%).

Step 2: To a solution of phenyl acetylene (1.43 g, 14.0 mmol, 1.4 equiv.) in 20 mL THF was added 14.0 mL EtMgBr (1.0 M in THF, 14.0 mmol, 1.4 equiv.) slowly at 0 °C under N_2. The mixture was heated at 40 °C and stirred for 1 h to afford the (phenylethynyl) magnesium bromide solution (caution: gas evolution). The prepared Grignard reagent was added to a solution of ketone **S7** (2.66 g, 10.0 mmol, 1.0 equiv.) in 20 mL THF at 0 °C under N_2. After the consumption of ketone, Ac_2O (1.22 g, 12.0 mmol, 1.2 equiv.) and DMAP (122 mg, 1.0 mmol, 0.1 equiv.) were added to the reaction mixture at 0 °C. The mixture was stirred at room temperature for 6 h. The reaction was quenched by the addition of 20 mL of saturated aq. NH_4Cl solution and extracted by EtOAc (30 mL x

3). The combined organic layers were dried over Na_2SO_4, concentrated under reduced pressure, and the residue purified by column chromatography on silica gel to afford propargyl ester **S8** (3.49 g, 85%).

^{1}H NMR (400 MHz, $CDCl_3$) δ 7.42 (dd, *J* = 6.5, 3.0 Hz, 2H), 7.32 – 7.27 (m, 3H), 5.84 (dtt, *J* = 16.7, 10.0, 6.6 Hz, 2H), 5.05 (dt, *J* = 17.1, 2.0 Hz, 2H), 4.96 (dd, *J* = 10.1, 1.9 Hz, 2H), 2.39 – 2.13 (m, 3H), 2.10 – 1.88 (m, 11H), 1.46 (s, 9H). **^{13}C NMR** (101 MHz, $CDCl_3$) δ 171.7, 168.3, 139.2, 138.8, 131.7, 128.4, 128.2, 122.6, 114.6, 114.4, 87.9, 87.5, 81.0, 79.4, 57.0, 32.8, 32.6, 30.0, 29.5, 28.0, 22.8, 22.2. **HRMS** (ESI-TOF) m/z Cal. for $C_{26}H_{34}NaO_4$ $(M+Na)^+$: 433.2349, found: 433.2343.

Step 3: Copper iodide (4.570 g, 24.0 mmol, 3.0 equiv.) and lithium bromide (dried with heat gun under vacuum) (2.082 g, 24.0 mmol, 3.0 equiv.) were added to a 100 mL Schlenk flask under N_2, and then 40 mL THF was added to the flask. 8 mL of methylmagnesium bromide solution (3.0 M in THF, 24.0 mmol, 3.0 equiv.) was added slowly to the mixture at 0 °C and a bright yellow slurry was formed immediately. The mixture was stirred at 0 °C for another 1 h before the addition of a solution of **S8** (3.29 g, 8.0 mmol, 1.0 equiv.) in 8 mL THF. The resulting mixture was stirred for another 4 h before quenching with 4 mL saturated aq.NH_4Cl solution (dropwise addition at first due to gas evolution) and then 30 mL NH_4OH solution (25% aq.). The mixture was extracted by Et_2O (30 mL x 3), the combined organic layers were dried over Na_2SO_4, filtered and concentrated in vacuo. The residue was concentrated under vacuum and purified by column chromatography on silica gel to afford the allene **S9** as a colorless oil (2.64 g, 90%).

Step 4: A solution of **S9** (2.42 g, 6.6 mmol, 1.0 equiv.) in 5 mL THF was added slowly to a suspension of $LiAlH_4$ (377 mg, 9.9 mmol, 1.5 equiv.) in 20 mL THF at 0 °C. The mixture was stirred at 50 °C for 3 h. After cooling to room temperature, $NaSO_4 \cdot 10H_2O$ (500 mg) was added to the reaction mixture to quench the reaction. The mixture was filtered through a short pad of silica gel, and the filter residue was washed with 100 mL Et_2O. The solvent was removed under vacuum and the corresponding alcohol was obtained as a colorless oil. The obtained alcohol was dissolved in 20 mL of DCM followed by the addition of Dess–Martin periodinane (DMP) (3.82 g, 9 mmol, 1.5 equiv.) at 0 ºC. After being stirred at room temperature for 4 h, the mixture was filtrated and concentrated under vacuum. The residue was purified by column chromatography with silica gel to afford the aldehyde **S10** as a colorless oil (1.70 g, 87%).

Step 5: Methyltriphenylphosphonium bromide (3.2 g, 9.0 mmol, 2.25 equiv.) was added

to a round-bottomed flask charged with 10 mL THF at 0 °C. KO*t*Bu (842 mg, 7.6 mmol, 1.9 equiv.) was added and the mixture was stirred at 0 °C for 30 min. A solution of **S10** (1.18 g, 4.0 mmol, 1.0 equiv.) in 10 mL THF was added to the reaction and then the resulting mixture was stirred at room temperature for 4 h. The mixture was filtrated and washed with EtOAc. The residue was concentrated and purified by column chromatography on silica gel to afford the aldehyde **1o** as a colorless oil. (1.10 g, 94%). ^{1}**H NMR** (400 MHz, $CDCl_3$) δ = 7.43 – 7.38 (m, 2H), 7.37 – 7.28 (m, 2H), 7.22 – 7.16 (m, *J* = 7.1, 1.3 Hz, 1H), 5.89 – 5.70 (m, 3H), 5.16 (dd, *J* = 10.9, 1.2 Hz, 1H), 5.09 – 4.87 (m, 5H), 2.10 (s, 3H), 2.04 – 1.94 (m, 4H), 1.71 – 1.46 (m, 7H). ^{13}**C NMR** (101 MHz, $CDCl_3$) δ 203.2, 144.5, 139.2, 139.2, 138.3, 128.2, 126.2, 125.5, 114.1, 114.1, 113.5, 105.4, 100.4, 47.2, 33.9, 33.6, 28.6, 28.5, 17.5, 14.9. HRMS (ESI-TOF) m/z Cal. for C22H28(M)+: 292.2186, found: 292.2182.

3.5 Procedure for the Synthesis of Allene 1p and 1q

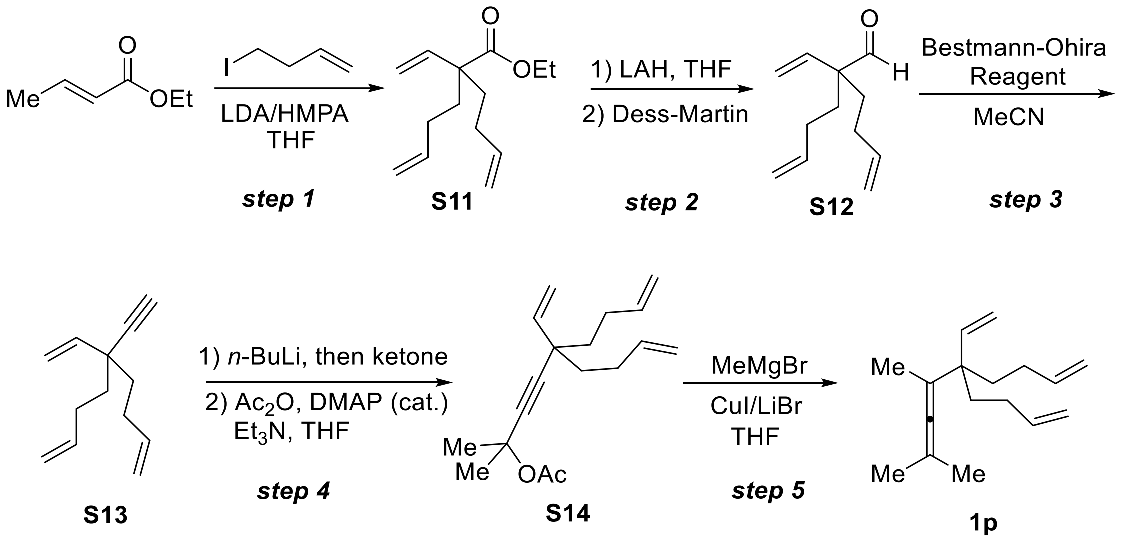

Step 1: To a solution of 24.0 mL LDA (1.0 M in THF, 24.0 mmol, 1.2 equiv.) and HMPA (3.8 mL, 22.0 mmol, 1.1 equiv.) in dry THF (30 mL) was added ethyl crotonate (2.5 mL, 20.0 mmol, 1.0 equiv.) slowly at -78 °C, and the resulting solution was stirred for 30 min. 4-Iodobutene (4.73 g, 26.0 mmol, 1.3 equiv.) was added to the mixture, and the stirring was continued for another 2 h at room temperature. To the reaction mixture was then added LDA (24.0 mL, 1.0 M, 1.2 equiv.) and HMPA (3.8 mL, 22.0 mmol, 1.1 equiv.) at -78 °C, and the resulting solution was stirred for 30 min. To the mixture was added 4-iodobutene (4.37 g, 24.0 mmol, 1.2 equiv.), and the stirring was continued for another 20 h at room temperature. The reaction was quenched by addition of saturated aq. NH_4Cl at 0 °C, and the mixture was diluted with EtOAc. The organic layer was

washed with 1 N HCl, saturated aq. $NaHCO_3$ and brine and then dried over Na_2SO_4. After filtration of the solvent was removed under reduced pressure. The residue was purified by silica gel column chromatography (Pentane: EtOAc = 15:1) to afford dialkylated ester **S11** as a colorless oil (3.20 g, 72%). **^{1}H NMR** (400 MHz, $CDCl_3$) δ 6.01 (dd, *J* = 17.8, 11.0 Hz, 1H), 5.78 (ddt, *J* = 16.8, 10.2, 6.5 Hz, 2H), 5.20 (dd, *J* = 11.0, 0.9 Hz, 1H), 5.10 (dd, *J* = 17.8, 0.9 Hz, 1H), 5.04 – 4.90 (m, 4H), 4.15 (q, *J* = 7.1 Hz, 2H), 2.04 – 1.72 (m, 8H), 1.25 (t, *J* = 7.1 Hz, 3H). **^{13}C NMR** (101 MHz, $CDCl_3$) δ 175.0, 139.6, 138.3, 114.7, 114.5, 60.6, 51.8, 35.4, 28.7, 14.2. **HRMS** (ESI-TOF) m/z Cal. for $C_{14}H_{22}NaO_2$ $(M+Na)^+$: 225.1512, found: 245.1502.

Step 2: To a suspension of $LiAlH_4$ (546 mg, 14.4 mmol) in Et_2O (80 mL) was added dropwise a solution of dialkylated ester **S11** (3.20 g, 14.4 mmol) in Et_2O (30 mL) at 0 °C. After stirring at room temperature for 1.5 h, the reaction was quenched by 20 mL of saturated aq. Rochelle salt, the mixture was stirred for 1.5 h and diluted with EtOAc. The combined organic layers were dried over Na_2SO_4, and concentrated under reduced pressure. The residue was purified by silica gel column chromatography (Pentane: EtOAc = 5:1) to afford alcohol as a colorless oil. To a solution of the obtained alcohol in 50 mL CH_2Cl_2 was added Dess–Martin periodinane (DMP) (7.4 g, 17.3 mmol, 1.2 equiv.) at room temperature, and the resulting mixture was stirred for 4 h. The reaction was quenched with sat. $NaHCO_3$ solution, and extracted with EtOAc. The combined organic layers ere dried over Na_2SO_4, and concentrated under reduced pressure. The residue was purified by silica gel column chromatography (Pentane: EtOAc = 5:1) to afford the aldehyde **S12** as a colorless oil (2.4 g, 91%).

Step 3: Dimethyl-2-oxopropylphosphonate (4.8 g, 28.8 mmol, 2.4 equiv.) was added to a suspension of K_2CO_3 (15.0 g, 108.0 mmol, 9.0 equiv.) and 1H-imidazole-1-sulfonyl azide sulfate (8.5 g, 31.2 mmol, 2.6 equiv.) in MeCN (100 mL), and the resulting mixture was stirred at room temperature for 2 h. Then a solution of aldehyde (2.0 g, 12.0 mmol, 1.0 equiv.) in 30 mL MeOH was added to the reaction and the resulting mixture was stirred for 15 h. The reaction mixture was filtered, the filter cake was washed with Et_2O (50 mL), and the clear solution was carefully evaporated. The residue was purified by silica gel column chromatography (Pentane) to afford the alkyne **S13** as a colorless oil (1.6 g, 75%). **^{1}H NMR** (400 MHz, $CDCl_3$) δ 5.82 (ddt, *J* = 16.9, 10.2, 6.5 Hz, 2H), 5.56 – 5.38 (m, 2H), 5.16 (dd, *J* = 9.8, 2.0 Hz, 1H), 5.01 (dq, *J* = 17.1, 1.8 Hz, 2H), 4.94 (dq, *J* = 10.3, 1.5 Hz, 2H), 2.33 (s, 1H), 2.27 – 2.02 (m, 4H), 1.68 (td, *J*

= 12.8, 4.8 Hz, 2H), 1.49 (td, J = 12.8, 4.8 Hz, 2H). **^{13}C NMR** (101 MHz, $CDCl_3$) δ 140.9, 138.6, 115.3, 114.3, 85.8, 73.4, 43.5, 40.0, 29.3.

Step 4: To a solution of alkyne (730 mg, 4.2 mmol, 1.05 equiv.) in 10 mL of dry THF was added *n*-BuLi (2.5 M, 1.6 mL, 1.0 equiv.) at -78 ºC, and the resulting mixture was stirred at -78 ºC for 1h before the addition of acetone (355 µL, 4.8 mmol, 1.2 equiv.). After the consumption of ketone, Ac_2O (1.2 equiv.) and DMAP (10 mol%) were added at 0 °C. The resulting mixture was stirred at room temperature for 6 h. The reaction was quenched by the addition of 10 mL aq. sat. NH_4Cl solution and extracted by EtOAc, and the organic layer was dried over Na_2SO_4. The organic phase was filtered, concentrated in vacuo and the residue was purified by column chromatography on silica gel to afford propargyl ester **S14** as a colorless oil (990 mg, 86%).

Step 5: Copper iodide (1.71 g, 9.0 mmol, 3.0 equiv.) and lithium bromide (dried with heat gun under vacuum) (780 mg, 9.0 mmol, 3.0 equiv.) were added to a 50 mL Schlenk flask under N_2, and then 20 mL THF was added to the flask. 3 mL of methylmagnesium bromide solution (3.0 M in THF, 9.0 mmol, 3.0 equiv.) was added slowly to the mixture at 0 °C and a bright yellow slurry was formed immediately. The mixture was stirred at 0 °C for another 1 h before the addition of a solution of **S14** (823 mg, 3.0 mmol, 1.0 equiv.) in 4mL THF. The resulting mixture was stirred for another 4 h before quenching with 4 mL saturated aq. NH_4Cl solution (dropwise addition at first due to gas evolution) and then 20 mL NH_4OH solution (25% aq.). The mixture was extracted by Et_2O (20 mL x 3), the combined organic layers were dried over Na_2SO_4, filtered and concentrated in vacuo. The residue was concentrated under vacuum and purified by column chromatography on silica gel to afford the allene **1p** as a colorless oil (510 mg, 74%). **^{1}H NMR** (400 MHz, $CDCl_3$) δ 5.83 (ddt, J = 16.9, 10.3, 6.6 Hz, 2H), 5.68 (dd, J = 17.7, 10.9 Hz, 1H), 5.11 – 4.87 (m, 7H), 1.93 (q, J = 8.1 Hz, 4H), 1.67 (s, 6H), 1.59 – 1.40 (m, 7H). **^{13}C NMR** (101 MHz, $CDCl_3$) δ 200.9, 145.1, 139.8, 114.0, 113.0, 100.6, 94.9, 46.6, 34.2, 28.6, 21.1, 15.5. **HRMS** (ESI-TOF) m/z Cal. for $C_{17}H_{26}Na$ $(M+Na)^+$: 253.1927, found: 253.1939.

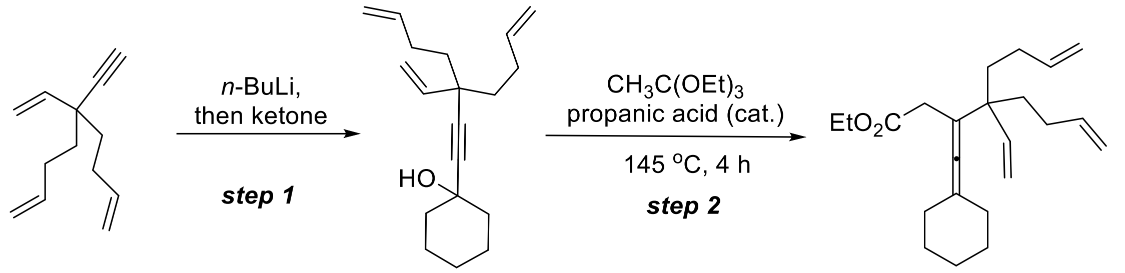

S13 **S15** **1q**

Step 1: To a solution of alkyne (400 mg, 2.2 mmol, 1.0 equiv.) in 20 mL dry THF was added *n*-BuLi (2.5 M, 0.9 mL, 1.0 equiv.) at -78 ºC, and the resulting mixture was stirred at -78 ºC for 0.5 h before the addition of cyclohexanone (250 μL, 2.4 mmol, 1.2 equiv.). The reaction was quenched by the addition of 10 mL aq. sat. NH_4Cl solution and the mixture was extracted by EtOAc (20 mL x 3). The combined organic layers were dried over Na_2SO_4 and concentrated under vacumm and the residue was purified by column chromatography on silica gel to afford propargyl alcohol **S15** as a colorless oil (500 mg, 83%).

Step 2: A 50 mL round-bottomed flask was equipped with a Dean-Stark apparatus and a condenser. Propargylic alcohol **S15** (400 mg, 2.2 mmol, 1.0 equiv.), triethyl orthoacetate (10 mL), and propanoic acid (16.3 mg. 0.44 mmol, 0.2 equiv.) were added sequentially. After the reaction had been refluxed at 145 °C for 4 h, the mixture was cooled down to 0 °C in an ice bath. Et_2O (10 mL) and HCl (aq., 1.0 M, 10 mL) were added. The organic layer was collected, and the aqueous layer was extracted with Et_2O (20 mL x 3). The combined organic layers were dried over Na_2SO_4, filtered and concentrated in vacuo. The residue was purified by column chromatography on silica gel (Petroleum ether /Et_2O = 30/1) to afford the desired product **1l** (260 mg, 55%) as a colorless oil. **^{1}H NMR** (400 MHz, $CDCl_3$) δ = 5.82 (ddt, J = 16.8, 10.1, 6.5 Hz, 2H), 5.68 (dd, J = 17.7, 10.9 Hz, 1H), 5.13 (dd, J = 10.9, 1.2 Hz, 1H), 5.07 – 4.97 (m, 3H), 4.95 – (m, 2H), 4.08 (q, J = 7.2 Hz, 2H), 2.82 (s, 2H), 2.19 – 2.04 (m, 4H), 1.95 – 1.92 (m, 4H), 1.65 – 1.43 (m, 10H), 1.24 (t, J = 7.2 Hz, 3H). **^{13}C NMR** (101 MHz, $CDCl_3$) δ = 197.6, 172.1, 144.3, 139.3, 114.0, 113.9, 105.7, 99.7, 60.4, 46.5, 35.6, 33.9, 31.6, 28.2, 27.6, 26.2, 14.2. **HRMS** (ESI-TOF) m/z Cal. for $C_{23}H_{35}O_2$ $(M+H)^+$: 343.2632, found: 343.2617.

4. Transformation of Products

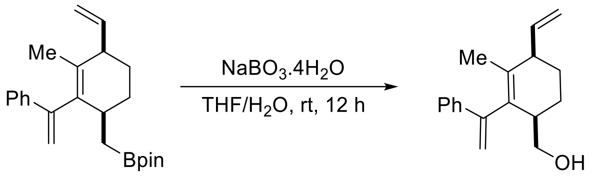

2a **4**

To a 10 mL vial was added $NaBO_3 \cdot 4H_2O$ (62.0 mg, 0.4 mmol, 4.0 equiv.), THF (0.5 mL), H_2O (0.5 mL) and **2a** (36.4 mg, 0.1 mmol, 1.0 equiv.) in sequence. The mixture was stirred at room temperature for 12 h. The mixture was extracted by EtOAc (10 mL x 3), and the combined organic layers were dried over Na_2SO_4 and concentrated in vacuo. The residue was purified by column chromatography on silica gel (Petroleum ether: EtOAc = 10:1) to afford **4** as a colorless oil (25.2 mg, 99% yield). **^{1}H NMR** (400 MHz, $CDCl_3$) δ = 7.36 – 7.15 (m, 5H), 5.75 (ddd, J = 17.5, 9.5, 8.0 Hz, 1H), 5.56 (d, J = 1.7 Hz, 1H), 5.12 – 4.98 (m, 2H), 4.93 (d, J = 1.7 Hz, 1H), 3.48 – 3.37 (m, 2H), 2.76 – 2.68 (m, 1H), 2.22 – 2.14 (m, 1H), 1.80 – 1.43 (m, 1H), 1.18 (br, 1H). **^{13}C NMR** (101 MHz, $CDCl_3$) δ = 148.5, 141.6, 139.4, 134.8, 134.1, 128.5(2), 128.4(9), 127.7, 126.2, 126.2, 115.1, 114.3, 65.0, 45.4, 40.6, 27.0, 22.3, 20.0. **HRMS** (ESI-TOF) m/z Cal. for $C_{18}H_{22}NaO$ $(M+Na)^+$: 277.1563, found: 277.1565.

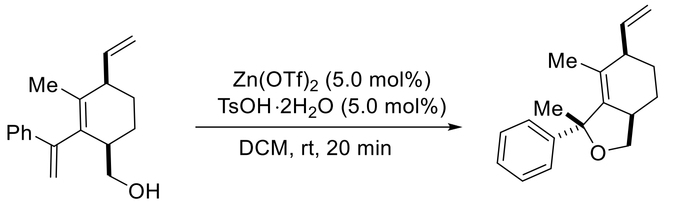

4 **5**, 99%, dr = 2:1

To a solution of **4** (25.4 mg, 0.1 mmol, 1.0 equiv.) in 2 mL dichloromethane, $Zn(OTf)_2$ (1.7 mg, 0.005 mmol, 0.05 equiv.) and TsOH (1.0 mg, 0.005 mmol, 0.05 equiv.) were added in sequence. The resulting mixture was stirred at room temperature for 20 min before the addition of 3.0 mL of Et_2O. The mixture was filtered through a short pad of silica gel followed by concentration under vacuum to give **5** as a colorless oil (25.2 mg, 99% yield, 2:1 dr). major isomer: **^{1}H NMR** (400 MHz, $CDCl_3$) δ = 7.37 – 7.32 (m, 2H), 7.28 – 7.14 (m, 3H), 5.73 (ddd, J = 17.1, 10.1, 8.2 Hz, 1H), 5.00 – 4.85 (m, 2H), 4.01 (dd, J = 8.2, 7.4 Hz, 1H), 3.35 (dd, J = 11.5, 8.1 Hz, 1H), 2.80 – 2.69 (m, 1H), 2.58 – 2.51 (m, 1H), 1.81 – 1.64 (m, 5H), 1.44 – 1.16 (m, 2H), 1.08 (dd, J = 2.5, 3H). **^{13}C NMR** (101 MHz, $CDCl_3$) δ = 145.3, 141.9, 141.5, 128.1, 127.0, 126.4, 126.0, 114.8, 83.6, 71.8, 44.4, 41.8, 28.9, 25.0, 20.4, 18.0. Minor isomer (distinct peaks) **^{1}H NMR**

(400 MHz, $CDCl_3$) δ = 7.42 – 7.38 (m, 2H), 4.07 (t, J = 7.7 Hz, 1H), 3.32 (s, 1H), 2.69 – 2.60 (m, 1H), 1.47 (d, J = 2.5, 3H). **^{13}C NMR** (101 MHz, $CDCl_3$) δ = 145.0, 142.2, 141.7, 128.2, 127.1, 125.7, 114.6, 84.5, 71.09, 44.8, 43.5, 28.6, 24.9, 20.8, 18.6. **HRMS** (ESI-TOF) m/z Cal. for $C_{18}H_{22}NaO_2$ $(M+Na)^+$: 277.1563, found: 297.1564.

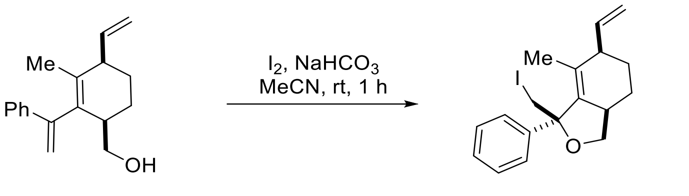

4 **6**, 82%, dr = 14:1

Prepared following a previously reported procedure.[8] To a solution of **4** (25.4 mg, 0.1 mmol, 1.0 equiv.) in 10 mL of MeCN, $NaHCO_3$ (27.4 mg, 0.3 mmol, 3.0 equiv.) and I_2 (0.54 mg, 0.2 mmol, 2.0 equiv.) were added under N_2 in sequence. The mixture was stirred at room temperature for 1 h. Then the mixture was quenched with saturated aq. $Na_2S_2O_3$, and extracted with EtOAc (10 mL x 3). The organic layers were combined, washed with brine, dried over Na_2SO_4, and concentrated in vacuo. The residue was purified by column chromatography on silica gel (Petroleum ether: EtOAc = 30:1) to afford **6** as white solid (31.2 mg, 82%, 14:1 dr). **^{1}H NMR** (400 MHz, $CDCl_3$) δ = 7.39 – 7.35 (m, 2H), 7.29 – 7.15 (m, 3H), 5.73 (ddd, J = 17.0, 10.1, 8.4 Hz, 1H), 4.96 (dd, J = 10.1, 1.8 Hz, 1H), 4.90 (dd, J = 17.0, 1.8, 1H), 4.06 (t, J = 7.9 Hz, 1H), 4.02 (d, J = 10.8 Hz, 1H), 3.68 (d, J = 10.8 Hz, 1H), 3.28 (dd, J = 11.5, 8.1 Hz, 1H), 2.90 – 2.79 (m, 1H), 2.64 (t, J = 7.4 Hz, 1H), 1.88 – 1.69 (m, 3H), 1.36 – 1.17 (m, 4H). **^{13}C NMR** (101 MHz, $CDCl_3$) δ = 141.6, 141.3, 139.3, 128.9, 128.1, 127.6, 127.1, 115.2, 84.6, 71.62, 44.7, 43.3, 28.9, 20.6, 18.1, 14.8. Minor isomer (distinct peaks): **^{1}H NMR** (400 MHz, $CDCl_3$) δ = 5.64 – 5.54 (m, 1H), 5.04 (dd, J = 17.0, 1.9 Hz, 1H), 3.12 (dd, J = 11.1, 8.3 Hz, 1H). **HRMS** (ESI-TOF) m/z Cal. for $C_{18}H_{21}NaO$ $(M+Na)^+$: 403.0529, found: 403.0527.

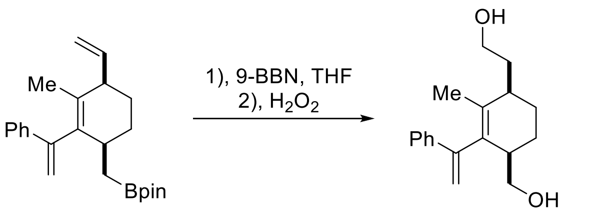

2a **7**

To a 10 mL vial was added THF (2.0 mL) and **2a** (36.4 mg, 0.1 mmol). To a solution of **2a** (36.4 mg, 0.1 mmol, 1.0 equiv.) in 2 mL THF was added a solution of 9-BBN (73.2 mg, 0.3 mmol, 3.0 equiv.) in 0.6 mL THF at 0 °C, and the resulting mixture was stirred for 6 h. To the mixture was then added 0.5 mL NaOH solution (3.0 M, aq.) and 0.5 mL

H_2O_2 solution (30% in H_2O) at 0 °C. After being stirred at room temperature for 1h, the mixture was extracted with EtOAc (10 mL x 3), the organic layers were combined, washed with brine, dried over Na_2SO_4, and concentrated in vacuo. The residue was purified by column chromatography on silica gel (Petroleum ether: EtOAc = 10:1) to afford **7** as a colorless oil (23.4 mg, 86% yield). **^{1}H NMR** (400 MHz, $CDCl_3$) δ = 7.40 – 7.24 (m, 5H), 5.62 (d, J = 1.6 Hz, 1H), 4.97 (d, J = 1.6 Hz, 1H), 3.84 – 3.68 (m, 2H), 3.55 – 3.41 (m, 2H), 2.31 – 2.19 (m, 2H), 1.99 – 1.89 (m, 1H), 1.86 – 1.59 (m, 8H), 1.44 (br, 2H). **^{13}C NMR** (101 MHz, $CDCl_3$) δ = 148.6, 139.4, 137.0, 133.5, 128.5, 127.7, 126.2, 114.3, 64.9, 61.1, 40.4, 36.9, 36.0, 25.0, 22.5, 19.9. **HRMS** (ESI-TOF) m/z Cal. for $C_{18}H_{24}NaO_2$ $(M+Na)^+$: 295.1669, found: 295.1673.

5. X-Ray Crystallographic Analysis

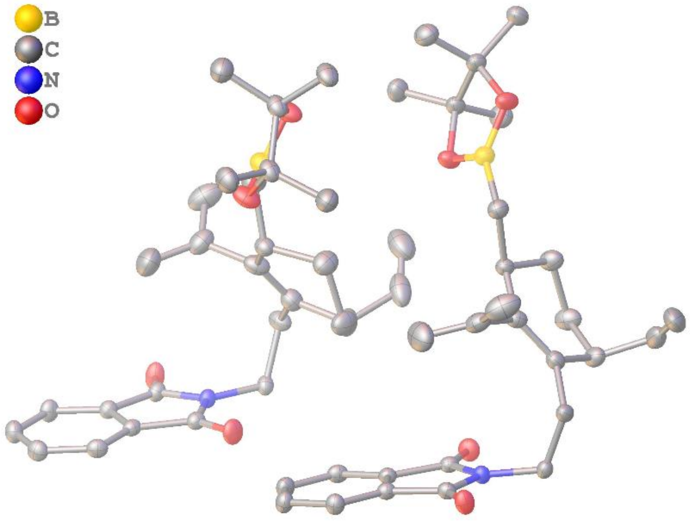

CCDC: 2354201

Table 1 Crystal data and structure refinement for 20230830_KWJ_b.

Identification code	20230830_KWJ_b
Empirical formula	$C_{56}H_{72}B_2N_2O_8$
Formula weight	922.77
Temperature/K	100.00(10)
Crystal system	monoclinic
Space group	$P2_1/n$
a/Å	10.21100(10)
b/Å	11.92230(10)
c/Å	42.7955(4)
α/°	90
β/°	96.7090(10)
γ/°	90
Volume/Å^3	5174.19(8)
Z	4
ρ_{calc}g/cm^3	1.185
μ/mm^{-1}	0.614
F(000)	1984.0

Crystal size/mm^3	0.5 × 0.2 × 0.1
Radiation	Cu Kα (λ = 1.54184)
2Θ range for data collection/°	7.702 to 160.052
Index ranges	-11 ≤ h ≤ 12, -15 ≤ k ≤ 14, -54 ≤ l ≤ 51
Reflections collected	53371
Independent reflections	10740 [R_{int} = 0.0288, R_{sigma} = 0.0225]
Data/restraints/parameters	10740/0/624
Goodness-of-fit on F^2	1.029
Final R indexes [I>=2σ (I)]	R_1 = 0.0699, wR_2 = 0.1854
Final R indexes [all data]	R_1 = 0.0767, wR_2 = 0.1904
Largest diff. peak/hole / e Å^{-3}	1.34/-0.39

Table 2 Fractional Atomic Coordinates (×10^4) and Equivalent Isotropic Displacement Parameters (Å^2×10^3) for 20230830_KWJ_b. U_{eq} is defined as 1/3 of the trace of the orthogonalised U_{IJ} tensor.

Atom	*x*	*y*	*z*	U(eq)
O1	1972.5(15)	6466.6(12)	5370.9(4)	24.5(3)
O4	2945.6(15)	10214.8(12)	5385.5(4)	24.7(3)
O6	7907.4(15)	751.5(12)	5438.1(4)	27.3(3)
O8	2754.5(16)	5021.8(13)	6896.2(3)	26.7(3)
O2	6967.8(15)	4501.0(13)	5402.3(4)	27.7(3)
O5	1807.9(16)	5952.7(13)	7278.6(3)	27.8(4)
O3	7709.2(18)	6328.1(14)	6868.9(4)	33.1(4)
O7	6742.3(18)	5511.4(14)	7268.3(4)	33.0(4)
N1	7120.9(16)	2569.1(14)	5440.6(4)	17.6(3)
N2	2131.5(16)	8401.0(14)	5384.5(4)	17.6(3)
C1	8093.4(19)	1753.9(17)	5430.3(5)	19.1(4)
C4	9050(2)	3511.8(16)	5382.9(4)	17.7(4)
C6	2623(2)	7315.5(16)	5365.8(4)	18.0(4)
C8	9338.2(19)	2371.9(16)	5400.4(5)	18.0(4)
C10	3116.2(19)	9210.4(17)	5373.8(4)	18.3(4)

Table 2 Fractional Atomic Coordinates (×10[4]) and Equivalent Isotropic Displacement Parameters (Å[2]×10[3]) for 20230830_KWJ_b. U_{eq} is defined as 1/3 of the trace of the orthogonalised U_{IJ} tensor.

Atom	*x*	*y*	*z*	U(eq)
C12	4055.7(19)	7437.7(16)	5335.2(4)	17.2(4)
C14	5008(2)	6640.1(17)	5300.6(5)	20.4(4)
C16	4351(2)	8578.1(16)	5343.1(4)	17.7(4)
C18	7618(2)	3651.5(17)	5409.0(5)	18.6(4)
C20	757.2(19)	8668.0(17)	5412.0(5)	19.3(4)
C22	10599(2)	1970.8(17)	5383.5(5)	22.1(4)
C24	10014(2)	4296.6(17)	5343.3(5)	20.9(4)
C26	5743.9(19)	2313.6(17)	5472.2(5)	19.4(4)
C28	5610(2)	8964.2(17)	5320.4(5)	21.5(4)
C30	1671(2)	6788.3(18)	6414.0(5)	20.7(4)
C32	510(2)	8978.8(18)	5746.6(5)	22.7(4)
C34	11284(2)	3900.4(18)	5321.5(5)	23.3(4)
C36	6285(2)	7023.5(18)	5276.0(5)	22.9(4)
C38	11574(2)	2761.4(18)	5343.6(5)	23.9(4)
C40	6578(2)	8165.2(18)	5286.0(5)	23.3(4)
C42	2508(2)	4174.4(19)	7128.2(5)	23.7(4)
C44	6657(2)	4417.4(19)	6426.2(5)	23.0(4)
C46	7468(2)	7235.9(19)	7083.3(5)	25.5(5)
C48	5494(2)	2062(2)	5809.2(5)	26.4(5)
C50	586(2)	7968.8(19)	5965.8(5)	23.2(4)
C52	1552(2)	7815.5(18)	6200.7(5)	23.4(4)
C54	2327(2)	4905.6(19)	7421.7(5)	25.0(5)
C56	7281(2)	6576(2)	7389.6(5)	26.6(5)
C7	2043(2)	7066.6(19)	6764.9(5)	25.7(5)
C29	-536(2)	7136(2)	5900.9(5)	29.0(5)
C15	3671(2)	3376(2)	7170.0(6)	32.6(5)
C31	1361(2)	4432(2)	7630.7(6)	32.1(5)

Table 2 Fractional Atomic Coordinates (×10^4) and Equivalent Isotropic Displacement Parameters (Å^2×10^3) for 20230830_KWJ_b. U_{eq} is defined as 1/3 of the trace of the orthogonalised U_{IJ} tensor.

Atom	*x*	*y*	*z*	U(eq)
C2	1264(2)	3557(2)	6998.9(6)	33.2(5)
C33	2660(2)	8657(2)	6263.8(5)	31.7(5)
C17	8646(2)	8019(2)	7110.2(6)	35.5(6)
C35	5557(2)	3133(2)	6009.7(5)	29.1(5)
C9	6510(2)	3346.1(19)	6238.3(5)	29.2(5)
C37	6236(2)	7848(2)	6944.5(6)	34.8(5)
C19	3626(2)	5185(2)	7619.9(6)	34.5(5)
C39	416(2)	6070(2)	6373.9(5)	29.6(5)
C5	6339(2)	7124(2)	7591.7(5)	31.6(5)
C41	4433(2)	3959(2)	5930.4(6)	34.2(5)
C21	-136(3)	5974(2)	6028.4(6)	32.8(5)
C43	7018(3)	4256(2)	6783.3(5)	31.6(5)
C11	8581(3)	6317(3)	7592.6(6)	38.8(6)
C45	7630(3)	2512(2)	6320.1(6)	37.0(6)
C23	-1799(2)	7535(3)	6019.3(6)	41.2(7)
C47	4832(3)	5145(2)	6025.1(6)	37.6(6)
C3	5403(3)	5156(2)	6371.3(6)	35.1(5)
C49	3187(3)	3602(3)	6062.8(7)	45.9(7)
B	2205(3)	6002(2)	6983.3(6)	25.5(5)
C25	2394(3)	9689(2)	6426.1(7)	49.4(8)
C51	3884(3)	8410(3)	6186.0(7)	50.1(8)
C13	-1954(3)	8428(3)	6184.1(7)	52.3(8)
C53	8880(3)	2791(3)	6250.2(8)	53.8(8)
C27	7383(4)	1516(3)	6491.1(7)	50.9(8)
C55	3054(3)	2784(3)	6250.1(8)	56.2(9)
B3	7162(3)	5378(2)	6976.4(6)	31.6(6)

Table 3 Anisotropic Displacement Parameters (Å^2×10^3) for 20230830_KWJ_b. The Anisotropic displacement factor exponent takes the form: -2π^2[h^{2}a*^{2}U$_{11}$+2hka*b*U$_{12}$+…].

Atom	U$_{11}$	U$_{22}$	U$_{33}$	U$_{23}$	U$_{13}$	U$_{12}$
O1	24.8(8)	16.6(7)	32.3(8)	0.6(6)	4.3(6)	-5.9(6)
O4	23.9(8)	14.0(7)	37.1(8)	0.3(6)	7.7(6)	0.9(6)
O6	20.5(7)	14.0(7)	46.9(9)	0.1(6)	2.4(6)	-1.4(6)
O8	32.4(8)	26.0(8)	23.0(7)	6.7(6)	9.1(6)	5.7(7)
O2	27.1(8)	16.7(7)	39.8(9)	0.5(6)	6.2(7)	7.4(6)
O5	36.3(9)	26.8(8)	21.2(7)	5.8(6)	6.7(6)	9.6(7)
O3	44.9(10)	29.1(9)	27.8(8)	-8.3(7)	15.3(7)	-6.5(7)
O7	45.3(10)	31.2(9)	24.4(8)	-8.2(7)	11.8(7)	-11.7(8)
N1	16.3(8)	15.5(8)	20.9(8)	-0.9(6)	1.7(6)	1.4(6)
N2	17.2(8)	15.4(8)	20.7(8)	1.9(6)	3.9(6)	-0.1(6)
C1	18.5(9)	16.6(9)	21.6(9)	-1.2(7)	-0.2(7)	1.3(7)
C4	20.8(10)	15.5(9)	16.4(9)	-0.8(7)	0.9(7)	1.9(7)
C6	21.0(10)	16.8(9)	16.0(9)	0.9(7)	1.3(7)	-1.0(8)
C8	19.0(10)	15.1(9)	19.5(9)	-1.0(7)	0.9(7)	-0.5(7)
C10	20.4(10)	16.5(9)	18.4(9)	1.1(7)	3.2(7)	-1.4(8)
C12	19.5(10)	15.8(9)	16.3(9)	1.7(7)	2.2(7)	-0.2(7)
C14	25.4(10)	15.0(9)	20.7(9)	1.6(7)	2.7(8)	1.0(8)
C16	20.8(10)	14.7(9)	17.5(9)	0.3(7)	2.0(7)	-0.3(7)
C18	20.6(10)	16.5(9)	18.5(9)	-1.1(7)	1.5(7)	1.6(8)
C20	15.9(9)	20.1(10)	21.7(9)	4.2(8)	1.4(7)	0.7(7)
C22	21.0(10)	14.9(9)	30.3(11)	-1.1(8)	2.3(8)	2.0(8)
C24	26.4(10)	14.5(9)	21.6(9)	0.3(7)	2.4(8)	-1.3(8)
C26	15.9(9)	20.2(10)	22.0(10)	-3.7(8)	1.5(7)	0.6(7)
C28	20.9(10)	16.5(9)	27.0(10)	0.7(8)	2.9(8)	-2.1(8)
C30	21.1(10)	22.4(10)	18.7(9)	2.8(8)	3.2(7)	1.2(8)
C32	21.1(10)	26.4(11)	21.2(10)	4.7(8)	4.5(8)	6.3(8)
C34	22.9(10)	20.6(10)	26.5(10)	0.0(8)	3.4(8)	-5.5(8)

Table 3 Anisotropic Displacement Parameters (Å^2×10^3) for 20230830_KWJ_b. The Anisotropic displacement factor exponent takes the form: -2π^2[h^{2}a*^{2}U$_{11}$+2hka*b*U$_{12}$+…].

Atom	U11	U22	U33	U23	U13	U12
C36	21.9(10)	20.5(10)	26.4(10)	3.3(8)	3.4(8)	4.6(8)
C38	18.9(10)	23.0(10)	29.9(11)	-2.2(8)	3.6(8)	-0.1(8)
C40	18.4(10)	22.0(10)	29.4(11)	2.7(8)	2.1(8)	0.6(8)
C42	24.0(10)	24.5(11)	23.5(10)	7.8(8)	5.9(8)	3.4(8)
C44	22.7(10)	26.3(11)	20.0(10)	-2.5(8)	2.6(8)	-2.9(8)
C46	25.1(11)	25.8(11)	26.5(11)	-8.6(9)	7.0(8)	-3.4(9)
C48	26.8(11)	30.1(12)	22.6(10)	-3.5(9)	4.8(8)	-9.2(9)
C50	22.9(10)	25.0(11)	22.4(10)	2.0(8)	6.2(8)	1.7(8)
C52	28.9(11)	21.6(10)	20.4(10)	1.2(8)	5.4(8)	0.1(9)
C54	25.5(11)	27.1(11)	22.6(10)	7.7(8)	4.1(8)	4.5(9)
C56	25.4(11)	29.5(12)	25.7(10)	-7.9(9)	6.0(8)	-3.3(9)
C7	26.3(11)	27.4(11)	23.3(10)	1.7(8)	1.9(8)	1.2(9)
C29	23.1(11)	36.7(13)	26.3(11)	6.7(9)	-0.6(8)	-6.4(9)
C15	29.9(12)	32.0(12)	36.7(13)	7.5(10)	7.0(10)	9.0(10)
C31	30.9(12)	39.2(13)	27.6(11)	10.2(10)	8.9(9)	3.7(10)
C2	31.2(12)	35.8(13)	32.2(12)	0.9(10)	2.5(10)	-2.1(10)
C33	33.9(12)	32.2(12)	26.7(11)	6.4(9)	-6.1(9)	-7.7(10)
C17	29.1(12)	37.9(14)	40.1(13)	-2.9(11)	6.4(10)	-8.5(11)
C35	29.7(12)	28.8(12)	29.7(11)	0.1(9)	7.1(9)	0.0(9)
C9	41.0(13)	24.0(11)	23.9(10)	-2.2(9)	9.7(9)	-1.6(10)
C37	29.9(12)	42.3(14)	31.9(12)	0.3(10)	2.0(9)	1.3(11)
C19	33.4(13)	39.9(14)	29.4(12)	2.5(10)	0.0(10)	-1.8(11)
C39	30.8(12)	29.6(12)	27.9(11)	5.7(9)	1.2(9)	-6.7(10)
C5	28.9(12)	39.3(13)	27.5(11)	-10.6(10)	6.9(9)	-1.7(10)
C41	25.1(12)	36.9(14)	39.6(13)	-9.4(11)	0.0(10)	5.9(10)
C21	37.1(13)	31.1(13)	29.3(12)	3.0(10)	0.2(10)	-12.3(10)
C43	35.1(13)	32.8(13)	26.6(11)	-1.2(9)	2.3(9)	-0.7(10)

Table 3 Anisotropic Displacement Parameters (Å^2×10^3) for 20230830_KWJ_b. The Anisotropic displacement factor exponent takes the form: -2π^2[h^{2}a*^{2}U$_{11}$+2hka*b*U$_{12}$+…].

Atom	U_{11}	U_{22}	U_{33}	U_{23}	U_{13}	U_{12}
C11	33.7(13)	46.5(16)	35.9(13)	-0.4(11)	3.1(10)	4.5(11)
C45	36.6(13)	36.1(14)	35.6(13)	-1.3(11)	-7.1(10)	4.2(11)
C23	22.1(12)	61.4(18)	40.2(14)	23.2(13)	3.9(10)	-1.3(12)
C47	42.5(14)	34.9(14)	35.0(13)	0.4(10)	2.4(11)	14.7(11)
C3	39.4(14)	33.0(13)	32.0(12)	-6.5(10)	1.4(10)	6.4(11)
C49	27.3(13)	58.7(19)	52.2(17)	-27.2(15)	6.4(12)	-1.2(12)
B	25.5(12)	27.8(13)	23.1(11)	2.5(10)	2.6(9)	1.7(10)
C25	70(2)	31.1(14)	42.3(15)	0.0(12)	-13.1(14)	-7.1(14)
C51	33.2(14)	67(2)	49.5(16)	-5.0(15)	3.0(12)	-15.5(14)
C13	34.0(15)	86(3)	39.2(15)	11.3(16)	15.6(12)	13.6(15)
C53	35.5(15)	73(2)	51.1(18)	11.1(16)	-1.4(13)	8.1(15)
C27	64(2)	37.8(16)	45.7(16)	6.1(13)	-13.7(14)	-1.3(14)
C55	43.3(17)	80(3)	47.1(17)	-16.6(17)	14.5(13)	-6.7(16)
B3	37.2(15)	31.6(14)	26.5(12)	-2.8(11)	5.7(11)	-3.0(11)

Table 4 Bond Lengths for 20230830_KWJ_b.

Atom	Atom	Length/Å	Atom	Atom	Length/Å
O1	C6	1.212(2)	C32	C50	1.523(3)
O4	C10	1.212(2)	C34	C38	1.391(3)
O6	C1	1.211(3)	C36	C40	1.393(3)
O8	C42	1.459(2)	C42	C54	1.558(3)
O8	B	1.367(3)	C42	C15	1.517(3)
O2	C18	1.210(2)	C42	C2	1.516(3)
O5	C54	1.462(3)	C44	C9	1.508(3)
O5	B	1.373(3)	C44	C43	1.541(3)
O3	C46	1.458(3)	C44	C3	1.549(3)
O3	B3	1.366(3)	C46	C56	1.559(3)

Table 4 Bond Lengths for 20230830_KWJ_b.

Atom	Atom	Length/Å	Atom	Atom	Length/Å
O7	C56	1.455(3)	C46	C17	1.517(3)
O7	B3	1.376(3)	C46	C37	1.513(3)
N1	C1	1.394(3)	C48	C35	1.536(3)
N1	C18	1.399(3)	C50	C52	1.336(3)
N1	C26	1.460(3)	C50	C29	1.517(3)
N2	C6	1.394(3)	C52	C33	1.512(3)
N2	C10	1.398(3)	C54	C31	1.515(3)
N2	C20	1.457(2)	C54	C19	1.525(3)
C1	C8	1.487(3)	C56	C5	1.515(3)
C4	C8	1.391(3)	C56	C11	1.530(3)
C4	C18	1.488(3)	C7	B	1.573(3)
C4	C24	1.383(3)	C29	C21	1.527(3)
C6	C12	1.491(3)	C29	C23	1.516(4)
C8	C22	1.383(3)	C33	C25	1.454(4)
C10	C16	1.488(3)	C33	C51	1.363(4)
C12	C14	1.380(3)	C35	C9	1.321(3)
C12	C16	1.392(3)	C35	C41	1.519(3)
C14	C36	1.397(3)	C9	C45	1.526(3)
C16	C28	1.379(3)	C39	C21	1.524(3)
C20	C32	1.528(3)	C41	C47	1.514(4)
C22	C38	1.396(3)	C41	C49	1.513(4)
C24	C34	1.393(3)	C43	B3	1.571(4)
C26	C48	1.523(3)	C45	C53	1.385(4)
C28	C40	1.393(3)	C45	C27	1.432(4)
C30	C52	1.524(3)	C23	C13	1.297(5)
C30	C7	1.541(3)	C47	C3	1.528(3)
C30	C39	1.533(3)	C49	C55	1.279(5)

Table 5 Bond Angles for 20230830_KWJ_b.

Atom	Atom	Atom	Angle/°	Atom	Atom	Atom	Angle/°
B	O8	C42	107.32(17)	O3	C46	C56	101.57(17)
B	O5	C54	106.89(17)	O3	C46	C17	108.26(18)
B3	O3	C46	107.30(18)	O3	C46	C37	107.72(19)
B3	O7	C56	106.25(18)	C17	C46	C56	114.88(19)
C1	N1	C18	111.83(16)	C37	C46	C56	113.31(19)
C1	N1	C26	123.69(17)	C37	C46	C17	110.4(2)
C18	N1	C26	124.45(16)	C26	C48	C35	111.41(18)
C6	N2	C10	111.97(16)	C52	C50	C32	123.5(2)
C6	N2	C20	124.33(17)	C52	C50	C29	121.6(2)
C10	N2	C20	123.70(16)	C29	C50	C32	114.98(18)
O6	C1	N1	124.92(19)	C50	C52	C30	124.1(2)
O6	C1	C8	129.01(19)	C50	C52	C33	121.0(2)
N1	C1	C8	106.05(17)	C33	C52	C30	114.88(18)
C8	C4	C18	108.00(17)	O5	C54	C42	102.20(16)
C24	C4	C8	121.21(19)	O5	C54	C31	109.62(18)
C24	C4	C18	130.78(18)	O5	C54	C19	107.18(19)
O1	C6	N2	124.95(19)	C31	C54	C42	114.5(2)
O1	C6	C12	128.93(19)	C31	C54	C19	109.68(19)
N2	C6	C12	106.12(16)	C19	C54	C42	113.19(19)
C4	C8	C1	108.11(17)	O7	C56	C46	102.62(16)
C22	C8	C1	130.03(19)	O7	C56	C5	109.96(18)
C22	C8	C4	121.84(19)	O7	C56	C11	107.1(2)
O4	C10	N2	124.98(19)	C5	C56	C46	114.2(2)
O4	C10	C16	129.17(19)	C5	C56	C11	109.23(19)
N2	C10	C16	105.86(16)	C11	C56	C46	113.34(19)
C14	C12	C6	130.74(18)	C30	C7	B	113.70(19)
C14	C12	C16	121.48(19)	C50	C29	C21	111.32(18)
C16	C12	C6	107.78(17)	C23	C29	C50	112.7(2)
C12	C14	C36	117.25(19)	C23	C29	C21	111.6(2)

Table 5 Bond Angles for 20230830_KWJ_b.

Atom	Atom	Atom	Angle/°	Atom	Atom	Atom	Angle/°
C12	C16	C10	108.27(17)	C25	C33	C52	118.0(2)
C28	C16	C10	130.04(18)	C51	C33	C52	120.1(2)
C28	C16	C12	121.68(19)	C51	C33	C25	121.8(3)
O2	C18	N1	124.74(19)	C9	C35	C48	123.5(2)
O2	C18	C4	129.30(19)	C9	C35	C41	120.8(2)
N1	C18	C4	105.96(16)	C41	C35	C48	115.6(2)
N2	C20	C32	113.25(16)	C44	C9	C45	113.9(2)
C8	C22	C38	117.03(19)	C35	C9	C44	125.2(2)
C4	C24	C34	117.42(19)	C35	C9	C45	120.8(2)
N1	C26	C48	113.46(16)	C21	C39	C30	111.10(18)
C16	C28	C40	117.26(19)	C47	C41	C35	111.9(2)
C52	C30	C7	113.82(18)	C49	C41	C35	112.3(2)
C52	C30	C39	112.02(17)	C49	C41	C47	112.0(2)
C39	C30	C7	109.71(17)	C39	C21	C29	109.4(2)
C50	C32	C20	112.54(18)	C44	C43	B3	114.3(2)
C38	C34	C24	121.3(2)	C53	C45	C9	118.6(3)
C40	C36	C14	121.09(19)	C53	C45	C27	122.1(3)
C34	C38	C22	121.2(2)	C27	C45	C9	119.0(3)
C28	C40	C36	121.2(2)	C13	C23	C29	127.1(3)
O8	C42	C54	101.96(17)	C41	C47	C3	109.1(2)
O8	C42	C15	108.65(17)	C47	C3	C44	110.94(19)
O8	C42	C2	106.99(17)	C55	C49	C41	127.2(3)
C15	C42	C54	114.90(18)	O8	B	O5	112.9(2)
C2	C42	C54	113.17(18)	O8	B	C7	123.1(2)
C2	C42	C15	110.4(2)	O5	B	C7	124.0(2)
C9	C44	C43	114.87(19)	O3	B3	O7	113.1(2)
C9	C44	C3	111.78(19)	O3	B3	C43	123.1(2)
C43	C44	C3	108.60(18)	O7	B3	C43	123.8(2)

Table 6 Torsion Angles for 20230830_KWJ_b.

A	B	C	D	Angle/°	A	B	C	D	Angle/°
O1	C6	C12	C14	1.0(4)	C32	C50	C29	C23	-76.9(2)
O1	C6	C12	C16	-179.7(2)	C42	O8	B	O5	10.4(3)
O4	C10	C16	C12	-179.1(2)	C42	O8	B	C7	-169.2(2)
O4	C10	C16	C28	0.1(4)	C44	C9	C45	C53	-66.6(3)
O6	C1	C8	C4	176.4(2)	C44	C9	C45	C27	107.9(3)
O6	C1	C8	C22	-2.1(4)	C44	C43	B3	O3	-37.9(4)
O8	C42	C54	O5	28.9(2)	C44	C43	B3	O7	141.5(2)
O8	C42	C54	C31	147.34(18)	C46	O3	B3	O7	-9.1(3)
O8	C42	C54	C19	-86.0(2)	C46	O3	B3	C43	170.4(2)
O3	C46	C56	O7	-29.6(2)	C48	C35	C9	C44	175.7(2)
O3	C46	C56	C5	-148.58(19)	C48	C35	C9	C45	-0.5(4)
O3	C46	C56	C11	85.5(2)	C48	C35	C41	C47	-154.9(2)
N1	C1	C8	C4	-2.1(2)	C48	C35	C41	C49	78.2(3)
N1	C1	C8	C22	179.3(2)	C50	C52	C33	C25	77.8(3)
N1	C26	C48	C35	73.4(2)	C50	C52	C33	C51	-106.1(3)
N2	C6	C12	C14	-178.2(2)	C50	C29	C21	C39	52.6(3)
N2	C6	C12	C16	1.1(2)	C50	C29	C23	C13	-6.0(4)
N2	C10	C16	C12	0.3(2)	C52	C30	C7	B	-178.31(19)
N2	C10	C16	C28	179.5(2)	C52	C30	C39	C21	41.2(3)
N2	C20	C32	C50	-72.6(2)	C52	C50	C29	C21	-23.7(3)
C1	N1	C18	O2	178.00(19)	C52	C50	C29	C23	102.6(3)
C1	N1	C18	C4	-1.3(2)	C54	O5	B	O8	9.7(3)
C1	N1	C26	C48	80.3(2)	C54	O5	B	C7	-170.7(2)
C1	C8	C22	C38	177.6(2)	C56	O7	B3	O3	-11.4(3)
C4	C8	C22	C38	-0.8(3)	C56	O7	B3	C43	169.2(2)
C4	C24	C34	C38	-0.9(3)	C7	C30	C52	C50	-136.9(2)
C6	N2	C10	O4	179.90(19)	C7	C30	C52	C33	45.6(3)
C6	N2	C10	C16	0.4(2)	C7	C30	C39	C21	168.7(2)
C6	N2	C20	C32	101.9(2)	C29	C50	C52	C30	3.0(3)

Table 6 Torsion Angles for 20230830_KWJ_b.

A	B	C	D	Angle/°	A	B	C	D	Angle/°
C6	C12	C14	C36	179.66(19)	C29	C50	C52	C33	-179.6(2)
C6	C12	C16	C10	-0.9(2)	C15	C42	C54	O5	146.24(19)
C6	C12	C16	C28	179.87(18)	C15	C42	C54	C31	-95.4(2)
C8	C4	C18	O2	-179.4(2)	C15	C42	C54	C19	31.3(3)
C8	C4	C18	N1	-0.1(2)	C2	C42	C54	O5	-85.6(2)
C8	C4	C24	C34	0.0(3)	C2	C42	C54	C31	32.8(3)
C8	C22	C38	C34	-0.1(3)	C2	C42	C54	C19	159.5(2)
C10	N2	C6	O1	179.78(19)	C17	C46	C56	O7	-146.20(19)
C10	N2	C6	C12	-1.0(2)	C17	C46	C56	C5	94.8(2)
C10	N2	C20	C32	-78.1(2)	C17	C46	C56	C11	-31.1(3)
C10	C16	C28	C40	-178.5(2)	C35	C9	C45	C53	110.1(3)
C12	C14	C36	C40	0.0(3)	C35	C9	C45	C27	-75.5(3)
C12	C16	C28	C40	0.6(3)	C35	C41	C47	C3	-53.1(3)
C14	C12	C16	C10	178.52(17)	C35	C41	C49	C55	7.5(4)
C14	C12	C16	C28	-0.7(3)	C9	C44	C43	B3	-179.7(2)
C14	C36	C40	C28	-0.1(3)	C9	C44	C3	C47	-40.4(3)
C16	C12	C14	C36	0.4(3)	C9	C35	C41	C47	25.2(3)
C16	C28	C40	C36	-0.2(3)	C9	C35	C41	C49	-101.7(3)
C18	N1	C1	O6	-176.5(2)	C37	C46	C56	O7	85.6(2)
C18	N1	C1	C8	2.1(2)	C37	C46	C56	C5	-33.3(3)
C18	N1	C26	C48	-101.5(2)	C37	C46	C56	C11	-159.3(2)
C18	C4	C8	C1	1.4(2)	C39	C30	C52	C50	-11.8(3)
C18	C4	C8	C22	-179.93(18)	C39	C30	C52	C33	170.7(2)
C18	C4	C24	C34	-178.94(19)	C39	C30	C7	B	55.3(2)
C20	N2	C6	O1	-0.2(3)	C41	C35	C9	C44	-4.4(4)
C20	N2	C6	C12	179.08(16)	C41	C35	C9	C45	179.4(2)
C20	N2	C10	O4	-0.1(3)	C41	C47	C3	C44	61.9(3)
C20	N2	C10	C16	-179.60(17)	C21	C29	C23	C13	120.2(3)
C20	C32	C50	C52	110.7(2)	C43	C44	C9	C35	136.4(2)

Table 6 Torsion Angles for 20230830_KWJ_b.

A	B	C	D	Angle/°	A	B	C	D	Angle/°
C20	C32	C50	C29	-69.9(2)	C43	C44	C9	C45	-47.1(3)
C24	C4	C8	C1	-177.81(18)	C43	C44	C3	C47	-168.2(2)
C24	C4	C8	C22	0.9(3)	C23	C29	C21	C39	-74.3(3)
C24	C4	C18	O2	-0.3(4)	C47	C41	C49	C55	-119.4(3)
C24	C4	C18	N1	179.0(2)	C3	C44	C9	C35	12.1(3)
C24	C34	C38	C22	1.0(3)	C3	C44	C9	C45	-171.4(2)
C26	N1	C1	O6	1.9(3)	C3	C44	C43	B3	-53.7(3)
C26	N1	C1	C8	-179.44(16)	C49	C41	C47	C3	74.0(3)
C26	N1	C18	O2	-0.4(3)	B	O8	C42	C54	-24.3(2)
C26	N1	C18	C4	-179.74(16)	B	O8	C42	C15	-146.0(2)
C26	C48	C35	C9	-110.4(3)	B	O8	C42	C2	94.7(2)
C26	C48	C35	C41	69.7(3)	B	O5	C54	C42	-23.9(2)
C30	C52	C33	C25	-104.7(2)	B	O5	C54	C31	-145.7(2)
C30	C52	C33	C51	71.5(3)	B	O5	C54	C19	95.4(2)
C30	C7	B	O8	37.8(3)	B3	O3	C46	C56	23.8(2)
C30	C7	B	O5	-141.8(2)	B3	O3	C46	C17	145.1(2)
C30	C39	C21	C29	-62.9(3)	B3	O3	C46	C37	-95.5(2)
C32	C50	C52	C30	-177.56(19)	B3	O7	C56	C46	25.3(2)
C32	C50	C52	C33	-0.2(3)	B3	O7	C56	C5	147.1(2)
C32	C50	C29	C21	156.81(19)	B3	O7	C56	C11	-94.3(2)

Table 7 Hydrogen Atom Coordinates (Å×10^4) and Isotropic Displacement Parameters (Å^2×10^3) for 20230830_KWJ_b.

Atom	*x*	*y*	*z*	U(eq)
H14	4803.06	5862.48	5293.82	24
H20A	203.51	8012.85	5341.9	23
H20B	481.59	9301.82	5269.9	23
H22	10793.01	1191.97	5398.41	27
H24	9818.49	5075.47	5331.32	25

Table 7 Hydrogen Atom Coordinates (Å×10[4]) and Isotropic Displacement Parameters (Å[2]×10[3]) for 20230830_KWJ_b.

Atom	*x*	*y*	*z*	U(eq)
H26A	5467.95	1657.67	5338.6	23
H26B	5190.77	2958.77	5393.34	23
H28	5808.09	9742.91	5327.97	26
H30	2397.95	6315.56	6347.46	25
H32A	1171.53	9541.42	5831.32	27
H32B	-373.02	9326.22	5740.74	27
H34	11964.86	4418.2	5291.04	28
H36	6964.17	6497.01	5252	27
H38	12451.91	2516.91	5331.26	29
H40	7454.92	8403.26	5269.1	28
H44	7391.34	4852.24	6348.41	28
H48A	6162.2	1521.65	5903.71	32
H48B	4614.45	1712.21	5808.23	32
H7A	1353.23	7556.54	6835.96	31
H7B	2881.49	7492.22	6788.69	31
H29	-723.03	7063.22	5667.13	35
H15A	3735.55	2976.81	6972.1	49
H15B	3545.83	2832.74	7335.88	49
H15C	4483.66	3800.9	7228.97	49
H31A	492.24	4352.74	7508.81	48
H31B	1296.85	4941.75	7807.93	48
H31C	1670.49	3696.54	7710.36	48
H2A	512.99	4073.41	6984.6	50
H2B	1108.65	2933.44	7139.25	50
H2C	1366.15	3263.6	6789.14	50
H17A	8531.07	8605.69	7265.02	53
H17B	8719.01	8364.63	6905.1	53
H17C	9450.64	7593.07	7177.64	53

Table 7 Hydrogen Atom Coordinates (Å×10^4) and Isotropic Displacement Parameters (Å^2×10^3) for 20230830_KWJ_b.

Atom	*x*	*y*	*z*	U(eq)
H37A	5475.32	7344.62	6940.01	52
H37B	6340.3	8088.66	6729.9	52
H37C	6096.46	8505.54	7073.83	52
H19A	3975.44	4510.36	7730.76	52
H19B	3475.74	5767.28	7773.41	52
H19C	4261.89	5457.97	7482.75	52
H39A	619.52	5311.57	6460.57	36
H39B	-255.28	6411.41	6493.55	36
H5A	6662.96	7874.42	7654.4	47
H5B	6279.54	6667.61	7779.91	47
H5C	5463.99	7186.14	7471.48	47
H41	4227.86	3958	5696.15	41
H21A	-911.61	5470.16	6006.54	39
H21B	539.81	5650.98	5906.61	39
H43A	7859.76	3838.06	6819.42	38
H43B	6329.13	3790.01	6864.32	38
H11A	9211.26	6003.21	7460.25	58
H11B	8422.2	5772.39	7755.66	58
H11C	8941.16	7008.67	7692.06	58
H23	-2558.29	7082.42	5967.14	49
H47A	4054.52	5645.74	5992.72	45
H47B	5499.2	5421.25	5893.36	45
H3A	4734.02	4872.13	6501.52	42
H3B	5624.26	5935.43	6437.3	42
H49	2418.61	4032.52	5999.89	55
H25A	1687.37	10104.17	6301.21	74
H25B	3193.34	10150.03	6454.2	74
H25C	2120.4	9508.87	6632.35	74

Table 7 Hydrogen Atom Coordinates (Å×10^4) and Isotropic Displacement Parameters (Å^2×10^3) for 20230830_KWJ_b.

**Atom**	***x***	***y***	***z***	**U(eq)**
H51A	4592.43	8918.29	6237.75	60
H51B	4029	7728.86	6079.9	60
H13A	-1226.91	8911.15	6242.92	63
H13B	-2795.05	8599.2	6246.09	63
H53A	8847.26	2953.13	6024.91	81
H53B	9482.64	2162.69	6304.69	81
H53C	9195.16	3454.9	6371.51	81
H27A	8088.29	1022.9	6560.36	61
H27B	6513.66	1349.05	6535.16	61
H55A	3790.32	2325.47	6320.71	67
H55B	2219.2	2635.98	6318.65	67

6. Determination of the Relative Configuration of 3k

The assignment of ^{1}H and ^{13}C NMR chemical shifts for compound **3k** was achieved by analyzing its ^{1}H NMR, ^{13}C NMR, HSQC and HMBC spectrums (**Figure S1**).

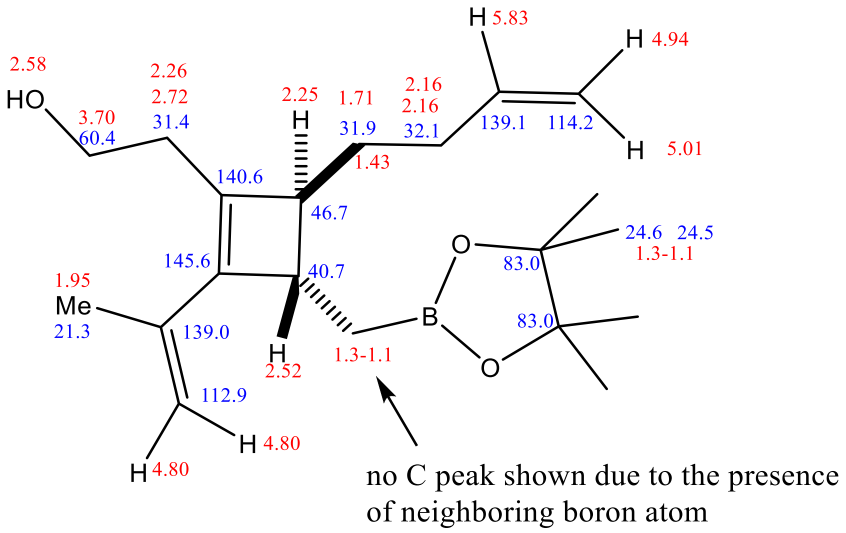

Figure S1. The assignment of ^{1}H and ^{13}C NMR chemical shifts of compound **3k**.

The *trans* configuration of **3k** was confirmed by its NOE spectrums: There was a NOE effect between H^a and H^c; The NOE effect was also observed between H^b and H^d or H$^{d'}$. The ^{1}H-^{1}H COSY spectrum of **3k** showed no coupling between the two protons on the cyclobutene ring further verified its relative structure, as *cis* coupling will give a higher coupling constant than a trans coupling in a four-membered ring.

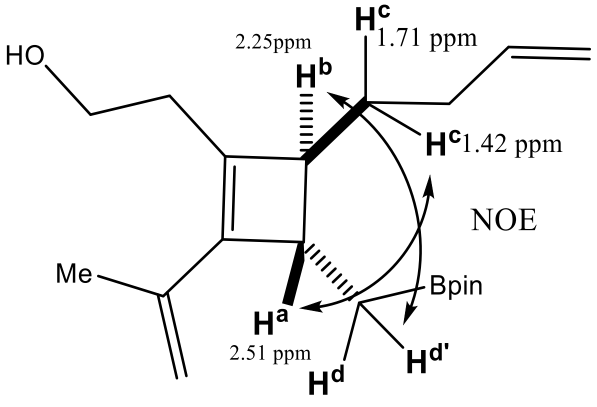

Figure S2. NOE effect H^a/ H^c, and H^b/H$^{d\ or\ d'}$ for compound **3k**

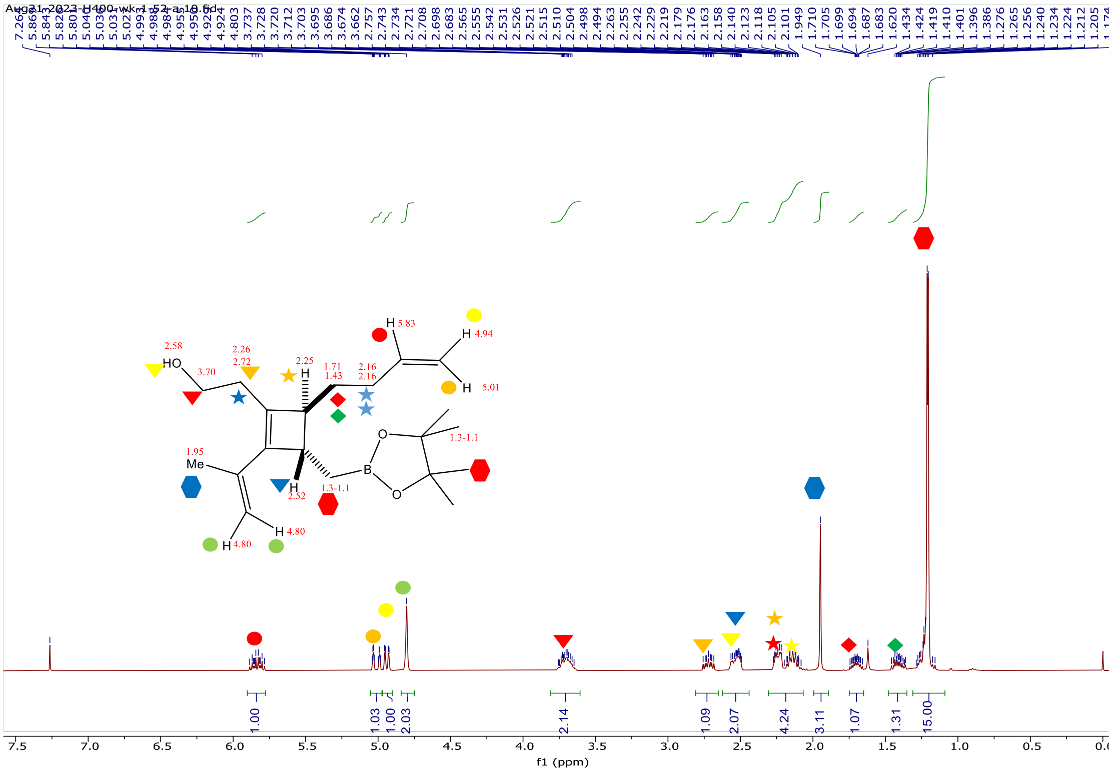
Aug21-2023-H400-wk-1-52-a.10.fid
HO
Me
O
B
O
H 5.83
H 4.94
H 5.01
2.58
3.70
2.26
2.72
2.25
1.71
1.43
2.16
2.16
1.3-1.1
1.95
2.52
1.3-1.1
H 4.80
H 4.80
1.00
1.03
1.00
2.03
2.14
1.09
2.07
4.24
3.11
1.07
1.31
15.00
f1 (ppm)

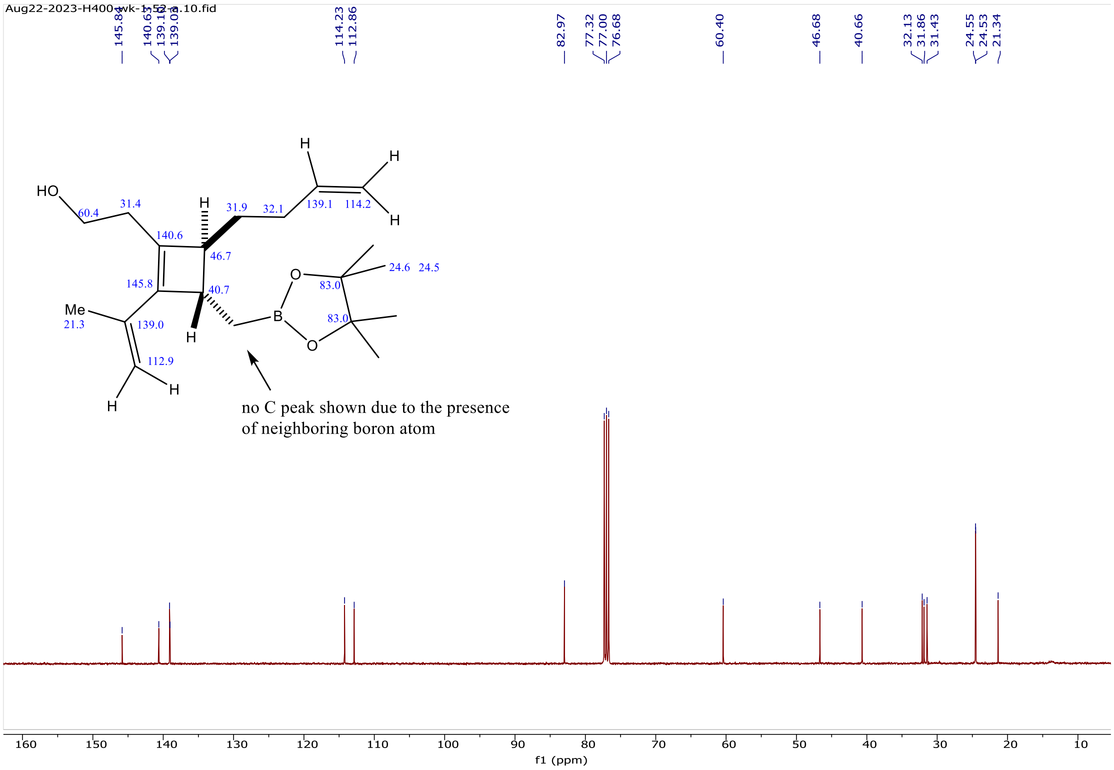
Aug22-2023-H400-wk-1-52-a.10.fid
145.84
140.61
139.13
139.04
114.23
112.86
82.97
77.32
77.00
76.68
60.40
46.68
40.66
32.13
31.86
31.43
24.55
24.53
21.34
HO
60.4
31.4
140.6
145.8
46.7
40.7
31.9
32.1
139.1
114.2
83.0
83.0
24.6 24.5
Me
21.3
139.0
112.9
no C peak shown due to the presence
of neighboring boron atom
f1 (ppm)

HSQC spectrum of 3k

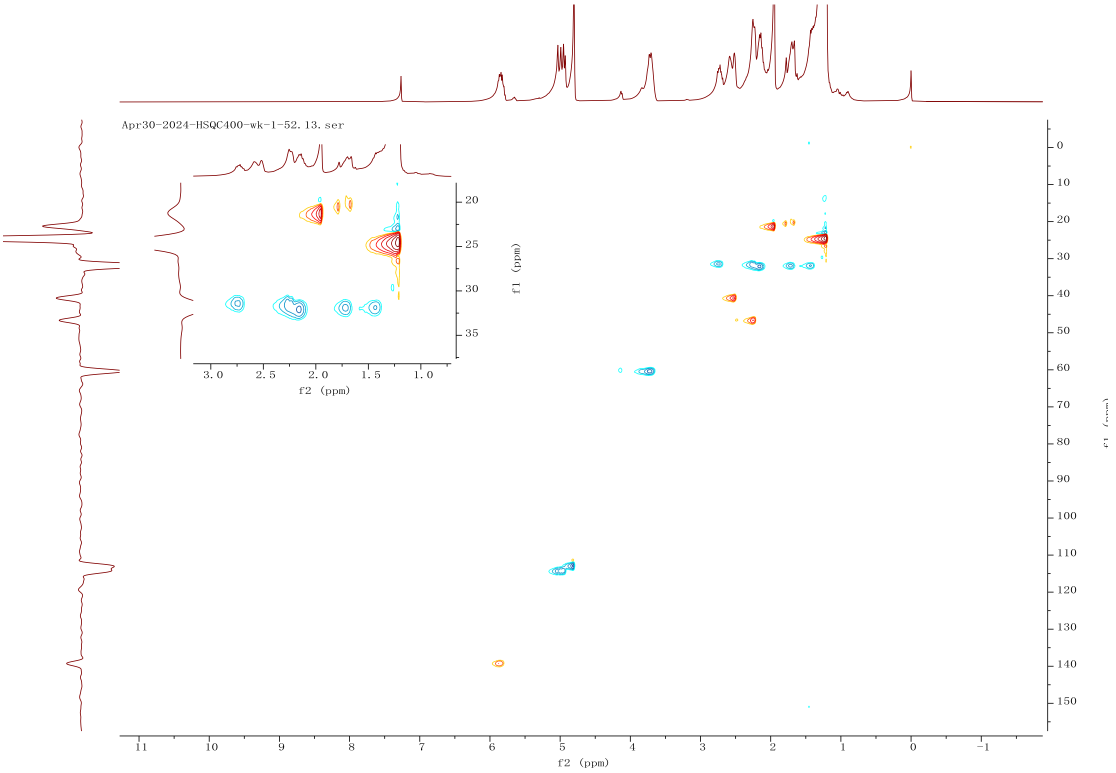

HMBC spectrum of 3k

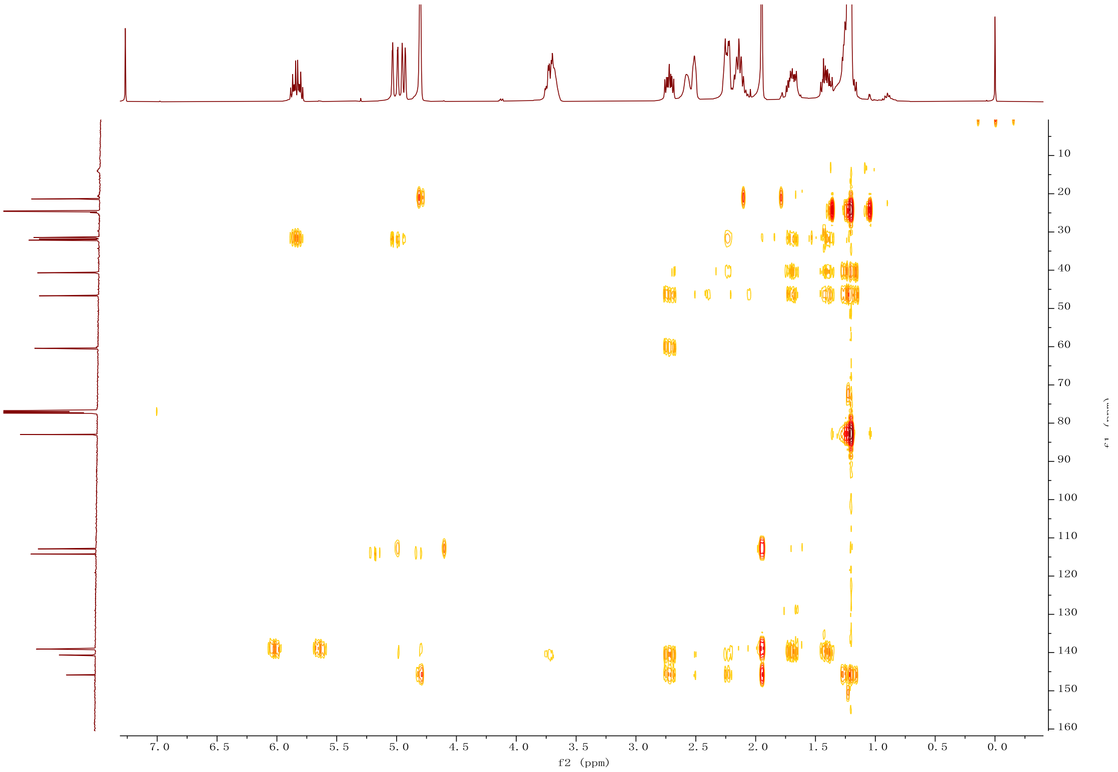

NOESY spectrum of 3k (full)

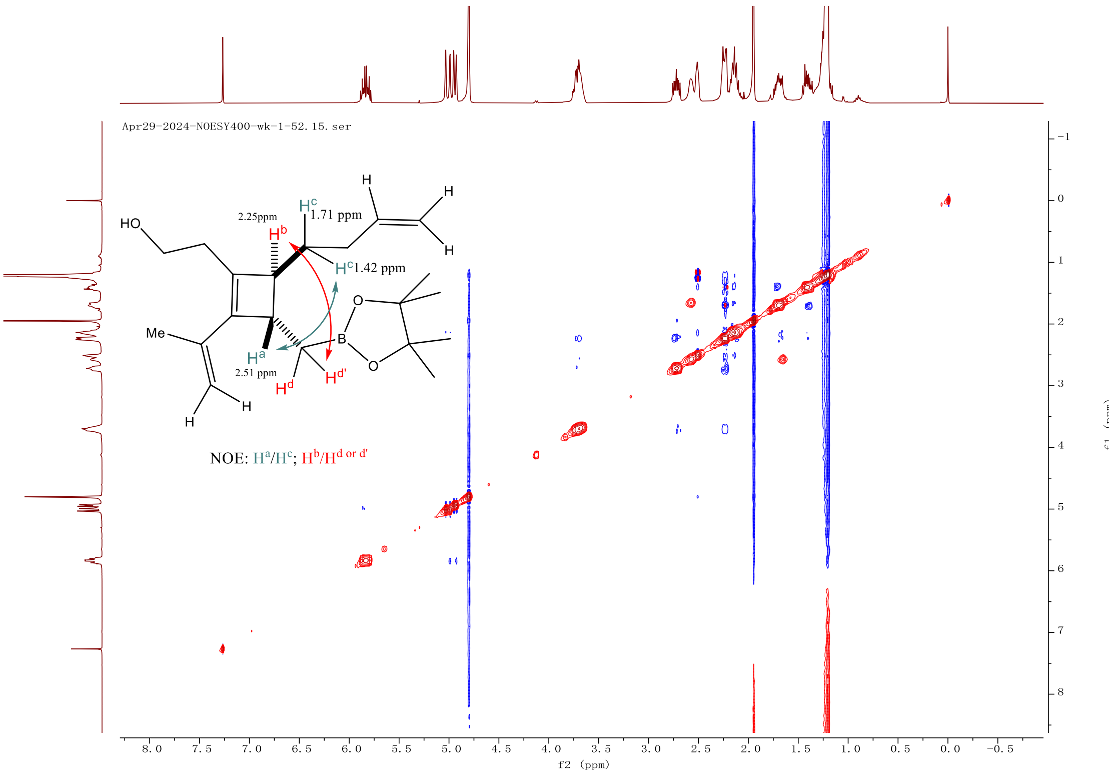

NOESY spectrum of 3k (zoom in)

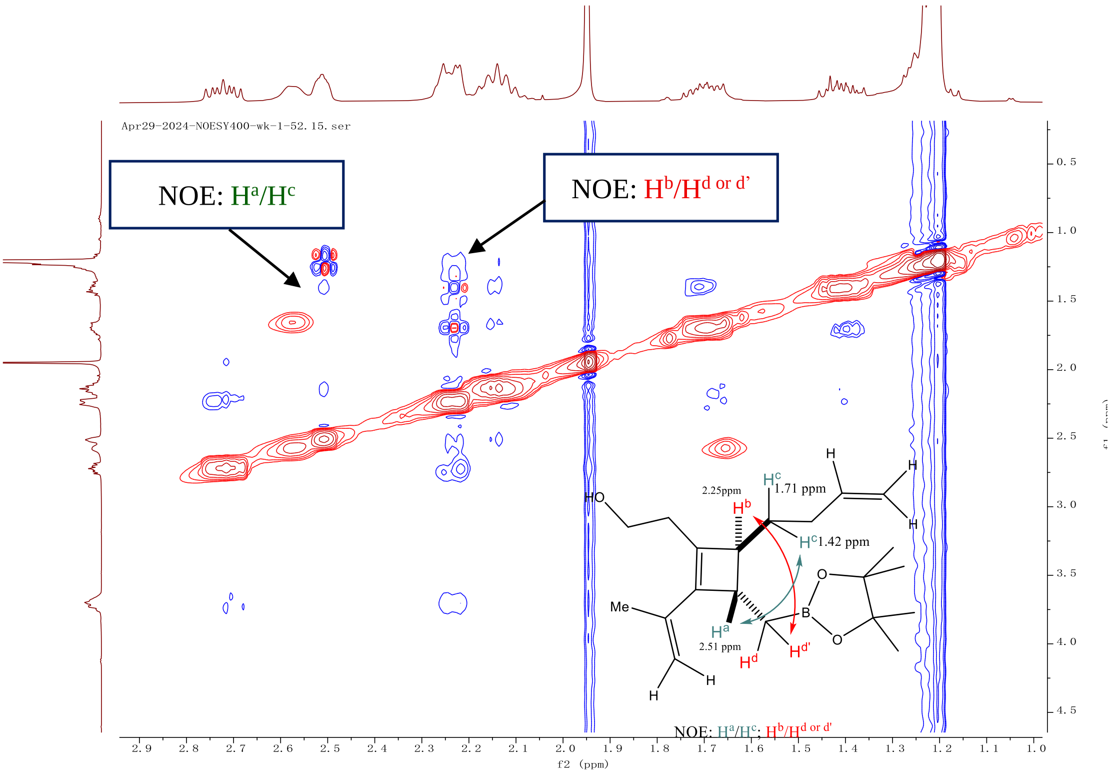

^{1}H-^{1}H COSY of 3k

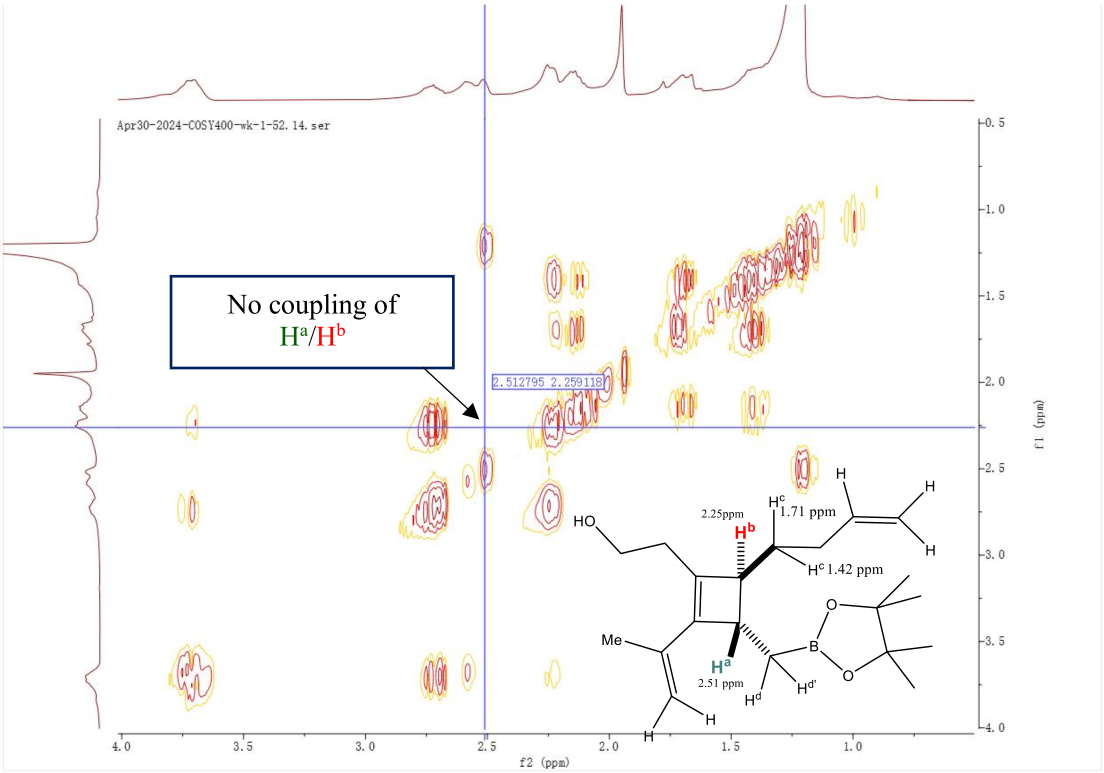

7. Computational Details

All density functional calculations in the present study were performed by using the B3LYP-D3 functional (with BJ dispersion),[1,2,3] as implemented in the Gaussian 16 program package.[4] For the geometry optimizations, the LANL2DZ pseudopotential[5] and its corresponding basis sets were used for Pd, and the 6-31G(d,p) basis sets were used for other atoms. Frequency calculations were carried out at the same level of theory as the geometry optimization to obtain the Gibbs free energy corrections and to identify all the stationary points as minima (no imaginary frequency) or transition states (one imaginary frequency). To obtain more accurate energies, single-point calculations using these optimized geometries were done employing a larger basis set, where all elements were described by 6-311+G(2d,2p), except Pd for which the LANL2TZ pseudopotential was used. The solvation effects of the toluene solvent were evaluated as single-point corrections using the SMD model.[6] The standard state correction of +1.9 kcal/mol was added for all species. The 3D images of the computed structures were prepared using CYLView.[7]

References

(1) Becke, A. D. Density-functional thermochemistry. III. The role of exact exchange. *J. Chem. Phys.* **1993**, *98*, 5648–5652.

(2) Grimme, S.; Antony, J.; Ehrlich, S.; Krieg, H. A consistent and accurate ab initio parametrization of density functional dispersion correction (DFT-D) for the 94 elements H-Pu. *J. Chem. Phys.* **2010**, *132*, 154104.

(3) Grimme, S.; Ehrlich, S.; Goerigk, L. Effect of the damping function in dispersion corrected density functional theory. *J. Comput. Chem.* **2011**, *32*, 1456–1465.

(4) Gaussian 16, Revision C.01, M. J. Frisch, G. W. Trucks, H. B. Schlegel, G. E. Scuseria, M. A. Robb, J. R. Cheeseman, G. Scalmani, V. Barone, G. A. Petersson, H. Nakatsuji, X. Li, M. Caricato, A. V. Marenich, J. Bloino, B. G. Janesko, R. Gomperts, B. Mennucci, H. P. Hratchian, J. V. Ortiz, A. F. Izmaylov, J. L. Sonnenberg, D. Williams-Young, F. Ding, F. Lipparini, F. Egidi, J. Goings, B. Peng, A. Petrone, T. Henderson, D. Ranasinghe, V. G. Zakrzewski, J. Gao, N. Rega, G. Zheng, W. Liang, M. Hada, M. Ehara, K. Toyota, R. Fukuda, J. Hasegawa, M. Ishida, T. Nakajima, Y. Honda, O. Kitao, H. Nakai, T. Vreven, K. Throssell, J. A. Montgomery, Jr., J. E. Peralta, F. Ogliaro, M. J. Bearpark, J. J. Heyd, E. N. Brothers, K. N. Kudin, V. N. Staroverov, T. A. Keith, R. Kobayashi, J. Normand, K. Raghavachari, A. P. Rendell, J. C. Burant, S. S. Iyengar, J. Tomasi, M. Cossi, J. M. Millam, M. Klene, C. Adamo, R. Cammi, J. W. Ochterski, R. L. Martin, K. Morokuma, O. Farkas, J. B. Foresman, and D. J. Fox, Gaussian, Inc., Wallingford CT, 2019.

(5) Hay, P. J.; Wadt, W. R. Ab initio effective core potentials for molecular calculations. Potentials for K to Au including the outermost core orbitals. *J. Chem. Phys.* **1985**, 82, 299–310.

(6) Marenich, A. V.; Cramer, J.; Truhlar, D. G. Universal Solvation Model Based on Solute Electron Density and on a Continuum Model of the Solvent Defined by the Bulk Dielectric Constant and Atomic Surface Tensions. *J. Phys. Chem. B* **2009**, *113*, 6378–6396.

(7) CYLview, 1.0b & Legault, C. Y. Université de Sherbrooke, 2009 (http://www.cylview.org).

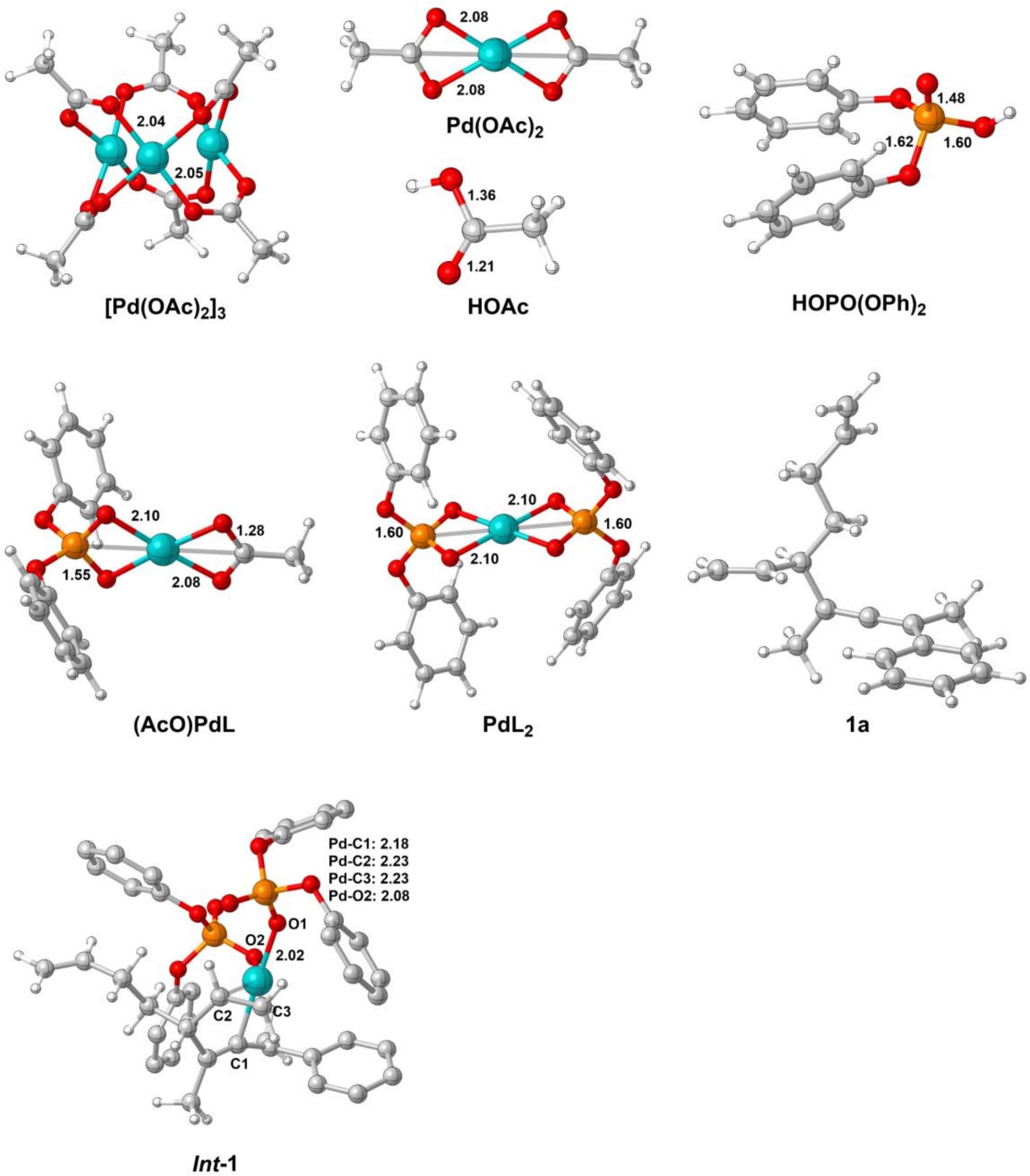

Figure S3. Optimized structures of the stationary points. Distances are given in Å. The hydrogen atoms of the phenyl groups in the Pd-complexes are omitted for clarity.

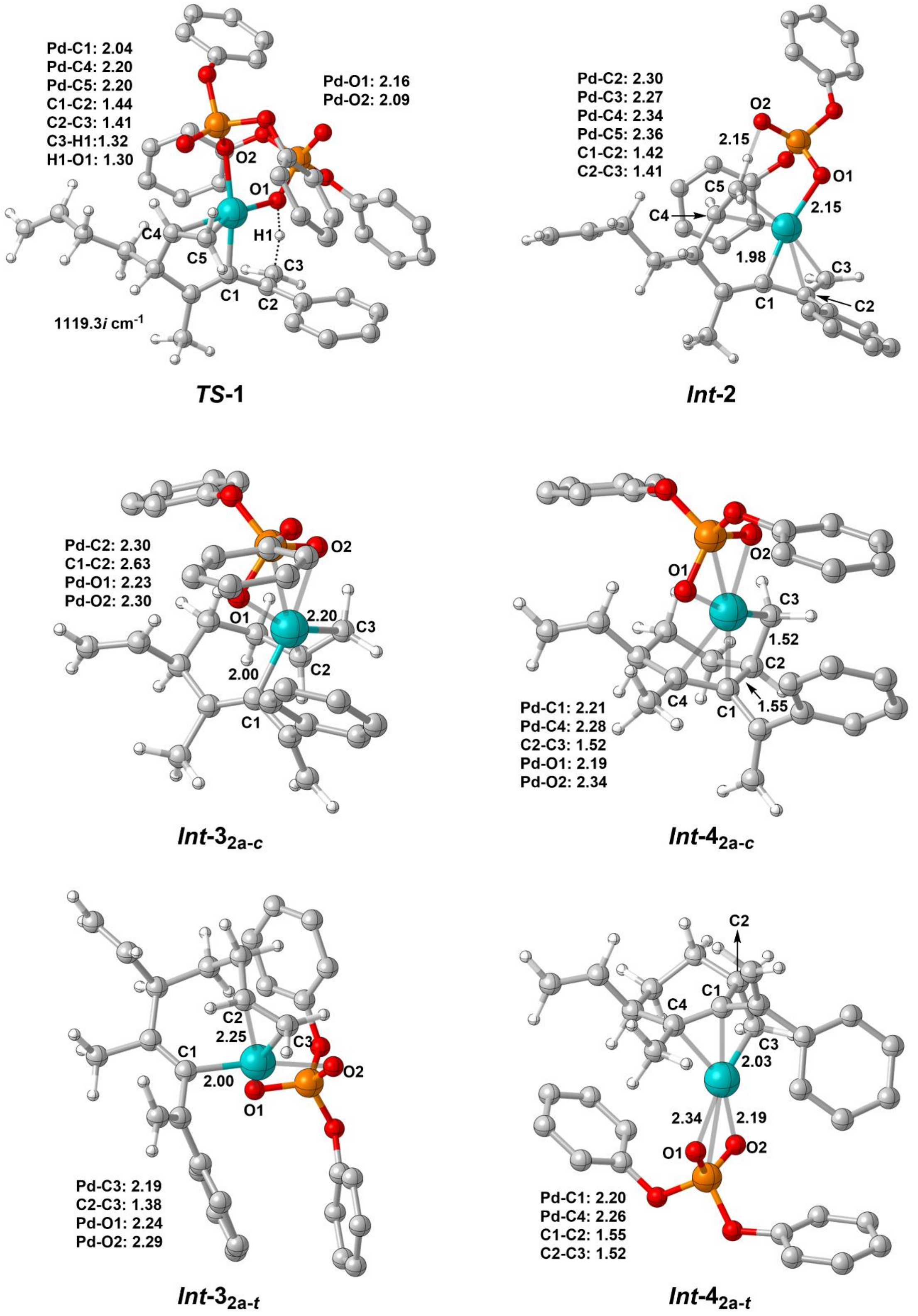

Figure S4. Optimized structures of the stationary points. Distances are given in Å. The hydrogen atoms of the phenyl groups are omitted for clarity.

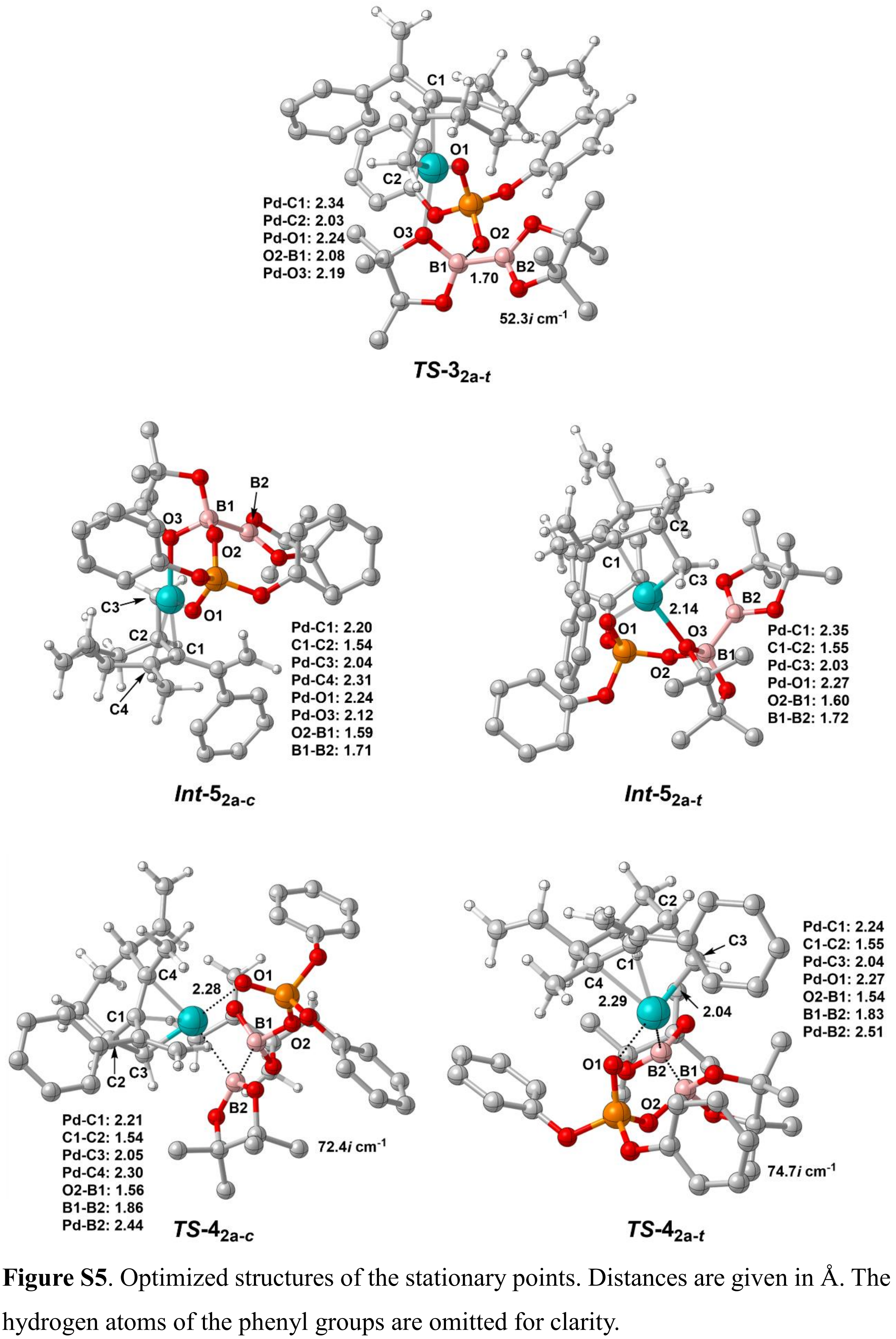

Figure S5. Optimized structures of the stationary points. Distances are given in Å. The hydrogen atoms of the phenyl groups are omitted for clarity.

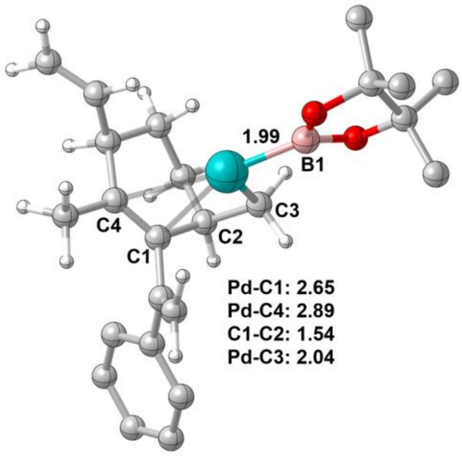

***Int*-6$_{2a\text{-}c}$**

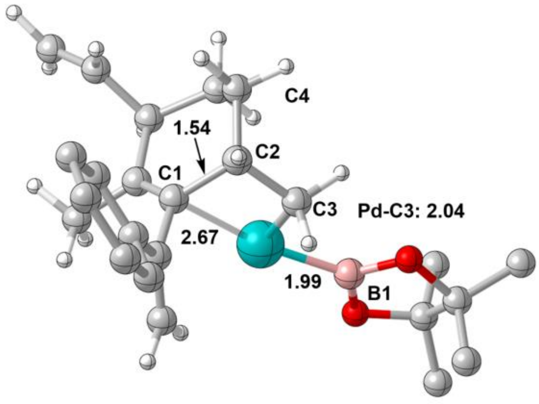

***Int*-6$_{2a\text{-}t}$**

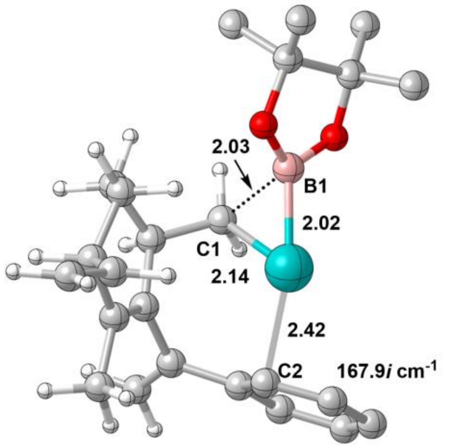

***TS*-5$_{2a\text{-}c}$**

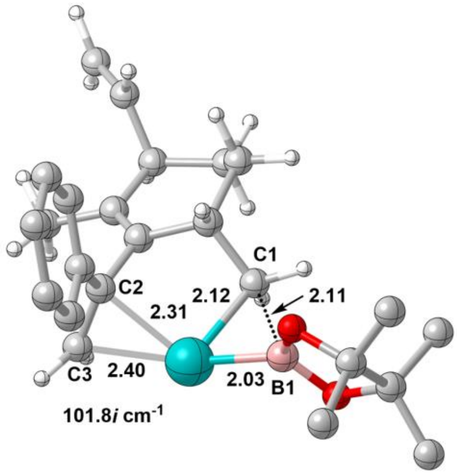

***TS*-5$_{2a\text{-}t}$**

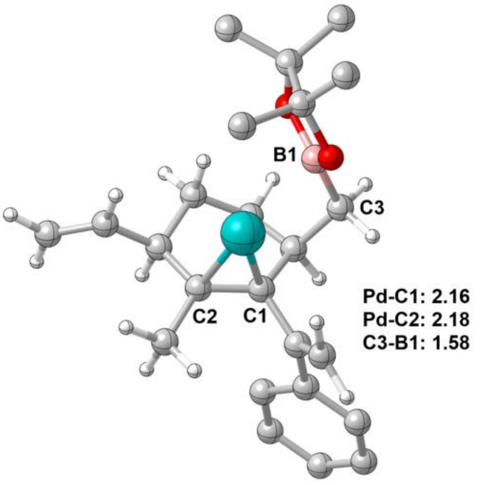

***Int*-7$_{2a\text{-}c}$**

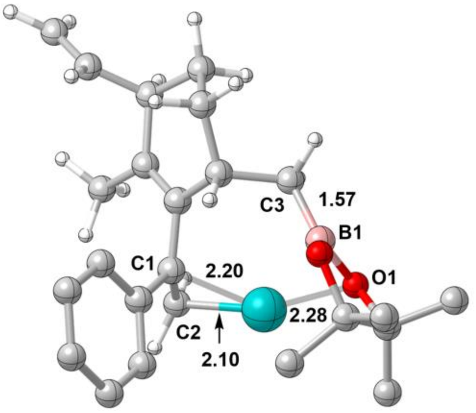

***Int*-7$_{2a\text{-}t}$**

Figure S6. Optimized structures of the stationary points. Distances are given in Å. The hydrogen atoms of the phenyl groups are omitted for clarity.

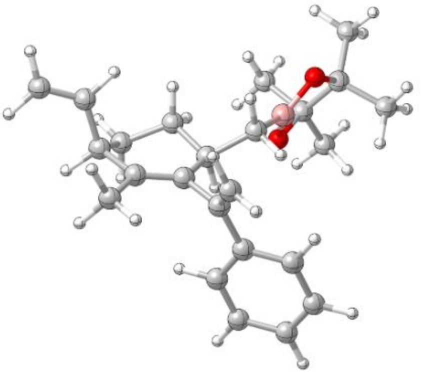

***cis*-2a**

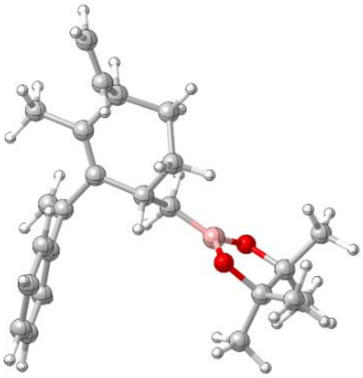

***trans*-2a**

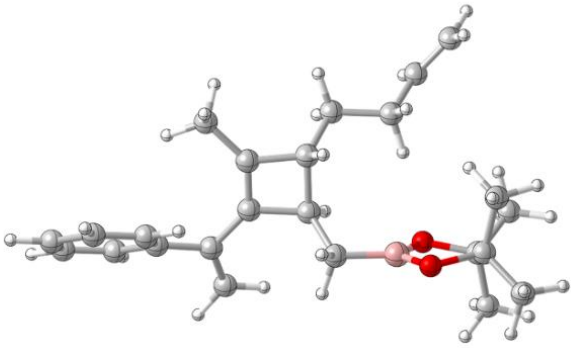

***trans*-3a**

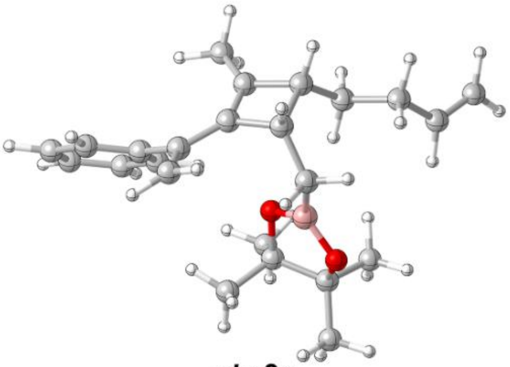

***cis*-3a**

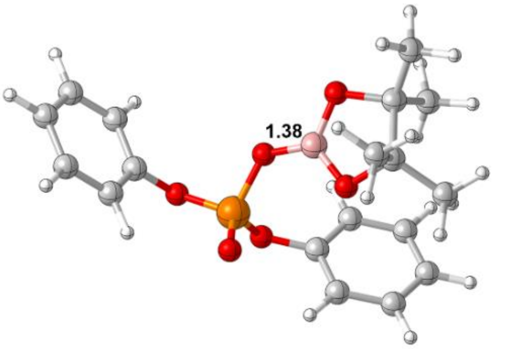

Bpin-OPO(OPh)$_2$

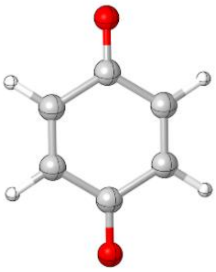

BQ

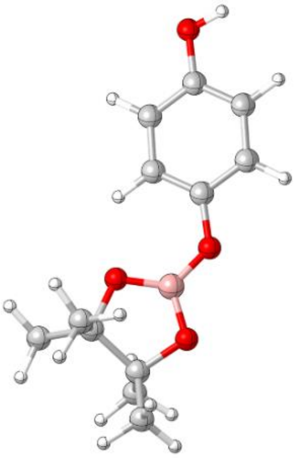

HO(C$_6$H$_4$)OBpin

Figure S7. Optimized structures of the stationary points. Distances are given in Å.

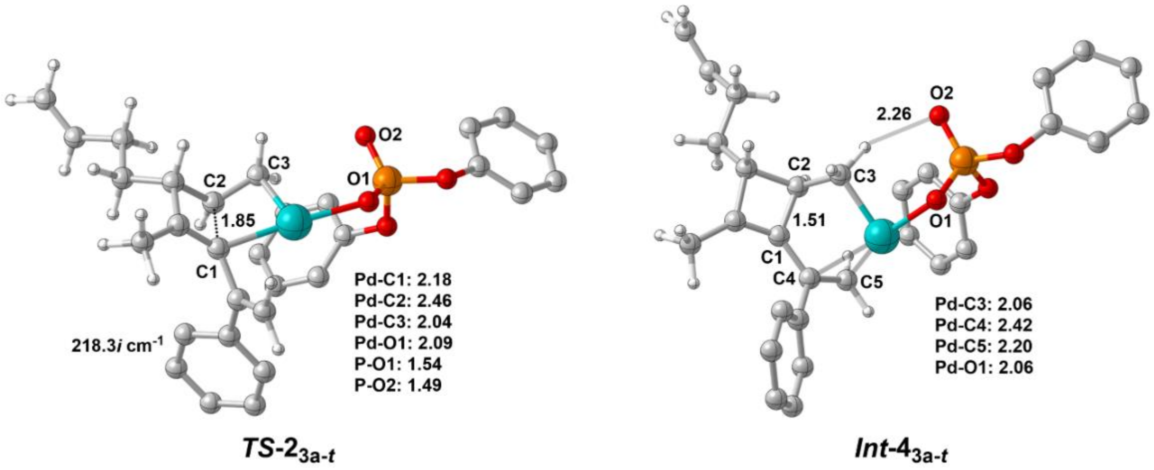

Figure S8. Optimized structures of the stationary points. Distances are given in Å. The hydrogen atoms of the phenyl groups are omitted for clarity.

Absolute energies and energy corrections

Table S1. Absolute energies and energy corrections (a.u.).

Structure	Electronic energy 6-31G(d,p)/LANL2DZ	Solvation correction	Thermal correction to Gibbs free energy	Single-point energy 6-311+G(2d,2p)/LANL2TZ
**[Pd(OAc)$_2$]$_3$**	-1751.43792	-0.01939	0.24196	-1751.91399
**Pd(OAc)$_2$**	-583.75907	-0.00997	0.06404	-583.92467
**HOAc**	-229.09441	-0.00579	0.03489	-229.17924
**HOPO(OPh)$_2$**	-1106.29331	-0.01682	0.16777	-1106.58172
**(AcO)PdL**	-1460.96567	-0.02117	0.19670	-1461.33553
**PdL$_2$**	-2338.17950	-0.03093	0.33385	-2338.75132
**1a**	-699.17279	-0.01584	0.29584	-699.36842
***Int*-1**	-3037.41345	-0.04385	0.66226	-3038.17362
***TS*-1**	-3037.41343	-0.04170	0.66150	-3038.16900
***Int*-2**	-1931.08162	-0.03389	0.46395	-1931.56356
***Int*-3$_{2a\text{-}c}$**	-1931.08744	-0.02778	0.47090	-1931.56472
***TS*-2$_{2a\text{-}c}$**	-1931.08187	-0.02780	0.47234	-1931.55544
***Int*-4$_{2a\text{-}c}$**	-1931.12056	-0.02725	0.47460	-1931.59330
***Int*-3$_{2a\text{-}t}$**	-1931.08498	-0.02791	0.47051	-1931.56245
***TS*-2$_{2a\text{-}t}$**	-1931.07577	-0.02817	0.47133	-1931.55049
***Int*-4$_{2a\text{-}t}$**	-1931.11800	-0.02732	0.47339	-1931.59196
***TS*-3$_{2a\text{-}t}$**	-2753.80334	-0.03283	0.82074	-2754.51061
***Int*-5$_{2a\text{-}c}$**	-2753.80758	-0.03356	0.81927	-2754.51502
***Int*-5$_{2a\text{-}t}$**	-2753.80582	-0.03230	0.82102	-2754.51105
***TS*-4$_{2a\text{-}c}$**	-2753.79320	-0.03251	0.82013	-2754.49800
***TS*-4$_{2a\text{-}t}$**	-2753.79257	-0.03077	0.82445	-2754.49542
***Int*-6$_{2a\text{-}c}$**	-1236.69701	-0.02163	0.45828	-1237.01021
***Int*-6$_{2a\text{-}t}$**	-1236.69558	-0.02135	0.45791	-1237.00885
***TS*-5$_{2a\text{-}c}$**	-1236.69698	-0.01971	0.46174	-1237.00696
***TS*-5$_{2a\text{-}t}$**	-1236.69891	-0.02120	0.46132	-1237.01125
***Int*-7$_{2a\text{-}c}$**	-1236.73764	-0.01875	0.46354	-1237.04682
***Int*-7$_{2a\text{-}t}$**	-1236.74795	-0.01865	0.46642	-1237.05692
***cis*-2a**	-1109.95939	-0.01915	0.46337	-1110.27007
***trans*-2a**	-1109.95886	-0.01911	0.46354	-1110.26955
**Bpin-OPO(OPh)$_2$**	-1517.07343	-0.02120	0.32745	-1517.47739
**BQ**	-381.47116	-0.01117	0.05482	-381.59519
**HO(C$_6$H$_4$)OBpin**	-793.49010	-0.014628	0.23642	-2754.51321
***TS*-2$_{3a\text{-}t}$**	-1931.04113	-0.03581	0.46556	-1931.51950
***Int*-4$_{3a\text{-}t}$**	-1931.06756	-0.03505	0.46566	-1931.54331

Cartesian coordinates

[Pd(OAc)$_2$]$_3$

Pd	5.807132	8.332491	8.573900
Pd	4.613610	5.534456	7.698210
Pd	5.812282	7.495850	5.520707
C	3.182793	7.427844	9.533448
C	1.969968	7.677043	10.402067
H	1.623996	8.703893	10.279062
H	2.260217	7.533438	11.448170
H	1.179785	6.965590	10.160200
C	6.513246	5.752786	9.929867
C	7.349454	5.101637	11.008925
H	7.289169	4.015891	10.941962
H	7.007647	5.444772	11.988102
C	3.144982	6.150440	5.230459
C	1.934141	5.914900	4.355183
H	1.889854	6.663490	3.563485
H	1.025518	5.927578	4.958002
H	2.029576	4.926163	3.894204
C	6.564237	4.656584	5.595273
C	7.379960	3.501022	5.059727
H	8.035361	3.831568	4.254520
H	6.709803	2.711867	4.712376
C	4.862425	10.141021	6.372858
C	4.334692	11.507453	5.995761
H	5.157540	12.087231	5.564469
H	3.970191	12.028815	6.881433
H	3.549355	11.413608	5.245022
C	8.227111	8.588517	6.928707
C	9.686064	8.964656	6.794699
H	10.205043	8.182388	6.234078
H	10.143412	9.093473	7.775022
H	9.764110	9.890185	6.218173
O	6.349972	7.003846	10.034020
O	6.064573	4.992482	9.021043
O	5.589996	4.348985	6.342867
O	6.938033	5.819792	5.259539
O	4.757790	9.236970	5.490523
O	3.032172	5.808739	6.446222
O	4.163445	6.638853	4.660539
O	3.895552	8.440915	9.263182
O	3.386534	6.229614	9.182327
O	5.383578	10.036805	7.520983
O	7.583380	8.472668	5.844812
O	7.786914	8.425379	8.105628
H	8.388126	5.424294	10.889635
H	7.980269	3.090458	5.877574

Pd(OAc)$_2$

Pd	0.000000	0.000065	0.000007
O	1.772473	-1.088433	-0.017239
O	1.772591	1.088490	-0.016886
O	-1.772457	-1.088478	0.017191
O	-1.772604	1.088446	0.016845
C	2.444725	-0.000005	-0.017102
C	-2.444724	-0.000055	0.017033
C	3.938309	-0.000122	0.013016
H	4.272022	-0.003797	1.056155
H	4.321343	-0.897636	-0.475543
H	4.321431	0.900538	-0.469597
C	-3.938311	-0.000155	-0.012939
H	-4.321419	0.897923	0.474516
H	-4.272118	0.002081	-1.056053
H	-4.321268	-0.900259	0.470850

HOAc

O	-4.246632	1.871704	-4.698700
C	-4.107218	3.019299	-5.057096
O	-2.986996	3.736434	-4.787954
C	-5.107709	3.834225	-5.835823
H	-4.660467	4.178113	-6.772924
H	-5.988528	3.227729	-6.041032
H	-5.388786	4.723373	-5.263852
H	-2.403564	3.141513	-4.287836

HOPO(OPh)$_2$

P	2.139283	-0.084568	0.093165
O	3.493133	0.083873	-0.743068
H	4.224138	-0.325295	-0.258896
O	2.279476	-0.641162	1.456252
O	1.563743	1.423208	-0.026545
O	1.241086	-0.944661	-0.943516
C	0.200565	1.678048	0.169343
C	-0.456064	2.404640	-0.818943
C	-0.462621	1.220523	1.306384
C	-1.819673	2.660636	-0.674243
C	-1.829005	1.470992	1.428787
H	0.081062	0.660939	2.058643
C	-2.510337	2.186243	0.442500
H	-2.360494	1.101399	2.299870
H	-3.573899	2.376380	0.545411
C	-0.033280	-1.422009	-0.600260
C	-1.097069	-1.034750	-1.409234
C	-0.212233	-2.265457	0.493298
C	-2.378379	-1.489388	-1.102745
C	-1.501528	-2.711396	0.787575
H	0.637502	-2.534861	1.108007
C	-2.584739	-2.325094	-0.003131
H	-1.655690	-3.363160	1.642061
H	-3.584640	-2.673600	0.235361
H	-2.342692	3.223131	-1.441496
H	0.101658	2.743711	-1.684843
H	-3.216056	-1.181822	-1.720695
H	-0.913453	-0.366290	-2.241868

(AcO)PdL

Pd	-0.005773	1.343771	-0.096967
O	-0.016995	3.208463	0.819429
C	-0.026382	3.775879	-0.327985
O	-0.015530	3.006167	-1.349039
C	-0.079117	5.262725	-0.466296
H	0.428078	5.570214	-1.382498
H	-1.126608	5.576291	-0.529227
H	0.373292	5.735720	0.406856
P	0.010804	-1.288105	0.153658
O	0.003591	-0.236354	1.288477
O	0.005546	-0.469262	-1.154099
O	1.216177	-2.335972	0.259265
O	-1.180719	-2.351816	0.258950
C	2.544710	-1.880717	0.161097
C	3.312206	-2.370410	-0.889378
C	3.064008	-1.006946	1.112946
C	4.645434	-1.971898	-0.988428
C	4.397733	-0.612600	0.995213
H	2.431394	-0.644091	1.913998
C	5.188961	-1.092229	-0.050004
H	4.816414	0.070341	1.727691
H	6.225771	-0.782328	-0.133029
C	-2.515084	-1.913950	0.161747
C	-3.276127	-2.411288	-0.889843
C	-3.045889	-1.049397	1.115678
C	-4.614550	-2.030319	-0.987870
C	-4.384775	-0.672606	0.999014
H	-2.418043	-0.680094	1.917532
C	-5.169663	-1.160231	-0.047296
H	-4.812498	0.002807	1.733258
H	-6.210556	-0.864075	-0.129442
H	5.256083	-2.346467	-1.803844
H	2.862124	-3.046726	-1.607153
H	-5.220244	-2.411068	-1.804118
H	-2.817162	-3.079870	-1.609237

PdL$_2$

Pd	0.000001	-0.000084	0.001456
O	-0.038341	-1.695375	1.233012
O	-0.054427	-1.694689	-1.230075
P	0.065043	2.627624	0.001187

O	0.038250	1.695285	1.232924
O	0.054336	1.694485	-1.230158
O	-1.129341	3.696063	0.014121
O	1.299857	3.648559	-0.012191
C	-2.439961	3.207004	0.203236
C	-2.990638	2.292233	-0.691766
C	-3.148364	3.684061	1.299766
C	-4.288592	1.833483	-0.461941
C	-4.449993	3.226305	1.507797
H	-2.676772	4.392202	1.971570
C	-5.019248	2.297929	0.633140
H	-5.014213	3.590668	2.360475
H	-6.030170	1.940797	0.803583
C	2.591138	3.111553	-0.203502
C	3.319395	3.572574	-1.293874
C	3.105182	2.168189	0.683524
C	4.603481	3.068585	-1.503906
C	4.385739	1.663807	0.451783
H	2.507675	1.830219	1.521575
C	5.135788	2.111249	-0.637243
H	4.788810	0.913618	1.124012
H	6.133092	1.718383	-0.809195
H	-4.720715	1.105135	-1.140131
H	-2.407670	1.940449	-1.534342
H	5.182841	3.419992	-2.351856
H	2.876172	4.304541	-1.959437
P	-0.065105	-2.627771	0.001304
O	1.129435	-3.696056	0.014249
O	-1.299792	-3.648837	-0.011954
C	2.439972	-3.206814	0.203336
C	3.148285	-3.683245	1.300185
C	2.990618	-2.292368	-0.692019
C	4.449852	-3.225248	1.508119
H	2.676705	-4.391095	1.972305
C	4.288491	-1.833366	-0.462296
H	2.407653	-1.941053	-1.534794
C	5.019091	-2.297230	0.633077
H	5.014024	-3.589139	2.361029
H	4.720607	-1.105269	-1.140759
H	6.029960	-1.939914	0.803452
C	-2.591089	-3.111830	-0.203405
C	-3.105281	-2.168668	0.683742
C	-3.319122	-3.572592	-1.294031
C	-4.385792	-1.664216	0.451878
H	-2.507911	-1.830879	1.521963
C	-4.603161	-3.068537	-1.504188
H	-2.875775	-4.304397	-1.959689
C	-5.135627	-2.111398	-0.637402
H	-4.788981	-0.914176	1.124206
H	-5.182367	-3.419729	-2.352333
H	-6.132899	-1.718501	-0.809464

1a

C	-0.308949	-0.919371	1.084658
C	3.545527	2.533115	0.237452
C	3.440968	1.062720	-0.051081
C	2.005819	-1.047516	0.199031
C	0.733342	-1.657874	0.799689
H	3.555513	0.883356	-1.125592
H	3.504089	2.815844	1.290580
H	2.863586	-1.541941	0.678248
H	4.267485	0.530427	0.443186
C	-1.345733	-0.147415	1.341382
C	-1.504479	0.471680	2.715760
H	-0.683894	0.169146	3.368240
H	-1.510660	1.566226	2.655437
C	0.741182	-3.158486	0.990982
H	1.498532	-3.447011	1.730836
H	-0.230122	-3.524518	1.328953
H	1.000829	-3.657842	0.050902
C	2.040943	-1.372164	-1.275339
H	1.278130	-0.869036	-1.871125
C	2.893668	-2.212330	-1.861311
H	3.663959	-2.726694	-1.291635
H	2.857513	-2.414205	-2.927621
C	3.657843	3.487575	-0.685776
H	3.716931	4.538830	-0.420021
H	3.696987	3.248531	-1.746097
H	-2.447384	0.163802	3.182520
C	-2.366427	0.139574	0.299077
C	-3.458888	0.978552	0.566896
C	-2.261372	-0.418357	-0.988345
C	-4.410750	1.252875	-0.416788
H	-3.572072	1.425369	1.548125
C	-3.209087	-0.145236	-1.967067
H	-1.423966	-1.073168	-1.208359
C	-4.292128	0.694369	-1.687437
H	-5.246660	1.906385	-0.184480
H	-3.105448	-0.588574	-2.953329
H	-5.032386	0.907850	-2.452476
C	2.112114	0.467006	0.439906
H	1.989740	0.661248	1.512107
H	1.277739	0.973673	-0.058538

***Int*-1**

Pd	0.637935	0.622812	-0.108708
O	-2.377824	-0.899153	2.800822
O	-0.400851	0.365236	1.678631
O	0.865893	-2.433983	-2.117428
O	1.294960	-1.268069	0.195142
C	0.049962	2.667030	-0.570693
C	-3.796659	-0.514688	-3.376417
C	-2.360236	-0.092273	-3.510747
C	-0.593021	1.607893	-2.669416
C	-0.415850	2.836539	-1.796445
H	-4.089538	-0.890728	-2.398466
H	-0.444805	1.892875	-3.723619
H	-2.157818	0.225842	-4.541781
C	0.604960	3.056427	0.622497
C	-0.219290	3.273598	1.858887
H	-1.240398	3.539993	1.592569
H	-0.268116	2.352681	2.450416
C	-0.790447	4.190181	-2.342613
H	-1.839152	4.194080	-2.661476
H	-0.657811	4.976717	-1.596967
H	-0.185879	4.432385	-3.225006
C	0.472526	0.578454	-2.336723
H	0.244687	-0.472444	-2.513726
C	1.785762	0.873221	-2.004273
H	2.163588	1.887698	-2.003829
H	2.524126	0.082235	-1.990999
C	-4.702102	-0.440912	-4.351126
H	-5.729451	-0.757024	-4.197198
H	-4.446578	-0.074154	-5.343114
H	0.233204	4.065037	2.460609
P	1.414793	-2.499780	-0.734431
O	0.835411	-3.776420	0.067352
O	3.029593	-2.862597	-0.708755
C	0.964065	-3.849302	1.461658
C	-0.126667	-3.498804	2.249913
C	2.162118	-4.283609	2.022621
C	-0.008548	-3.565273	3.637305
H	-1.041579	-3.163605	1.783963
C	2.265049	-4.356320	3.413171
H	2.989990	-4.544740	1.373905
C	1.185370	-3.993836	4.221692
H	-0.851261	-3.261219	4.249256
H	3.194170	-4.694269	3.862908
H	1.275438	-4.043984	5.302647
C	3.907630	-1.821462	-0.972642
C	4.241176	-0.921227	0.041980
C	4.424924	-1.678941	-2.259929
C	5.086834	0.147674	-0.249082
H	3.802467	-1.052542	1.023555
C	5.280205	-0.609246	-2.537125
H	4.135123	-2.392271	-3.023227
C	5.604111	0.310424	-1.537655
H	5.330492	0.863042	0.529143
H	5.682651	-0.491421	-3.538750
H	6.259447	1.147471	-1.758677

C	-2.025947	1.045294	-2.532914
C	2.068132	3.277833	0.704090
C	2.759554	2.976734	1.890794
C	2.780530	3.794978	-0.391421
C	4.134843	3.174724	1.969725
H	2.220806	2.559771	2.734600
C	4.152086	4.007535	-0.302671
H	2.243525	4.045374	-1.300206
C	4.833221	3.692969	0.876126
H	4.662717	2.923246	2.883974
H	4.690870	4.418789	-1.150211
H	5.904916	3.852496	0.942563
H	-1.715149	-0.954884	-3.303471
H	-2.175625	0.709654	-1.503345
H	-2.732308	1.866950	-2.701425
P	-1.785720	-0.320521	1.570138
O	-1.653706	-1.335487	0.302376
O	-2.801842	0.752560	0.811839
C	-2.682224	-2.102770	-0.237983
C	-2.319096	-2.916417	-1.311664
C	-3.993384	-2.070521	0.238889
C	-3.295666	-3.696205	-1.928436
H	-1.290554	-2.920761	-1.654316
C	-4.956473	-2.859785	-0.394155
H	-4.254904	-1.456843	1.090914
C	-4.617244	-3.669579	-1.478076
H	-3.016746	-4.323897	-2.769000
H	-5.978676	-2.837262	-0.028460
H	-5.374287	-4.276031	-1.965062
C	-3.266844	1.915014	1.385015
C	-3.564226	2.959527	0.505209
C	-3.438903	2.069996	2.763281
C	-4.012472	4.178829	1.010806
H	-3.427553	2.800340	-0.557790
C	-3.878718	3.300771	3.253249
H	-3.225628	1.238929	3.425451
C	-4.162068	4.359268	2.388151
H	-4.241045	4.990183	0.325841
H	-4.004593	3.427259	4.324459
H	-4.504782	5.311063	2.781312

***TS*-1**

Pd	0.162859	0.883119	0.373673
O	-0.679915	-3.043620	0.132149
O	-0.730820	-0.618120	-0.890397
O	2.454584	0.631667	3.206866
O	1.513614	-0.553789	1.076328
C	-0.849764	2.345376	-0.632939
C	4.696128	3.674332	-1.633276
C	3.613813	3.974629	-0.635238
C	1.111773	3.684575	-0.078839
C	-0.248846	3.535825	-0.729246
H	4.926720	2.619529	-1.774840
H	1.207662	4.690917	0.360409
H	3.587256	5.051196	-0.425122
C	-2.068000	1.805226	-1.179257
C	-1.973620	1.084062	-2.383675
H	-1.142029	1.342114	-3.036906
H	-1.426206	0.071601	-1.745420
C	-0.825374	4.710274	-1.475513
H	-0.118442	5.090697	-2.221598
H	-1.753792	4.446793	-1.987904
H	-1.036248	5.540102	-0.788714
C	1.263202	2.662720	1.037867
H	2.257315	2.286950	1.267033
C	0.275279	2.383621	1.972168
H	-0.676242	2.902666	1.970259
H	0.541618	1.817400	2.856214
C	5.351087	4.587038	-2.350380
H	6.116658	4.309743	-3.068762
H	5.149560	5.649960	-2.237278
H	-2.879879	0.740402	-2.870785
P	2.028928	-0.624199	2.530376
O	3.211152	-1.738181	2.561650
O	0.873504	-1.431997	3.400027
C	3.382347	-2.700317	1.574985
C	2.325980	-3.486912	1.119671
C	4.671789	-2.857471	1.066843
C	2.569155	-4.427045	0.119420
H	1.324325	-3.339348	1.500372
C	4.903528	-3.813593	0.076631
H	5.467090	-2.227052	1.449701
C	3.852145	-4.596537	-0.404514
H	1.737942	-5.013457	-0.257442
H	5.905470	-3.937096	-0.323851
H	4.030892	-5.328975	-1.185590
C	-0.341749	-0.773790	3.537132
C	-1.344924	-0.986322	2.588431
C	-0.530292	0.106311	4.602923
C	-2.549499	-0.290033	2.707329
H	-1.166789	-1.689706	1.783569
C	-1.743049	0.789448	4.714582
H	0.277792	0.255202	5.309850
C	-2.750156	0.600395	3.765028
H	-3.328634	-0.441697	1.967194
H	-1.896641	1.474716	5.543293
H	-3.689142	1.137274	3.850284
C	2.231078	3.516335	-1.131057
C	-3.268156	1.756095	-0.335543
C	-4.266991	0.785799	-0.539775
C	-3.425545	2.691509	0.702650
C	-5.390225	0.756013	0.278216
H	-4.151723	0.030785	-1.306218
C	-4.563851	2.675342	1.500589
H	-2.652145	3.433688	0.862485
C	-5.545732	1.703387	1.294563
H	-6.139025	-0.013019	0.122692
H	-4.679904	3.409224	2.291433
H	-6.426658	1.679295	1.928668
H	3.849072	3.472725	0.312597
H	2.263265	2.463166	-1.429698
H	1.979931	4.087146	-2.031578
P	-0.495176	-2.176231	-1.051128
O	0.912804	-2.384602	-1.794878
O	-1.513874	-2.526147	-2.295706
C	1.650319	-1.329929	-2.335754
C	2.871118	-1.023768	-1.744114
C	1.178073	-0.641440	-3.450768
C	3.633278	0.009536	-2.288825
H	3.191550	-1.576018	-0.871247
C	1.947055	0.398336	-3.976670
H	0.230558	-0.928300	-3.893899
C	3.175000	0.726222	-3.397554
H	4.589368	0.253997	-1.836031
H	1.591031	0.943162	-4.846023
H	3.768267	1.539203	-3.803644
C	-2.891585	-2.439502	-2.152411
C	-3.601050	-1.978323	-3.263544
C	-3.557253	-2.809339	-0.980069
C	-4.990635	-1.875860	-3.199441
H	-3.053089	-1.711757	-4.160917
C	-4.948081	-2.698565	-0.931237
H	-2.989137	-3.179179	-0.135250
C	-5.670130	-2.232216	-2.031427
H	-5.541032	-1.518685	-4.064409
H	-5.466828	-2.987117	-0.022130
H	-6.751779	-2.155622	-1.984574

***Int*-2**

Pd	0.870417	-0.758946	-0.101616
O	-2.352623	0.023834	-1.550482
O	-0.983575	-1.822192	-0.297595
C	2.649217	-0.037525	0.399657
C	0.988944	5.705547	-0.540972
C	1.225603	4.359924	-1.164631
C	2.253442	2.025544	-0.821466
C	3.119472	1.165419	0.082703
H	0.420600	5.699203	0.389657
H	2.783779	2.132405	-1.781749
H	1.741248	4.477104	-2.125896

C	2.849589	-1.349255	0.907955
C	1.776566	-1.786496	1.707534
H	1.601069	-2.842478	1.877885
C	4.438833	1.684652	0.590614
H	4.297624	2.482992	1.327481
H	5.018675	0.886140	1.058861
H	5.027841	2.106593	-0.232552
C	0.900677	1.357866	-1.099156
H	0.047471	1.753841	-0.551815
C	0.659385	0.469555	-2.111098
H	1.462615	0.154208	-2.773401
H	-0.361874	0.179179	-2.345985
C	1.430312	6.865229	-1.026600
H	1.232871	7.808299	-0.526020
H	2.001886	6.912958	-1.950695
H	1.279320	-1.088804	2.373640
P	-2.252626	-0.985243	-0.450428
O	-2.568613	-0.267098	1.022187
O	-3.449782	-2.087475	-0.415947
C	-1.950384	0.915803	1.349773
C	-2.249775	2.103160	0.671815
C	-1.039003	0.915568	2.409582
C	-1.633831	3.289764	1.069936
H	-2.942663	2.068077	-0.159175
C	-0.428619	2.108846	2.799408
H	-0.844569	-0.019843	2.922850
C	-0.724674	3.299482	2.132713
H	-1.869698	4.213687	0.549813
H	0.272345	2.107920	3.628964
H	-0.259724	4.229425	2.445462
C	-4.795745	-1.748375	-0.407040
C	-5.616966	-2.490643	0.441333
C	-5.325873	-0.755909	-1.234261
C	-6.987217	-2.235508	0.465972
H	-5.167295	-3.253421	1.067485
C	-6.698348	-0.506834	-1.191448
H	-4.663784	-0.197453	-1.884597
C	-7.533521	-1.240888	-0.347759
H	-7.627136	-2.813418	1.126376
H	-7.115938	0.266002	-1.830470
H	-8.600453	-1.041215	-0.325144
C	2.042931	3.439564	-0.243157
C	3.856663	-2.267639	0.325962
C	4.189542	-3.458479	0.988289
C	4.520092	-1.943351	-0.867567
C	5.155050	-4.312935	0.460500
H	3.708474	-3.703948	1.929149
C	5.482204	-2.799339	-1.394138
H	4.261719	-1.024125	-1.381953
C	5.801251	-3.987949	-0.732974
H	5.406424	-5.229595	0.984645
H	5.981195	-2.542492	-2.323356
H	6.552598	-4.654935	-1.144066
H	0.258288	3.887331	-1.379501
H	1.535093	3.343846	0.723125
H	3.014074	3.907357	-0.052452

***Int*-3$_{2a\text{-}c}$**

Pd	0.589576	0.882916	-0.940473
O	-0.388675	-0.704178	0.292099
O	0.027407	-0.963406	-2.199579
C	0.657731	2.237355	0.536088
C	2.390831	2.218880	-1.440552
C	3.588838	1.509559	-0.848825
C	2.702312	1.179536	1.631051
C	1.542230	2.174072	1.535411
H	2.171409	3.201716	-1.040443
H	3.517747	1.711787	2.141349
H	4.318147	2.268817	-0.540871
C	-0.415291	3.212960	0.291692
C	-0.130473	4.525501	0.167766
H	-0.910016	5.261329	-0.002104
C	1.410159	3.049749	2.764412
H	2.258425	3.743463	2.835269
H	0.490693	3.634916	2.752930
H	1.427546	2.428911	3.666834
C	2.250858	0.042169	2.524841
H	1.425998	-0.540567	2.117838
C	2.773718	-0.249321	3.715180
H	3.595831	0.326456	4.134521
H	2.401865	-1.075684	4.314147
C	1.693928	1.842953	-2.572737
H	0.995563	2.531229	-3.039341
H	1.982741	0.970893	-3.154845
H	0.886637	4.890612	0.263181
P	-0.484115	-1.623760	-0.929144
O	0.293277	-3.029970	-0.620091
O	-1.975855	-2.199635	-1.167303
C	1.672191	-2.960529	-0.416032
C	2.164488	-3.129795	0.875274
C	2.528575	-2.739366	-1.495226
C	3.542385	-3.084802	1.090541
H	1.467210	-3.284310	1.690784
C	3.904471	-2.697801	-1.267343
H	2.106574	-2.593224	-2.482076
C	4.414400	-2.870817	0.021889
H	3.929462	-3.200411	2.097563
H	4.578975	-2.531297	-2.102003
H	5.485876	-2.836544	0.192729
C	-2.976545	-2.236099	-0.205265
C	-4.272963	-1.997304	-0.655757
C	-2.719436	-2.527272	1.133721
C	-5.330194	-2.042338	0.251597
H	-4.429202	-1.769498	-1.703961
C	-3.785623	-2.558603	2.033235
H	-1.705300	-2.711599	1.465141
C	-5.090964	-2.316692	1.600020
H	-6.341443	-1.855420	-0.096724
H	-3.590791	-2.776966	3.078793
H	-5.914356	-2.345017	2.306498
C	3.281598	0.569944	0.336996
C	-1.796855	2.682520	0.129906
C	-2.689377	3.240161	-0.798440
C	-2.223836	1.598035	0.911580
C	-3.979058	2.730118	-0.935008
H	-2.358140	4.057458	-1.431920
C	-3.513484	1.090466	0.776909
H	-1.529769	1.136963	1.603337
C	-4.393959	1.653922	-0.147260
H	-4.654922	3.163386	-1.666594
H	-3.824381	0.244468	1.379348
H	-5.392176	1.243050	-0.263238
H	4.063316	0.901294	-1.627206
H	4.211767	0.066100	0.618492
H	2.622769	-0.225503	-0.023479

***TS*-2$_{2a\text{-}c}$**

Pd	0.289012	0.522640	-0.694582
O	-0.218626	-0.941730	1.105411
O	-0.422097	-1.449065	-1.372051
C	0.772010	2.347939	0.217702
C	1.760836	2.269758	-1.621972
C	3.205212	2.047846	-1.227767
C	2.916336	1.443131	1.228015
C	1.673340	2.321643	1.224571
H	1.438315	3.300778	-1.727107
H	3.721611	2.068287	1.641620
H	3.627243	2.990212	-0.860947
C	-0.354815	3.309622	0.097172
C	-0.108861	4.628730	-0.007654
H	-0.912583	5.354101	-0.080197
C	1.505413	3.138254	2.482768
H	2.292253	3.901128	2.551251
H	0.538359	3.637126	2.529223
H	1.620258	2.486127	3.354979
C	2.708683	0.277398	2.170926
H	1.883915	-0.382743	1.910195
C	3.445984	0.054601	3.258168
H	4.266741	0.710743	3.540164
H	3.258642	-0.797831	3.904531

C	1.068627	1.311148	-2.424460
H	0.238073	1.656629	-3.037525
H	1.631205	0.473207	-2.834511
H	0.906750	5.012308	0.001954
P	-0.381627	-2.025419	0.050308
O	0.761873	-3.183682	0.171001
O	-1.627895	-3.018792	0.347156
C	2.068843	-2.870236	-0.192116
C	3.042783	-2.851036	0.802951
C	2.395063	-2.620054	-1.527177
C	4.367388	-2.580735	0.457404
H	2.750376	-3.039228	1.829412
C	3.722948	-2.349985	-1.859090
H	1.609770	-2.629311	-2.272492
C	4.711533	-2.330532	-0.871746
H	5.125600	-2.554233	1.233446
H	3.984900	-2.159252	-2.895759
H	5.743227	-2.121764	-1.137515
C	-2.935674	-2.540213	0.314838
C	-3.481184	-2.032208	-0.863679
C	-3.692464	-2.645227	1.479736
C	-4.817358	-1.632703	-0.867726
H	-2.856907	-1.948058	-1.744737
C	-5.028511	-2.242375	1.460361
H	-3.228196	-3.041832	2.375651
C	-5.594836	-1.738062	0.287236
H	-5.249128	-1.232902	-1.779963
H	-5.625539	-2.324449	2.363503
H	-6.635573	-1.429242	0.274435
C	3.373871	0.958657	-0.157236
C	-1.735761	2.762490	0.042744
C	-2.702695	3.327992	-0.802360
C	-2.093336	1.657781	0.835846
C	-3.995692	2.809893	-0.847672
H	-2.427571	4.160017	-1.443361
C	-3.384651	1.136737	0.783146
H	-1.361143	1.196672	1.488536
C	-4.339110	1.712453	-0.055949
H	-4.730443	3.253892	-1.513156
H	-3.639034	0.270854	1.384066
H	-5.338624	1.293302	-0.103000
H	3.766190	1.766585	-2.127285
H	4.421402	0.654444	-0.083280
H	2.820195	0.064215	-0.459968

***Int*-4$_{2a\text{-}c}$**

Pd	0.288074	0.532699	-0.344895
O	0.024117	-1.167832	1.241114
O	-0.290342	-1.368796	-1.259527
C	0.536210	2.675454	0.144765
C	0.908187	3.004255	-1.320404
C	2.411743	3.240625	-1.477873
C	2.925544	1.872189	0.597134
C	1.445497	1.999536	0.958185
H	0.354699	3.890766	-1.646257
H	3.403362	2.695272	1.154690
H	2.683705	4.174702	-0.968617
C	-0.718640	3.316231	0.662003
C	-0.622412	4.496634	1.287093
H	-1.507222	5.018691	1.637424
C	1.135349	1.703724	2.405324
H	1.829568	2.252959	3.052641
H	0.116955	1.986017	2.670046
H	1.271063	0.638696	2.607507
C	3.525680	0.580589	1.089474
H	3.084474	-0.326928	0.684903
C	4.544047	0.502587	1.943292
H	5.002867	1.392072	2.369868
H	4.956003	-0.455642	2.241580
C	0.415408	1.728673	-1.980612
H	-0.607847	1.772968	-2.358094
H	1.095471	1.250261	-2.688511
H	0.339841	4.966190	1.469970
P	-0.179348	-2.124505	0.075513
O	0.977508	-3.268127	0.009382
O	-1.401781	-3.164936	0.287536
C	2.296975	-2.882912	-0.213802
C	3.240172	-3.194528	0.763121
C	2.667105	-2.246602	-1.400811
C	4.579808	-2.870681	0.543927
H	2.912539	-3.685033	1.672741
C	4.008921	-1.918531	-1.600281
H	1.906895	-2.014169	-2.136057
C	4.967115	-2.227954	-0.633522
H	5.319593	-3.116043	1.299857
H	4.303971	-1.420916	-2.519228
H	6.008190	-1.967632	-0.794559
C	-2.732470	-2.774974	0.155364
C	-3.224192	-2.297291	-1.059399
C	-3.566964	-2.952109	1.257040
C	-4.583162	-2.001022	-1.164226
H	-2.543846	-2.153743	-1.889167
C	-4.925331	-2.654447	1.136006
H	-3.144470	-3.324540	2.183497
C	-5.437317	-2.180757	-0.074238
H	-4.973057	-1.625254	-2.105085
H	-5.581450	-2.793338	1.989731
H	-6.494588	-1.952306	-0.165868
C	3.224641	2.082649	-0.895698
C	-2.038872	2.691513	0.372681
C	-2.984118	3.371743	-0.404916
C	-2.356120	1.414685	0.866809
C	-4.216895	2.783993	-0.697681
H	-2.739558	4.356869	-0.790720
C	-3.589950	0.835191	0.583393
H	-1.648074	0.871647	1.483865
C	-4.519834	1.514404	-0.206626
H	-4.935509	3.318650	-1.312069
H	-3.821911	-0.148212	0.973813
H	-5.472685	1.048513	-0.437289
H	2.645282	3.374427	-2.540584
H	4.297729	2.263639	-1.013359
H	3.004051	1.155178	-1.435392

***Int*-3$_{2a\text{-}t}$**

Pd	-0.377358	-0.405336	0.801163
O	0.456758	1.648829	1.380460
O	0.336429	0.754190	-0.976161
C	-0.829807	-2.082090	-0.193664
C	-2.087584	-1.196425	2.023710
C	-3.229521	-0.335095	1.552165
C	-3.242513	-1.447629	-0.842607
C	-1.951177	-2.273287	-0.901651
H	-2.184657	-2.256504	1.813982
C	0.288334	-3.022661	0.002987
C	0.039025	-4.228919	0.555281
H	0.826816	-4.959159	0.709399
C	-2.037777	-3.413447	-1.901228
H	-2.347898	-3.022882	-2.878630
H	-1.083152	-3.925514	-2.016336
H	-2.794736	-4.148105	-1.605771
C	-4.388099	-2.358643	-0.446684
C	-5.503471	-2.535135	-1.153021
H	-5.654192	-2.034985	-2.106920
H	-6.304901	-3.181464	-0.807955
C	-1.042929	-0.820760	2.849013
H	-0.389252	-1.574424	3.277059
H	-0.984272	0.179048	3.271701
H	-0.968156	-4.515531	0.837684
P	0.680072	1.961143	-0.099946
O	-0.136783	3.286365	-0.573694
O	2.142085	2.570994	-0.419114
C	-1.526982	3.258701	-0.484796
C	-2.266521	3.184994	-1.662912
C	-2.150858	3.339225	0.760417
C	-3.659952	3.207511	-1.592499
H	-1.742900	3.112671	-2.609341
C	-3.545060	3.358752	0.816739
H	-1.540657	3.379334	1.654526
C	-4.302066	3.294417	-0.355372

H	-4.243017	3.154112	-2.506768
H	-4.039748	3.428904	1.780958
H	-5.386246	3.313860	-0.304930
C	3.335638	1.913036	-0.133046
C	4.365567	2.082027	-1.056389
C	3.526096	1.182238	1.040005
C	5.614858	1.521513	-0.793934
H	4.175857	2.658307	-1.955107
C	4.778434	0.617536	1.280530
H	2.711043	1.074198	1.744641
C	5.826318	0.788787	0.375436
H	6.422311	1.658868	-1.506899
H	4.932394	0.042062	2.188054
H	6.799110	0.351316	0.577175
C	-3.277961	-0.164282	0.017259
C	1.664594	-2.593535	-0.360855
C	2.782848	-3.036720	0.364645
C	1.866851	-1.743732	-1.458007
C	4.068036	-2.659930	-0.013872
H	2.637228	-3.661743	1.240378
C	3.153919	-1.359804	-1.829212
H	1.010523	-1.364916	-2.001267
C	4.258013	-1.823886	-1.116394
H	4.922541	-3.003188	0.561976
H	3.291465	-0.686328	-2.668806
H	5.257332	-1.509992	-1.396000
H	-4.208110	0.357265	-0.225605
H	-2.465411	0.497796	-0.294257
H	-4.269764	-2.886437	0.500305
H	-3.440456	-1.118274	-1.872671
H	-4.167065	-0.789415	1.898927
H	-3.160068	0.659861	2.001982

***TS*-2**$_{2a\text{-}t}$

Pd	-0.192172	0.429121	-0.777055
O	0.569374	-1.566314	-1.358934
O	0.256245	-0.926744	1.075153
C	-0.846739	2.164688	0.171650
C	-1.609875	2.145336	-1.811698
C	-3.072680	1.854402	-1.567673
C	-2.991553	0.992124	0.849099
C	-1.843800	1.978519	1.064972
H	-1.311054	3.188383	-1.814376
C	0.192100	3.221605	0.225980
C	-0.167741	4.519265	0.236680
H	0.570324	5.313022	0.289299
C	-1.826363	2.655197	2.415534
H	-1.936774	1.898355	3.200484
H	-0.902257	3.207842	2.579199
H	-2.671879	3.342304	2.532391
C	-4.263987	1.475901	1.506237
C	-4.875010	0.859630	2.516498
H	-4.457148	-0.038842	2.963856
H	-5.807411	1.227306	2.934078
C	-0.801632	1.250994	-2.570699
H	0.080164	1.649074	-3.069208
H	-1.277319	0.408778	-3.071685
H	-1.213239	4.810911	0.217521
P	0.548806	-2.054152	0.095855
O	-0.488506	-3.299676	0.279503
O	1.871098	-2.910753	0.479035
C	-1.833589	-3.050571	0.023794
C	-2.687049	-2.778127	1.091445
C	-2.308430	-3.109918	-1.286272
C	-4.046313	-2.581799	0.841856
H	-2.275266	-2.726871	2.092972
C	-3.669694	-2.914000	-1.522605
H	-1.609131	-3.304946	-2.090615
C	-4.540443	-2.651472	-0.462358
H	-4.718461	-2.367394	1.666686
H	-4.049028	-2.966196	-2.538753
H	-5.598720	-2.500400	-0.651022
C	3.143664	-2.350934	0.406562
C	3.938272	-2.424117	1.549012
C	3.626627	-1.797472	-0.779425
C	5.246231	-1.940112	1.500648
H	3.523997	-2.860617	2.450940
C	4.934603	-1.314216	-0.810828
H	2.977403	-1.744222	-1.644622
C	5.748446	-1.385241	0.321240
H	5.871028	-1.997490	2.386775
H	5.315829	-0.876476	-1.728180
H	6.767006	-1.011243	0.285827
C	-3.289135	0.655128	-0.631539
C	1.619984	2.810286	0.194921
C	2.555868	3.519477	-0.572867
C	2.054172	1.696378	0.933888
C	3.894300	3.130104	-0.597325
H	2.222869	4.362941	-1.170191
C	3.391940	1.308237	0.906293
H	1.345888	1.125177	1.522383
C	4.314802	2.022873	0.141585
H	4.605207	3.683569	-1.204137
H	3.709503	0.437415	1.468459
H	5.351828	1.704612	0.111433
H	-4.322497	0.306586	-0.700518
H	-2.665054	-0.179825	-0.963014
H	-4.708918	2.377506	1.081959
H	-2.695197	0.060863	1.348400
H	-3.553048	2.745840	-1.150017
H	-3.548387	1.651670	-2.535305

***Int*-4**$_{2a\text{-}t}$

Pd	-0.290979	0.323352	-0.442401
O	0.761164	-1.377428	-1.336200
O	0.374092	-1.241224	1.164373
C	-1.154688	2.267914	0.131155
C	-1.429716	2.624779	-1.350121
C	-2.915750	2.491628	-1.689331
C	-3.261665	0.799367	0.183240
C	-1.935443	1.296516	0.761933
H	-1.088373	3.645624	-1.550956
C	-0.162244	3.128038	0.851113
C	-0.593210	4.070667	1.697055
H	0.103440	4.739097	2.193231
C	-1.713232	0.921135	2.207557
H	-1.802097	-0.162310	2.321755
H	-0.730763	1.226259	2.563812
H	-2.475709	1.389140	2.838678
C	-4.398252	1.400979	0.983925
C	-5.262055	0.688638	1.705830
H	-5.176300	-0.392653	1.776870
H	-6.080976	1.155616	2.244800
C	-0.564106	1.571505	-2.021692
H	0.449183	1.898490	-2.263175
H	-1.021594	1.010957	-2.838971
H	-1.649299	4.194303	1.918525
P	0.771519	-2.136369	0.000403
O	-0.183030	-3.457709	-0.068822
O	2.164263	-2.930684	0.225712
C	-1.554159	-3.206580	-0.126985
C	-2.315216	-3.354711	1.030319
C	-2.137086	-2.817368	-1.333332
C	-3.691443	-3.129028	0.971850
H	-1.821783	-3.640895	1.951944
C	-3.513654	-2.592127	-1.378630
H	-1.507481	-2.689564	-2.205790
C	-4.293652	-2.747488	-0.230052
H	-4.292082	-3.249281	1.868446
H	-3.975953	-2.291885	-2.313928
H	-5.363670	-2.569792	-0.270662
C	3.373530	-2.234221	0.177985
C	4.134361	-2.170717	1.342914
C	3.823532	-1.673021	-1.016709
C	5.380206	-1.541955	1.306660
H	3.745329	-2.615083	2.252105
C	5.068740	-1.044468	-1.037351
H	3.194505	-1.722264	-1.896571
C	5.851033	-0.979503	0.117806
H	5.980246	-1.491044	2.209959

H	5.425682	-0.602602	-1.962484
H	6.820450	-0.491667	0.093247
C	-3.442575	1.103680	-1.315827
C	1.282295	2.961699	0.515959
C	1.958958	3.927345	-0.236829
C	1.969401	1.803888	0.918283
C	3.293664	3.731080	-0.602467
H	1.430848	4.824613	-0.545797
C	3.302095	1.614208	0.561762
H	1.466030	1.046677	1.510405
C	3.964000	2.572928	-0.208843
H	3.804822	4.483084	-1.196575
H	3.818755	0.716152	0.878552
H	4.998361	2.413030	-0.497915
H	-4.503224	1.013220	-1.570426
H	-2.913585	0.338340	-1.891358
H	-4.499906	2.485608	0.932448
H	-3.299355	-0.284085	0.327576
H	-3.479713	3.267482	-1.156052
H	-3.057453	2.674277	-2.760839

***TS*-2**$_{\text{3a-}t}$

Pd	0.286094	-0.161318	-1.418417
O	-2.514029	1.897130	-0.267677
O	-1.767672	-0.117305	-1.775469
C	2.454291	-0.389802	-1.324594
C	5.048387	3.926626	1.490667
C	3.916921	3.434356	0.633522
C	3.158560	1.686194	-1.066621
C	3.444129	0.400547	-1.796338
H	5.430101	3.217931	2.226966
H	2.764120	2.485404	-1.707190
H	3.558180	4.244357	-0.012513
C	2.280645	-1.857231	-1.351895
C	1.903049	-2.464978	-2.501803
H	1.808030	-3.543908	-2.564416
C	4.642113	0.043044	-2.606572
H	5.567863	0.288737	-2.073000
H	4.647253	-1.020349	-2.855926
H	4.654016	0.617422	-3.541055
C	2.060599	1.034786	-0.205641
H	2.380878	0.670810	0.769614
C	0.685601	1.492608	-0.287083
H	0.468733	2.347379	-0.925968
H	0.057054	1.424284	0.594738
C	5.608569	5.131761	1.395496
H	6.431534	5.432616	2.036514
H	5.256857	5.866072	0.674556
H	1.724362	-1.891618	-3.404948
P	-2.692518	0.481791	-0.697948
O	-2.665146	-0.548214	0.613036
O	-4.172400	0.090740	-1.253186
C	-1.648612	-0.406955	1.533970
C	-1.623045	0.685907	2.405236
C	-0.638602	-1.370995	1.572175
C	-0.563735	0.817171	3.303499
H	-2.408363	1.427869	2.335304
C	0.413658	-1.234353	2.479775
H	-0.688996	-2.211328	0.889305
C	0.459244	-0.135550	3.340156
H	-0.537189	1.670071	3.975485
H	1.194210	-1.984932	2.509241
H	1.279041	-0.029153	4.044519
C	-5.337361	0.339351	-0.541058
C	-6.311471	-0.658109	-0.578396
C	-5.561543	1.538641	0.138757
C	-7.525803	-0.455987	0.075687
H	-6.099681	-1.574799	-1.117793
C	-6.779111	1.721733	0.795670
H	-4.787388	2.296279	0.147737
C	-7.764027	0.733103	0.767921
H	-8.284624	-1.232532	0.046756
H	-6.957893	2.651309	1.328549
H	-8.709090	0.887732	1.279503
C	4.324254	2.230326	-0.232625
C	2.528325	-2.600877	-0.091229
C	1.771846	-3.735313	0.239782
C	3.536275	-2.179601	0.788240
C	2.030030	-4.439942	1.412922
H	0.961557	-4.043813	-0.412827
C	3.799346	-2.889014	1.958666
H	4.123213	-1.300852	0.541384
C	3.046993	-4.021407	2.274977
H	1.427827	-5.307766	1.662658
H	4.588447	-2.555599	2.625615
H	3.242905	-4.568070	3.191969
H	3.070246	3.142998	1.271093
H	4.717983	1.428229	0.407178
H	5.143086	2.523960	-0.899862

***Int*-4**$_{\text{3a-}t}$

Pd	0.345961	-1.007132	-0.151601
O	-2.438546	1.369914	-0.834448
O	-1.448194	-1.036354	-1.173396
C	2.999220	0.252337	-0.040745
C	3.064574	5.862595	0.225936
C	2.579127	4.595824	-0.418994
C	3.092624	2.153387	-0.929263
C	3.944221	0.895014	-0.763219
H	3.285506	5.797711	1.292431
H	2.764057	2.310829	-1.965408
H	2.323977	4.786333	-1.468414
C	2.679341	-1.078541	0.472500
C	1.767493	-1.168125	1.521567
H	1.556195	-2.132206	1.975204
C	5.313932	0.587417	-1.255678
H	6.031108	1.331140	-0.887139
H	5.646630	-0.403036	-0.938680
H	5.352971	0.630129	-2.351754
C	1.989237	1.377779	-0.083845
H	1.763073	1.853358	0.876878
C	0.740491	0.913527	-0.785577
H	0.890349	0.715684	-1.851033
H	-0.186919	1.458030	-0.611291
C	3.256217	7.019009	-0.407796
H	3.619175	7.902288	0.108970
H	3.049270	7.124882	-1.470242
H	1.491499	-0.299437	2.109671
P	-2.581262	-0.107124	-0.682927
O	-2.915220	-0.531644	0.889432
O	-3.894253	-0.758057	-1.376727
C	-2.039262	-0.225605	1.907485
C	-1.534137	1.067123	2.095178
C	-1.693632	-1.257221	2.785906
C	-0.671528	1.310491	3.164090
H	-1.822603	1.847850	1.402135
C	-0.841424	-0.995074	3.858633
H	-2.109323	-2.244652	2.616121
C	-0.321587	0.287308	4.049912
H	-0.282473	2.313799	3.313270
H	-0.583286	-1.796156	4.545114
H	0.339788	0.490122	4.886578
C	-5.189917	-0.305299	-1.150401
C	-6.172475	-1.286457	-1.026220
C	-5.513609	1.051613	-1.102297
C	-7.501368	-0.905038	-0.847262
H	-5.879203	-2.329514	-1.071347
C	-6.847512	1.416308	-0.914064
H	-4.728698	1.791115	-1.203047
C	-7.844017	0.447255	-0.787865
H	-8.268333	-1.667826	-0.750688
H	-7.106305	2.470295	-0.871762
H	-8.878930	0.742682	-0.645488
C	3.613273	3.459462	-0.338509
C	3.381662	-2.268402	-0.068506
C	3.798911	-3.305443	0.779001
C	3.635624	-2.373280	-1.446101
C	4.451018	-4.423198	0.261125
H	3.629940	-3.222274	1.847858
C	4.281550	-3.492596	-1.962517

H	3.302912	-1.578193	-2.104623
C	4.691878	-4.520812	-1.109985
H	4.775739	-5.215029	0.929049
H	4.460181	-3.566818	-3.030689
H	5.197816	-5.392648	-1.513014
H	1.653809	4.262506	0.072428
H	3.900606	3.290157	0.707971
H	4.526353	3.764785	-0.865342

***TS*-3$_{2a\text{-}t}$**

Pd	-0.318504	-0.005396	-1.161318
O	-0.662403	-0.599156	0.968502
O	0.539672	1.485837	2.024416
C	-0.494811	-1.942293	-2.461918
C	0.081748	-1.118504	-3.642801
C	1.549812	-1.464301	-3.891901
C	1.848169	-2.333032	-1.518590
C	0.323339	-2.422941	-1.453585
H	-0.507700	-1.335213	-4.540408
C	-1.916839	-2.390095	-2.629786
C	-2.147594	-3.619317	-3.108699
H	-3.158929	-3.968457	-3.292157
C	-0.212328	-3.258404	-0.323093
H	0.203633	-2.907086	0.623518
H	-1.298459	-3.216633	-0.264261
H	0.093653	-4.300446	-0.459135
C	2.404200	-3.724227	-1.755110
C	3.033922	-4.457318	-0.838521
H	3.161486	-4.102481	0.179611
H	3.428915	-5.441902	-1.070831
C	-0.121769	0.308559	-3.154786
H	-1.072019	0.746405	-3.469640
H	0.714774	0.991042	-3.323207
H	-1.334805	-4.307719	-3.321010
P	-0.426236	0.341518	2.135339
O	-1.846990	0.981806	2.644968
O	-0.110056	-0.598308	3.447534
C	-3.068454	0.340402	2.647129
C	-4.184750	1.175010	2.745011
C	-3.221715	-1.046818	2.573407
C	-5.463440	0.620145	2.761911
H	-4.028136	2.246367	2.807691
C	-4.509078	-1.585855	2.582519
H	-2.355157	-1.688563	2.490269
C	-5.632471	-0.763518	2.675746
H	-6.327687	1.273151	2.837879
H	-4.629047	-2.662884	2.513923
H	-6.627984	-1.195376	2.680187
C	0.741062	-1.683697	3.359347
C	1.905214	-1.654083	2.587652
C	0.399869	-2.814727	4.100875
C	2.734747	-2.775948	2.581597
H	2.153495	-0.775590	2.003017
C	1.237919	-3.929837	4.078365
H	-0.514160	-2.800859	4.684771
C	2.409990	-3.915822	3.320188
H	3.652331	-2.750639	2.001498
H	0.971942	-4.809886	4.656171
H	3.064345	-4.781738	3.307235
C	2.362812	-1.366326	-2.600675
C	-3.037085	-1.442715	-2.372570
C	-3.878173	-1.043874	-3.419620
C	-3.281786	-0.958624	-1.077347
C	-4.941068	-0.170767	-3.180436
H	-3.688503	-1.417739	-4.421335
C	-4.358398	-0.106648	-0.836295
H	-2.644517	-1.253002	-0.251257
C	-5.184646	0.295608	-1.887800
H	-5.580182	0.138233	-4.002479
H	-4.543678	0.244126	0.171958
H	-6.016162	0.968110	-1.698806
H	3.416706	-1.583549	-2.800245
H	2.314865	-0.348792	-2.201678
H	2.269511	-4.120912	-2.761754
H	2.195871	-1.987377	-0.540320
H	1.617124	-2.478972	-4.304485
H	1.956386	-0.785530	-4.650456
C	-0.971000	3.226656	-0.780012
C	-0.491421	4.243436	0.325955
B	1.022263	2.495169	0.269710
O	-0.076353	2.086904	-0.552367
O	0.859889	3.820007	0.601994
C	-0.429870	5.693782	-0.151790
H	-0.067074	6.321060	0.666361
H	-1.421929	6.054127	-0.443002
H	0.252479	5.813346	-0.994330
C	-1.294552	4.172286	1.628150
H	-2.311521	4.552292	1.485981
H	-0.794811	4.798557	2.371543
H	-1.339289	3.160494	2.022877
C	-0.701702	3.707964	-2.206296
H	-1.325373	4.570070	-2.457005
H	-0.933294	2.906024	-2.909163
H	0.347696	3.984910	-2.332693
C	-2.407045	2.747487	-0.636644
H	-2.639801	2.019536	-1.417312
H	-3.101897	3.587218	-0.738583
H	-2.574097	2.270273	0.328703
C	4.828288	1.857144	-0.347492
C	4.455290	0.552333	0.434074
B	2.588422	1.849679	0.147459
O	3.647068	2.675429	-0.152303
O	2.999836	0.536819	0.319703
C	5.021808	-0.732928	-0.153532
H	4.664523	-1.594039	0.416477
H	6.115223	-0.725450	-0.100897
H	4.726001	-0.872050	-1.192058
C	4.781958	0.640846	1.928699
H	5.861891	0.632331	2.100829
H	4.346167	-0.213747	2.450042
H	4.362955	1.550014	2.366876
C	4.972872	1.640684	-1.857609
H	5.886899	1.091588	-2.102033
H	5.012822	2.615917	-2.349397
H	4.118114	1.091980	-2.260842
C	6.037546	2.611141	0.193813
H	6.205610	3.510695	-0.404408
H	6.938790	1.991608	0.138724
H	5.884587	2.918488	1.228976

***Int*-5$_{2a\text{-}c}$**

Pd	-0.685241	-1.115900	0.353366
O	0.501720	-0.655360	-1.493003
O	2.409035	0.484666	-0.124749
C	-2.778883	-1.171911	-0.328025
C	-3.284560	-1.534906	1.083942
C	-3.788283	-2.977844	1.143554
C	-2.143223	-3.640324	-0.695405
C	-2.154755	-2.158949	-1.094703
H	-4.086316	-0.846995	1.367238
H	-2.808417	-4.115763	-1.433098
H	-4.666817	-3.078845	0.494206
C	-3.232692	0.137834	-0.893036
C	-2.409965	1.095822	-1.346269
H	-2.818152	1.996205	-1.793557
C	-1.834589	-1.948394	-2.552813
H	-2.619281	-2.434822	-3.148711
H	-1.784379	-0.898766	-2.823113
H	-0.885030	-2.416190	-2.810995
C	-0.770714	-4.247769	-0.866242
H	-0.018241	-3.878590	-0.169791
C	-0.444110	-5.166163	-1.774022
H	-1.170260	-5.545870	-2.488792
H	0.561193	-5.570379	-1.840987
C	-2.013975	-1.299828	1.884617
H	-1.954456	-0.332258	2.384646
H	-1.723163	-2.111927	2.550446
H	-1.331317	1.008952	-1.303507
C	-2.691456	-3.950270	0.710436
C	-4.709630	0.309495	-0.924016

C	-5.298970	1.540672	-0.598192
C	-5.546141	-0.762738	-1.272656
C	-6.682640	1.700306	-0.640712
H	-4.663510	2.365480	-0.291803
C	-6.929804	-0.603712	-1.314768
H	-5.104174	-1.720775	-1.528837
C	-7.503315	0.629276	-0.999370
H	-7.121881	2.659033	-0.381406
H	-7.560529	-1.441686	-1.596346
H	-8.581575	0.752843	-1.026795
H	-4.113588	-3.210293	2.164134
H	-3.055924	-4.982360	0.719881
H	-1.867872	-3.913004	1.430845
C	1.593272	-1.413601	2.684110
C	3.039567	-0.806929	2.706774
B	1.758191	0.572353	1.327656
O	1.043155	-0.745322	1.517347
O	2.816951	0.539750	2.277538
C	3.672531	-0.756991	4.096263
H	4.682656	-0.345627	4.015365
H	3.747784	-1.757653	4.535784
H	3.101111	-0.114398	4.767361
C	3.990164	-1.519673	1.734897
H	4.296708	-2.500025	2.113601
H	4.883746	-0.902904	1.610948
H	3.538309	-1.650276	0.753851
C	0.783451	-0.996453	3.914561
H	1.178320	-1.464437	4.820664
H	-0.254722	-1.308385	3.802630
H	0.805317	0.088848	4.032203
C	1.527981	-2.918386	2.458441
H	0.485317	-3.251203	2.441682
H	2.037974	-3.451245	3.267521
H	1.993326	-3.193543	1.510122
C	-0.870978	3.377976	2.207488
C	-0.318220	3.885563	0.832244
B	0.700942	1.915577	1.396569
O	0.088553	2.346478	2.558153
O	0.328069	2.692518	0.313902
C	-1.372126	4.357292	-0.160207
H	-0.886562	4.620325	-1.103663
H	-1.889006	5.244722	0.219991
H	-2.110825	3.581786	-0.359767
C	0.778934	4.946074	0.974749
H	0.370302	5.906574	1.303043
H	1.264389	5.084002	0.006506
H	1.539130	4.625496	1.691950
C	-2.237402	2.693358	2.088920
H	-3.037854	3.416745	1.904452
H	-2.454084	2.175525	3.027663
H	-2.238936	1.958040	1.280818
C	-0.894211	4.421465	3.317670
H	-1.274026	3.970798	4.238976
H	-1.549920	5.257378	3.051651
H	0.105079	4.810005	3.518569
P	1.752195	0.183198	-1.476921
O	1.450383	1.515887	-2.325897
O	2.915927	-0.482627	-2.400223
C	2.049508	2.764802	-2.234845
C	1.371738	3.789588	-2.893443
C	3.246239	3.007280	-1.563028
C	1.895093	5.080839	-2.874978
H	0.444496	3.557077	-3.405153
C	3.755538	4.306905	-1.551122
H	3.752404	2.210198	-1.035371
C	3.089991	5.346270	-2.201836
H	1.366334	5.878978	-3.387313
H	4.682766	4.502754	-1.021281
H	3.496618	6.352359	-2.184240
C	3.506577	-1.672934	-1.969140
C	2.738153	-2.811506	-1.719445
C	4.889494	-1.683239	-1.810557
C	3.378748	-3.970636	-1.279557
H	1.664772	-2.777579	-1.854370
C	5.516481	-2.853064	-1.381725
H	5.449010	-0.775973	-2.008706
C	4.763822	-3.996634	-1.107221
H	2.788026	-4.857806	-1.070861
H	6.593948	-2.864725	-1.249871
H	5.254091	-4.901737	-0.763070

***Int*-5**$_{2a-t}$

Pd	-0.237277	-0.022059	1.078165
O	-0.888931	0.820491	-0.926253
O	0.463511	-1.053012	-2.168115
C	-0.485855	1.642690	2.713489
C	0.555210	0.841828	3.541344
C	1.961172	1.437576	3.428524
C	1.355768	2.774488	1.359591
C	-0.106358	2.479688	1.685167
H	0.238461	0.845793	4.590445
C	-1.890633	1.610008	3.260236
C	-2.174384	2.416614	4.292655
H	-3.162966	2.433337	4.740110
C	-1.093953	3.312761	0.915780
H	-1.096241	2.986696	-0.128999
H	-2.103862	3.236146	1.317787
H	-0.782953	4.360884	0.938601
C	1.686718	4.179570	1.823612
C	1.964905	5.197152	1.009596
H	1.954171	5.076262	-0.069990
H	2.215796	6.180681	1.396355
C	0.437329	-0.540356	2.916003
H	-0.331902	-1.167692	3.371217
H	1.376643	-1.081888	2.788481
H	-1.429008	3.088723	4.708251
P	-0.565124	0.074727	-2.196311
O	-1.890257	-0.543080	-2.898649
O	-0.122476	1.133647	-3.348747
C	-3.178184	-0.055825	-2.725823
C	-4.203334	-0.994139	-2.845630
C	-3.459367	1.287554	-2.471852
C	-5.527848	-0.583833	-2.696705
H	-3.946713	-2.027194	-3.049924
C	-4.789160	1.680444	-2.314932
H	-2.656106	2.007168	-2.383949
C	-5.826239	0.752850	-2.424191
H	-6.325695	-1.314401	-2.788201
H	-5.011182	2.722556	-2.106572
H	-6.856751	1.068779	-2.298817
C	0.771020	2.163484	-3.059087
C	1.971880	1.925132	-2.390415
C	0.421962	3.439586	-3.495769
C	2.830459	3.000650	-2.158937
H	2.240515	0.929902	-2.054152
C	1.297237	4.501684	-3.266069
H	-0.521280	3.580520	-4.012383
C	2.504345	4.286265	-2.597455
H	3.761997	2.824516	-1.629841
H	1.032865	5.497026	-3.609917
H	3.186074	5.112778	-2.422254
C	2.330093	1.758456	1.978468
C	-2.908404	0.655171	2.738156
C	-3.592850	-0.178785	3.635625
C	-3.224448	0.573054	1.370766
C	-4.570480	-1.065350	3.183269
H	-3.339794	-0.136274	4.690504
C	-4.213447	-0.300781	0.922506
H	-2.716189	1.194061	0.645638
C	-4.887426	-1.124053	1.825721
H	-5.081451	-1.709958	3.892442
H	-4.448611	-0.338709	-0.134615
H	-5.649017	-1.812305	1.471570
H	3.344977	2.164456	1.924485
H	2.317067	0.849106	1.367334
H	1.696888	4.333741	2.902632
H	1.459711	2.749992	0.269791
H	2.014137	2.350710	4.033953
H	2.682072	0.733354	3.859401
C	-0.545290	-3.222756	0.498875

C	-0.455091	-3.983543	-0.870097
B	0.924139	-2.087347	-1.034858
O	-0.045477	-1.915384	0.106217
O	0.712973	-3.425211	-1.473757
C	-0.236839	-5.489193	-0.727134
H	-0.204089	-5.943718	-1.721309
H	-1.051739	-5.958044	-0.164681
H	0.709996	-5.702554	-0.229329
C	-1.670716	-3.724552	-1.770414
H	-2.562332	-4.248720	-1.410233
H	-1.436503	-4.084635	-2.775565
H	-1.891315	-2.661216	-1.842715
C	0.400886	-3.804553	1.553232
H	0.066232	-4.794755	1.875002
H	0.412767	-3.154974	2.430006
H	1.417548	-3.879828	1.162145
C	-1.951178	-3.061888	1.056319
H	-1.932241	-2.485161	1.985214
H	-2.388853	-4.041465	1.274817
H	-2.600007	-2.535939	0.357452
C	4.592168	-2.038982	0.399458
C	4.652335	-0.807581	-0.565225
B	2.551022	-1.738336	-0.619190
O	3.356861	-2.678057	-0.003692
O	3.241146	-0.543952	-0.797882
C	5.312810	0.436331	0.014300
H	5.284874	1.246287	-0.720369
H	6.362638	0.236673	0.252695
H	4.809855	0.778595	0.917868
C	5.263875	-1.140468	-1.930303
H	6.345692	-1.288524	-1.864089
H	5.068572	-0.311207	-2.615393
H	4.811097	-2.041235	-2.352123
C	4.431077	-1.643633	1.871630
H	5.351463	-1.218403	2.282761
H	4.173371	-2.535654	2.448595
H	3.625636	-0.915881	1.993923
C	5.734178	-3.036376	0.243092
H	5.594678	-3.866289	0.941276
H	6.697298	-2.563844	0.463280
H	5.767031	-3.447922	-0.766525

***TS*-4**$_{2a\text{-}c}$

Pd	-0.793284	-0.697589	0.247194
O	0.373819	-0.488483	-1.698115
O	2.271434	0.411483	-0.172580
C	-2.924205	-0.902288	-0.288689
C	-3.340637	-1.199652	1.164251
C	-3.769315	-2.656876	1.342085
C	-2.177535	-3.359806	-0.530252
C	-2.286114	-1.911757	-1.019129
H	-4.157814	-0.530423	1.449321
H	-2.842488	-3.921585	-1.205355
H	-4.672512	-2.840446	0.746791
C	-3.495780	0.332266	-0.912666
C	-2.763915	1.356382	-1.377406
H	-3.246969	2.204475	-1.853848
C	-2.056023	-1.774665	-2.503000
H	-2.884353	-2.271816	-3.027510
H	-2.009776	-0.737840	-2.822784
H	-1.130433	-2.268463	-2.797371
C	-0.778742	-3.901544	-0.716886
H	-0.009380	-3.404248	-0.125650
C	-0.458205	-4.912068	-1.523761
H	-1.204488	-5.415327	-2.134132
H	0.561536	-5.274286	-1.607339
C	-2.042826	-0.847978	1.867362
H	-2.016352	0.126641	2.342080
H	-1.623061	-1.617794	2.510488
H	-1.682668	1.370494	-1.306012
C	-2.648092	-3.604358	0.916757
C	-4.982346	0.348270	-0.966430
C	-5.701576	1.526763	-0.713751
C	-5.699215	-0.822837	-1.259650
C	-7.093484	1.538817	-0.774621
H	-5.159566	2.429140	-0.449031
C	-7.091370	-0.811497	-1.320259
H	-5.158020	-1.742673	-1.457196
C	-7.794091	0.370026	-1.078812
H	-7.632981	2.459247	-0.571815
H	-7.627726	-1.725269	-1.558278
H	-8.878936	0.378652	-1.120390
H	-4.032239	-2.835215	2.391261
H	-2.961828	-4.649192	1.006287
H	-1.795390	-3.486423	1.593136
C	1.993994	-1.944916	2.363202
C	3.037724	-0.822902	2.689849
B	1.539776	0.005790	1.139969
O	1.393601	-1.447755	1.149441
O	2.357865	0.366266	2.246480
C	3.358889	-0.660264	4.172309
H	4.098987	0.134800	4.300859
H	3.776863	-1.583797	4.587485
H	2.468722	-0.386558	4.740788
C	4.334689	-0.976480	1.882382
H	4.949056	-1.801432	2.257426
H	4.909106	-0.050105	1.970409
H	4.126364	-1.146952	0.825578
C	0.909498	-2.058949	3.439038
H	1.309063	-2.467296	4.371919
H	0.128818	-2.737298	3.083314
H	0.461690	-1.083589	3.643210
C	2.594581	-3.316579	2.076923
H	1.795986	-4.024971	1.833152
H	3.126355	-3.697600	2.955537
H	3.282721	-3.281540	1.233236
C	-0.898682	3.025788	2.302464
C	-0.144581	3.539983	1.025740
B	0.196343	1.284226	1.275132
O	-0.335892	1.695487	2.477693
O	0.158629	2.295364	0.333763
C	-0.966679	4.426802	0.100053
H	-0.360596	4.705343	-0.765147
H	-1.269787	5.344193	0.615426
H	-1.860956	3.913618	-0.256651
C	1.203879	4.193679	1.340814
H	1.072941	5.157927	1.840844
H	1.746597	4.356804	0.408543
H	1.810031	3.542468	1.975959
C	-2.406357	2.857483	2.087074
H	-2.907515	3.826902	2.009441
H	-2.831226	2.324385	2.942589
H	-2.617003	2.281045	1.183376
C	-0.632392	3.827297	3.570821
H	-1.181803	3.385115	4.406590
H	-0.967444	4.863065	3.452398
H	0.428294	3.826869	3.824078
P	1.680207	0.245970	-1.588442
O	1.539597	1.665467	-2.332141
O	2.857916	-0.413279	-2.491365
C	2.262656	2.824096	-2.092046
C	1.688840	3.994134	-2.588115
C	3.489677	2.848643	-1.430114
C	2.345499	5.210192	-2.408288
H	0.736524	3.930135	-3.102476
C	4.132988	4.075108	-1.254924
H	3.919609	1.939486	-1.032009
C	3.569775	5.256753	-1.737722
H	1.896608	6.121281	-2.792099
H	5.083247	4.100186	-0.730440
H	4.078878	6.204201	-1.593234
C	3.467361	-1.600379	-2.078631
C	2.717908	-2.747647	-1.824324
C	4.854712	-1.594673	-1.960376
C	3.386646	-3.909957	-1.440233
H	1.639454	-2.721287	-1.916832
C	5.508923	-2.765867	-1.578413
H	5.397189	-0.677900	-2.163147
C	4.777079	-3.925500	-1.315268
H	2.814251	-4.807475	-1.227709

H	6.590119	-2.768052	-1.481128
H	5.286982	-4.834808	-1.013277

***TS*-4$_{2a\text{-}t}$**

Pd	-0.133453	0.651399	-0.452276
O	0.339560	0.520754	1.766845
O	0.292442	-2.024377	1.352351
C	-0.486786	2.827440	-0.863088
C	-0.622892	2.556710	-2.378744
C	-2.067873	2.702038	-2.853262
C	-2.924307	2.059221	-0.550134
C	-1.529301	2.409317	-0.018471
H	0.026164	3.243532	-2.930697
C	0.630131	3.721846	-0.454838
C	0.409893	4.804459	0.309448
H	1.220145	5.484300	0.552118
C	-1.523184	2.673838	1.467849
H	-2.037546	1.863631	1.985074
H	-0.517759	2.757432	1.869817
H	-2.079568	3.591906	1.683299
C	-3.868668	3.198250	-0.219623
C	-4.909452	3.099852	0.605362
H	-5.122853	2.175813	1.135150
H	-5.576964	3.937095	0.786218
C	-0.116971	1.129779	-2.439896
H	0.948233	1.053559	-2.649839
H	-0.707620	0.403480	-2.991840
H	-0.570246	5.041168	0.704896
P	0.470753	-0.860338	2.351560
O	1.833013	-1.087509	3.191989
O	-0.538045	-1.105572	3.589826
C	3.102849	-0.837877	2.672737
C	4.052735	-1.846657	2.812340
C	3.429669	0.395968	2.112047
C	5.358590	-1.615118	2.378075
H	3.758848	-2.789936	3.258609
C	4.738614	0.612589	1.681776
H	2.668699	1.159829	2.019187
C	5.704849	-0.386969	1.811171
H	6.102855	-2.398459	2.482905
H	4.996862	1.570246	1.241995
H	6.721355	-0.209770	1.474265
C	-1.768962	-0.443810	3.615050
C	-2.762840	-0.766369	2.694531
C	-1.967060	0.519409	4.600390
C	-3.984580	-0.094334	2.772086
H	-2.575388	-1.505586	1.923977
C	-3.195013	1.178734	4.666756
H	-1.161823	0.742231	5.291478
C	-4.205967	0.874990	3.752571
H	-4.767913	-0.334469	2.059665
H	-3.357510	1.933303	5.430102
H	-5.159221	1.391630	3.804249
C	-2.978252	1.764824	-2.060152
C	1.980845	3.512295	-1.054956
C	2.571772	4.549450	-1.793262
C	2.696324	2.317865	-0.882562
C	3.843394	4.401904	-2.346057
H	2.016487	5.470848	-1.939479
C	3.973342	2.176923	-1.425014
H	2.268332	1.498852	-0.314198
C	4.549862	3.213404	-2.161173
H	4.279138	5.213513	-2.921211
H	4.521520	1.256906	-1.256897
H	5.541828	3.094513	-2.586655
H	-4.015189	1.848110	-2.402152
H	-2.663115	0.733731	-2.233945
H	-3.667141	4.142987	-0.725625
H	-3.266443	1.179937	0.005669
H	-2.389363	3.745211	-2.739913
H	-2.121205	2.464122	-3.921888
C	2.387433	-1.980324	-1.446518
C	1.892865	-3.425426	-1.095095
B	0.406718	-1.894746	-0.180965
O	1.625796	-1.163314	-0.527488
O	0.518230	-3.212024	-0.735697
C	1.935859	-4.411858	-2.258629
H	1.580515	-5.389833	-1.921146
H	2.957648	-4.533327	-2.633878
H	1.295443	-4.083629	-3.078627
C	2.615164	-4.017877	0.122872
H	3.647350	-4.297101	-0.111807
H	2.076178	-4.912956	0.444233
H	2.622965	-3.311727	0.953248
C	2.026504	-1.573085	-2.878196
H	2.588024	-2.156683	-3.613599
H	2.283819	-0.520939	-3.021962
H	0.957147	-1.700453	-3.061072
C	3.872445	-1.752433	-1.191822
H	4.151901	-0.746283	-1.511340
H	4.474780	-2.463921	-1.767221
H	4.117155	-1.853997	-0.135526
C	-2.641519	-2.485803	-2.464967
C	-3.356896	-2.498715	-1.060899
B	-1.242079	-1.558251	-0.891489
O	-1.533909	-1.570831	-2.248860
O	-2.299215	-2.067952	-0.163096
C	-4.503968	-1.493825	-0.936510
H	-4.836270	-1.463835	0.104200
H	-5.357482	-1.783399	-1.556243
H	-4.191848	-0.489715	-1.223117
C	-3.825625	-3.874765	-0.596263
H	-4.579538	-4.281687	-1.277987
H	-4.275419	-3.790530	0.396989
H	-2.992209	-4.574601	-0.531078
C	-3.490420	-1.956075	-3.615041
H	-4.352209	-2.607406	-3.792755
H	-2.889512	-1.933608	-4.528318
H	-3.853217	-0.945245	-3.422611
C	-2.016159	-3.832749	-2.842038
H	-1.425833	-3.698658	-3.752876
H	-2.779075	-4.592275	-3.035554
H	-1.341190	-4.177783	-2.056791

***Int*-6$_{2a\text{-}c}$**

Pd	-0.653882	0.383434	-0.432855
C	1.968372	0.369930	-0.053093
C	1.480600	-0.118715	1.321901
C	1.574692	1.003423	2.361579
C	1.490574	2.786461	0.554690
C	1.932320	1.680713	-0.408642
H	2.149940	-0.931852	1.633759
H	2.407036	3.349846	0.792678
H	2.634240	1.216064	2.557002
C	2.537884	-0.700340	-0.926202
C	1.954517	-1.085894	-2.072277
H	2.393951	-1.850252	-2.705593
C	2.466166	2.192730	-1.724168
H	3.179888	3.006150	-1.544700
H	2.956463	1.409284	-2.300895
H	1.665289	2.623308	-2.334407
C	0.532463	3.745385	-0.109467
H	-0.426024	3.307350	-0.396693
C	0.762332	5.038511	-0.329203
H	1.704808	5.502863	-0.047893
H	0.020163	5.681184	-0.792882
C	0.065240	-0.689975	1.148475
H	0.064435	-1.730817	0.820301
H	-0.573641	-0.580042	2.025290
H	1.019597	-0.644002	-2.401474
C	0.877194	2.268258	1.869895
C	3.788120	-1.334763	-0.430508
C	4.065493	-2.690985	-0.665556
C	4.723463	-0.577958	0.293687
C	5.251931	-3.264279	-0.214515
H	3.332335	-3.299865	-1.184666
C	5.910093	-1.151503	0.746342
H	4.516128	0.469484	0.489391
C	6.180723	-2.496563	0.491507
H	5.446369	-4.316172	-0.402165

H	6.624127	-0.547284	1.298210
H	7.102489	-2.946085	0.848137
H	1.134683	0.667214	3.306943
H	0.923652	3.066176	2.618314
H	-0.183912	2.047409	1.704941
C	-4.311105	-1.505304	0.595683
C	-4.761406	-0.406549	-0.429080
B	-2.494178	-0.318791	-0.121613
O	-2.959233	-1.082926	0.926714
O	-3.492856	-0.013608	-1.029374
C	-5.692765	-0.894617	-1.531519
H	-5.934384	-0.066130	-2.203053
H	-6.629116	-1.272657	-1.107531
H	-5.231362	-1.686575	-2.123020
C	-5.338265	0.843041	0.243046
H	-6.329263	0.653701	0.666209
H	-5.426991	1.636438	-0.503600
H	-4.679016	1.197587	1.039698
C	-4.197429	-2.899654	-0.028448
H	-5.180546	-3.327963	-0.245140
H	-3.679528	-3.558170	0.673574
H	-3.617259	-2.866535	-0.954278
C	-5.130493	-1.563987	1.879132
H	-4.731619	-2.343321	2.534293
H	-6.176126	-1.805290	1.660842
H	-5.094890	-0.616970	2.419517

***Int*-6$_{2a\text{-}t}$**

Pd	0.785636	0.470240	-0.494179
C	-1.850701	0.222322	-0.150987
C	-1.334986	-0.041969	1.274307
C	-1.475570	1.194578	2.170314
C	-1.718972	2.729988	0.167802
C	-1.948033	1.471715	-0.674238
H	-1.947513	-0.847472	1.703408
H	-2.533090	1.322900	2.432605
C	-2.271694	-1.009475	-0.886108
C	-1.592893	-1.509656	-1.930048
H	-1.935405	-2.393906	-2.458715
C	-2.414425	1.748142	-2.082003
H	-3.250564	2.454722	-2.071391
H	-2.717665	0.840595	-2.602829
H	-1.611003	2.220988	-2.661625
C	-3.058096	3.376771	0.450697
H	-3.724985	2.810739	1.101105
C	-3.455829	4.554422	-0.029365
H	-2.818359	5.144418	-0.683819
H	-4.426271	4.975213	0.216341
C	0.110363	-0.544654	1.143140
H	0.167964	-1.600171	0.871816
H	0.732561	-0.351277	2.017287
H	-0.674938	-1.045235	-2.275704
C	-0.956203	2.454431	1.477926
C	-3.496044	-1.669093	-0.357511
C	-3.641535	-3.065535	-0.380196
C	-4.536967	-0.898105	0.184896
C	-4.801403	-3.669254	0.099453
H	-2.827250	-3.676879	-0.755655
C	-5.697268	-1.502067	0.665515
H	-4.433551	0.181905	0.214397
C	-5.835547	-2.890017	0.622651
H	-4.893151	-4.751215	0.077749
H	-6.494688	-0.887743	1.073313
H	-6.736870	-3.361887	1.001987
H	-0.937247	1.026821	3.109413
H	-1.041699	3.325654	2.135877
H	0.107677	2.322625	1.246345
C	4.533253	-1.115702	0.694744
C	4.933755	-0.068345	-0.401938
B	2.662341	-0.084999	-0.116259
O	3.157420	-0.744887	0.987452
O	3.651721	0.211492	-1.036442
C	5.900839	-0.580830	-1.461716
H	6.104401	0.210730	-2.188144
H	6.851820	-0.877270	-1.006540
H	5.488335	-1.435866	-1.999038
C	5.435525	1.254593	0.184339
H	6.430768	1.149023	0.626288
H	5.489338	1.997615	-0.615549
H	4.749961	1.627447	0.949770
C	4.499813	-2.554171	0.169024
H	5.506323	-2.942999	-0.011654
H	4.011098	-3.189189	0.912397
H	3.927441	-2.616657	-0.760146
C	5.342153	-1.040643	1.983931
H	4.979100	-1.792889	2.689626
H	6.401221	-1.239896	1.789266
H	5.250849	-0.061637	2.456497
H	-1.135878	3.437235	-0.436994

***TS*-5$_{2a\text{-}c}$**

Pd	0.128645	-1.016115	-0.382424
C	1.956150	0.923031	1.081167
C	0.626924	0.959569	1.830692
C	-0.072834	2.319509	1.667303
C	1.512745	3.087326	-0.129362
C	2.393467	1.908794	0.273184
H	0.902219	0.869925	2.895180
H	1.885449	3.983053	0.393264
H	0.388824	3.038679	2.356779
C	2.795465	-0.296252	1.336538
C	3.558753	-0.394309	2.434571
H	4.184175	-1.259845	2.630345
C	3.790473	1.936736	-0.298096
H	4.416845	1.140784	0.106179
H	3.788410	1.863354	-1.391396
H	4.265003	2.898947	-0.066547
C	1.610551	3.350450	-1.612508
H	1.280813	2.526398	-2.247701
C	2.020985	4.488433	-2.169862
H	2.350870	5.329067	-1.563694
H	2.039620	4.625855	-3.246926
C	-0.302184	-0.254585	1.572189
H	0.092567	-1.163676	2.039313
H	-1.239560	-0.068860	2.108572
H	3.594319	0.412237	3.159662
C	0.042095	2.854955	0.245988
C	2.694791	-1.408157	0.346222
C	2.815976	-2.751728	0.746464
C	2.456246	-1.144829	-1.028865
C	2.682756	-3.794639	-0.167107
H	2.971983	-2.974683	1.796512
C	2.291901	-2.203495	-1.937257
H	2.463859	-0.122699	-1.386811
C	2.398487	-3.529349	-1.508451
H	2.769219	-4.821048	0.176145
H	2.113371	-1.979809	-2.984799
H	2.267349	-4.343230	-2.214161
H	-1.123656	2.228540	1.958315
H	-0.512451	3.792989	0.134550
H	-0.406755	2.137667	-0.446091
C	-3.728349	0.684231	-0.094793
C	-3.978681	-0.862619	-0.009938
B	-1.732945	-0.430402	0.148490
O	-2.294139	0.748696	-0.319972
O	-2.712359	-1.351008	0.503024
C	-5.087448	-1.283963	0.947335
H	-5.173561	-2.373949	0.952180
H	-6.050485	-0.867617	0.633463
H	-4.880248	-0.957184	1.967488
C	-4.186412	-1.511466	-1.382670
H	-5.156378	-1.247290	-1.814320
H	-4.141572	-2.597461	-1.267653
H	-3.398055	-1.211349	-2.077946
C	-4.005018	1.412863	1.223876
H	-5.076311	1.469254	1.437637
H	-3.612004	2.430510	1.154089
H	-3.512582	0.913722	2.062745
C	-4.431688	1.392583	-1.245743
H	-4.176207	2.455802	-1.233510

H	-5.518887	1.301998	-1.151366
H	-4.128056	0.983158	-2.210146

***TS*-5**$_{2a\text{-}t}$

Pd	-0.504506	0.168504	-1.445453
C	2.071503	-0.300069	-0.261041
C	1.047596	-0.898807	0.705225
C	1.661876	-1.997064	1.590749
C	3.733596	-2.141120	0.159653
C	3.280605	-0.844111	-0.506174
H	0.708776	-0.085882	1.358688
H	2.247453	-1.532132	2.393540
C	1.607187	0.961418	-0.932291
C	1.538735	1.084088	-2.305139
H	1.387579	2.052913	-2.770653
C	4.317722	-0.236212	-1.416774
H	5.299000	-0.264777	-0.931567
H	4.089804	0.794192	-1.688716
H	4.408474	-0.817077	-2.344301
C	4.798746	-1.848678	1.193051
H	4.475438	-1.262932	2.053149
C	6.069726	-2.241343	1.111909
H	6.431387	-2.820655	0.265416
H	6.793670	-2.004078	1.885823
C	-0.200717	-1.405175	-0.051892
H	-0.889177	-1.833729	0.683188
H	0.031443	-2.206324	-0.760507
H	1.841149	0.264488	-2.947215
C	2.557408	-2.923460	0.770053
C	1.402108	2.146761	-0.047835
C	0.486148	3.163177	-0.366149
C	2.158616	2.274104	1.126471
C	0.338676	4.273656	0.459082
H	-0.132720	3.064642	-1.252938
C	2.012087	3.387459	1.953288
H	2.872527	1.496350	1.377719
C	1.102011	4.391181	1.623748
H	-0.382585	5.043244	0.200776
H	2.609961	3.468722	2.856270
H	0.981180	5.254673	2.270807
H	0.862183	-2.564296	2.079650
H	2.942201	-3.745923	1.381988
H	1.970574	-3.371789	-0.040407
C	-4.140086	-1.347187	0.152462
C	-3.934408	0.018217	0.898216
B	-2.079384	-0.479432	-0.342146
O	-3.056291	-1.332926	-0.819670
O	-2.501397	0.223101	0.774282
C	-4.297301	0.006459	2.378295
H	-4.101207	0.990914	2.811768
H	-5.359568	-0.221226	2.515868
H	-3.707452	-0.728088	2.928571
C	-4.614780	1.196203	0.193188
H	-5.702973	1.159535	0.299371
H	-4.253331	2.127094	0.637486
H	-4.366574	1.208993	-0.871410
C	-3.911384	-2.564799	1.053020
H	-4.726318	-2.694011	1.771231
H	-3.853606	-3.460112	0.428804
H	-2.974374	-2.476456	1.609526
C	-5.461510	-1.482965	-0.593343
H	-5.504654	-2.455193	-1.091916
H	-6.306496	-1.418077	0.100195
H	-5.571775	-0.708212	-1.353368
H	4.200737	-2.760608	-0.618816

***Int*-7**$_{2a\text{-}c}$

Pd	-0.641196	0.207568	-1.100165
C	1.241709	0.012792	-0.064086
C	0.913940	-0.505205	1.350633
C	0.646444	0.633064	2.351366
C	0.629334	2.421611	0.564323
C	1.089934	1.367137	-0.446905
H	1.818679	-1.017467	1.700449
H	1.544202	2.908240	0.944973
H	1.615520	1.000258	2.715421
C	2.204010	-0.885619	-0.794658
C	1.867196	-1.597152	-1.881521
H	2.591837	-2.207860	-2.411706
C	1.863398	1.943887	-1.617073
H	2.768831	2.454132	-1.255636
H	2.169193	1.171512	-2.322199
H	1.271081	2.693601	-2.146276
C	-0.213385	3.488725	-0.083319
H	-1.141722	3.126179	-0.527826
C	0.080364	4.787328	-0.127837
H	1.001507	5.175567	0.300671
H	-0.584476	5.510200	-0.591316
C	-0.191088	-1.594468	1.296693
H	0.166721	-2.401377	0.646667
H	-0.301568	-2.024242	2.303031
H	0.855697	-1.561313	-2.273793
C	-0.119749	1.814892	1.760744
C	3.575372	-0.957057	-0.216560
C	4.352262	-2.123072	-0.322576
C	4.126074	0.142978	0.462452
C	5.642134	-2.175976	0.199596
H	3.927938	-3.001150	-0.798245
C	5.415921	0.090356	0.987413
H	3.538408	1.047015	0.573380
C	6.182652	-1.067593	0.854729
H	6.221863	-3.089891	0.107705
H	5.822004	0.956709	1.501433
H	7.186187	-1.110876	1.267072
H	0.114299	0.236016	3.222806
H	-0.257414	2.592524	2.520466
H	-1.117088	1.496058	1.446672
C	-3.843815	-0.578889	0.961362
C	-3.545374	-1.065824	-0.508079
B	-1.616262	-1.147958	0.777187
O	-2.523530	-0.435595	1.536941
O	-2.221818	-1.660177	-0.383503
C	-4.498547	-2.127437	-1.045037
H	-4.192872	-2.416863	-2.054080
H	-5.521173	-1.740351	-1.095835
H	-4.493809	-3.021656	-0.421065
C	-3.424370	0.078107	-1.522656
H	-4.382143	0.569593	-1.715970
H	-3.053670	-0.324088	-2.470229
H	-2.732555	0.860067	-1.156554
C	-4.571749	-1.628505	1.806734
H	-5.606271	-1.767535	1.480252
H	-4.577969	-1.296266	2.847721
H	-4.058014	-2.592405	1.763441
C	-4.560353	0.764159	1.054712
H	-4.704876	1.026693	2.105847
H	-5.544146	0.716787	0.576808
H	-3.978981	1.560585	0.587365

***Int*-7**$_{2a\text{-}t}$

Pd	-0.854020	-0.184477	-1.609514
C	1.813825	-0.262321	-0.191726
C	1.168695	-0.919391	1.033898
C	2.234046	-1.507938	1.966328
C	3.962970	-1.559210	0.150817
C	3.086844	-0.529389	-0.565083
H	0.644809	-0.129009	1.582151
H	2.800912	-0.695989	2.439162
C	0.996486	0.778790	-0.904150
C	0.867483	0.791986	-2.310757
H	0.644964	1.724394	-2.823741
C	3.807382	0.165723	-1.696648
H	4.851952	0.336479	-1.420863
H	3.356949	1.122254	-1.960852
H	3.818189	-0.458434	-2.600267
C	5.134214	-0.877867	0.825778
H	4.877994	-0.176863	1.620075
C	6.416313	-1.082557	0.525767
H	6.710952	-1.768672	-0.265123
H	7.217400	-0.577214	1.057068

C	0.101303	-2.000790	0.656259
H	0.107370	-2.771735	1.441834
H	0.368011	-2.503662	-0.277400
H	1.392301	0.048105	-2.902416
C	3.180040	-2.403843	1.171835
C	0.699103	2.023750	-0.127083
C	-0.296599	2.932448	-0.529032
C	1.457627	2.346174	1.009596
C	-0.530120	4.105819	0.180389
H	-0.909394	2.690920	-1.391861
C	1.219199	3.518390	1.727627
H	2.255096	1.678984	1.317968
C	0.222562	4.403344	1.320140
H	-1.311040	4.785209	-0.149119
H	1.819507	3.739997	2.605358
H	0.032115	5.313268	1.881226
H	1.749538	-2.063554	2.776310
H	3.881919	-2.931529	1.825680
H	2.597178	-3.167480	0.643777
C	-3.583236	-1.163864	0.014590
C	-3.229685	-0.268752	1.259750
B	-1.355521	-1.424389	0.601949
O	-2.258087	-1.574509	-0.465673
O	-1.942349	-0.799823	1.671843
C	-4.199322	-0.399606	2.428784
H	-3.864731	0.239962	3.249578
H	-5.203963	-0.078615	2.135513
H	-4.251539	-1.424933	2.797792
C	-3.013759	1.204444	0.910804
H	-3.955491	1.699236	0.656272
H	-2.572930	1.712542	1.771571
H	-2.318651	1.311538	0.074842
C	-4.305674	-2.460652	0.381114
H	-5.332188	-2.266615	0.703404
H	-4.334573	-3.111881	-0.495951
H	-3.783649	-2.989742	1.182541
C	-4.301151	-0.443672	-1.116646
H	-4.493897	-1.140404	-1.937026
H	-5.261105	-0.046780	-0.772289
H	-3.698188	0.382280	-1.501937
H	4.378909	-2.225617	-0.618815

***cis*-2a**

C	-2.057531	0.210654	0.318518
C	-0.618274	0.513059	-0.102553
C	-0.450202	1.941612	-0.635094
C	-2.943277	2.307406	-0.756013
C	-3.109017	0.998179	0.010013
H	-0.375115	2.634966	0.213026
H	-0.373501	-0.179847	-0.920458
H	-3.769005	2.373203	-1.477477
H	0.497308	2.008824	-1.178447
C	-2.226160	-1.086925	1.038535
C	-2.648868	-1.139307	2.311329
H	-2.874976	-0.231887	2.860812
H	-2.794861	-2.083261	2.827603
C	-4.541398	0.685202	0.360516
H	-5.159448	0.691580	-0.546863
H	-4.653939	-0.279910	0.852094
H	-4.952715	1.460629	1.017730
C	-3.089315	3.476974	0.191080
H	-2.312144	3.572409	0.948665
C	-4.093373	4.353258	0.170373
H	-4.893218	4.282146	-0.563236
H	-4.154037	5.172788	0.880414
C	0.395483	0.241013	1.028966
H	0.199888	0.904361	1.881069
H	0.255755	-0.775595	1.419187
C	-1.614896	2.350165	-1.530353
H	-1.680212	1.655313	-2.377577
H	-1.460702	3.351969	-1.946086
C	-1.860425	-2.316589	0.286695
C	-1.271580	-3.420116	0.924457
C	-2.096797	-2.392267	-1.095089
C	-0.950832	-4.570929	0.207750
H	-1.046328	-3.360784	1.984706
C	-1.777342	-3.543103	-1.812477
H	-2.543745	-1.539782	-1.597072
C	-1.204306	-4.638186	-1.163735
H	-0.490889	-5.412377	0.717868
H	-1.974678	-3.584902	-2.879701
H	-0.948446	-5.532906	-1.723333
B	1.892664	0.357348	0.583787
O	2.923233	0.683511	1.432362
O	2.320055	0.108553	-0.700303
C	4.166375	0.419050	0.726753
C	3.718328	0.494520	-0.777463
C	5.197116	1.460035	1.146070
H	5.430062	1.339783	2.207506
H	6.124715	1.336346	0.577830
H	4.826071	2.474618	0.994305
C	4.621894	-0.981271	1.148399
H	5.592508	-1.237908	0.714582
H	4.710071	-1.010464	2.237385
H	3.893742	-1.738818	0.846126
C	3.750856	1.917177	-1.344292
H	4.775406	2.267641	-1.497826
H	3.234520	1.924989	-2.307753
H	3.238770	2.616974	-0.678390
C	4.439230	-0.465735	-1.715682
H	4.045486	-0.353364	-2.729467
H	5.512165	-0.249502	-1.741076
H	4.297744	-1.503753	-1.411766

***trans*-2a**

C	-1.874562	-0.381458	0.661092
C	-0.492622	-0.553855	0.041444
C	-0.427154	-1.775915	-0.885098
C	-2.596593	-2.704102	0.013333
C	-2.824823	-1.338016	0.656401
H	-0.923849	-1.537136	-1.833039
H	-2.983209	-3.463071	0.708149
H	0.616058	-1.994411	-1.133797
C	-2.087052	0.956253	1.289304
C	-2.263834	1.092868	2.613431
H	-2.254089	0.228081	3.268055
H	-2.440835	2.061311	3.071337
C	-4.203377	-1.162589	1.241291
H	-4.320491	-1.775586	2.145120
H	-4.414529	-0.127841	1.510376
H	-4.960340	-1.508731	0.529253
C	-3.404355	-2.816659	-1.260512
H	-3.107201	-2.147875	-2.068035
C	-4.429989	-3.647425	-1.445224
H	-4.760502	-4.323494	-0.659915
H	-4.974712	-3.686536	-2.383958
C	-1.104829	-2.985936	-0.243697
H	-0.618754	-3.215770	0.712029
H	-1.002902	-3.875546	-0.874390
C	-2.053085	2.131509	0.379566
C	-1.533266	3.368502	0.792016
C	-2.549667	2.018678	-0.929009
C	-1.532954	4.464439	-0.068023
H	-1.106960	3.459050	1.786129
C	-2.549864	3.114279	-1.789639
H	-2.943359	1.062116	-1.258575
C	-2.043603	4.342834	-1.361987
H	-1.121706	5.412134	0.267521
H	-2.945485	3.009098	-2.795686
H	-2.037868	5.195834	-2.033949
C	0.597871	-0.597872	1.138513
H	0.421513	0.213740	1.857974
H	0.512353	-1.517239	1.731369
B	2.057699	-0.437258	0.593464
O	2.342845	0.005804	-0.677057
O	3.187825	-0.695139	1.332058
C	3.768427	0.279481	-0.733956
C	4.324830	-0.618634	0.429173
C	4.278112	-0.075202	-2.125450
H	5.364173	0.049895	-2.183246

H	4.029267	-1.103131	-2.392966
H	3.819499	0.587573	-2.864202
C	3.936025	1.779140	-0.471624
H	4.980163	2.090968	-0.564766
H	3.340924	2.333307	-1.201794
H	3.580272	2.045471	0.527288
C	5.511411	-0.033048	1.184891
H	6.366086	0.105754	0.514883
H	5.262663	0.927293	1.638635
H	5.812559	-0.717009	1.982940
C	4.630908	-2.053214	-0.012058
H	5.521565	-2.099977	-0.645069
H	4.803827	-2.666166	0.876108
H	3.789577	-2.483255	-0.562309
H	-0.293291	0.337781	-0.563865

Bpin-OPO(OPh)$_2$

O	1.178516	0.930941	1.734750
O	0.601982	-0.279723	-0.516550
P	1.180966	1.008387	0.261558
O	0.417326	2.261899	-0.416934
O	2.588571	1.239169	-0.478223
C	-0.971910	2.369666	-0.290785
C	-1.734014	2.265347	-1.450945
C	-1.556402	2.590651	0.954048
C	-3.121652	2.390495	-1.360994
H	-1.235622	2.091202	-2.398102
C	-2.943299	2.709270	1.029300
H	-0.929556	2.640963	1.836153
C	-3.728072	2.609586	-0.122174
H	-3.725653	2.316536	-2.260094
H	-3.411989	2.876310	1.994044
H	-4.807102	2.707462	-0.055074
C	3.679657	0.389300	-0.251936
C	4.286478	-0.165102	-1.374838
C	4.162968	0.157198	1.034390
C	5.410302	-0.973545	-1.205236
H	3.873722	0.042556	-2.355586
C	5.283707	-0.660518	1.186901
H	3.660611	0.600529	1.885438
C	5.910004	-1.224470	0.074163
H	5.891581	-1.409630	-2.075142
H	5.668585	-0.852069	2.183804
H	6.783363	-1.856004	0.202934
C	-2.424914	-1.685616	0.824695
C	-2.342643	-2.293024	-0.626941
B	-0.567768	-0.960299	-0.236068
O	-1.423593	-0.629797	0.772331
O	-0.959061	-2.018662	-1.003177
C	-2.578040	-3.794816	-0.709751
H	-2.483513	-4.124876	-1.747644
H	-3.585793	-4.044623	-0.363123
H	-1.853780	-4.347882	-0.110264
C	-3.223375	-1.551362	-1.635682
H	-4.283848	-1.759156	-1.468166
H	-2.960331	-1.882078	-2.643661
H	-3.067152	-0.471294	-1.576353
C	-1.973492	-2.660355	1.914105
H	-2.709352	-3.453645	2.072325
H	-1.847353	-2.108995	2.848862
H	-1.014288	-3.119402	1.660015
C	-3.764563	-1.056853	1.183626
H	-3.718913	-0.657332	2.200176
H	-4.563253	-1.804439	1.145497
H	-4.010406	-0.234156	0.511696

BQ

C	-0.631180	-0.396257	-0.001903
C	0.711273	-0.396297	0.002746
C	1.484240	0.872120	0.004506
C	0.711355	2.140583	0.000929
C	-0.631099	2.140623	-0.003720
C	-1.404065	0.872206	-0.005473
H	-1.219199	-1.308976	-0.003285
H	1.299234	-1.309051	0.005434
H	1.299374	3.053302	0.002305
H	-1.219059	3.053377	-0.006414
O	-2.629028	0.872243	-0.009700
O	2.709203	0.872084	0.008729

HO(C$_6$H$_4$)-OBpin

C	-4.333128	0.340541	0.029846
C	-3.260161	1.161938	0.385704
C	-1.960579	0.661601	0.399423
C	-1.727465	-0.672347	0.049246
C	-2.796476	-1.495962	-0.302155
C	-4.095920	-0.993158	-0.313520
H	-3.458441	2.194434	0.652498
H	-1.131519	1.301916	0.670111
H	-2.596384	-2.529207	-0.563943
H	-4.924162	-1.641895	-0.589876
O	-0.481809	-1.263791	0.056850
O	-5.586042	0.893894	0.037048
B	0.732919	-0.652974	0.023540
O	1.878253	-1.409117	-0.023304
O	0.965027	0.704496	0.030819
C	2.985133	-0.495978	0.194511
C	2.379148	0.881241	-0.264632
H	-6.225940	0.219979	-0.224625
C	4.179932	-0.973626	-0.621149
H	5.009429	-0.263591	-0.542901
H	4.521738	-1.939618	-0.240113
H	3.921585	-1.097557	-1.673669
C	2.488315	1.111787	-1.774159
H	1.861452	1.964046	-2.048117
H	3.517548	1.326802	-2.074486
H	2.138477	0.238442	-2.331178
C	2.886058	2.097219	0.499471
H	3.965880	2.214192	0.363734
H	2.396827	2.999529	0.122631
H	2.673998	2.016387	1.566475
C	3.308617	-0.537049	1.691078
H	3.515313	-1.570925	1.978314
H	4.184113	0.072482	1.931759
H	2.462877	-0.181947	2.286374

8. References

(1). Miró, J.; Gensch, T.; Ellwart, M.; Han, S.-J.; Lin, H.-H.; Sigman, M. S.; Toste, F. D. Enantioselective Allenoate-Claisen Rearrangement Using Chiral Phosphate Catalysts. *J. Am. Chem. Soc.* **2020**, *142*, 6390-6399.

(2). Guo, Q.-S.; Du, D.-M.; Xu, J. The Development of Double Axially Chiral Phosphoric Acids and Their Catalytic Transfer Hydrogenation of Quinolines. *Agnew. Chem. Int. Ed.* **2008**, *47*, 759-762.

(3). Austad, B. C.; Hague, A. B.; White, P.; Peluso, S.; Nair, S. J.; Depew, K. M.; Grogan, M. J.; Charette, A. B.; Yu, L.-C.; Lory, C. D.; *et al.* Development of a Multi Kilogram-Scale, Tandem Cyclopropanation Ring-Expansion Reaction en Route to Hedgehog Antagonist IPI-926. *Org. Proc. Res. & Dev.* **2016**, *20*, 786-798.

(4). Nifant'ev, I. E.; Tavtorkin, A. N.; Korchagina, S. y. A.; Gavrilenko, I. F.; Glebova, N. N.; Kostitsyna, N. N.; Yakovlev, V. A.; Bondarenko, G. N.; Filatova, M. P. Neodymium Tris-diarylphosphates: Systematic Study of the Structure–Reactivity Relationship in Butadiene and Isoprene Polymerisation. *Appl. Catal. A: Gen.* **2014**, *478*, 219-227.

(5). Peppard, D. F.; Ferraro, J. R.; Mason, G. W. Preparation, Physical Properties and Infra-red Studies of Several Alkyl and Aryl Phosphoric acids. *J. Inorg. Nucl. Chem.* **1961**, *16*, 246-256.

(6). Kim, S.; Park, H.; Fuß, F.; Lee, Y. Synthesis of ROS-responsive Poly(thioacetal)s with Narrow Molecular Weight Distributions via Lactone Ring-Opening Polymerization. *Polym. Chem.* **2023**, *14*, 2610-2616.

(7). Herrmann, J. L.; Kieczykowski, G. R.; Schlessinger, R. H. Deconjugative Alkylation of the Enolate Anion Derived from Ethyl Crotonate. *Tetrahedron Lett.* **1973**, *14*, 2433-2436.

(8). Chen, C.; Shen, X.; Chen, J.; Hong, X.; Lu, Z. Iron-Catalyzed Hydroboration of Vinylcyclopropanes. *Org. Lett.* **2017**, *19*, 5422-5425.

(9). Makabe, H.; Kong, L. K.; Hirota, M. Total Synthesis of (−)-Cassine. *Org. Lett.* **2003**, *5*, 27-29.

9. ^{1}H NMR and ^{13}C NMR Spectra

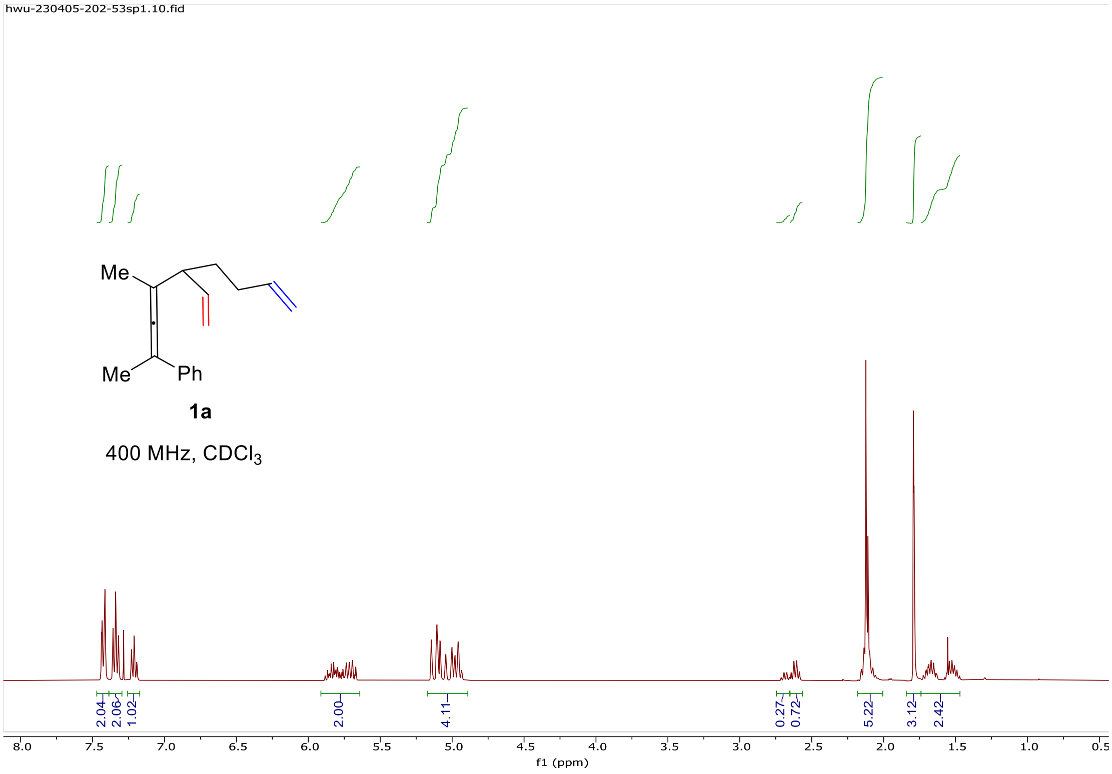

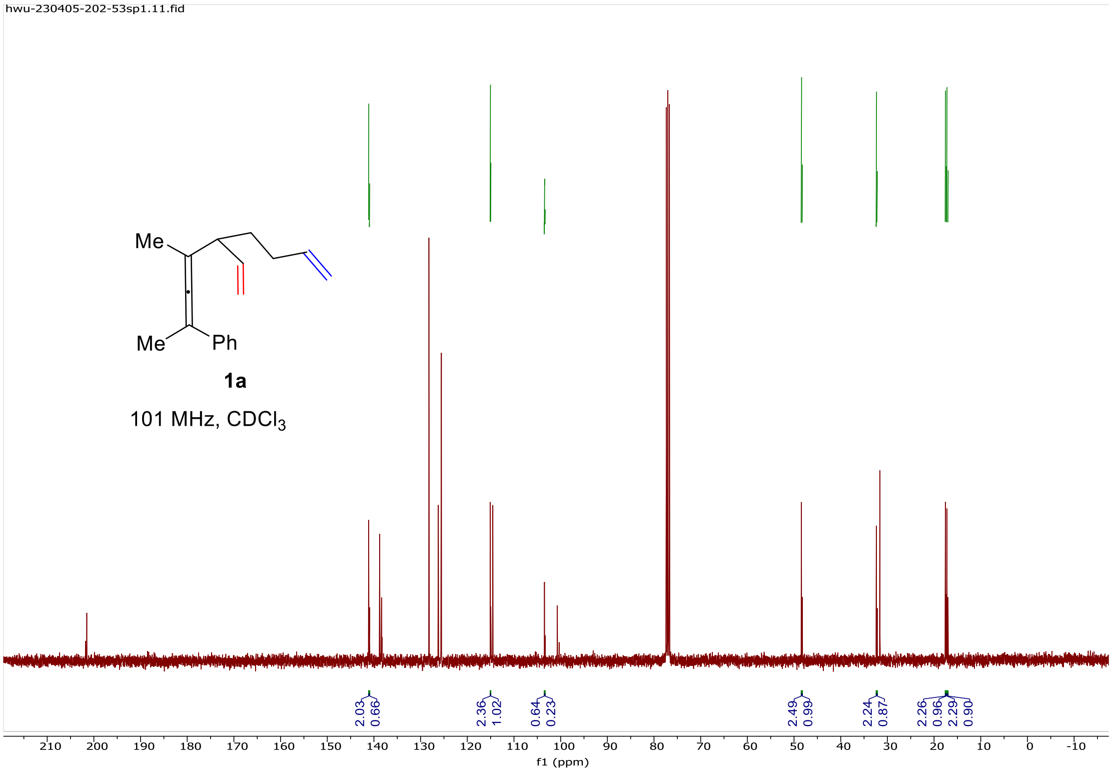

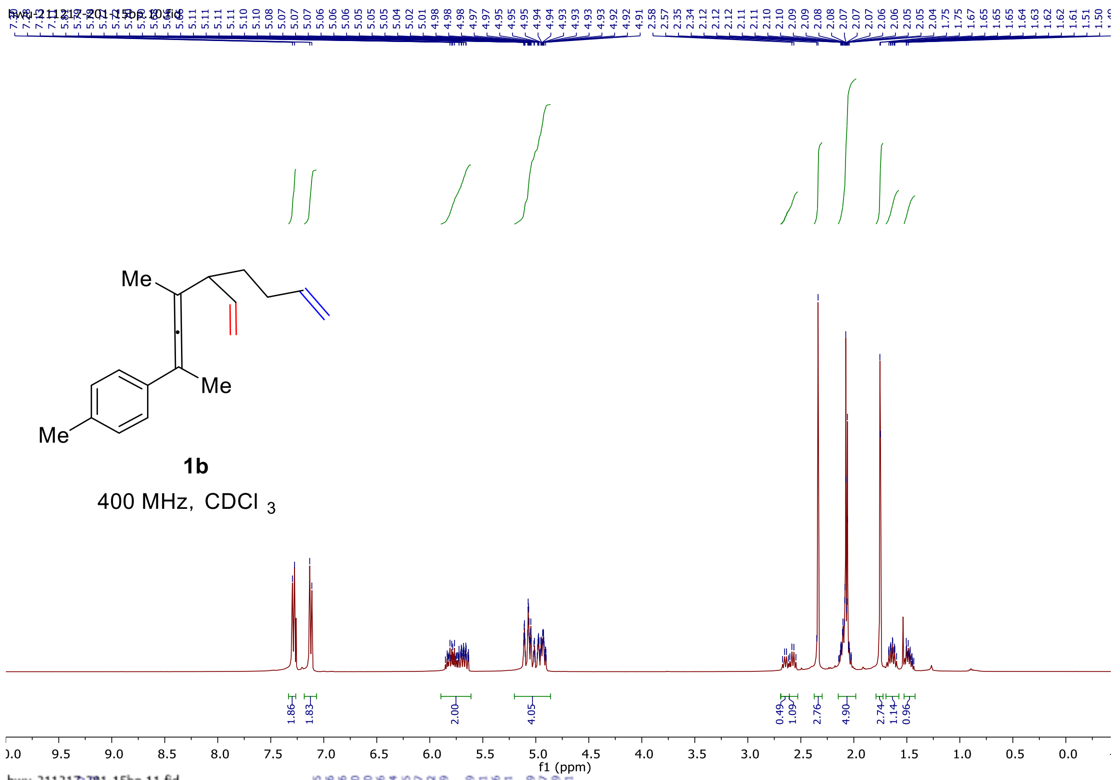
hwu-211217-201-15bp.10.fid
Me
Me
Me
1b
400 MHz, CDCl 3
f1 (ppm)

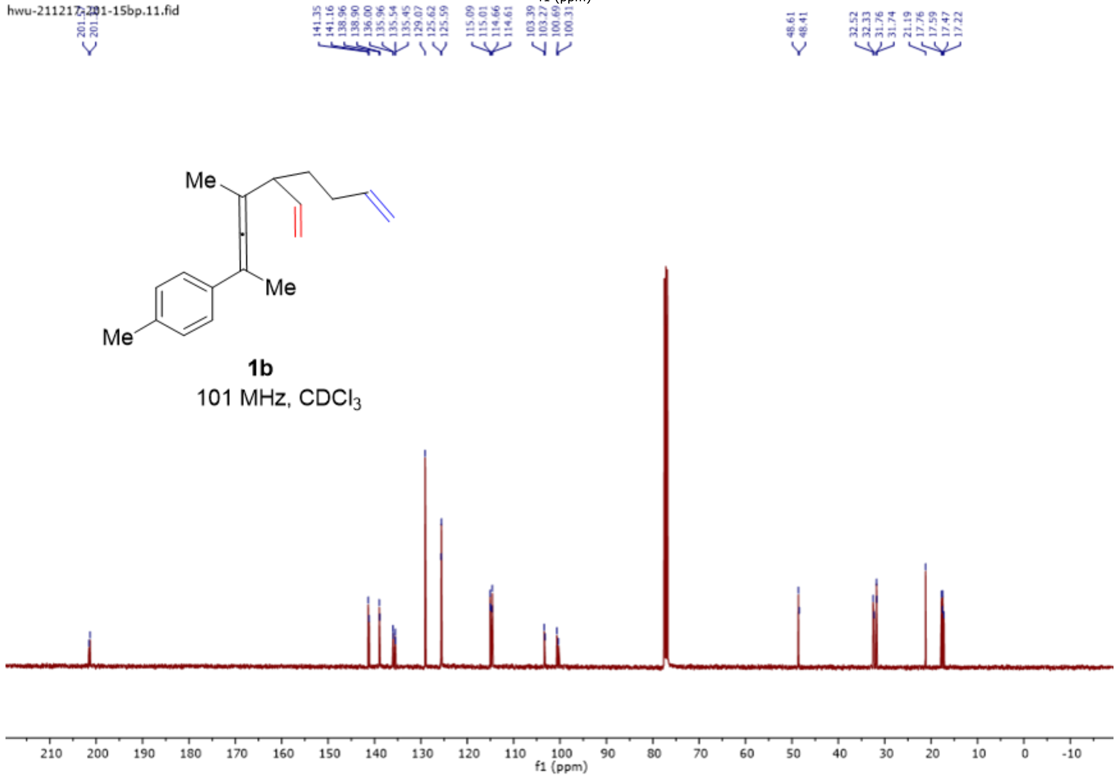
hwu-211217-201-15bp.11.fid
141.35
141.16
138.96
138.90
136.00
135.96
135.54
135.45
129.07
125.62
125.59
115.09
115.01
114.66
114.61
103.39
103.27
100.69
100.31
48.61
48.41
32.52
32.33
31.76
31.74
21.19
17.76
17.59
17.47
17.22
Me
Me
Me
1b
101 MHz, CDCl3
f1 (ppm)

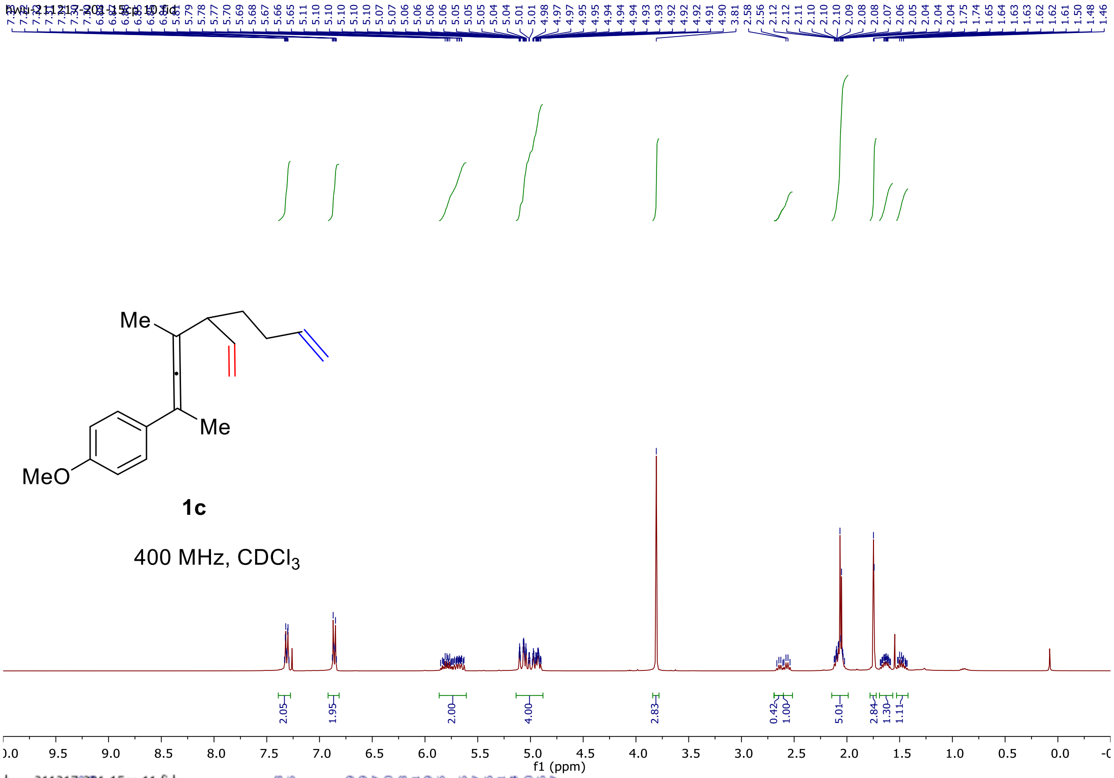
Me
Me
MeO
1c
400 MHz, CDCl3
f1 (ppm)

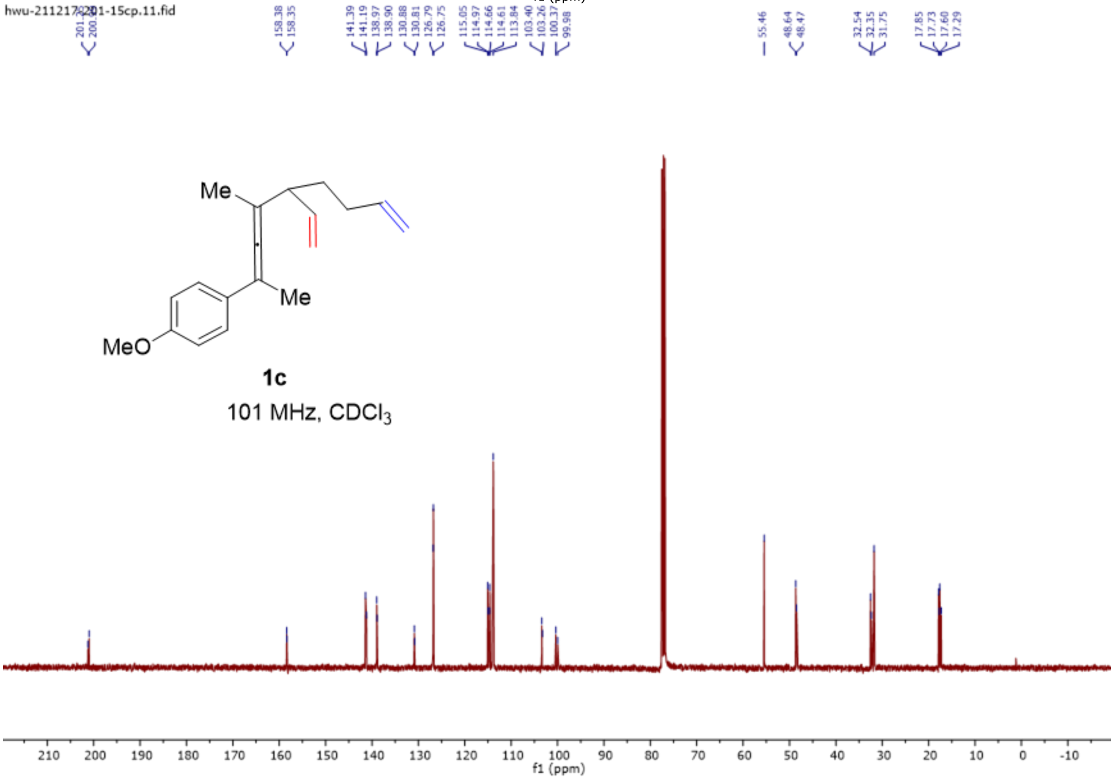
Me
Me
MeO
1c
101 MHz, CDCl3
f1 (ppm)

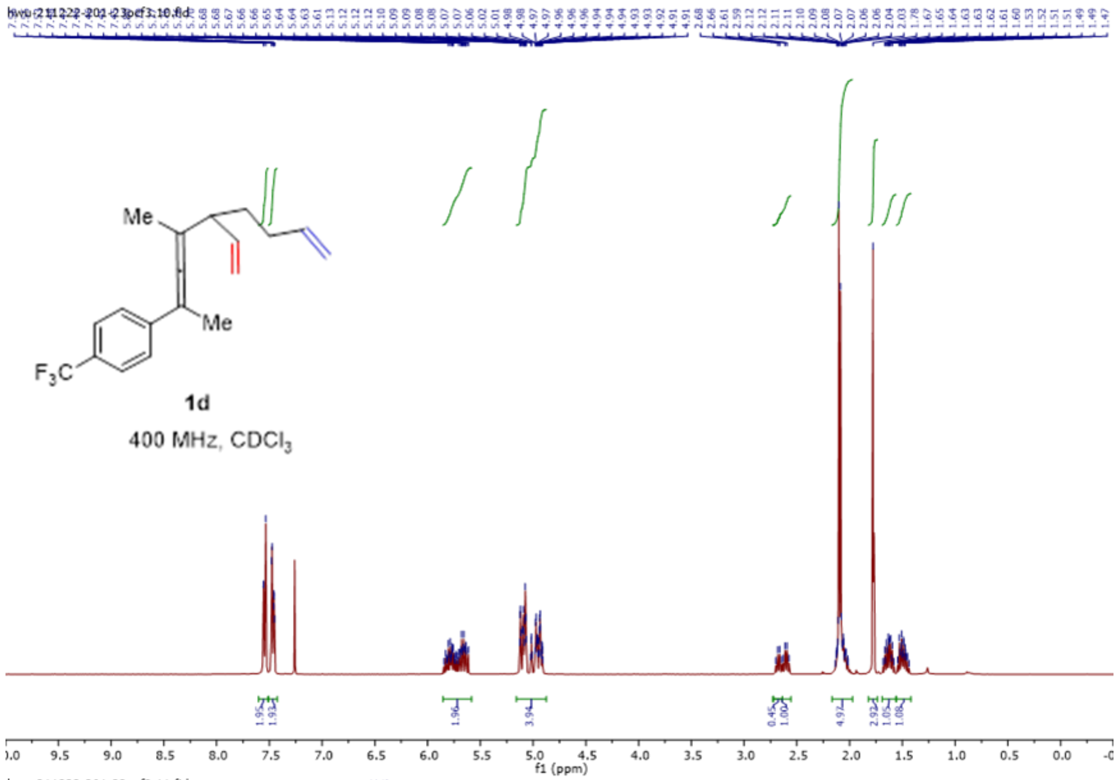
hwu-211222-201-23pcf3.10.fid
Me
Me
F3C
1d
400 MHz, CDCl3
f1 (ppm)

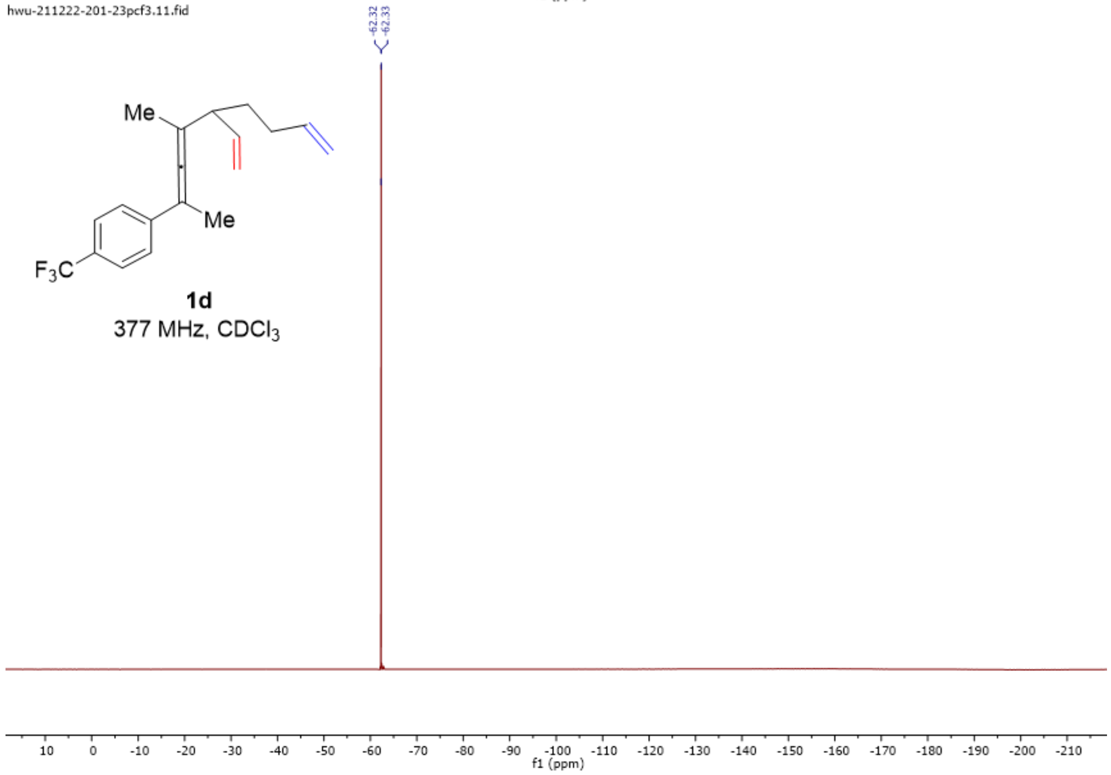
hwu-211222-201-23pcf3.11.fid
-62.32
-62.33
Me
Me
F3C
1d
377 MHz, CDCl3
f1 (ppm)

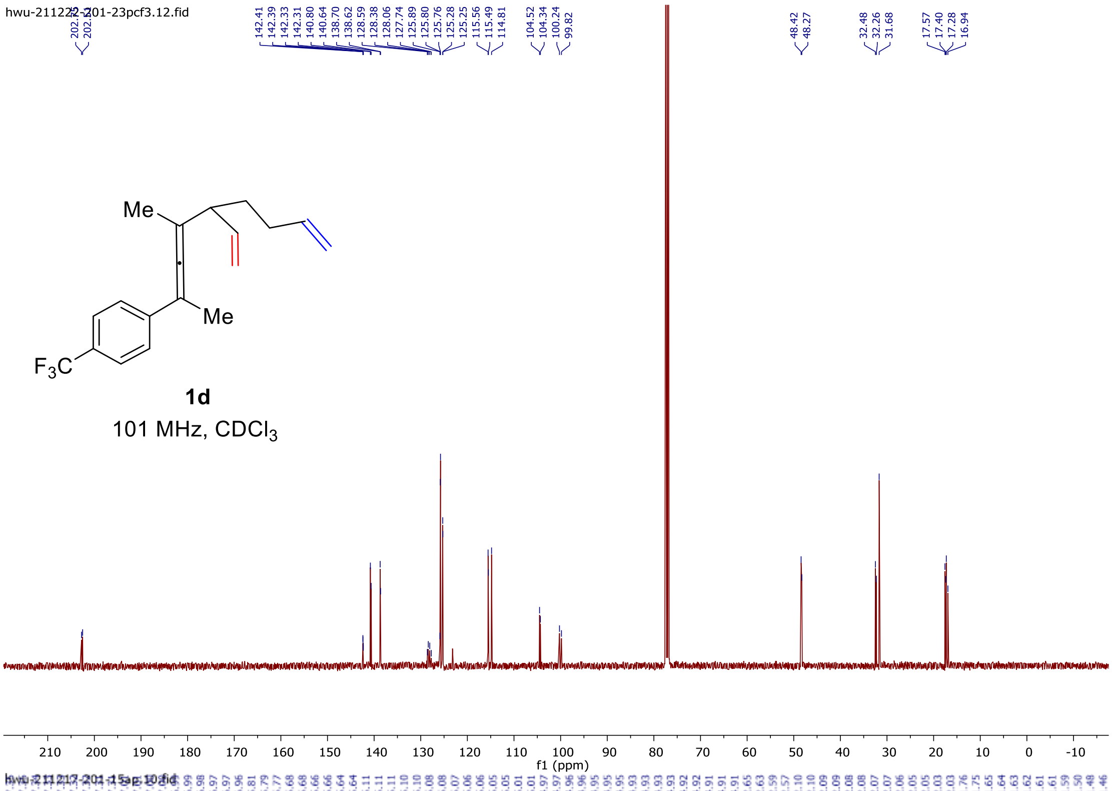

1d
101 MHz, $CDCl_3$

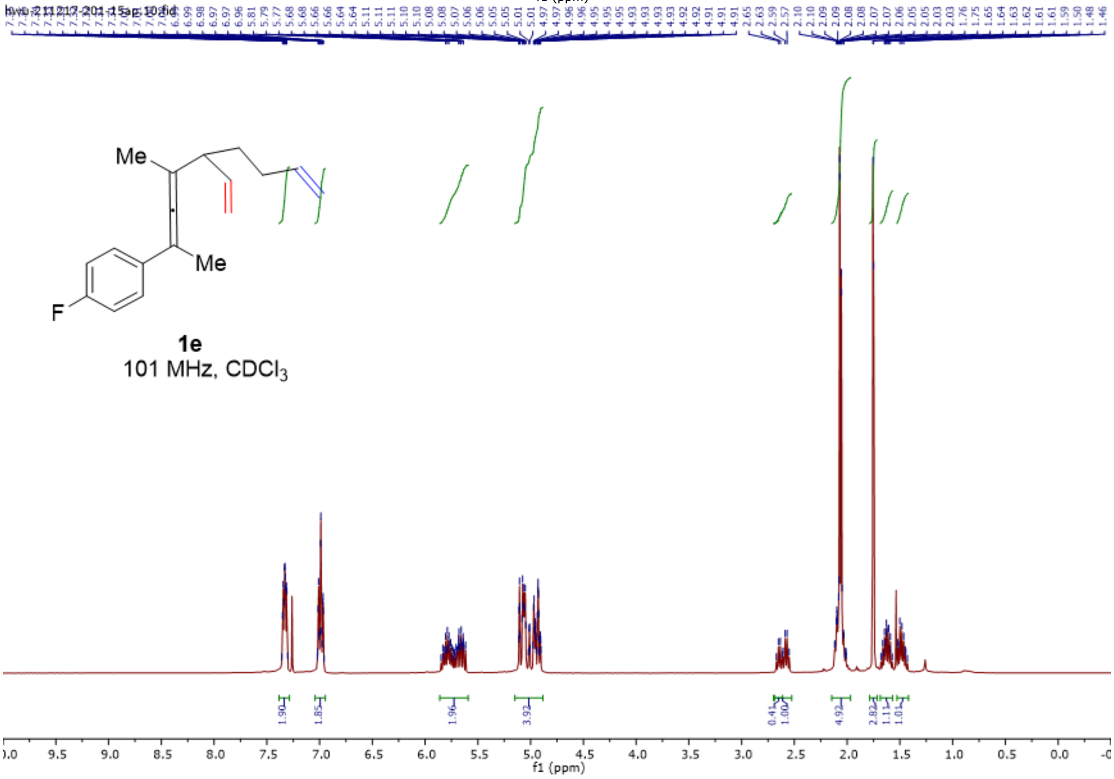

1e
101 MHz, $CDCl_3$

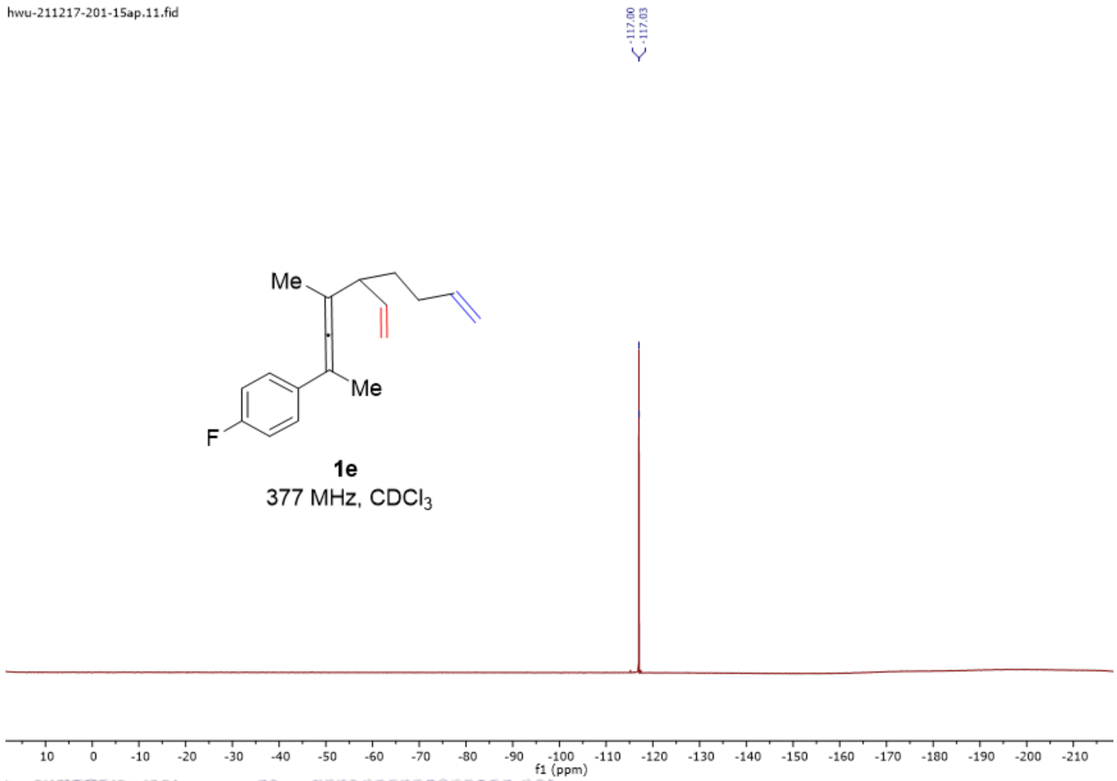
hwu-211217-201-15ap.11.fid
-117.00
-117.03
Me
Me
F
1e
377 MHz, CDCl3
f1 (ppm)

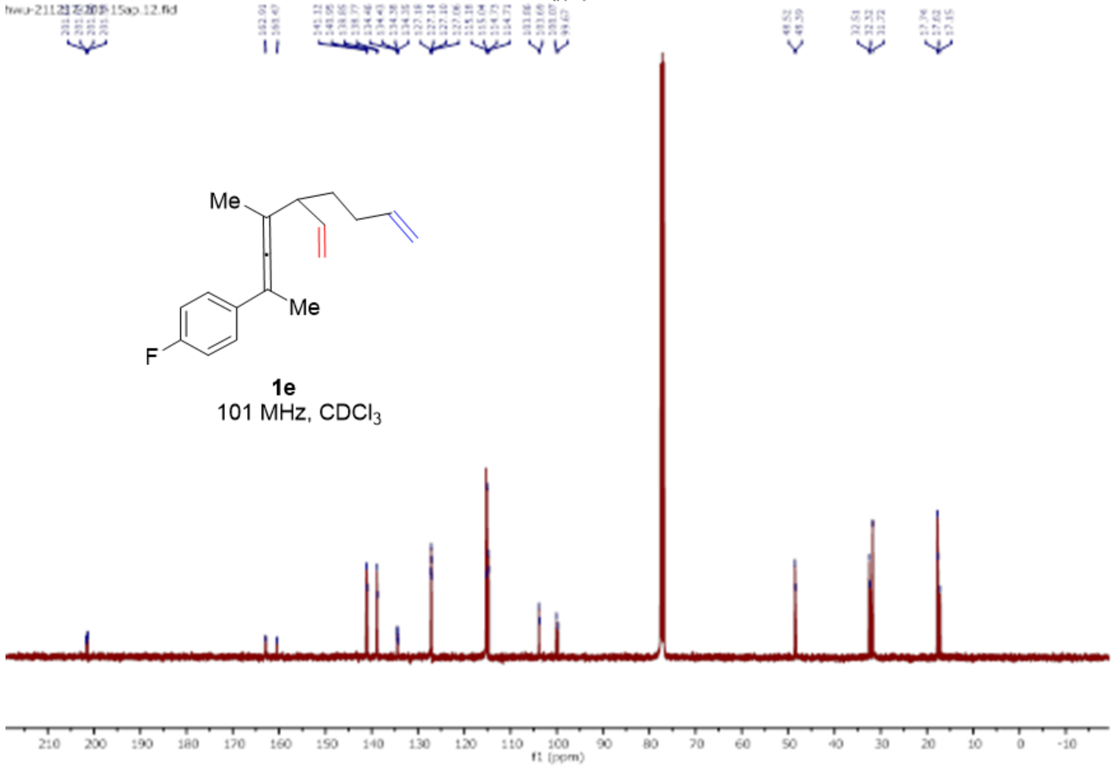
Me
Me
F
1e
101 MHz, CDCl3
f1 (ppm)

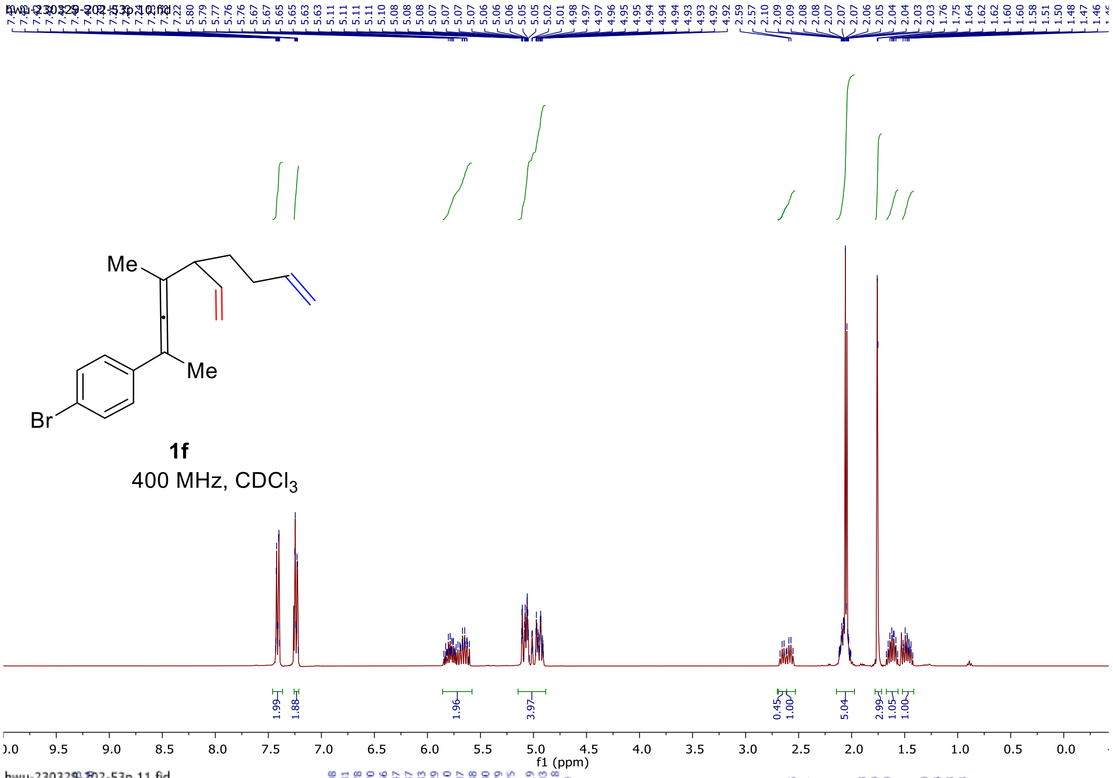

Me
Me
Br
1f
400 MHz, CDCl3
1.99
1.88
1.96
3.97
0.45
1.00
5.04
2.99
1.05
1.00
f1 (ppm)

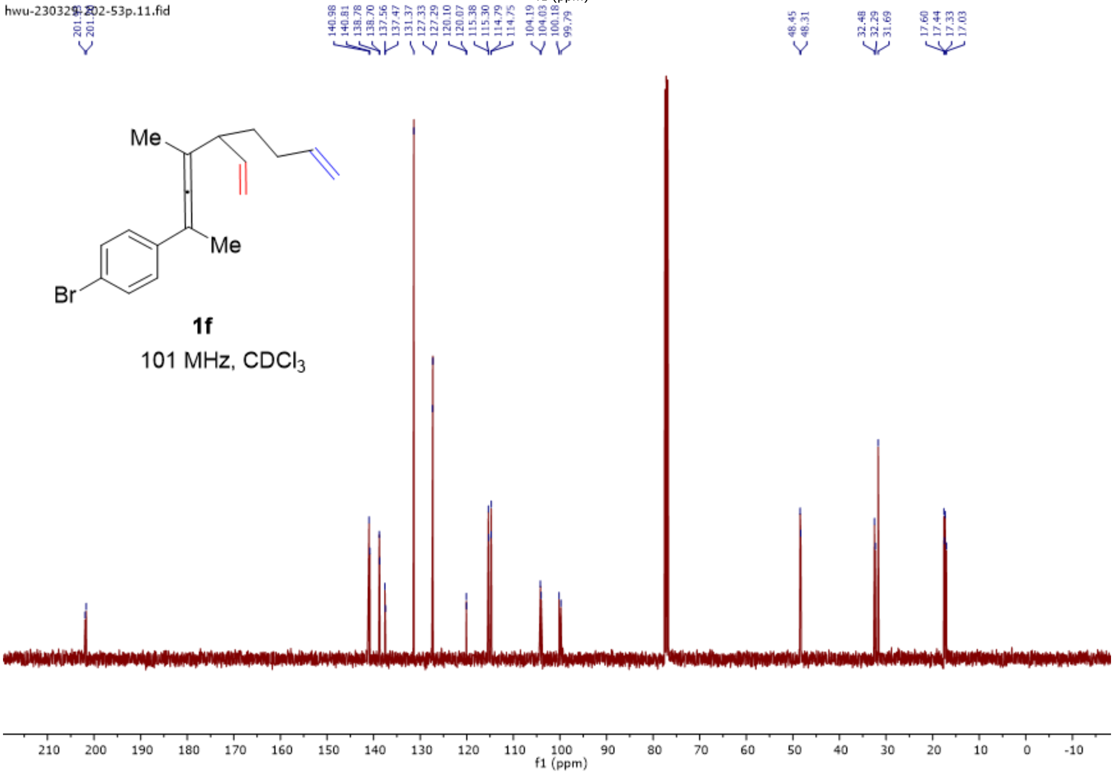

hwu-230329-202-53p.11.fid
140.98
140.81
138.78
138.70
137.56
137.47
131.37
127.33
127.29
120.10
120.07
115.38
115.30
114.79
114.75
104.19
104.03
100.18
99.79
48.45
48.31
32.48
32.29
31.69
17.60
17.44
17.33
17.03
Me
Me
Br
1f
101 MHz, CDCl3
f1 (ppm)

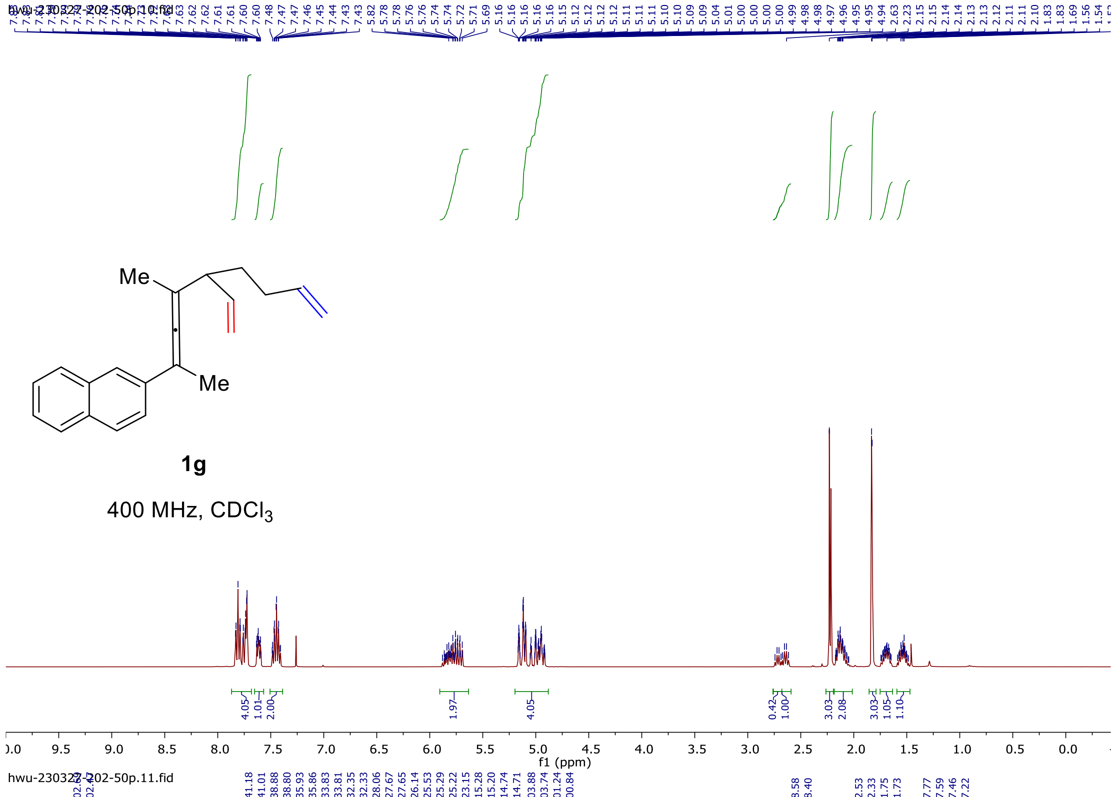

1g

400 MHz, $CDCl_3$

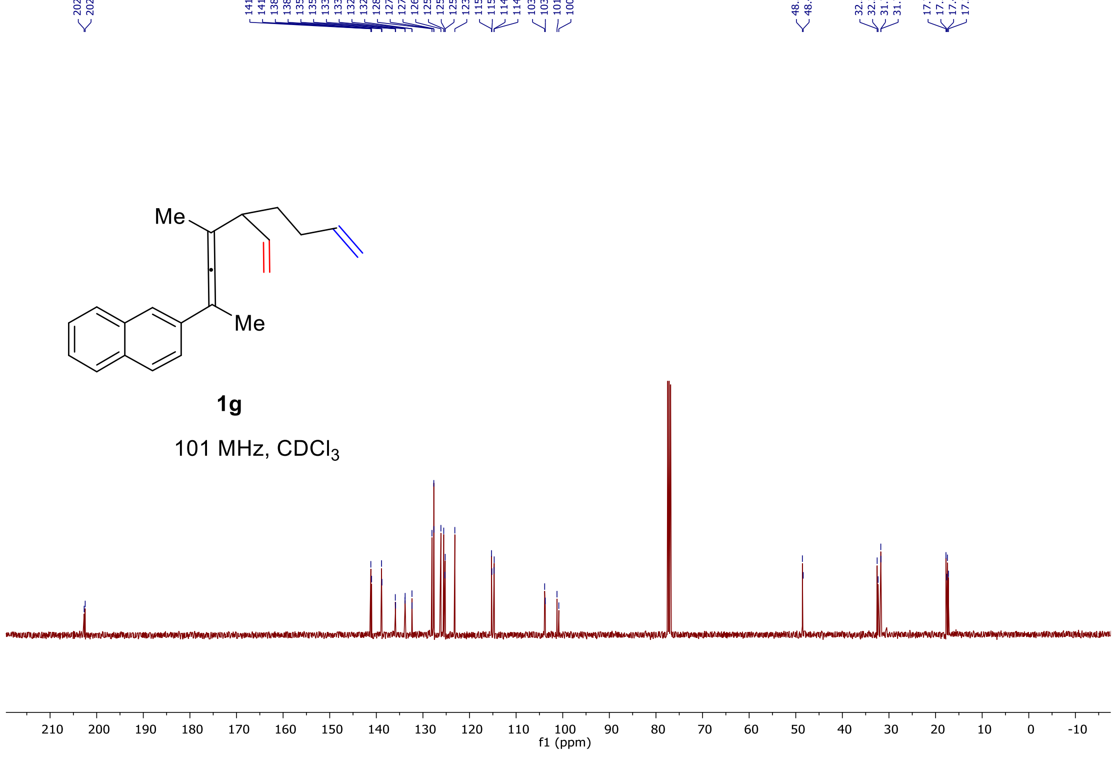

1g

101 MHz, $CDCl_3$

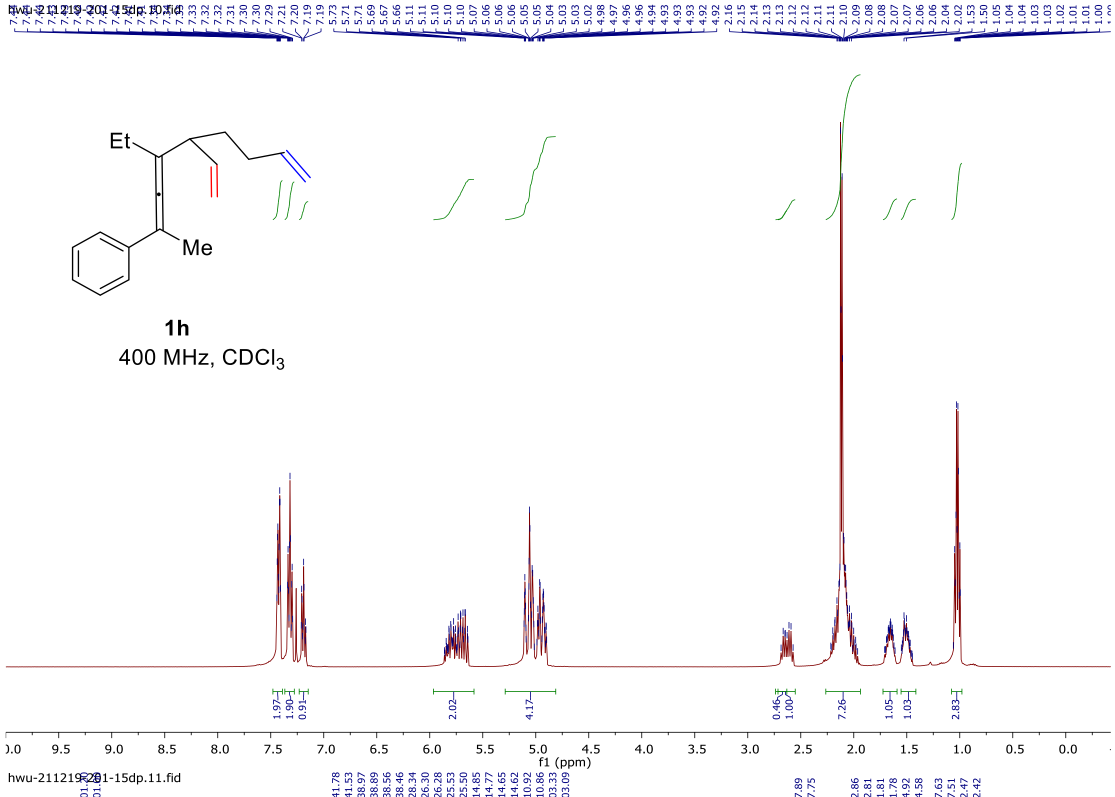
Et
Me
1h
400 MHz, CDCl$_3$
1.97
1.90
0.91
2.02
4.17
0.46
1.00
7.26
1.05
1.03
2.83
f1 (ppm)

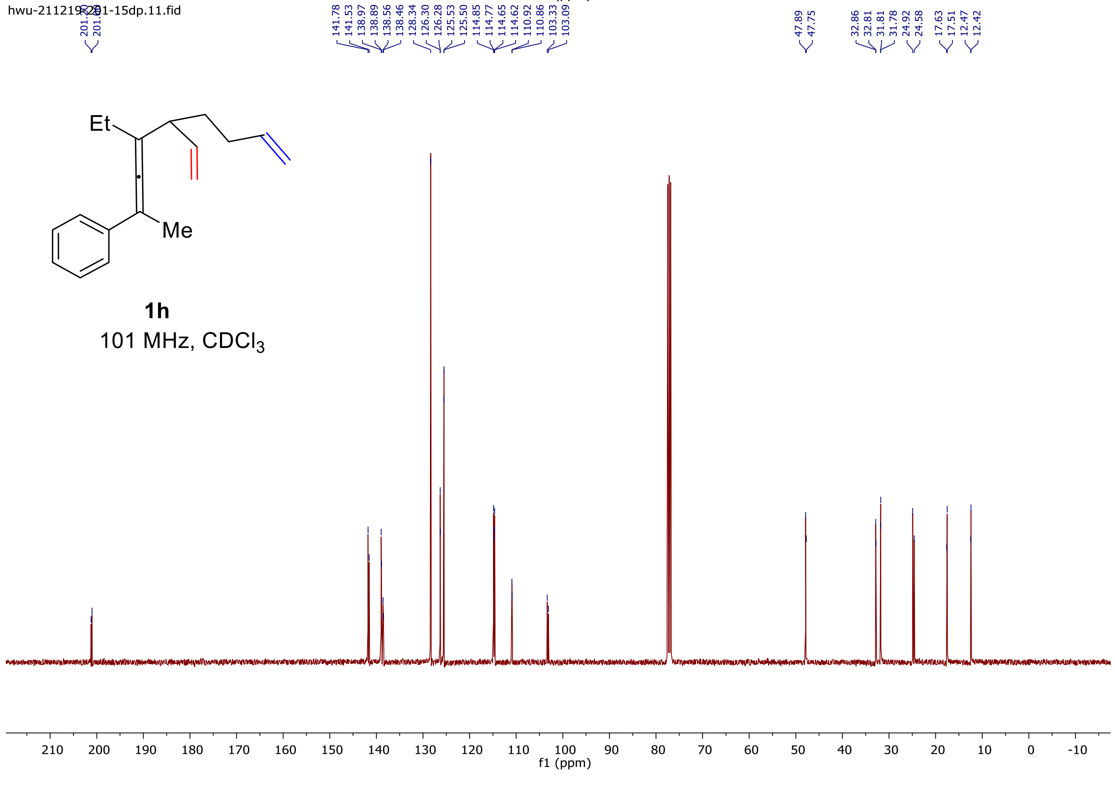
hwu-211219-201-15dp.11.fid
201.[illegible]
201.[illegible]
141.78
141.53
138.97
138.89
138.56
138.46
128.34
126.30
126.28
125.53
125.50
114.85
114.77
114.65
114.62
110.92
110.86
103.33
103.09
47.89
47.75
32.86
32.81
31.81
31.78
24.92
24.58
17.63
17.51
12.47
12.42
Et
Me
1h
101 MHz, CDCl$_3$
f1 (ppm)

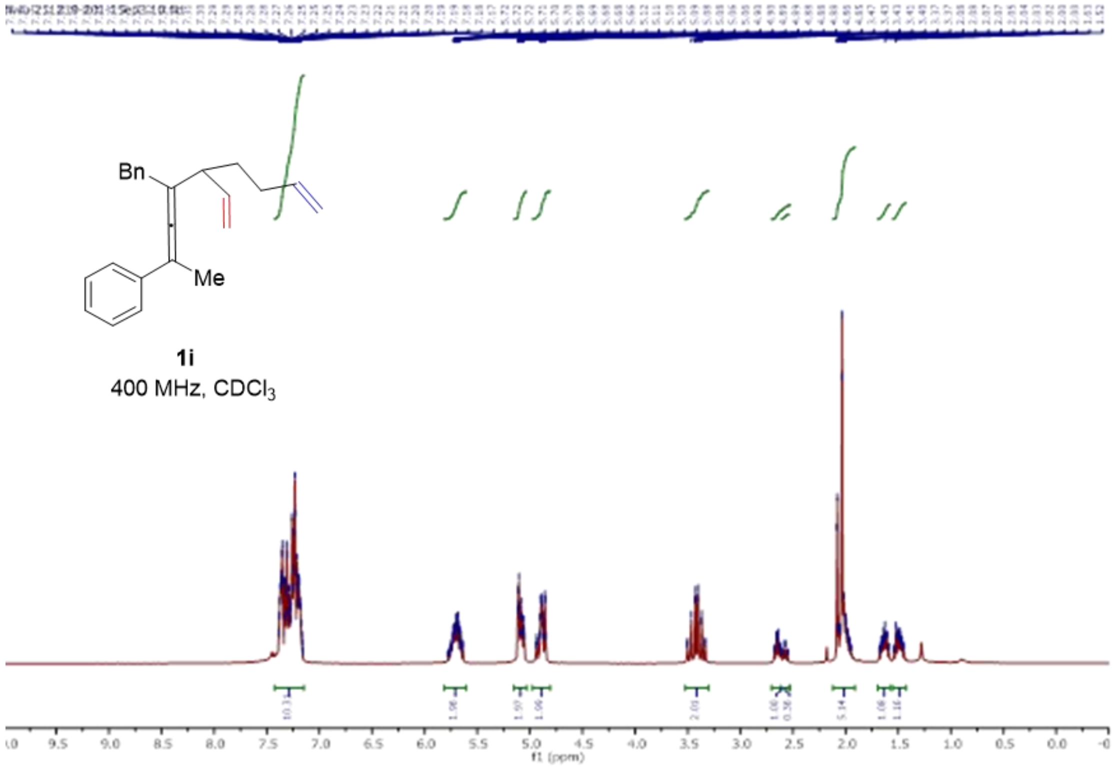
Bn
Me
1i
400 MHz, CDCl3
f1 (ppm)

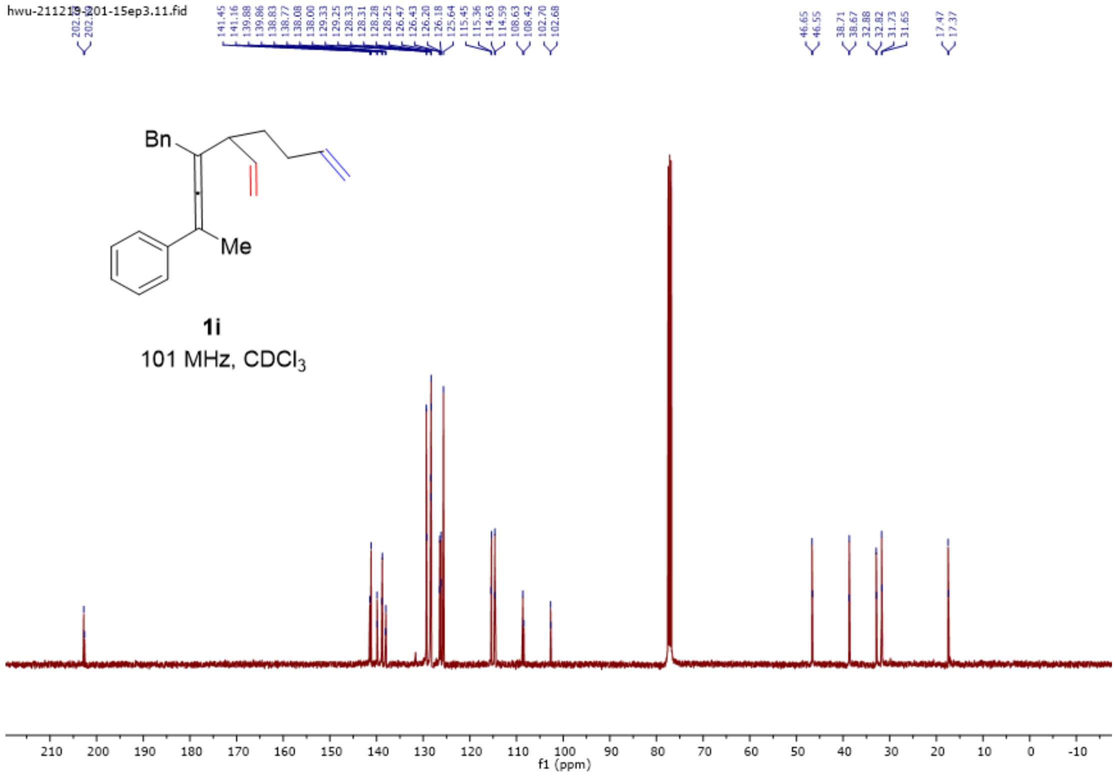
hwu-211219-201-15ep3.11.fid
Bn
Me
1i
101 MHz, CDCl3
f1 (ppm)

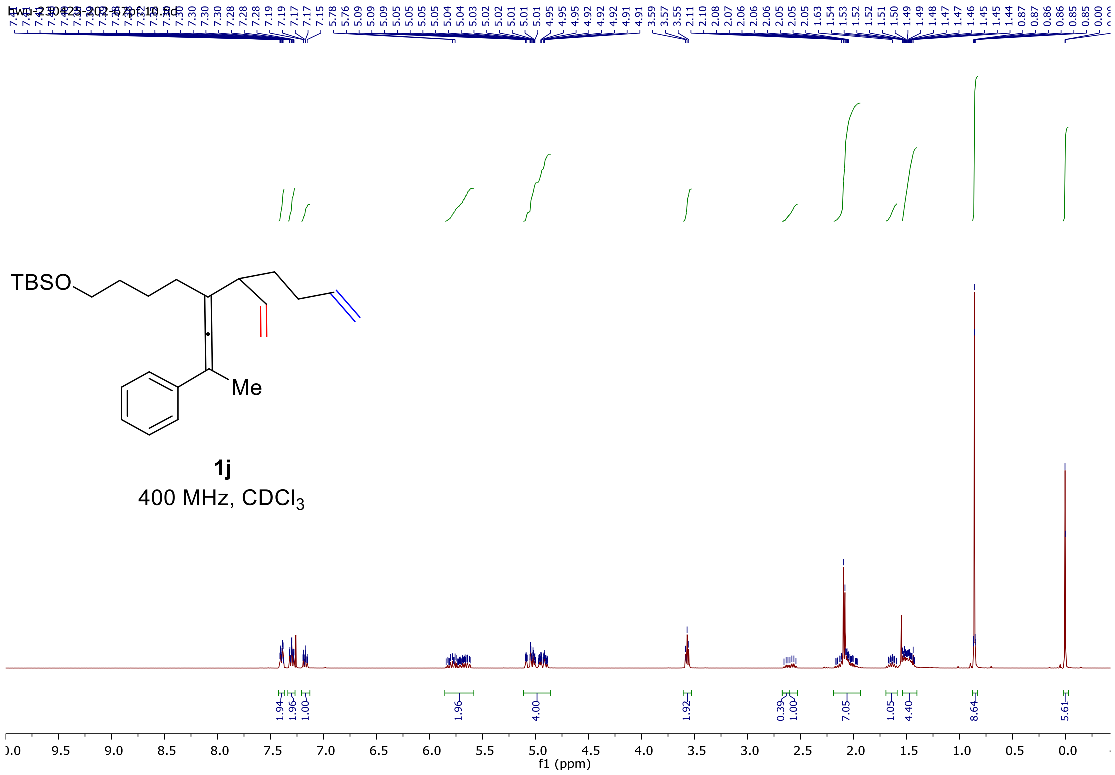
TBSO
Me
1j
400 MHz, CDCl3
f1 (ppm)

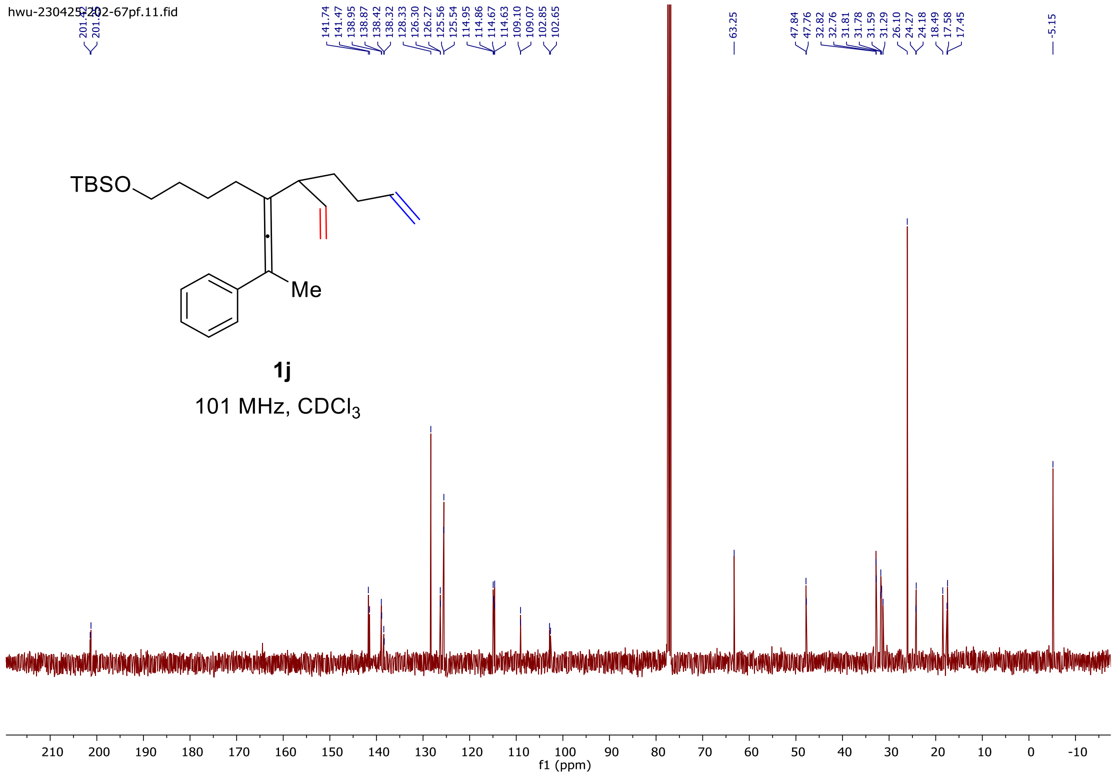
TBSO
Me
1j
101 MHz, CDCl3
f1 (ppm)

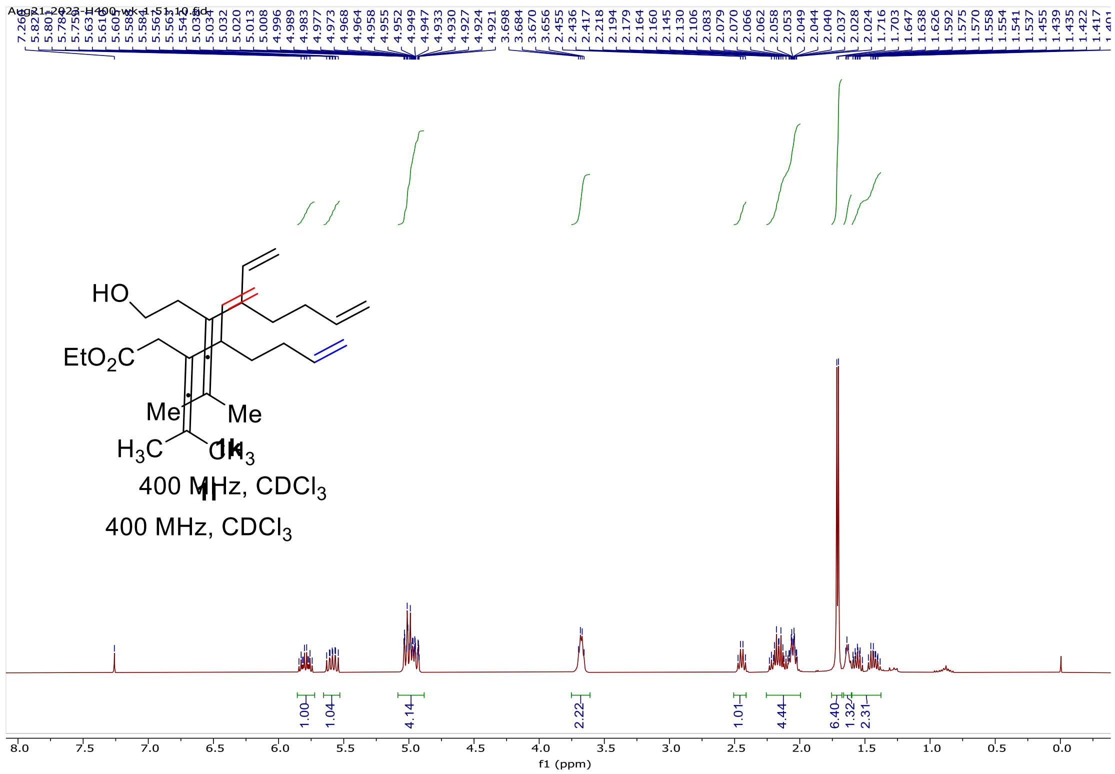

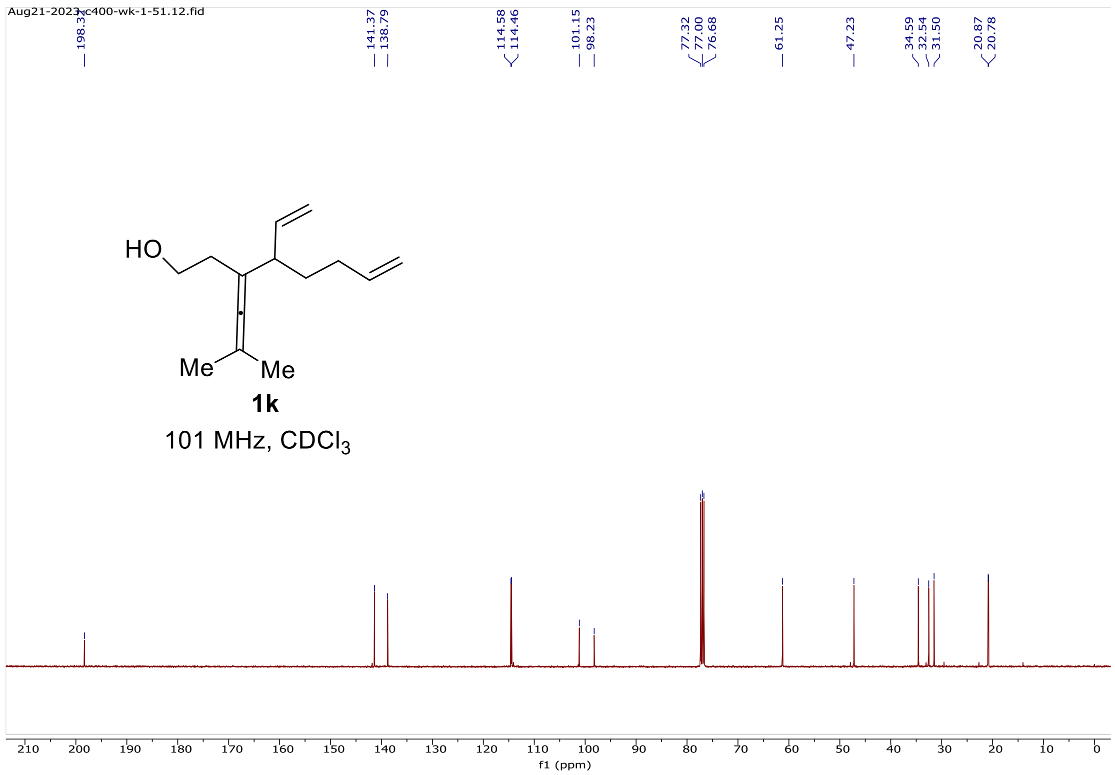

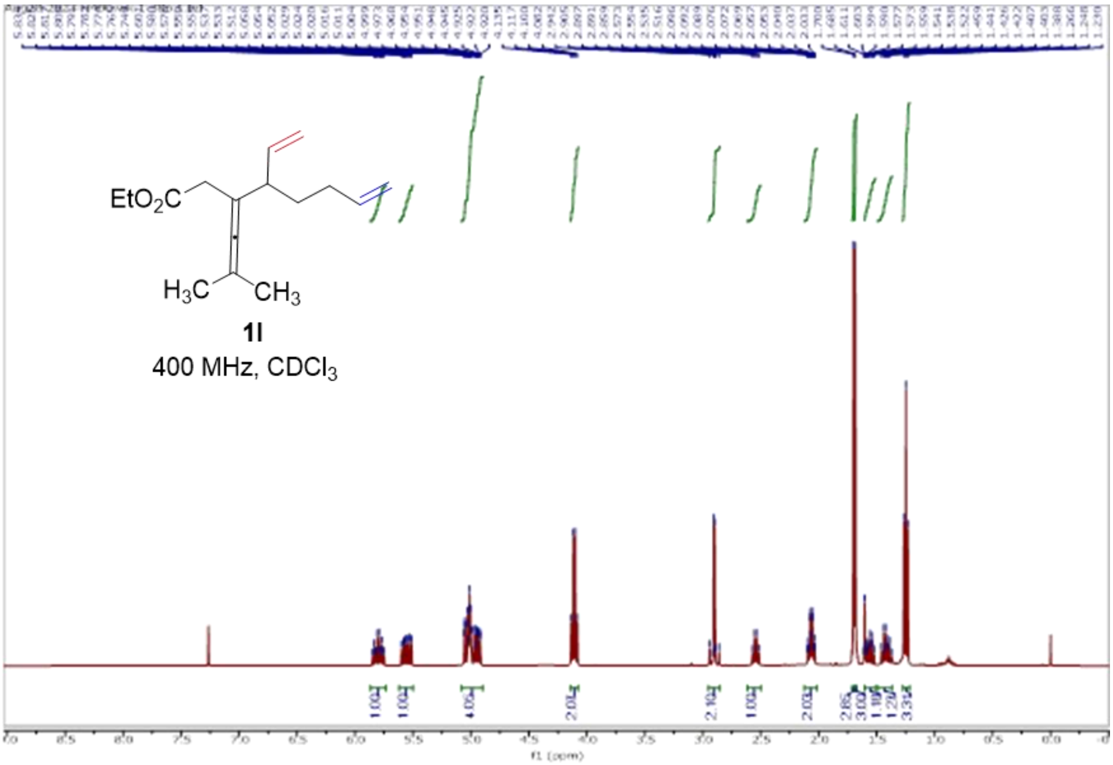
EtO2C
H3C
CH3
1l
400 MHz, CDCl3
f1 (ppm)

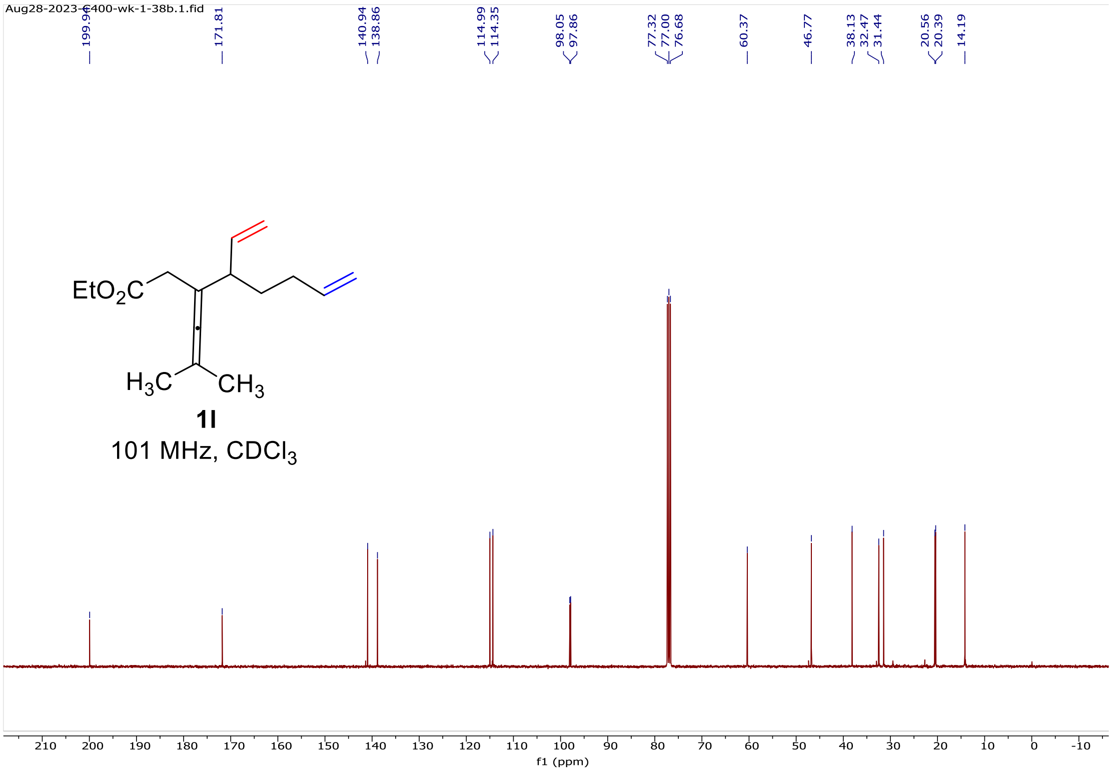
Aug28-2023-C400-wk-1-38b.1.fid
199.96
171.81
140.94
138.86
114.99
114.35
98.05
97.86
77.32
77.00
76.68
60.37
46.77
38.13
32.47
31.44
20.56
20.39
14.19
EtO2C
H3C
CH3
1l
101 MHz, CDCl3
f1 (ppm)

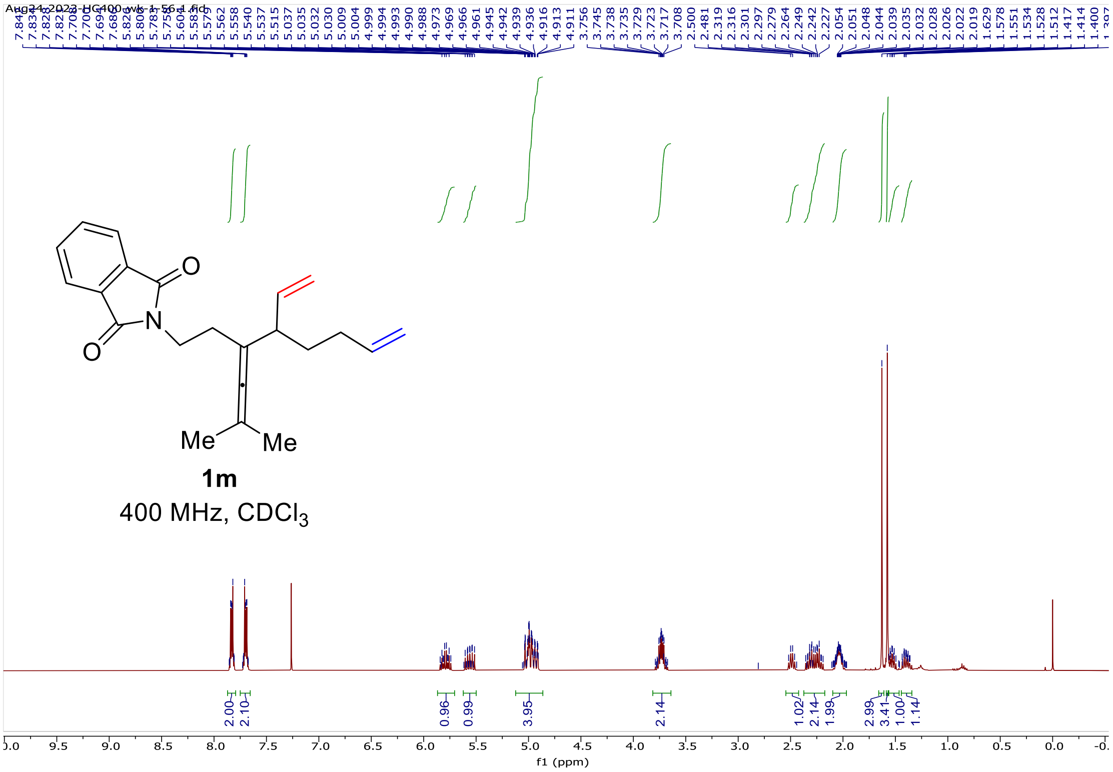
1m
400 MHz, CDCl3

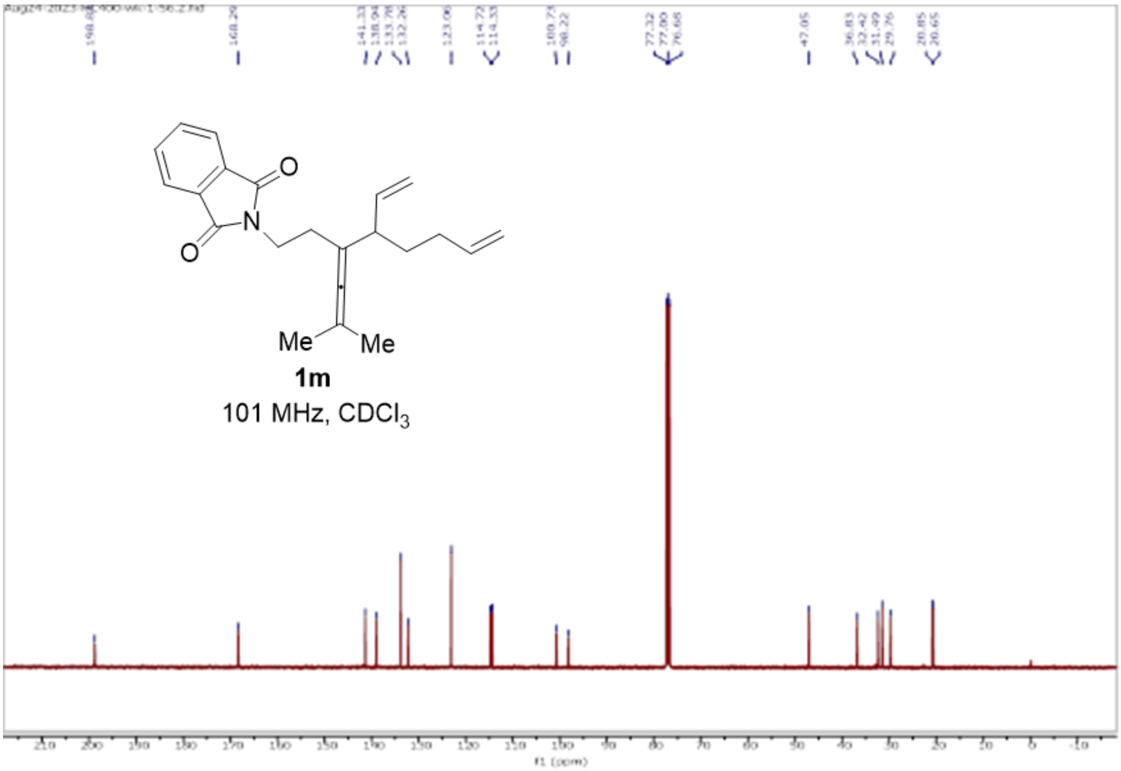
1m
101 MHz, CDCl3

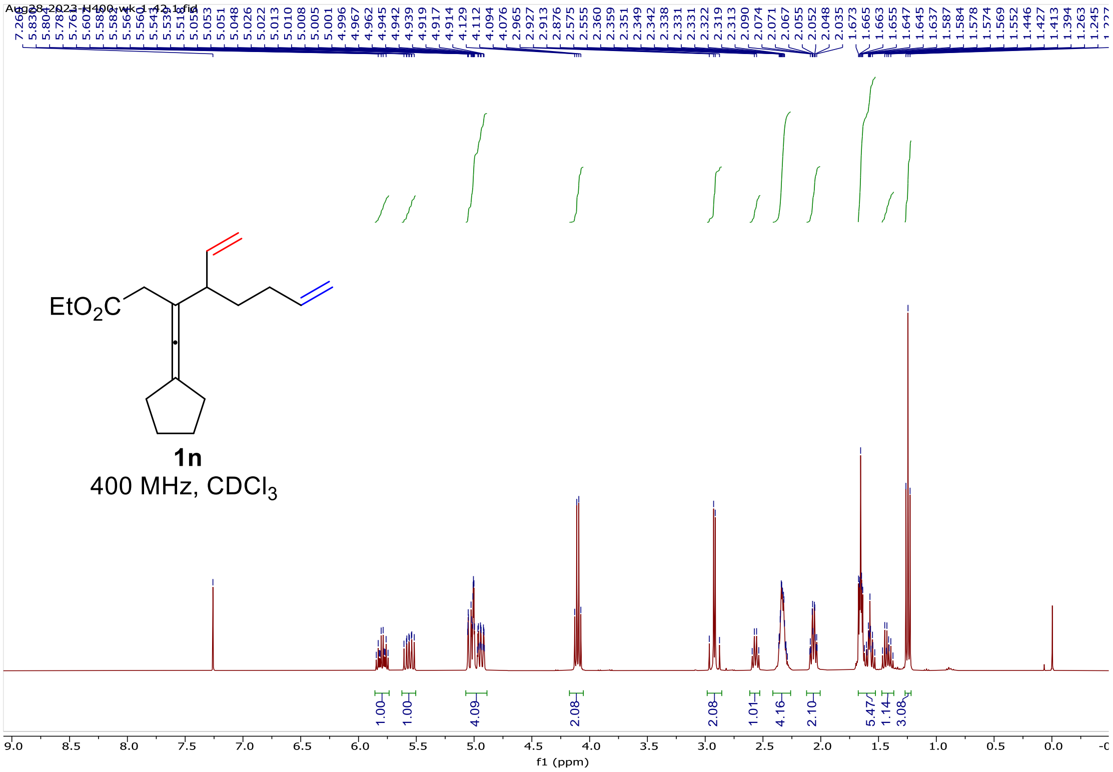
EtO2C
1n
400 MHz, CDCl3
1.00
1.00
4.09
2.08
2.08
1.01
4.16
2.10
5.47
1.14
3.08
f1 (ppm)

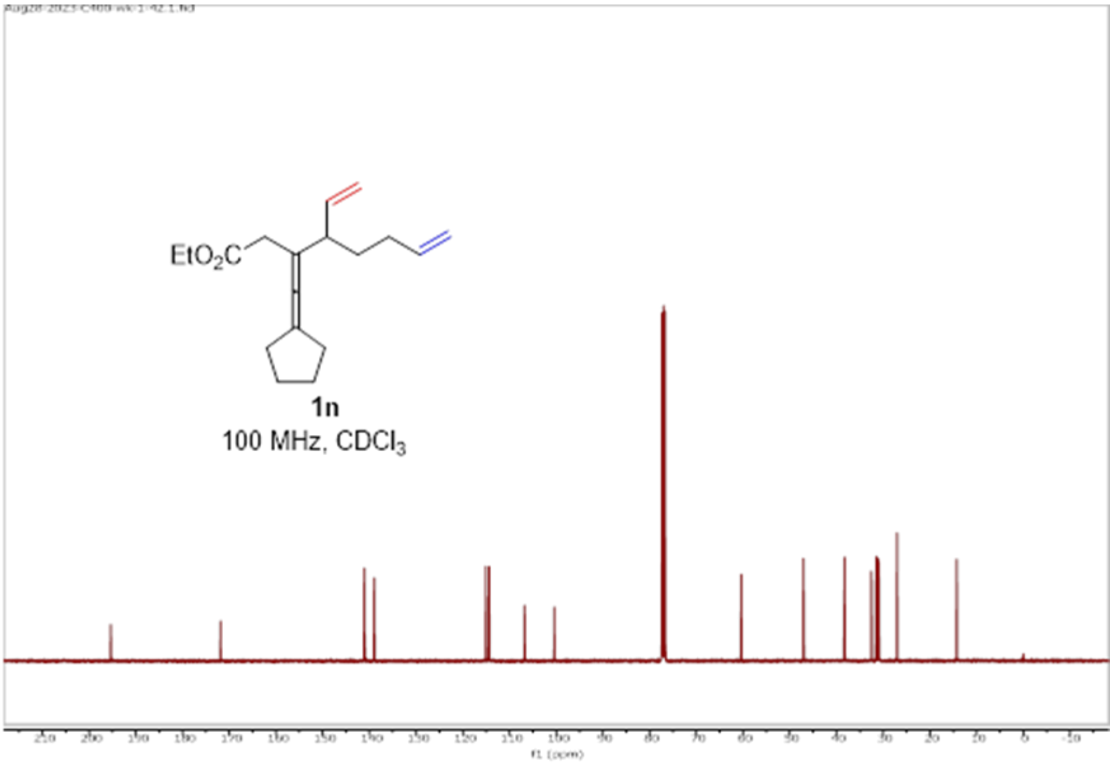
EtO2C
1n
100 MHz, CDCl3

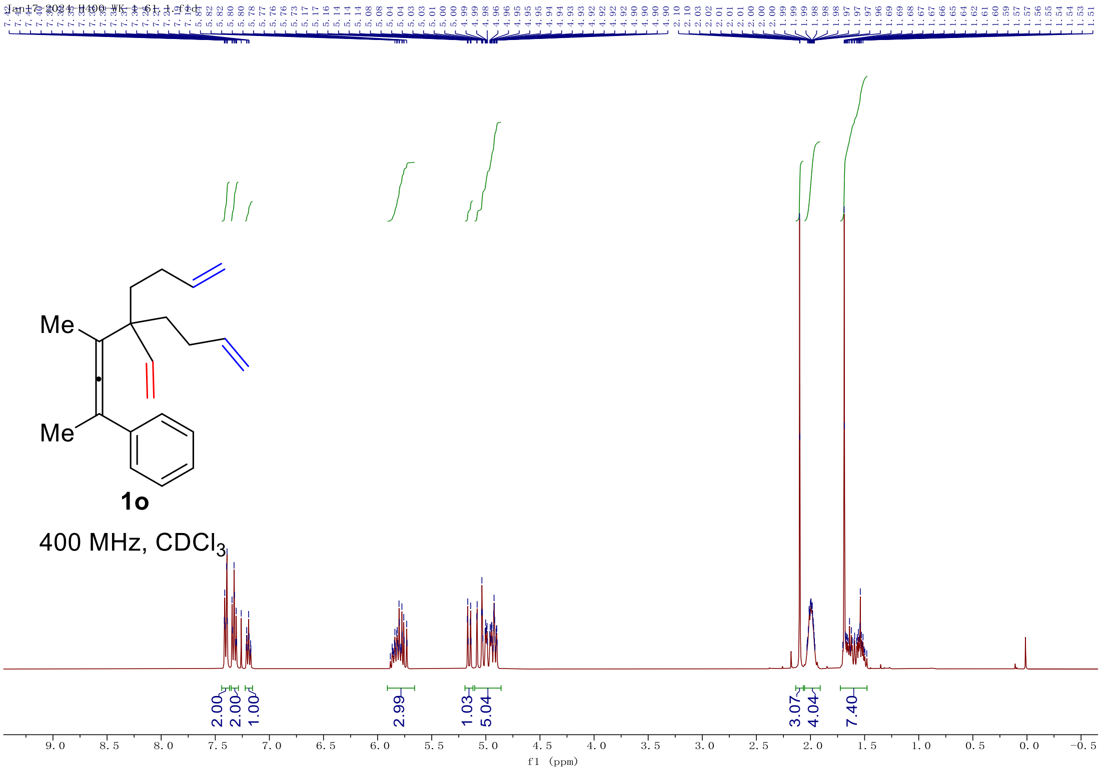

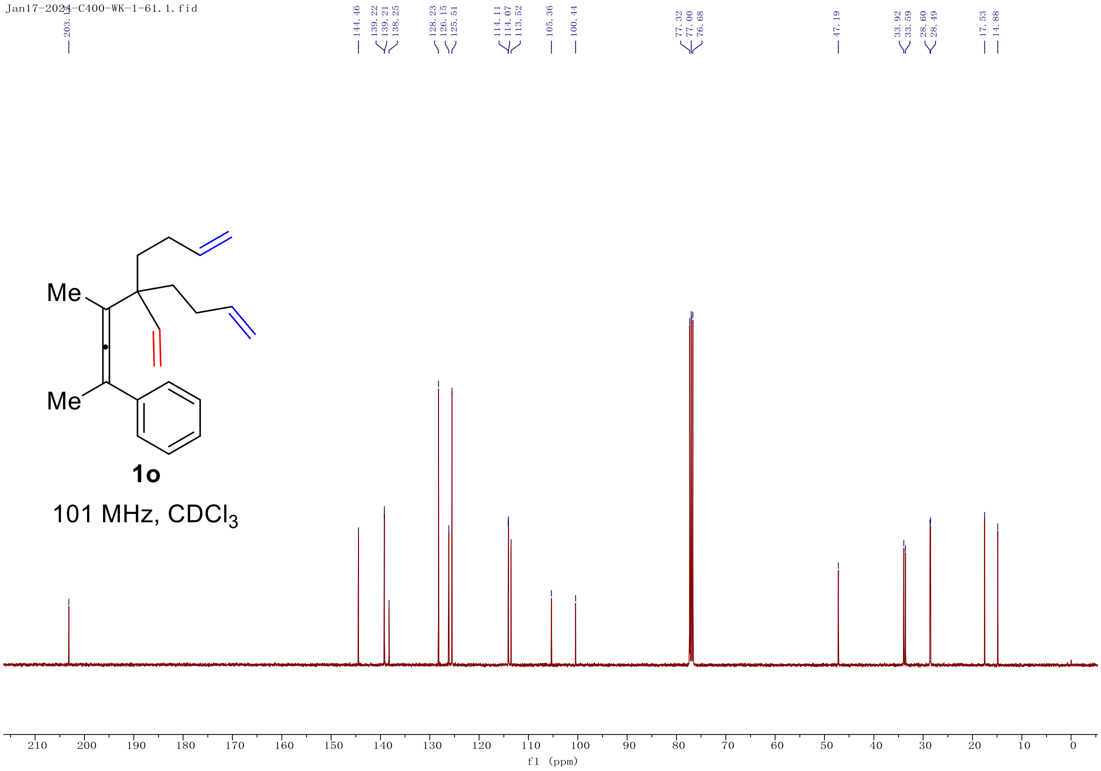

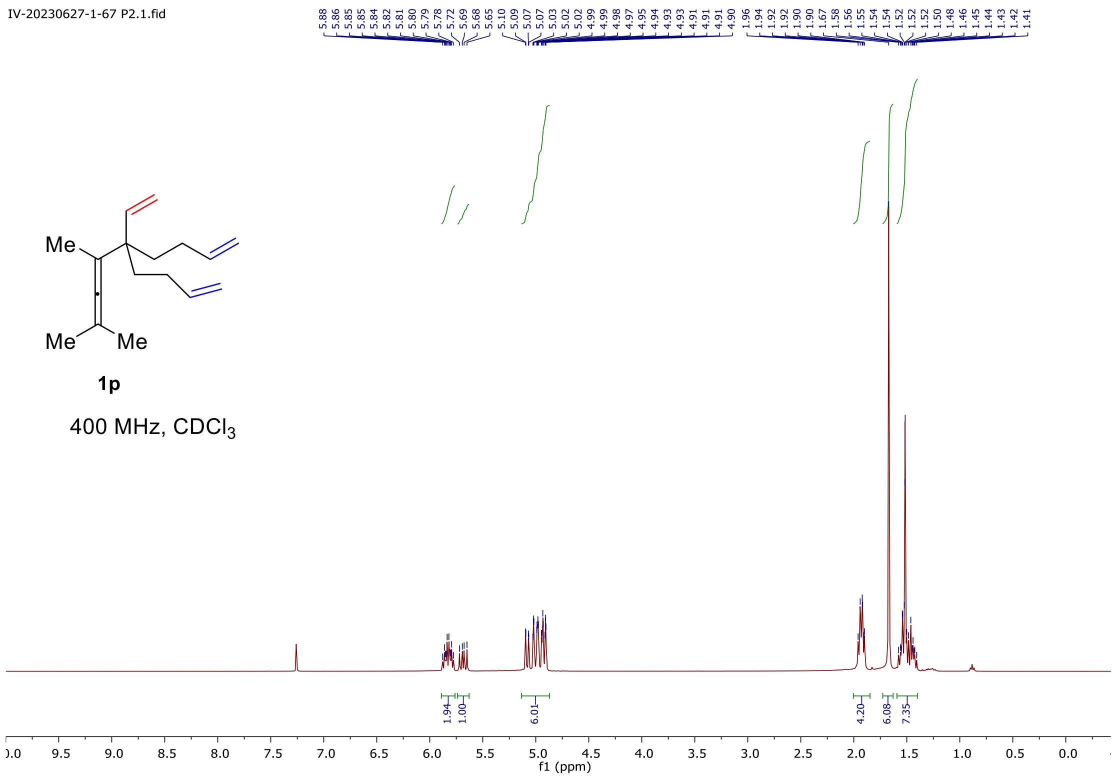
IV-20230627-1-67 P2.1.fid
Me
Me
Me
1p
400 MHz, CDCl3
f1 (ppm)

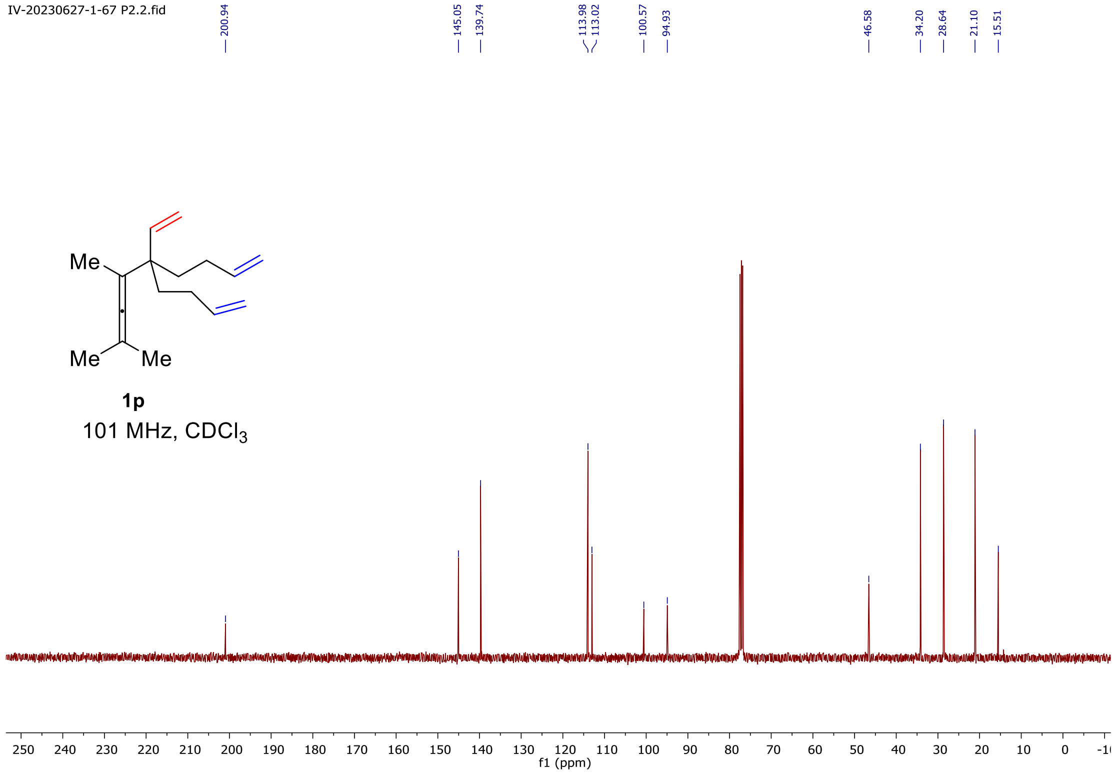
IV-20230627-1-67 P2.2.fid
200.94
145.05
139.74
113.98
113.02
100.57
94.93
46.58
34.20
28.64
21.10
15.51
Me
Me
Me
1p
101 MHz, CDCl3
f1 (ppm)

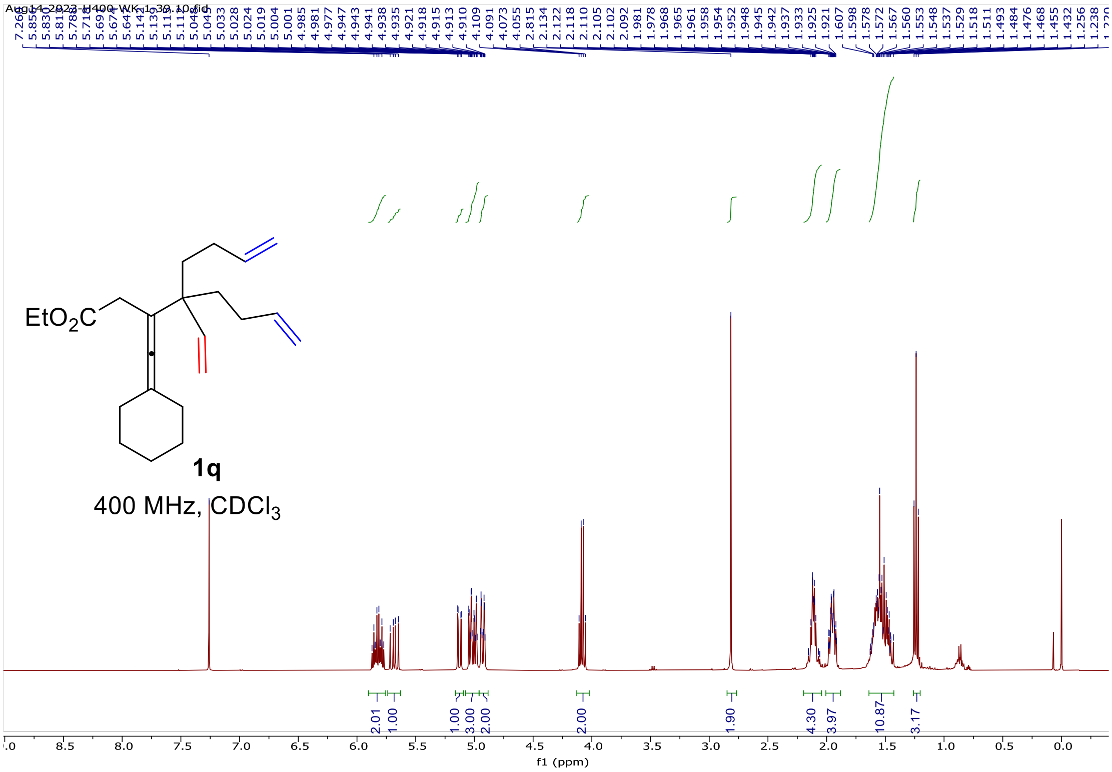
Aug14-2023-H400-WK-1-39.10.fid
EtO2C
1q
400 MHz, CDCl3
2.01
1.00
1.00
3.00
2.00
2.00
1.90
4.30
3.97
10.87
3.17
f1 (ppm)

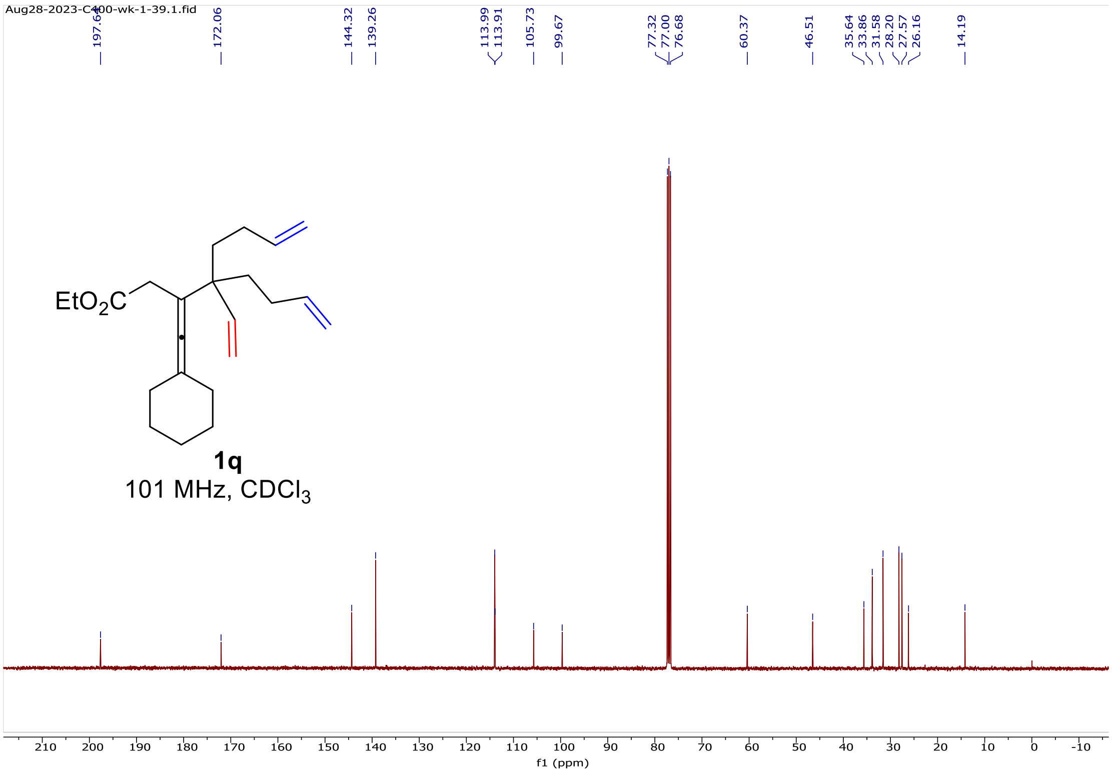
Aug28-2023-C400-wk-1-39.1.fid
197.60
172.06
144.32
139.26
113.99
113.91
105.73
99.67
77.32
77.00
76.68
60.37
46.51
35.64
33.86
31.58
28.20
27.57
26.16
14.19
EtO2C
1q
101 MHz, CDCl3
f1 (ppm)

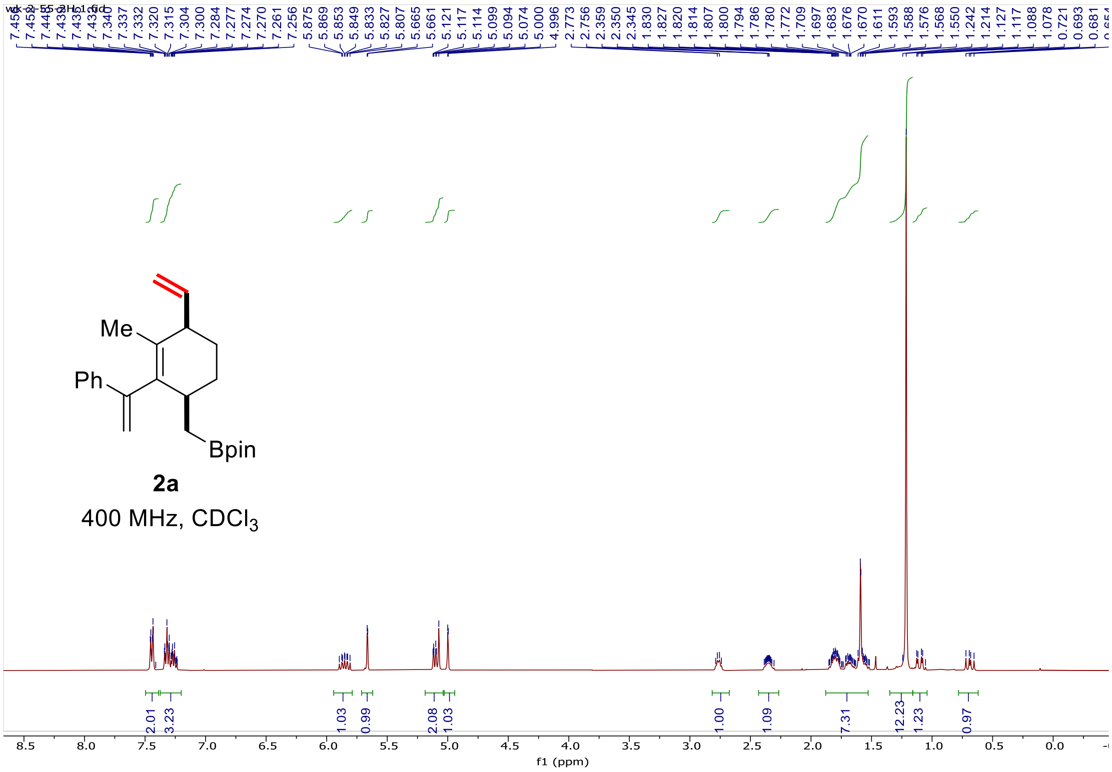
wk-2-55-2H.1.fid
7.453 7.445 7.443 7.437 7.433 7.340 7.337 7.332 7.320 7.315 7.304 7.300 7.284 7.277 7.274 7.270 7.261 7.256 5.875 5.869 5.853 5.849 5.833 5.827 5.807 5.665 5.661 5.121 5.117 5.114 5.099 5.094 5.074 5.000 4.996 2.773 2.756 2.359 2.350 2.345 1.830 1.827 1.820 1.814 1.807 1.800 1.794 1.786 1.780 1.772 1.709 1.697 1.683 1.676 1.670 1.611 1.593 1.588 1.576 1.568 1.550 1.242 1.214 1.127 1.117 1.088 1.078 0.721 0.693 0.681
Me
Ph
Bpin
2a
400 MHz, CDCl3
2.01
3.23
1.03
0.99
2.08
1.03
1.00
1.09
7.31
12.23
1.23
0.97
f1 (ppm)

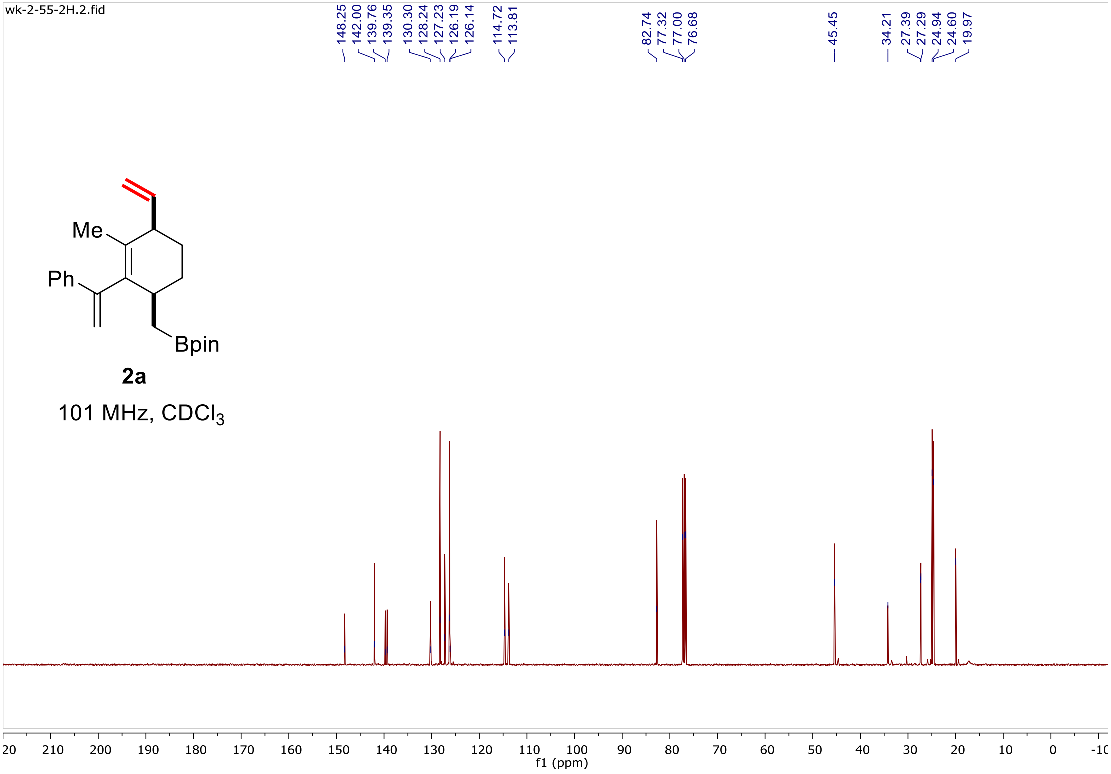
wk-2-55-2H.2.fid
148.25 142.00 139.76 139.35 130.30 128.24 127.23 126.19 126.14 114.72 113.81 82.74 77.32 77.00 76.68 45.45 34.21 27.39 27.29 24.94 24.60 19.97
Me
Ph
Bpin
2a
101 MHz, CDCl3
f1 (ppm)

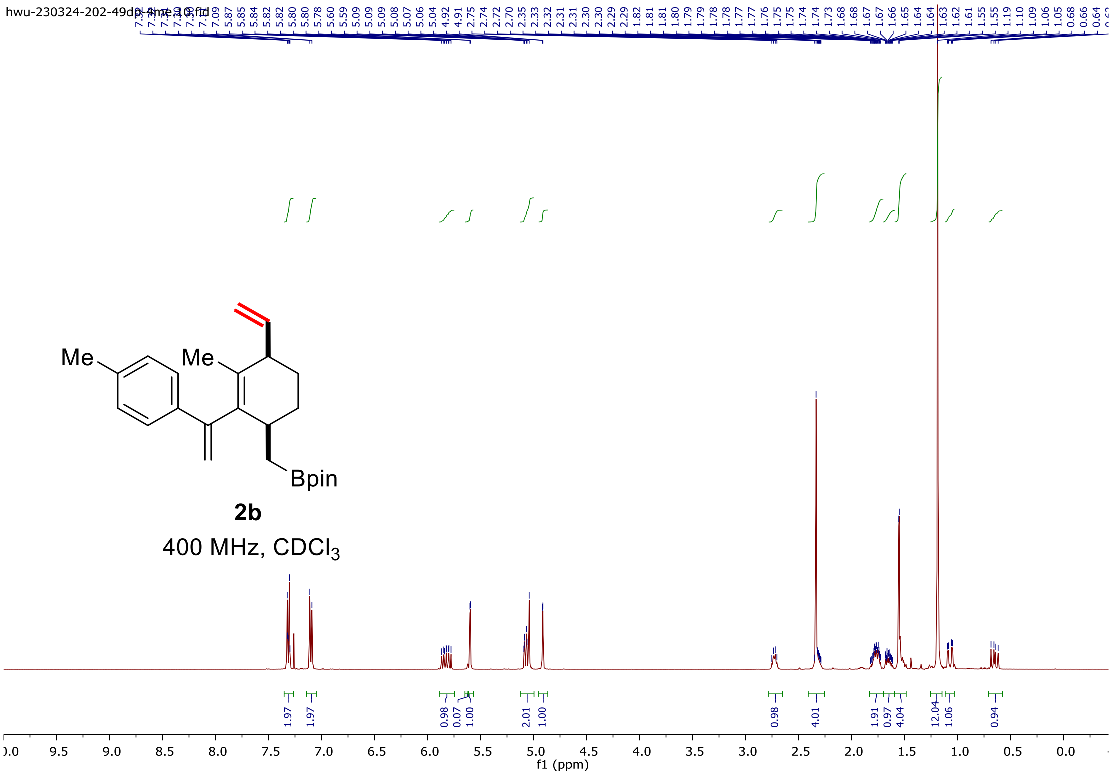
hwu-230324-202-49dp-4me.10.fid
5.87 5.85 5.84 5.82 5.82 5.80 5.80 5.78 5.60 5.59 5.09 5.09 5.09 5.08 5.07 5.06 5.04 4.92 4.91 2.75 2.74 2.72 2.70 2.35 2.33 2.32 2.31 2.31 2.30 2.30 2.29 2.29 1.82 1.81 1.81 1.80 1.79 1.79 1.78 1.78 1.77 1.77 1.76 1.75 1.75 1.74 1.74 1.73 1.68 1.67 1.67 1.66 1.65 1.64 1.64 1.63 1.62 1.61 1.55 1.55 1.19 1.10 1.09 1.06 1.05 0.68 0.66 0.64
Me
Me
Bpin
2b
400 MHz, CDCl3
1.97 1.97 0.98 0.07 1.00 2.01 1.00 0.98 4.01 1.91 0.97 4.04 12.04 1.06 0.94
f1 (ppm)

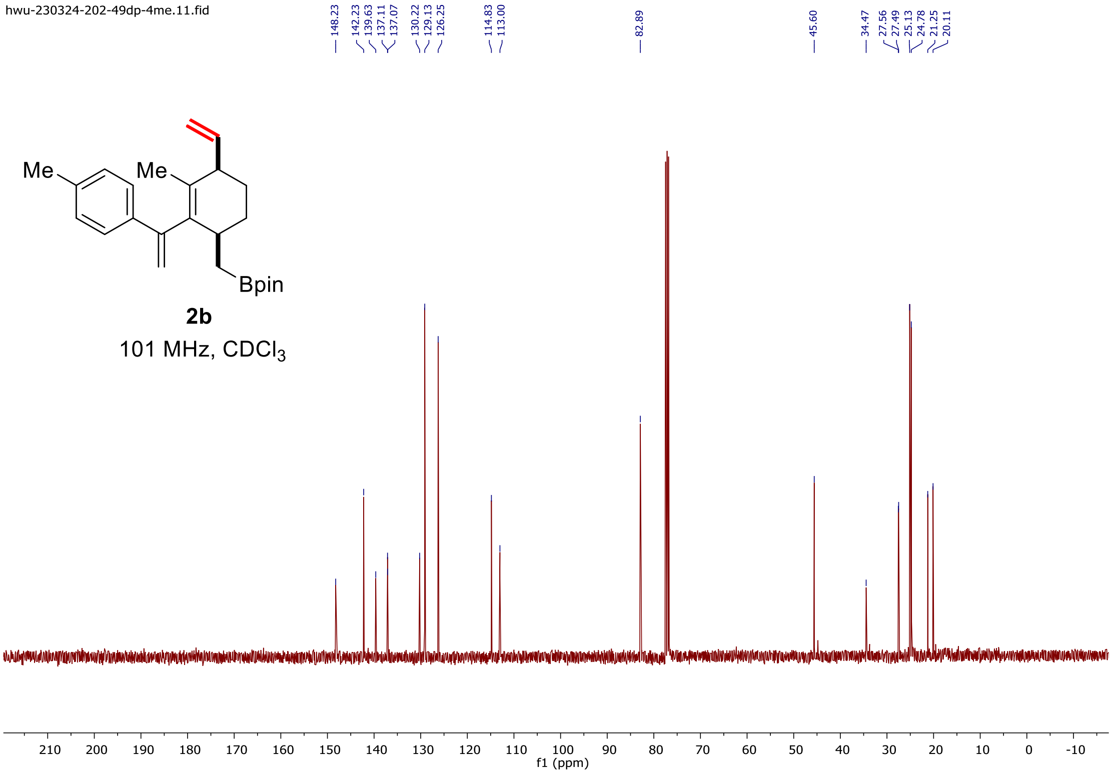
hwu-230324-202-49dp-4me.11.fid
148.23 142.23 139.63 137.11 137.07 130.22 129.13 126.25 114.83 113.00 82.89 45.60 34.47 27.56 27.49 25.13 24.78 21.25 20.11
Me
Me
Bpin
2b
101 MHz, CDCl3
f1 (ppm)

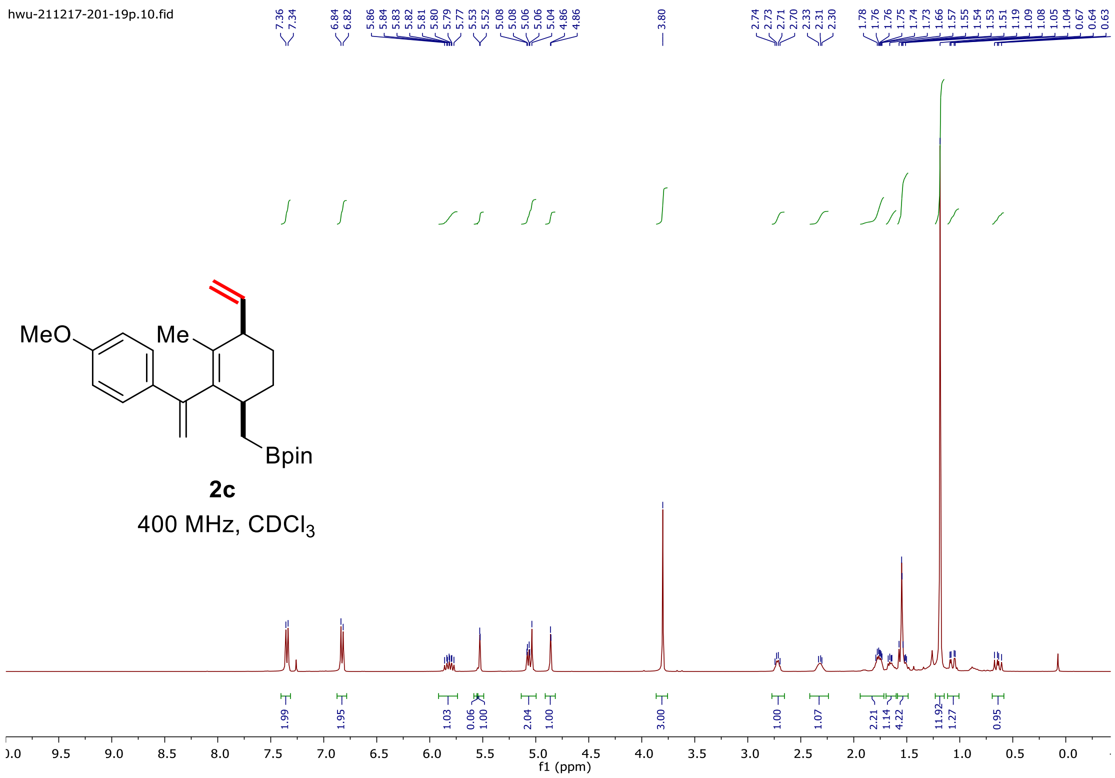
hwu-211217-201-19p.10.fid
7.36 7.34 6.84 6.82 5.86 5.84 5.83 5.82 5.81 5.80 5.79 5.77 5.53 5.52 5.08 5.08 5.06 5.06 5.04 4.86 4.86 3.80 2.74 2.73 2.71 2.70 2.33 2.31 2.30 1.78 1.76 1.76 1.75 1.74 1.73 1.66 1.57 1.55 1.54 1.53 1.51 1.19 1.09 1.08 1.05 1.04 0.67 0.64 0.63
MeO
Me
Bpin
2c
400 MHz, CDCl3
1.99 1.95 1.03 0.06 1.00 2.04 1.00 3.00 1.00 1.07 2.21 1.14 4.22 11.92 1.27 0.95
f1 (ppm)

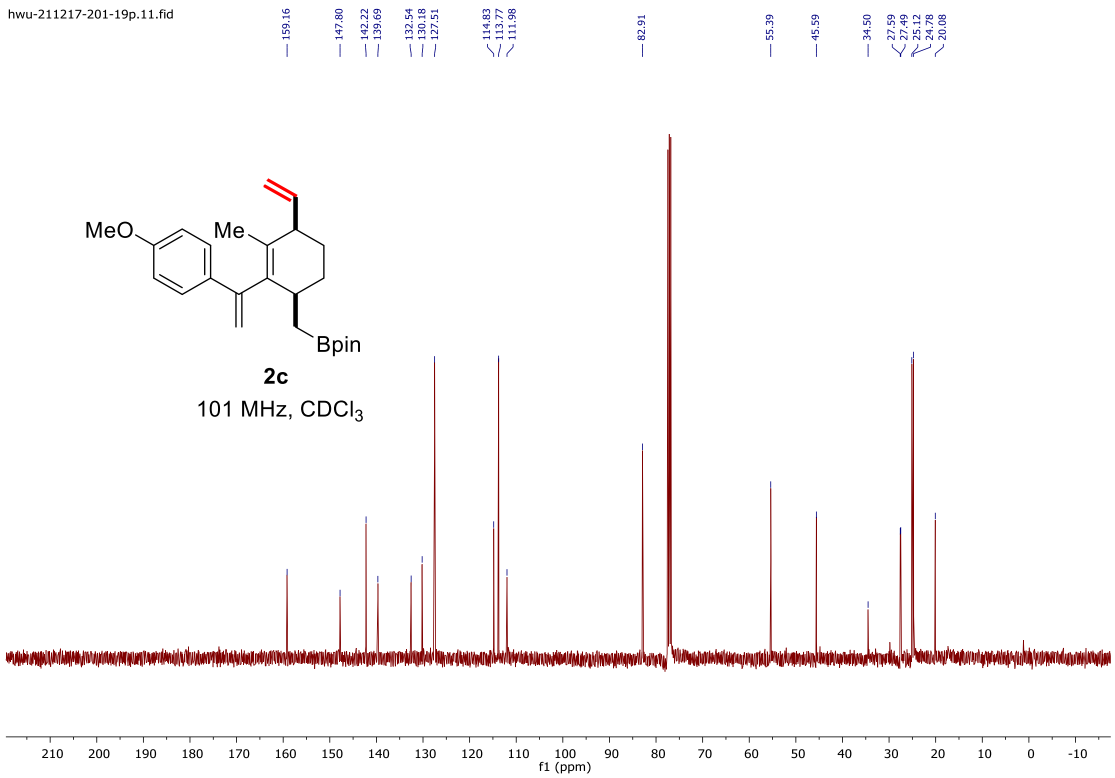
hwu-211217-201-19p.11.fid
159.16 147.80 142.22 139.69 132.54 130.18 127.51 114.83 113.77 111.98 82.91 55.39 45.59 34.50 27.59 27.49 25.12 24.78 20.08
MeO
Me
Bpin
2c
101 MHz, CDCl3
f1 (ppm)

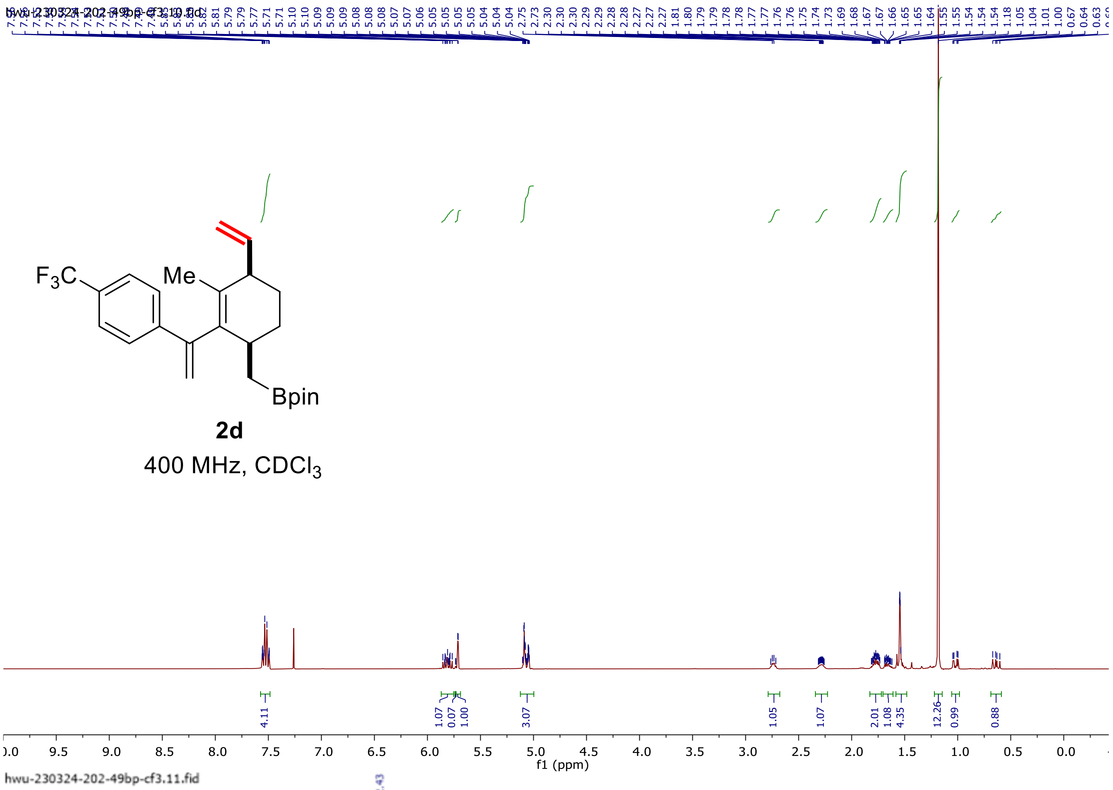
F3C
Me
Bpin
2d
400 MHz, CDCl3
4.11
1.07
0.07
1.00
3.07
1.05
1.07
2.01
1.08
4.35
12.26
0.99
0.88
f1 (ppm)

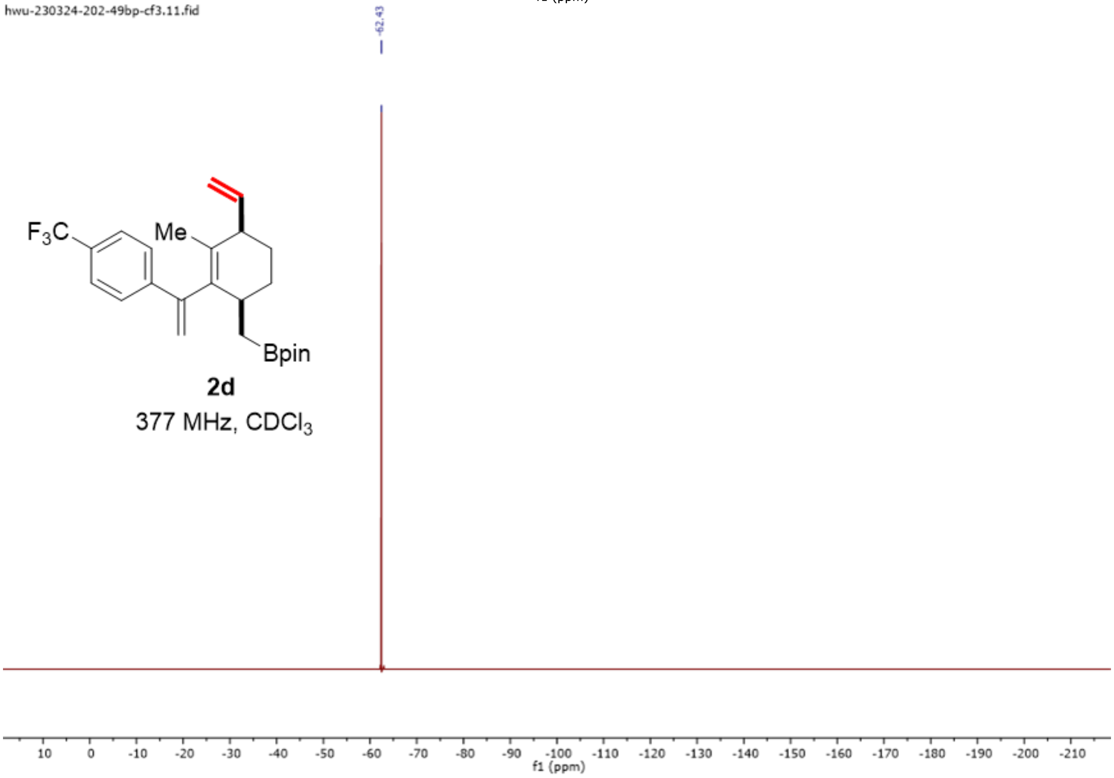
hwu-230324-202-49bp-cf3.11.fid
-62.43
F3C
Me
Bpin
2d
377 MHz, CDCl3
f1 (ppm)

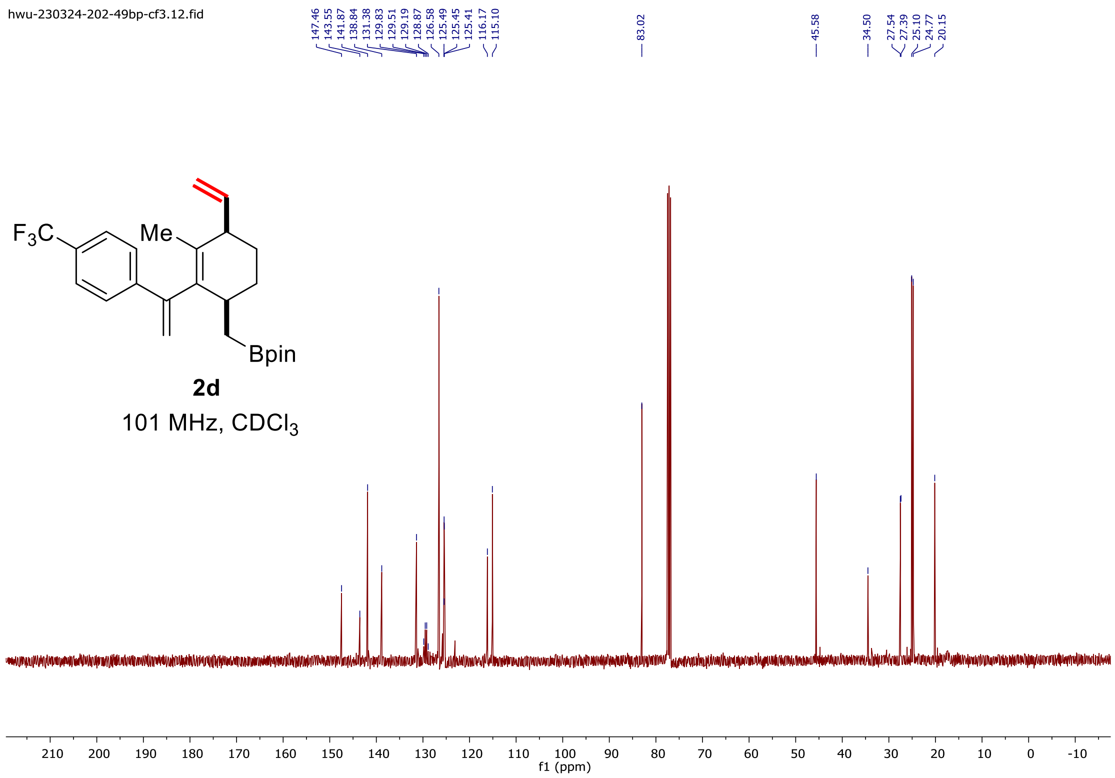
hwu-230324-202-49bp-cf3.12.fid
147.46
143.55
141.87
138.84
131.38
129.83
129.51
129.19
128.87
126.58
125.49
125.45
125.41
116.17
115.10
83.02
45.58
34.50
27.54
27.39
25.10
24.77
20.15
F_3C
Me
Bpin
2d
101 MHz, $CDCl_3$
f1 (ppm)

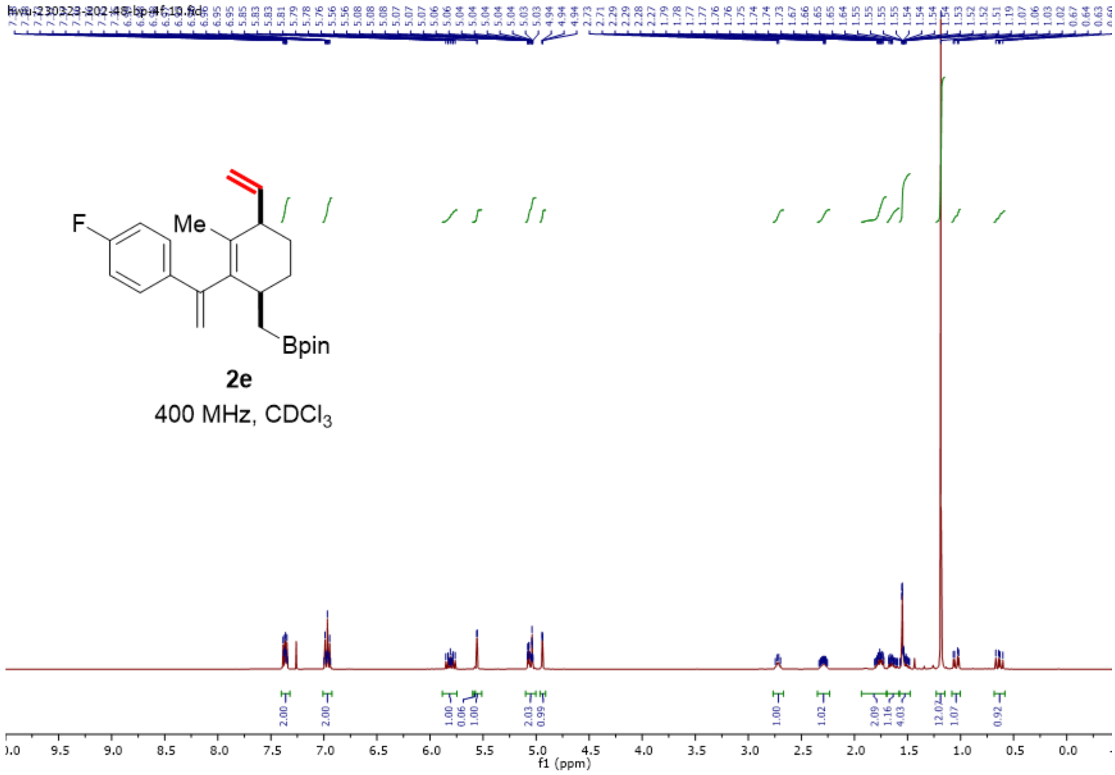
F
Me
Bpin
2e
400 MHz, $CDCl_3$
2.00
2.00
1.00
0.06
1.00
2.03
0.99
1.00
1.02
2.09
1.16
4.03
12.07
1.07
0.92
f1 (ppm)

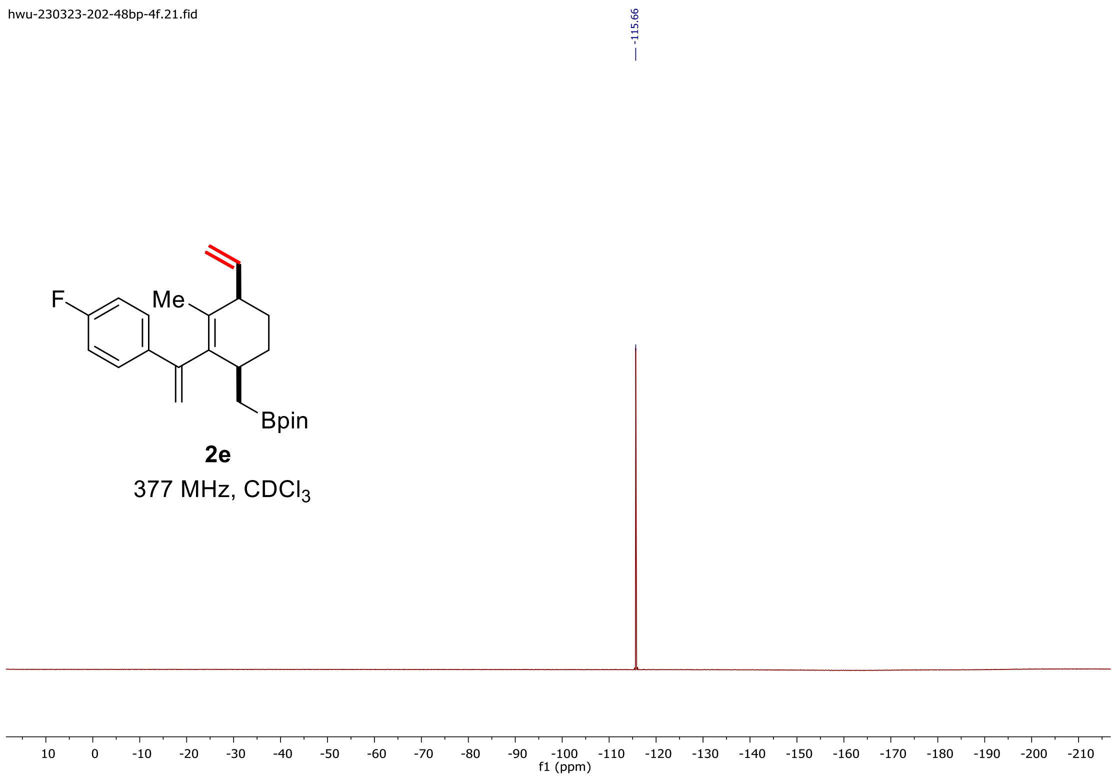
hwu-230323-202-48bp-4f.21.fid
-115.66
F
Me
Bpin
2e
377 MHz, CDCl3
f1 (ppm)

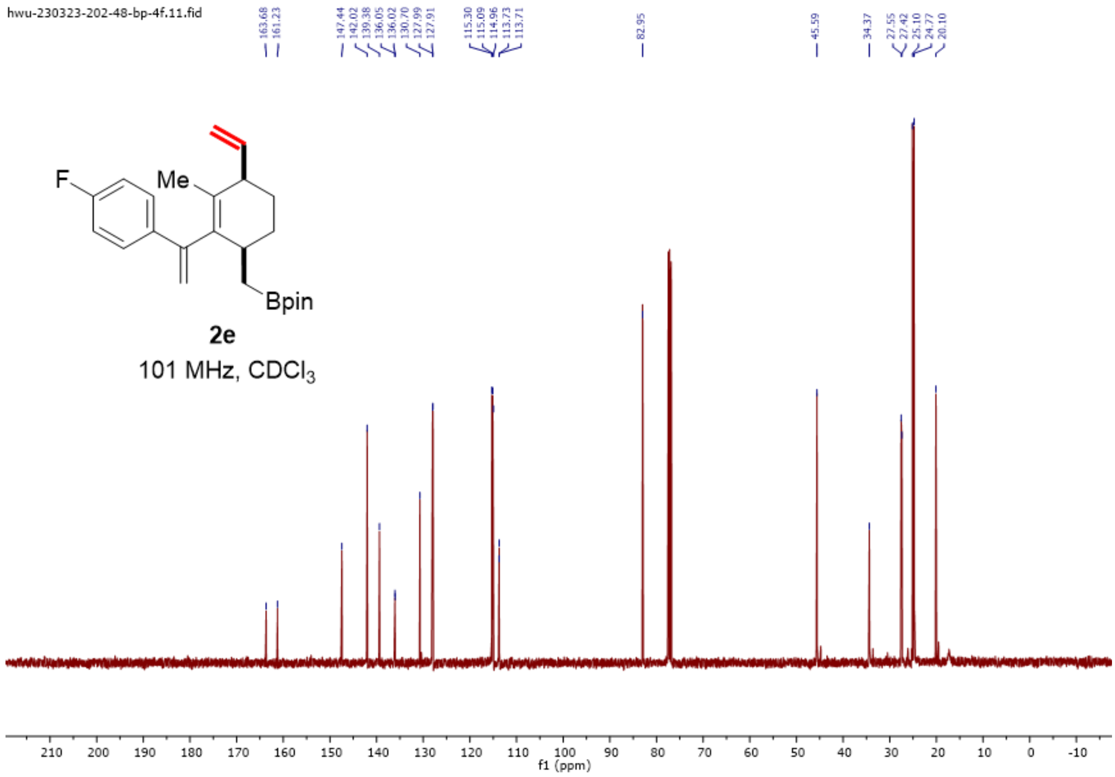
hwu-230323-202-48-bp-4f.11.fid
163.68
161.23
147.44
142.02
139.38
136.05
136.02
130.70
127.99
127.91
115.30
115.09
114.96
113.73
113.71
82.95
45.59
34.37
27.55
27.42
25.10
24.77
20.10
F
Me
Bpin
2e
101 MHz, CDCl3
f1 (ppm)

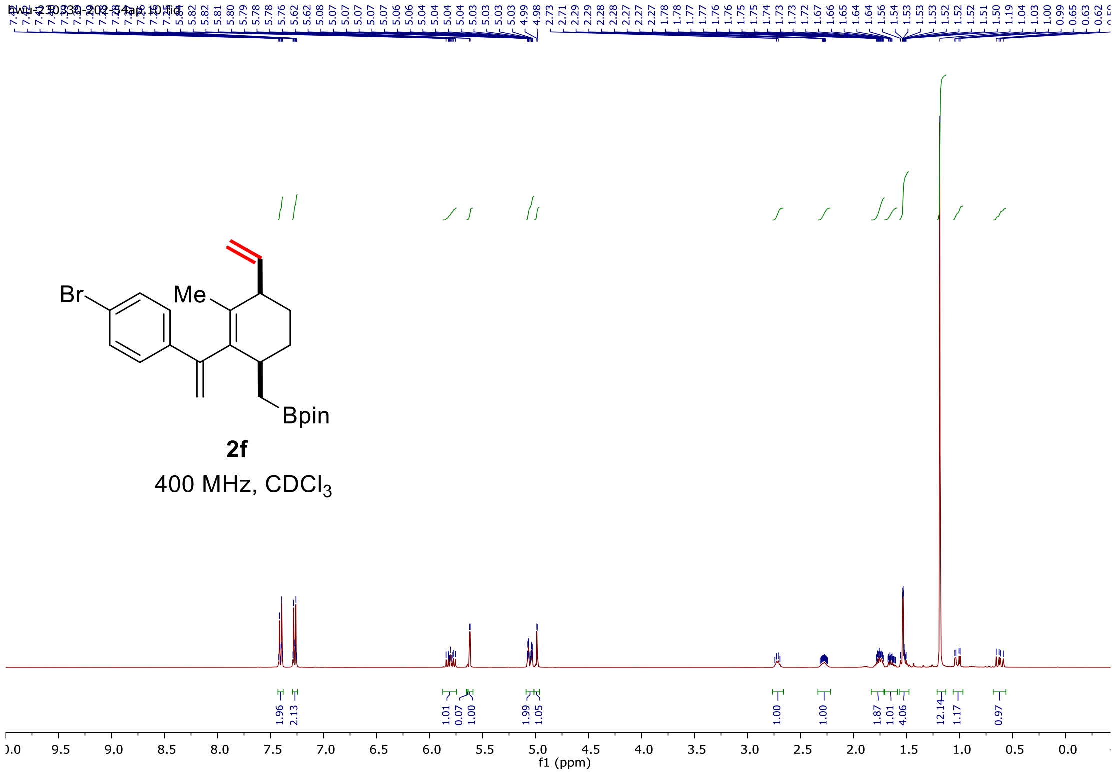
hwu-230330-202-54ap.10.fid
Br
Me
Bpin
2f
400 MHz, CDCl3
f1 (ppm)

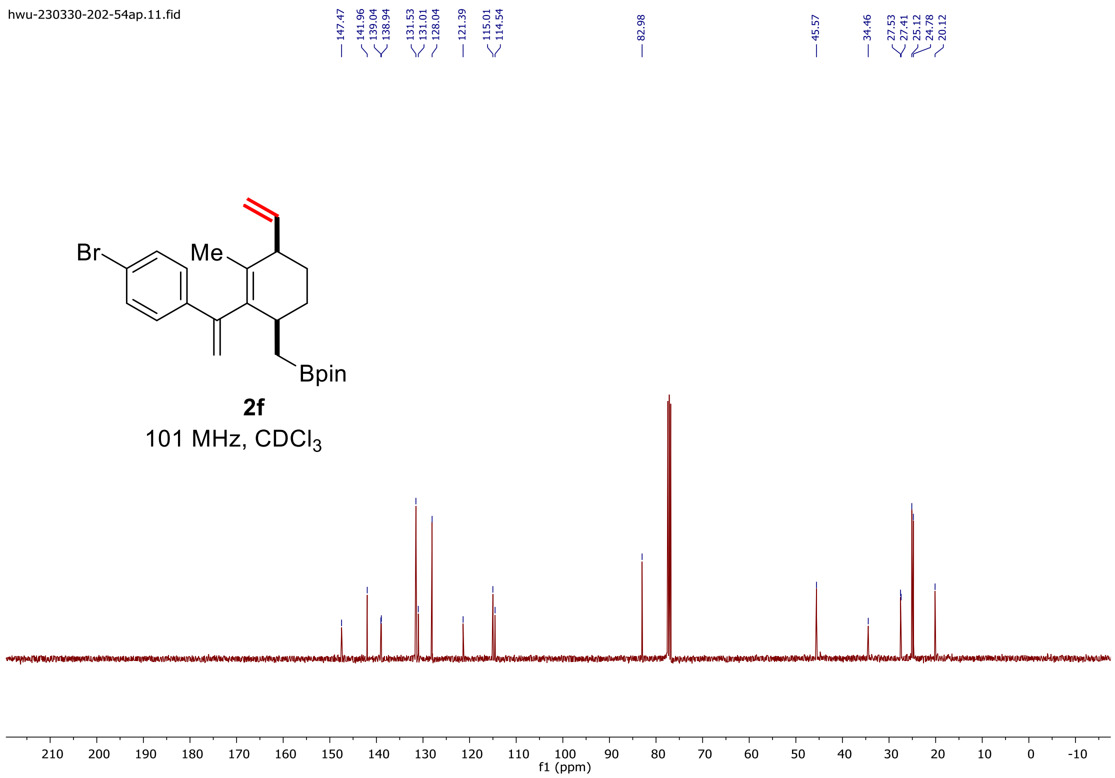
hwu-230330-202-54ap.11.fid
147.47
141.96
139.04
138.94
131.53
131.01
128.04
121.39
115.01
114.54
82.98
45.57
34.46
27.53
27.41
25.12
24.78
20.12
Br
Me
Bpin
2f
101 MHz, CDCl3
f1 (ppm)

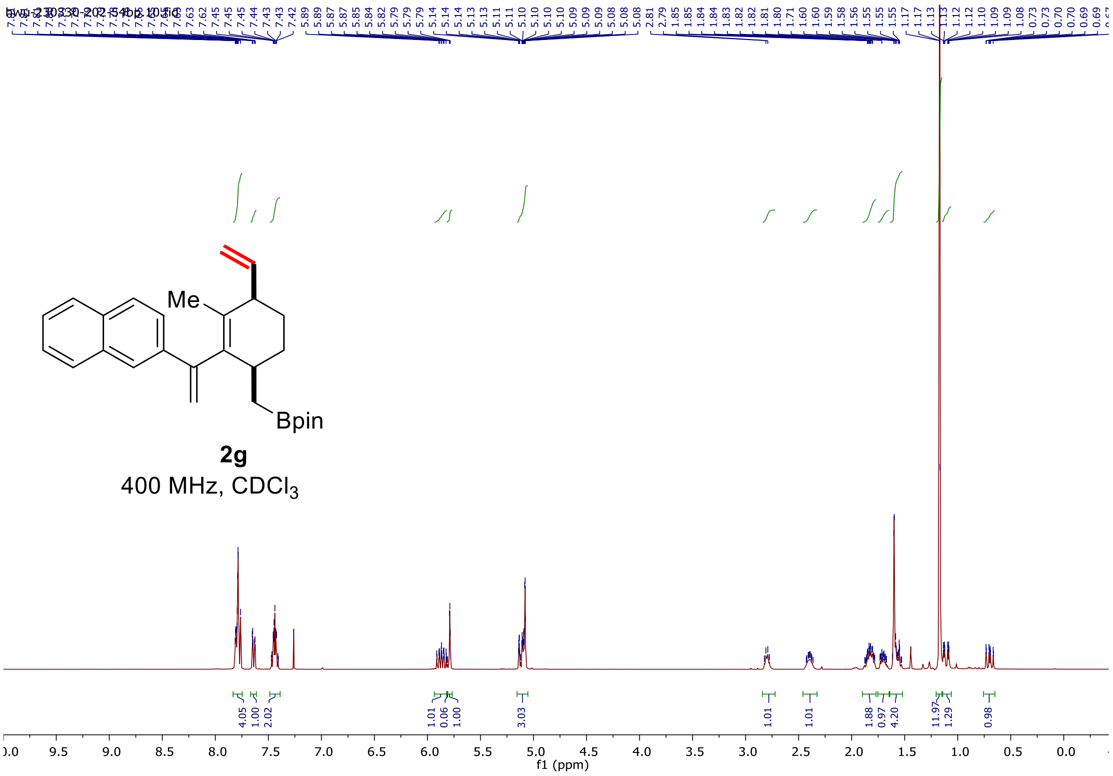
hwu-230330-202-54bp.10.fid
Me
Bpin
2g
400 MHz, CDCl3
f1 (ppm)

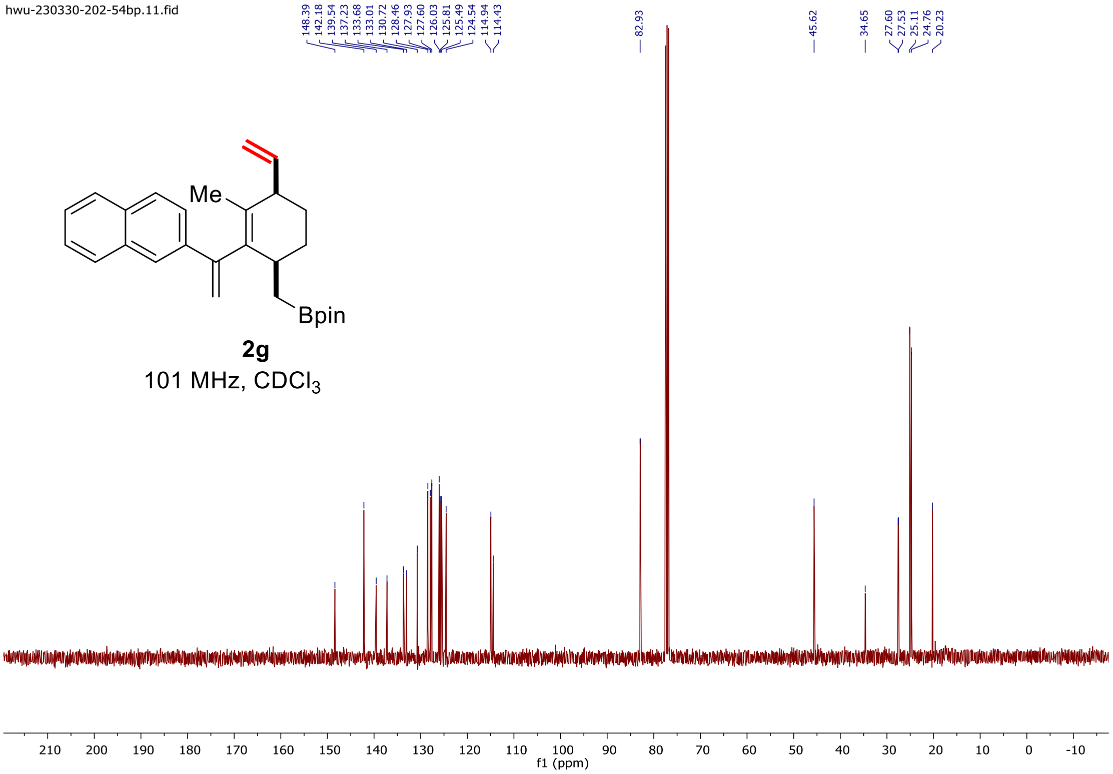
hwu-230330-202-54bp.11.fid
148.39
142.18
139.54
137.23
133.68
133.01
130.72
128.46
127.93
127.60
126.03
125.81
125.49
124.54
114.94
114.43
82.93
45.62
34.65
27.60
27.53
25.11
24.76
20.23
Me
Bpin
2g
101 MHz, CDCl3
f1 (ppm)

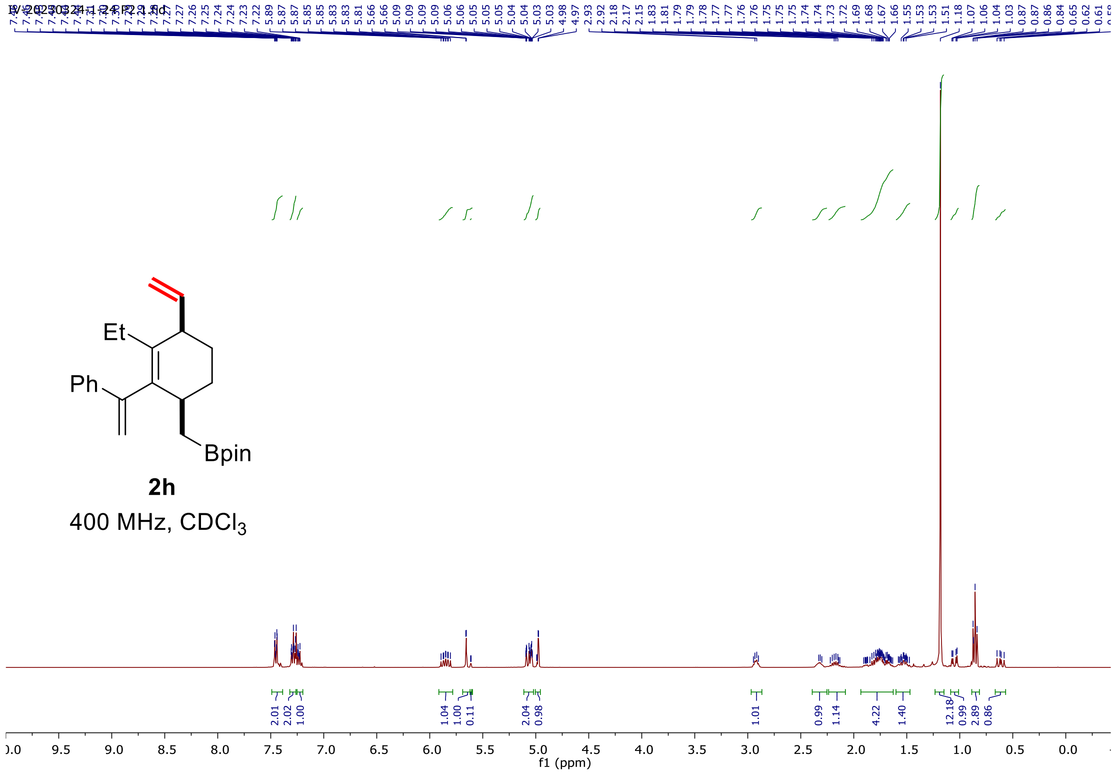
Et
Ph
Bpin
2h
400 MHz, CDCl3
7.27
7.26
7.25
7.24
7.24
7.23
7.22
5.89
5.87
5.87
5.85
5.85
5.83
5.83
5.81
5.66
5.66
5.09
5.09
5.09
5.09
5.06
5.06
5.05
5.05
5.05
5.04
5.04
5.03
5.03
4.98
4.97
2.93
2.92
2.18
2.17
2.15
1.83
1.81
1.79
1.78
1.77
1.77
1.76
1.76
1.75
1.75
1.75
1.74
1.74
1.73
1.72
1.69
1.68
1.67
1.66
1.55
1.53
1.53
1.51
1.18
1.07
1.06
1.04
1.03
0.87
0.87
0.86
0.84
0.65
0.62
0.61
2.01
2.02
1.00
1.04
1.00
0.11
2.04
0.98
1.01
0.99
1.14
4.22
1.40
12.18
0.99
2.89
0.86
f1 (ppm)

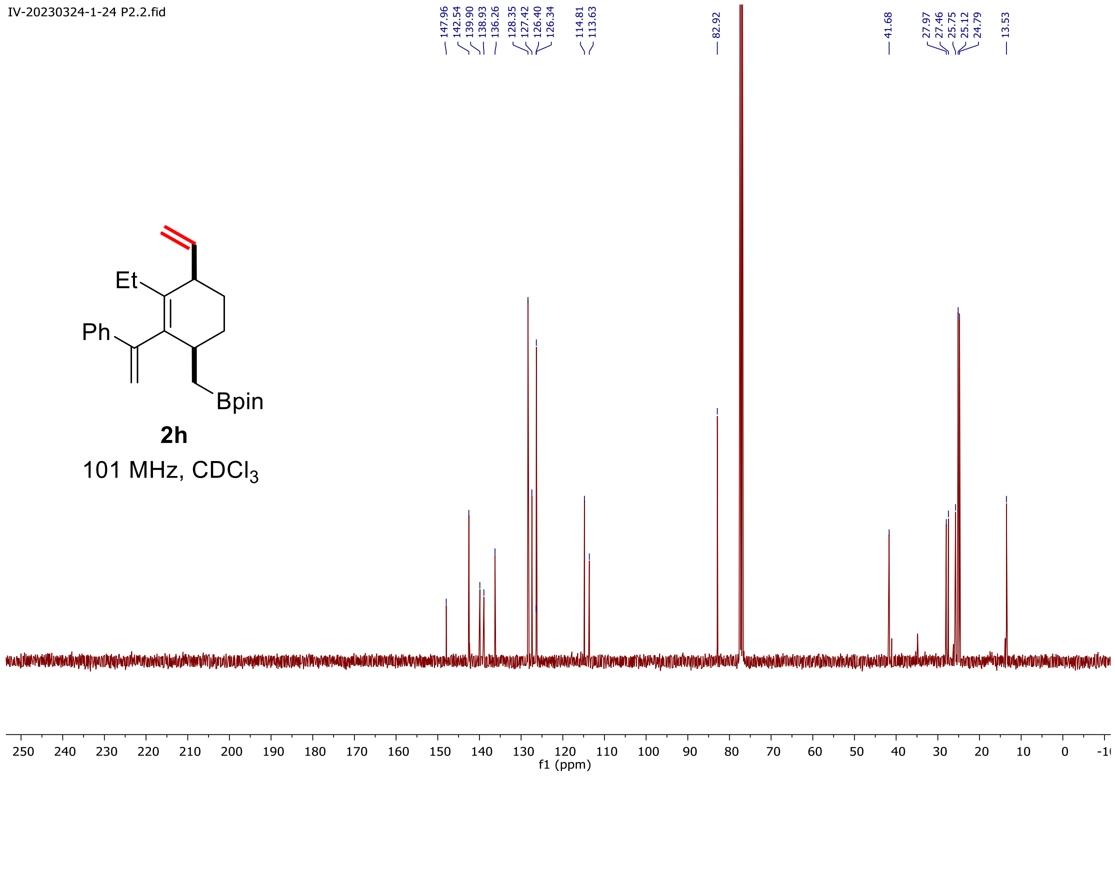
IV-20230324-1-24 P2.2.fid
147.96
142.54
139.90
138.93
136.26
128.35
127.42
126.40
126.34
114.81
113.63
82.92
41.68
27.97
27.46
25.75
25.12
24.79
13.53
Et
Ph
Bpin
2h
101 MHz, CDCl3
f1 (ppm)

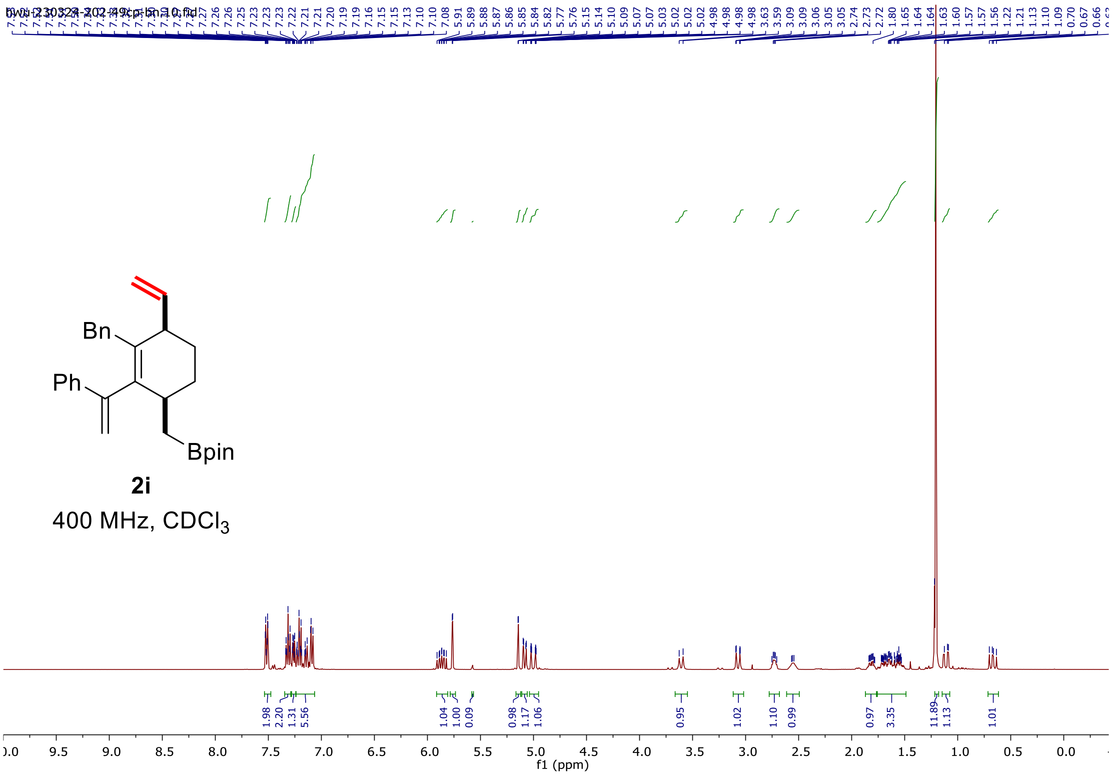
Bn
Ph
Bpin
2i
400 MHz, $CDCl_3$
f1 (ppm)

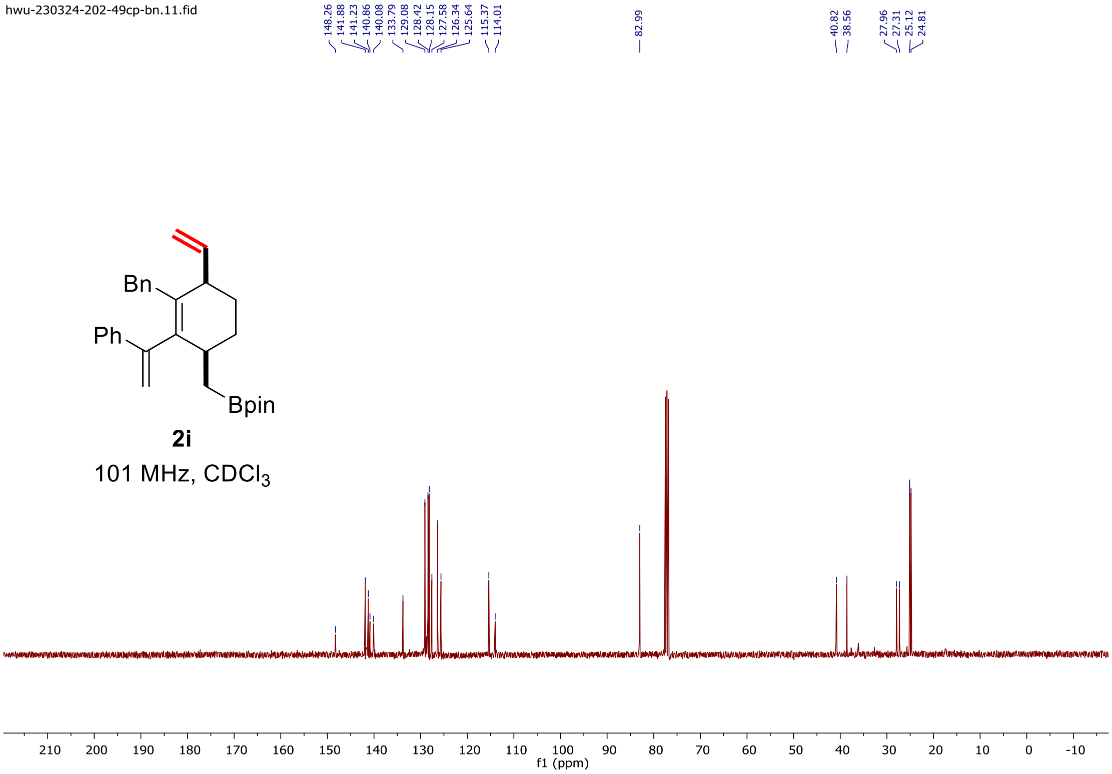
hwu-230324-202-49cp-bn.11.fid
148.26
141.88
141.23
140.86
140.08
133.79
129.08
128.42
128.15
127.58
126.34
125.64
115.37
114.01
82.99
40.82
38.56
27.96
27.31
25.12
24.81
Bn
Ph
Bpin
2i
101 MHz, $CDCl_3$
f1 (ppm)

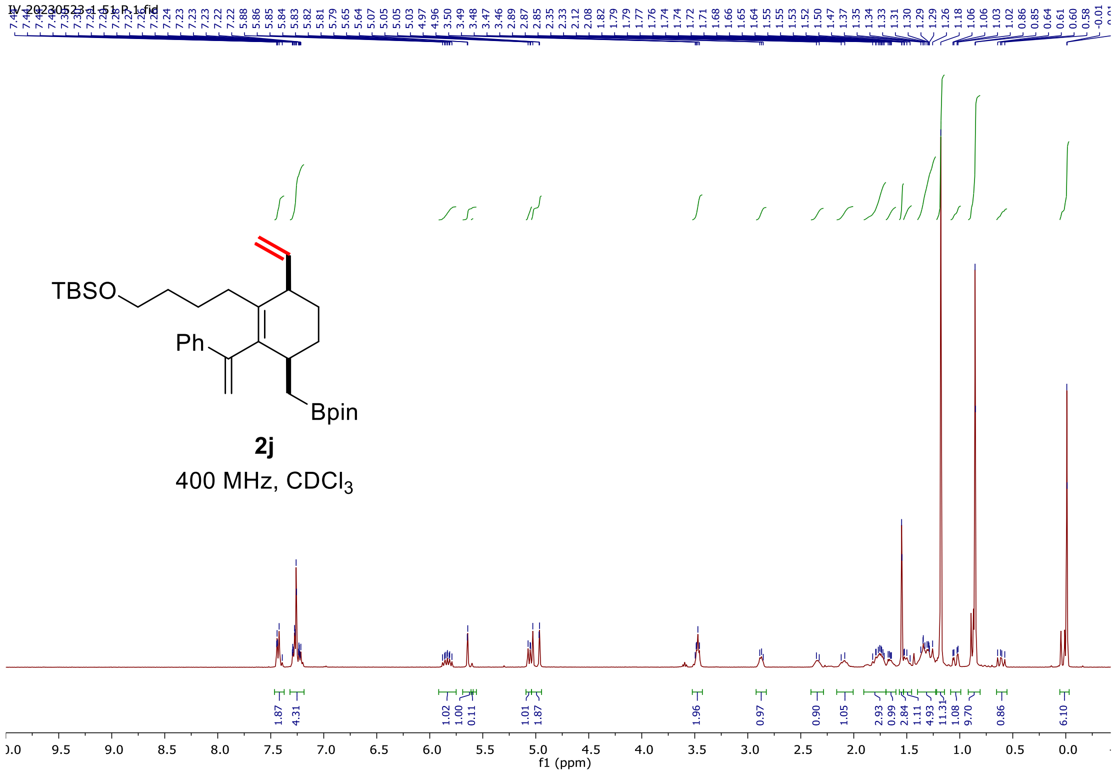
IV-20230523-1-51 P.1.fid
TBSO
Ph
Bpin
2j
400 MHz, CDCl3
f1 (ppm)

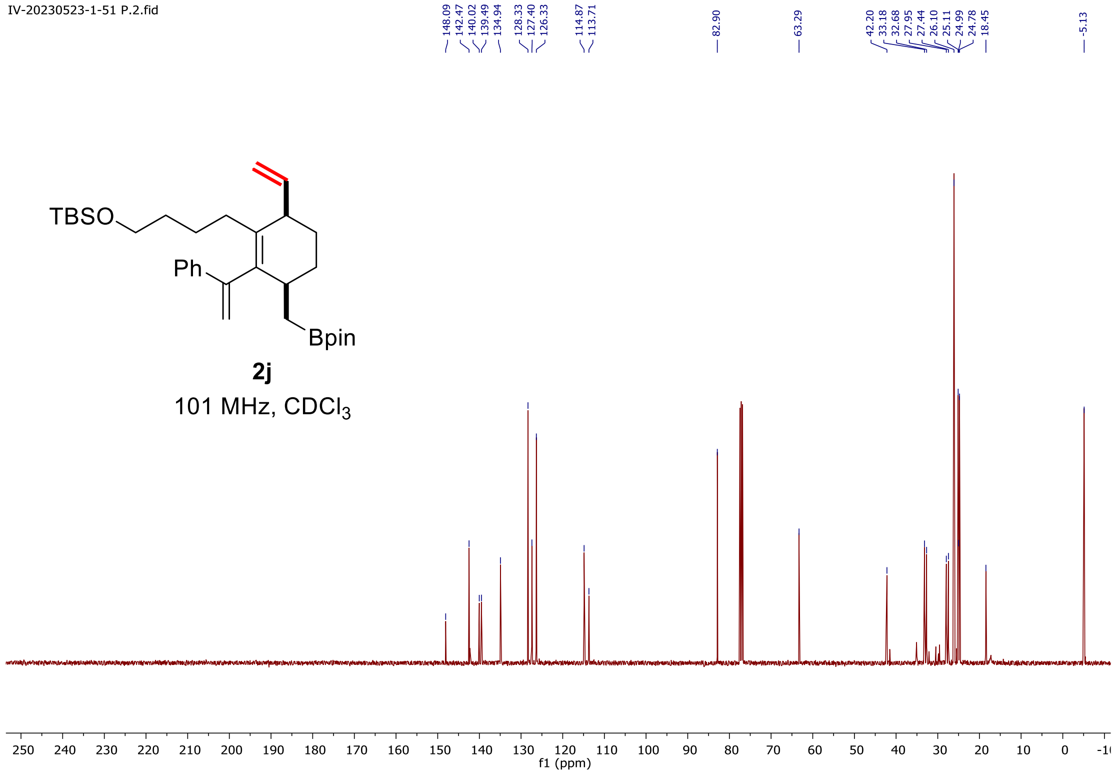
IV-20230523-1-51 P.2.fid
148.09
142.47
140.02
139.49
134.94
128.33
127.40
126.33
114.87
113.71
82.90
63.29
42.20
33.18
32.68
27.95
27.44
26.10
25.11
24.99
24.78
18.45
-5.13
TBSO
Ph
Bpin
2j
101 MHz, CDCl3
f1 (ppm)

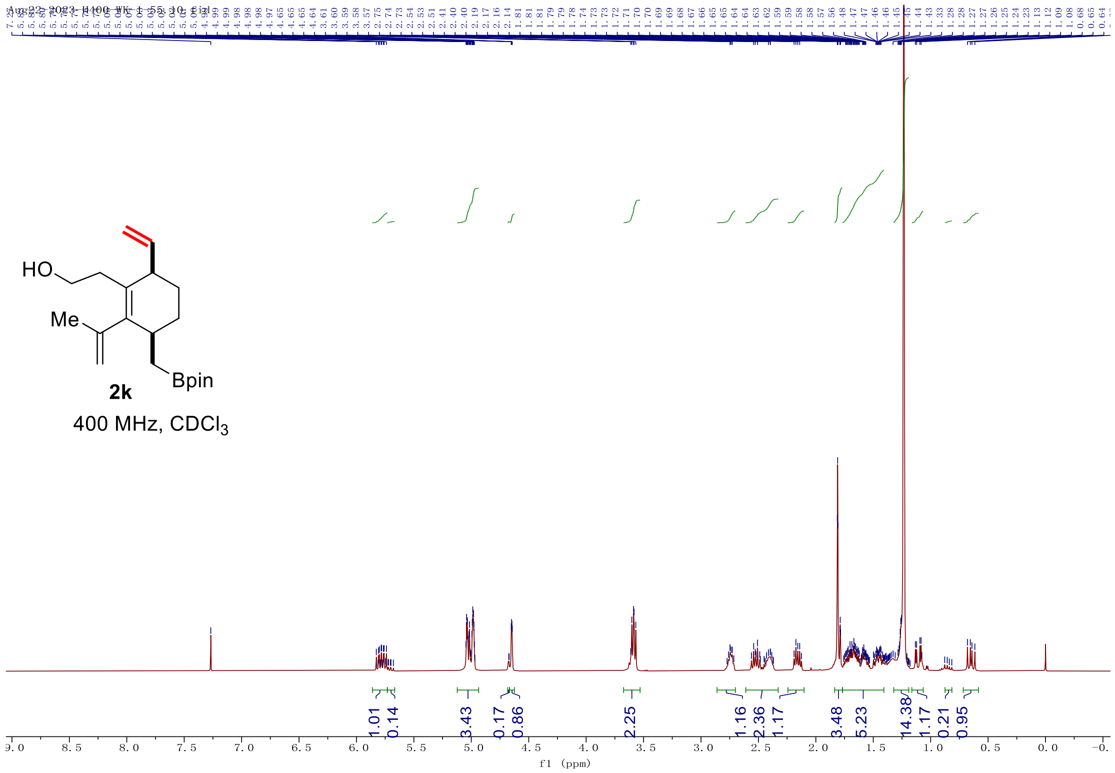
HO
Me
Bpin
2k
400 MHz, CDCl3
1.01
0.14
3.43
0.17
0.86
2.25
1.16
2.36
1.17
3.48
5.23
14.38
1.17
0.21
0.95
f1 (ppm)

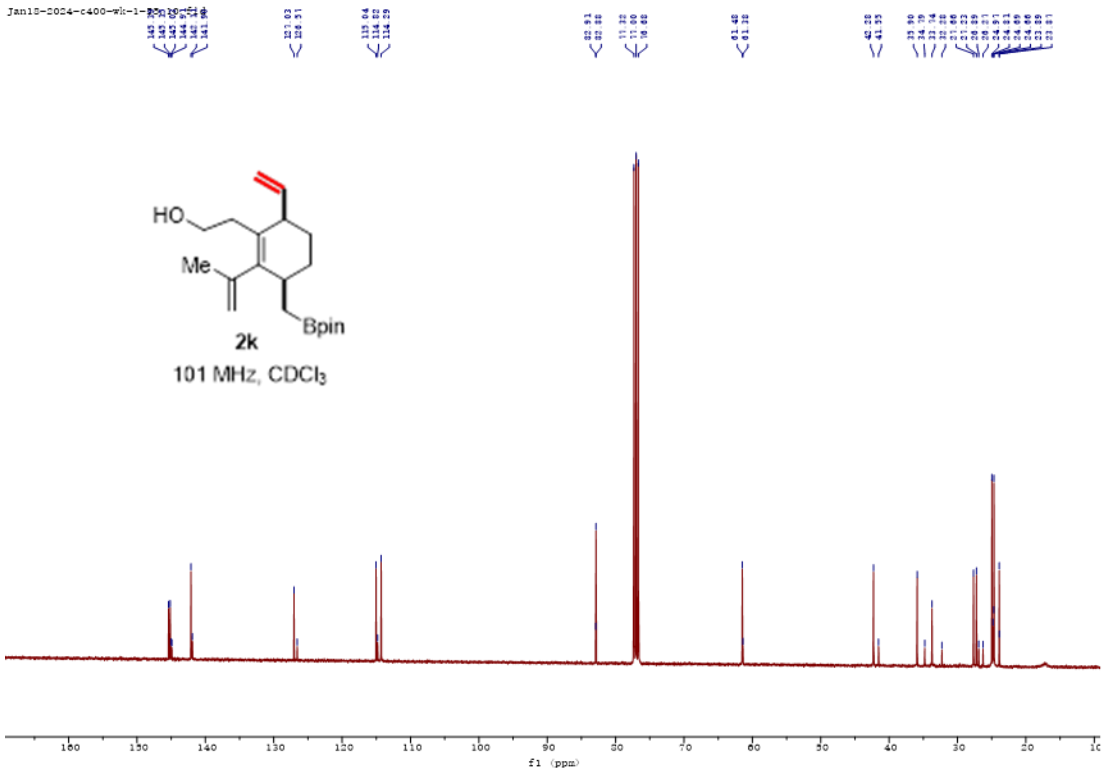
HO
Me
Bpin
2k
101 MHz, CDCl3
f1 (ppm)

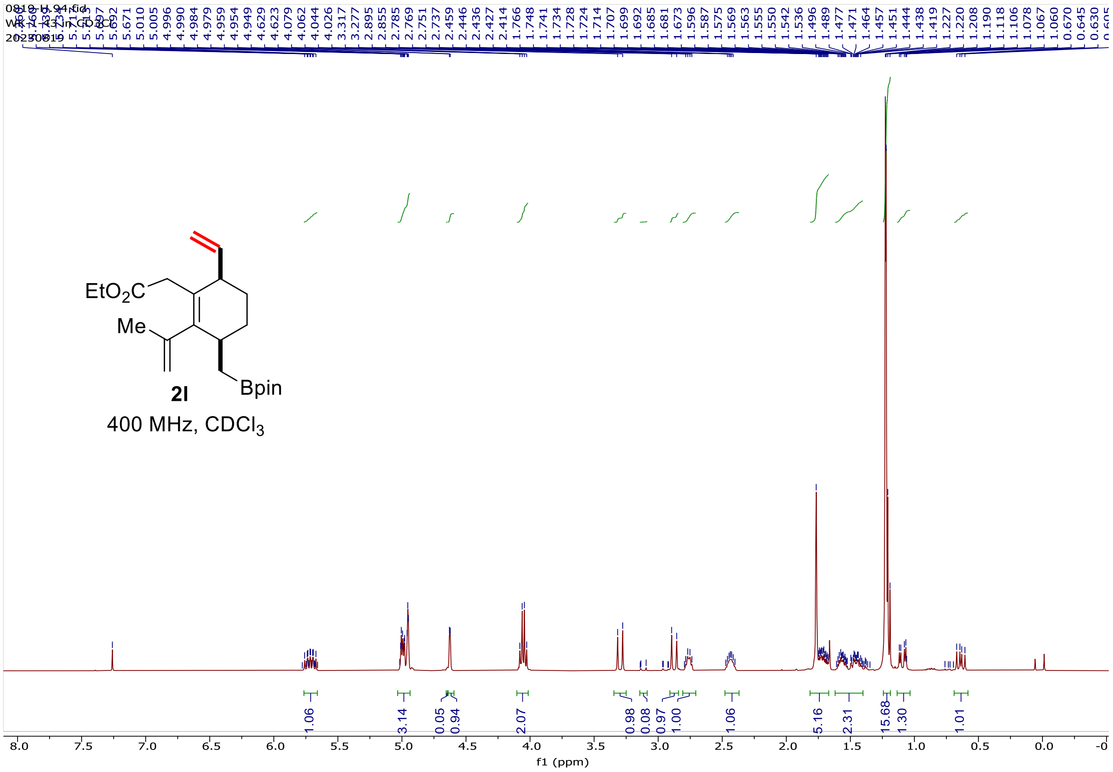
0819-H.94.fid
20230819
EtO2C
Me
Bpin
2l
400 MHz, CDCl3
f1 (ppm)

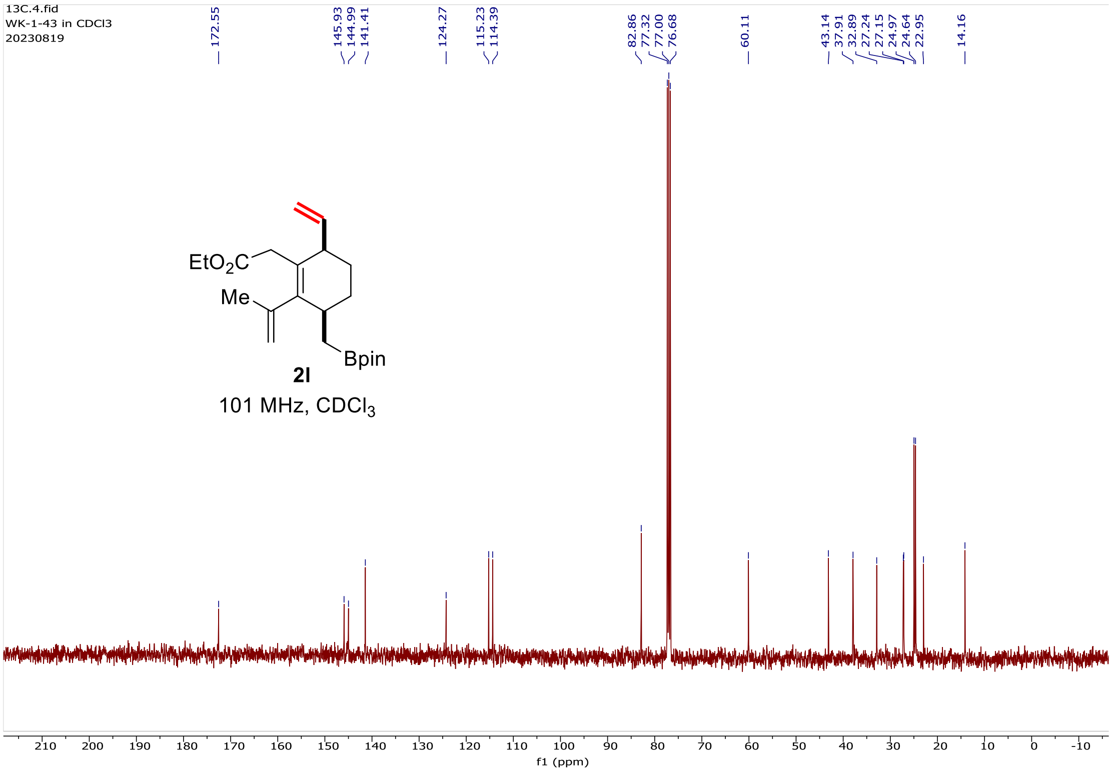
13C.4.fid
WK-1-43 in CDCl3
20230819
172.55
145.93
144.99
141.41
124.27
115.23
114.39
82.86
77.32
77.00
76.68
60.11
43.14
37.91
32.89
27.24
27.15
24.97
24.64
22.95
14.16
EtO2C
Me
Bpin
2l
101 MHz, CDCl3
f1 (ppm)

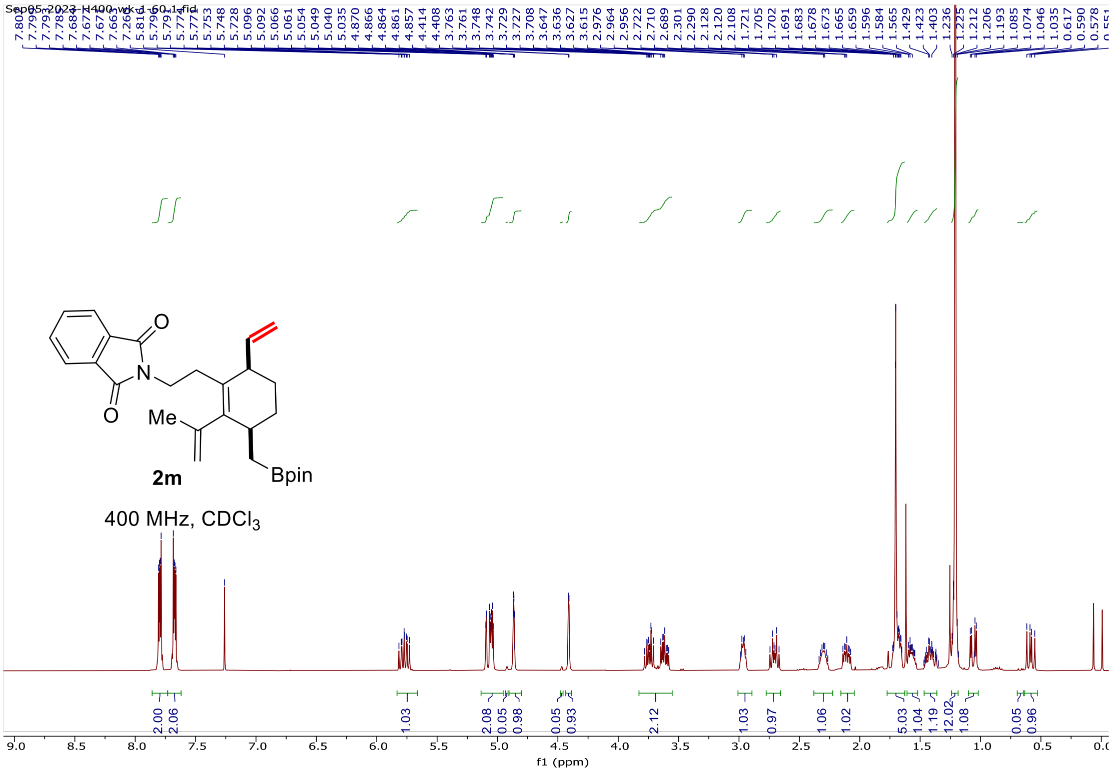
2m
400 MHz, CDCl3
f1 (ppm)

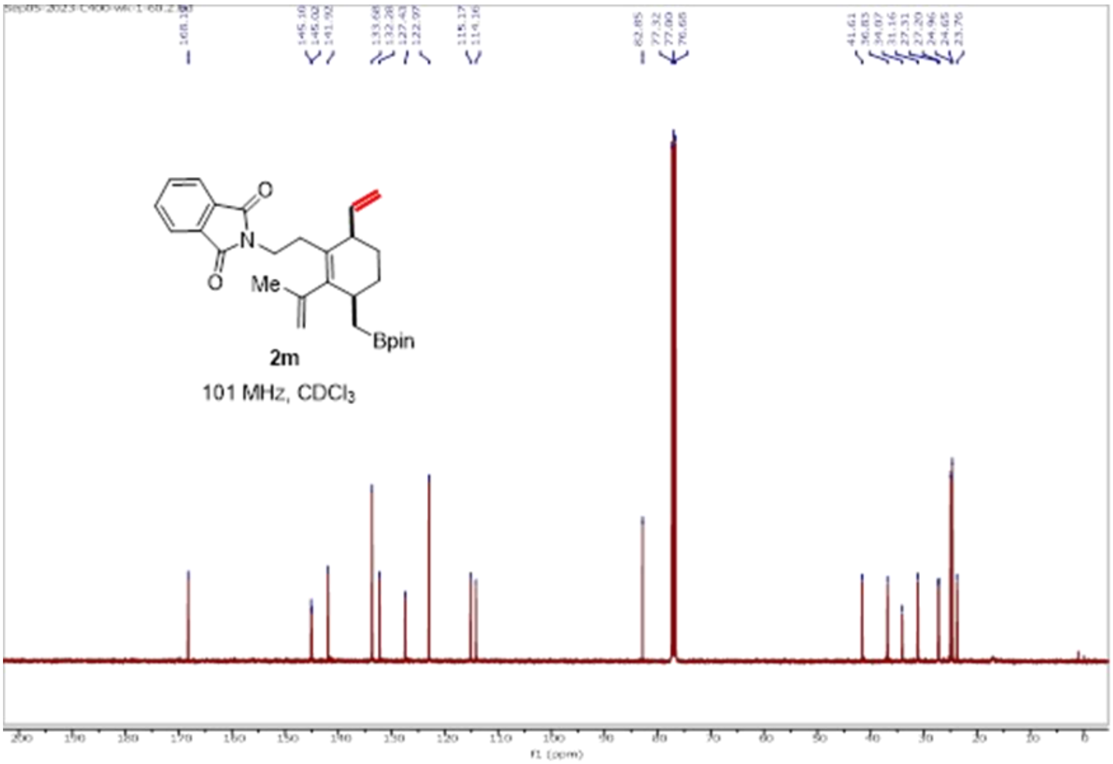
2m
101 MHz, CDCl3

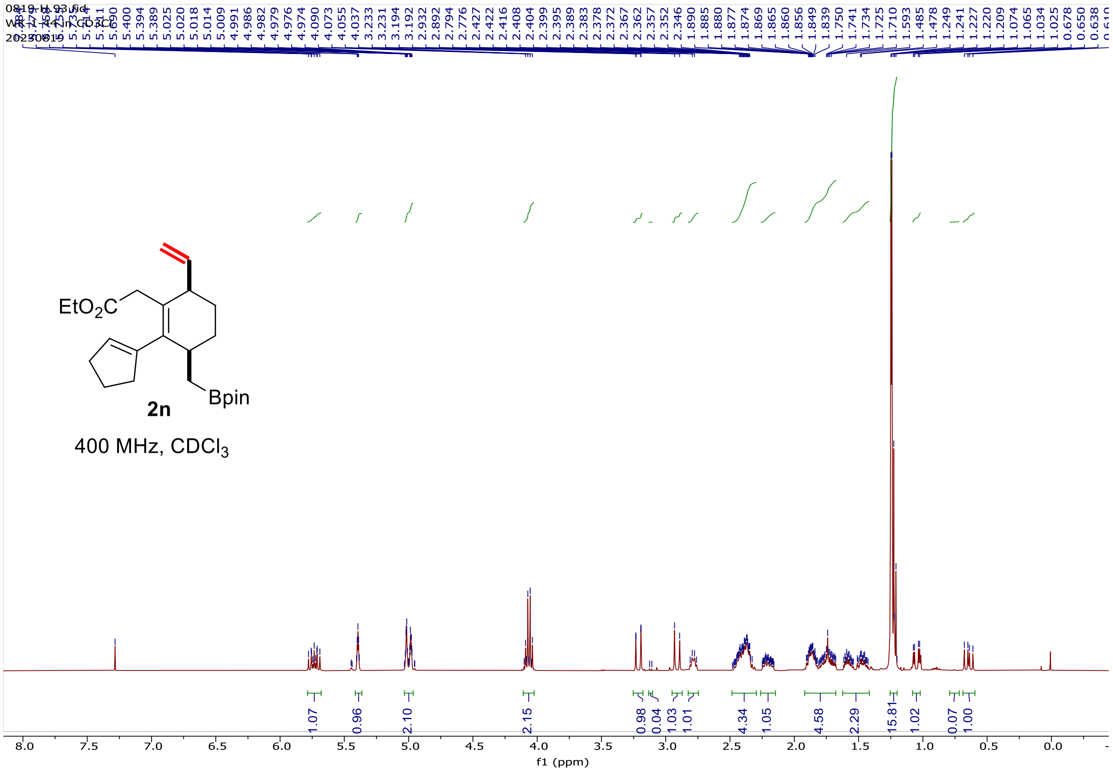
5.400
5.394
5.389
5.025
5.020
5.018
5.014
5.009
4.991
4.986
4.982
4.979
4.976
4.974
4.090
4.073
4.055
4.037
3.233
3.231
3.194
3.192
2.932
2.892
2.794
2.776
2.427
2.422
2.416
2.408
2.404
2.399
2.395
2.389
2.383
2.378
2.372
2.367
2.362
2.357
2.352
2.346
1.890
1.885
1.880
1.877
1.874
1.869
1.865
1.860
1.856
1.849
1.839
1.750
1.741
1.734
1.725
1.710
1.593
1.485
1.478
1.249
1.241
1.227
1.220
1.209
1.074
1.065
1.034
1.025
0.678
0.650
0.638
EtO2C
Bpin
2n
400 MHz, CDCl3
1.07
0.96
2.10
2.15
0.98
0.04
1.03
1.01
4.34
1.05
4.58
2.29
15.81
1.02
0.07
1.00
f1 (ppm)

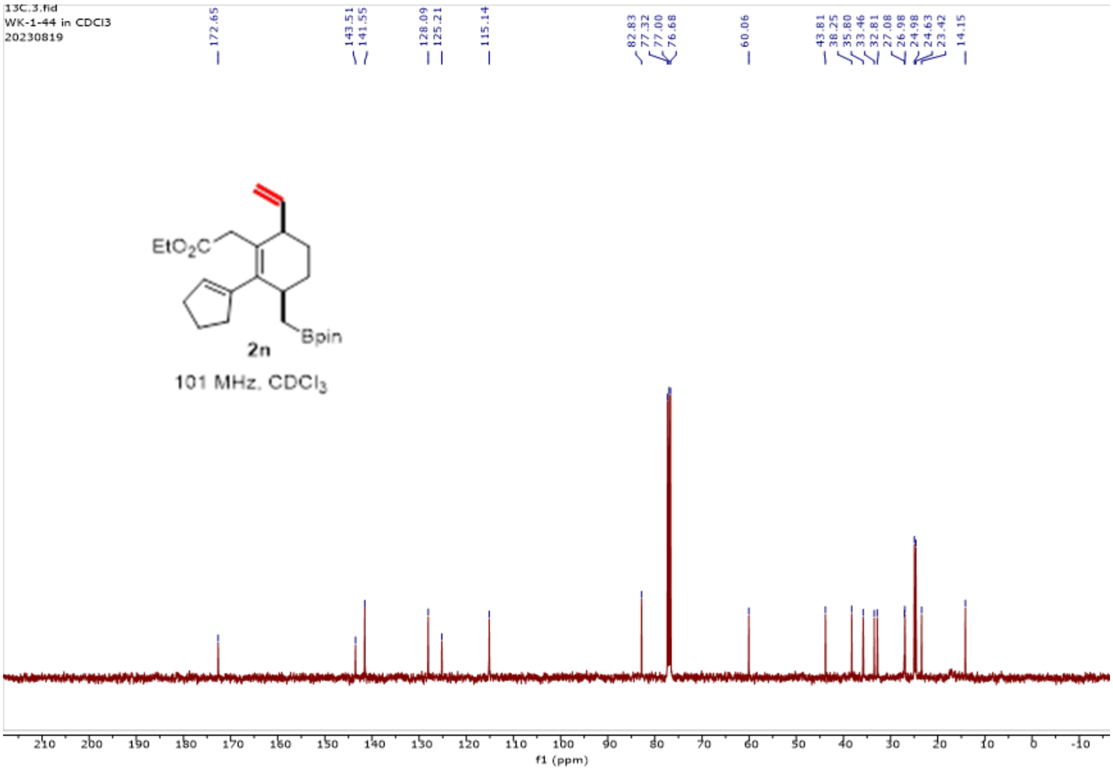
13C.3.fid
WK-1-44 in CDCl3
20230819
172.65
143.51
141.55
128.09
125.21
115.14
82.83
77.32
77.00
76.68
60.06
43.81
38.25
35.80
33.46
32.81
27.08
26.98
24.98
24.63
23.42
14.15
EtO2C
Bpin
2n
101 MHz, CDCl3
f1 (ppm)

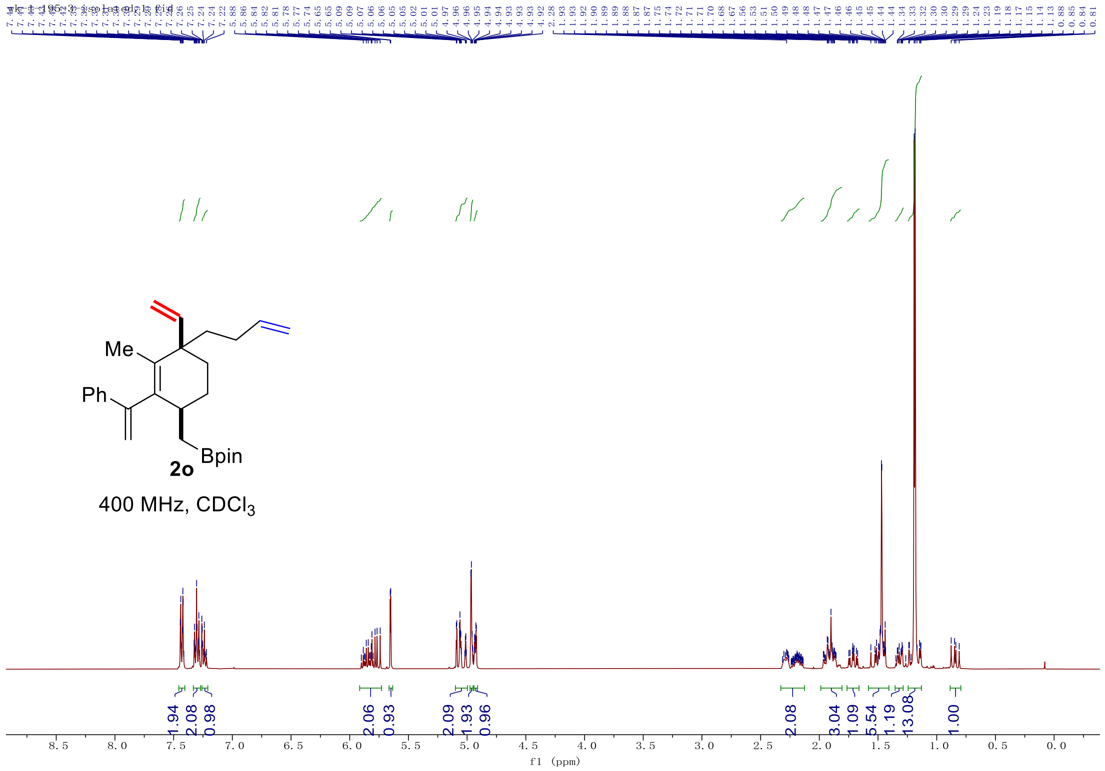
wk-1-195-3-isolated.1.fid
Me
Ph
Bpin
2o
400 MHz, $CDCl_3$
1.94
2.08
0.98
2.06
0.93
2.09
1.93
0.96
2.08
3.04
1.09
5.54
1.19
13.08
1.00
8.5 8.0 7.5 7.0 6.5 6.0 5.5 5.0 4.5 4.0 3.5 3.0 2.5 2.0 1.5 1.0 0.5 0.0
f1 (ppm)

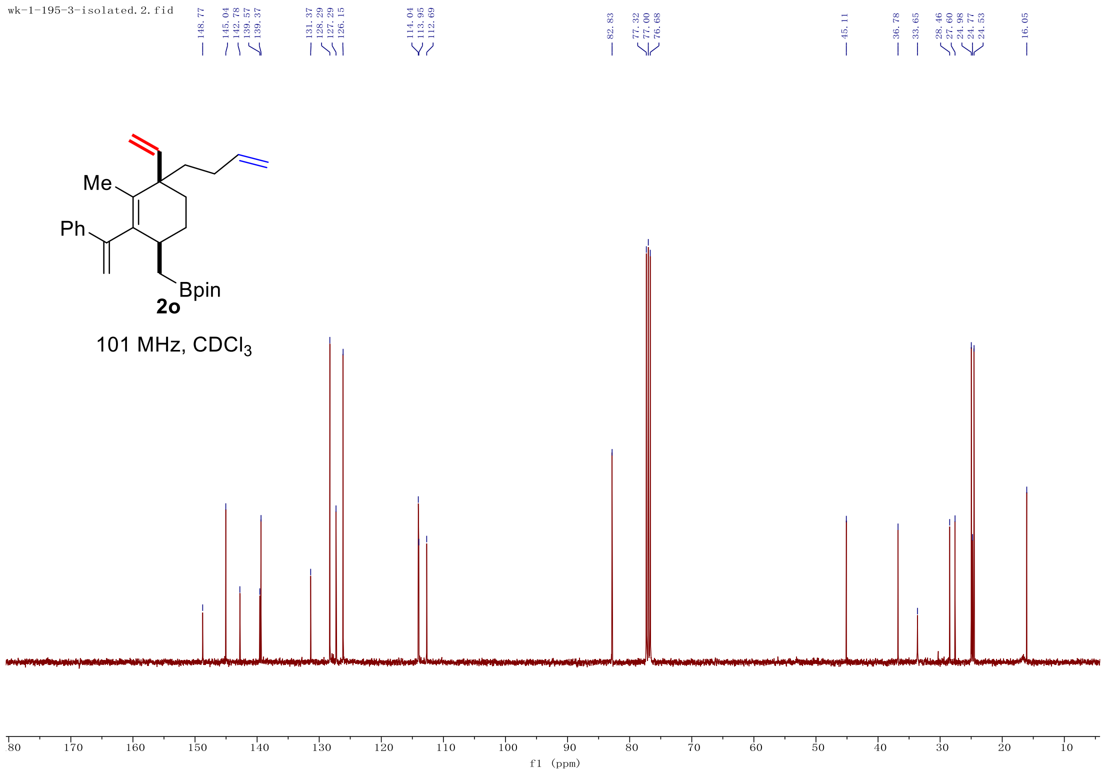
wk-1-195-3-isolated.2.fid
148.77
145.04
142.78
139.57
139.37
131.37
128.29
127.29
126.15
114.04
113.95
112.69
82.83
77.32
77.00
76.68
45.11
36.78
33.65
28.46
27.60
24.98
24.77
24.53
16.05
Me
Ph
Bpin
2o
101 MHz, $CDCl_3$
80 170 160 150 140 130 120 110 100 90 80 70 60 50 40 30 20 10
f1 (ppm)

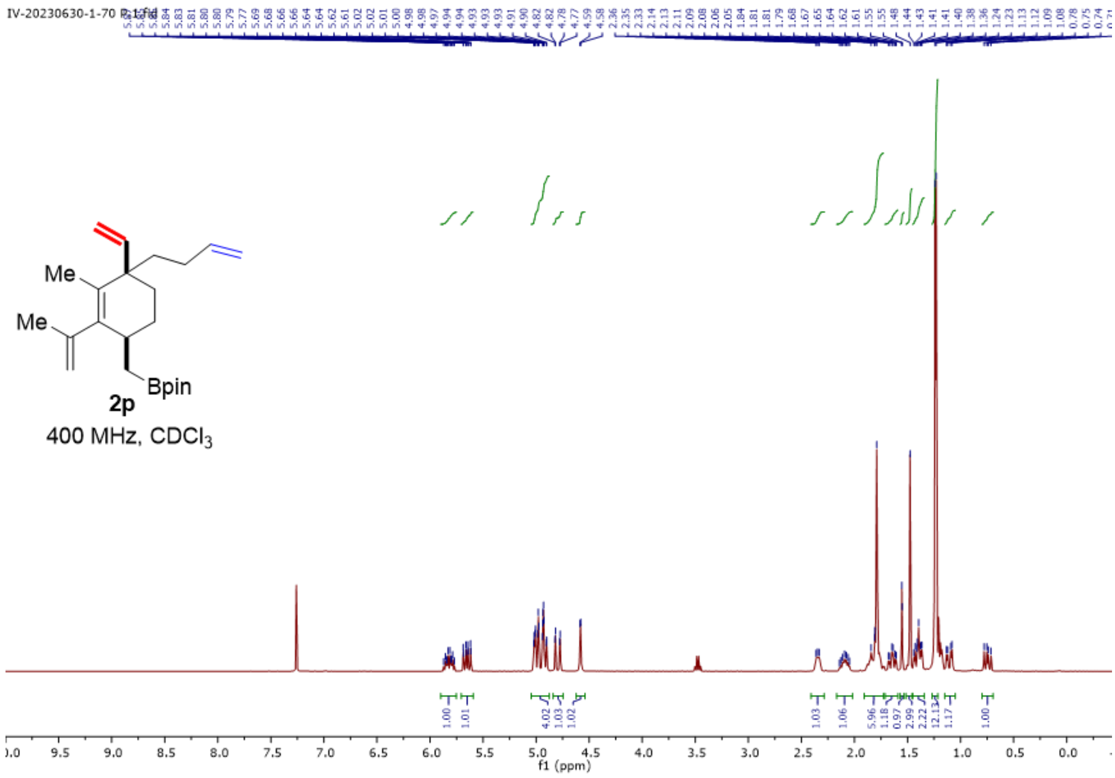
IV-20230630-1-70 P2.1.fid
Me
Me
Bpin
2p
400 MHz, CDCl3
f1 (ppm)

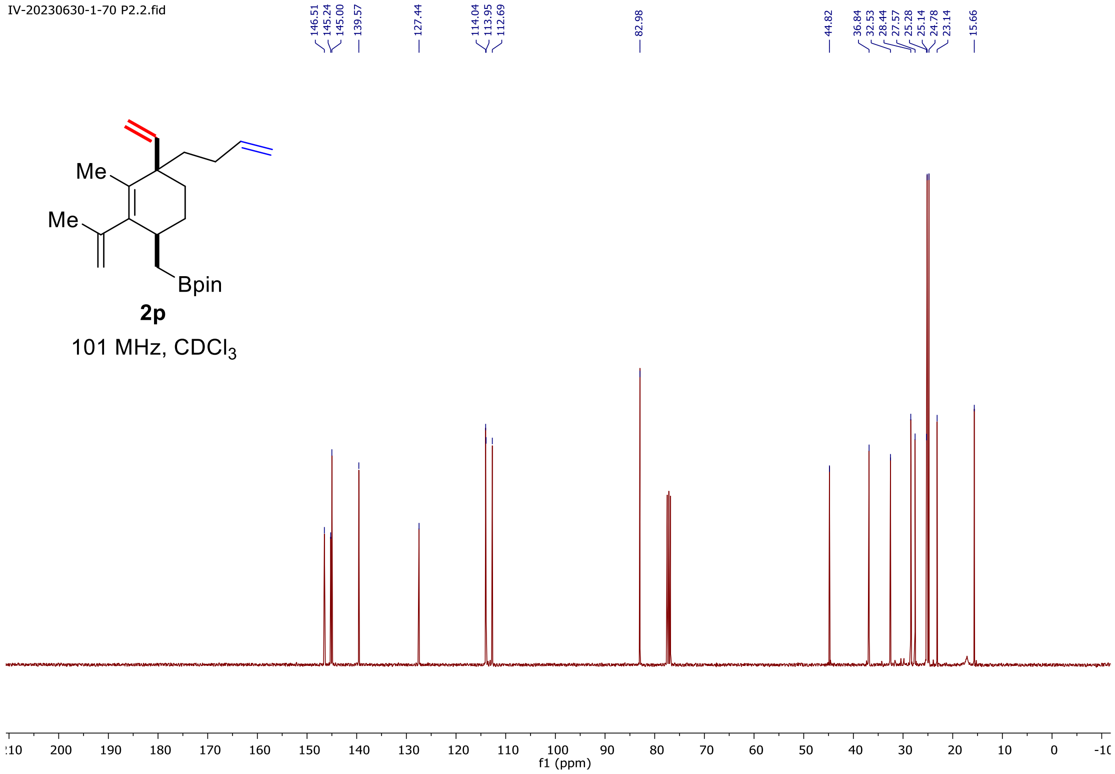
IV-20230630-1-70 P2.2.fid
146.51
145.24
145.00
139.57
127.44
114.04
113.95
112.69
82.98
44.82
36.84
32.53
28.44
27.57
25.28
25.14
24.78
23.14
15.66
Me
Me
Bpin
2p
101 MHz, CDCl3
f1 (ppm)

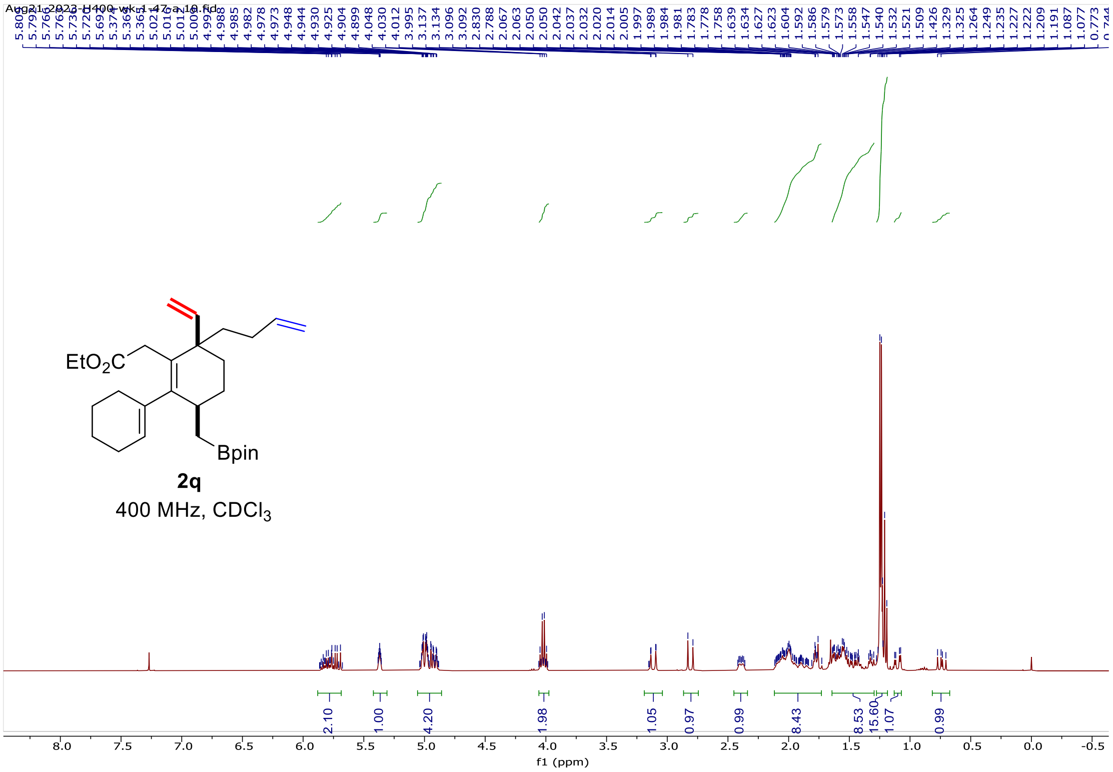
Aug21-2023-H400-wk-1-47-a.10.fid
5.805 5.791 5.763 5.734 5.722 5.693 5.374 5.363 5.024 5.011 5.006 4.997 4.988 4.985 4.982 4.978 4.973 4.948 4.944 4.930 4.925 4.904 4.899 4.048 4.030 4.012 3.995 3.137 3.134 3.096 3.092 2.830 2.788 2.067 2.063 2.050 2.042 2.037 2.032 2.020 2.014 2.005 1.997 1.989 1.984 1.981 1.783 1.778 1.758 1.639 1.634 1.627 1.623 1.604 1.592 1.586 1.579 1.573 1.558 1.547 1.540 1.532 1.521 1.509 1.426 1.329 1.325 1.264 1.249 1.235 1.227 1.222 1.209 1.191 1.087 1.077 0.773
EtO2C
Bpin
2q
400 MHz, CDCl3
2.10
1.00
4.20
1.98
1.05
0.97
0.99
8.43
8.53
15.60
1.07
0.99
f1 (ppm)

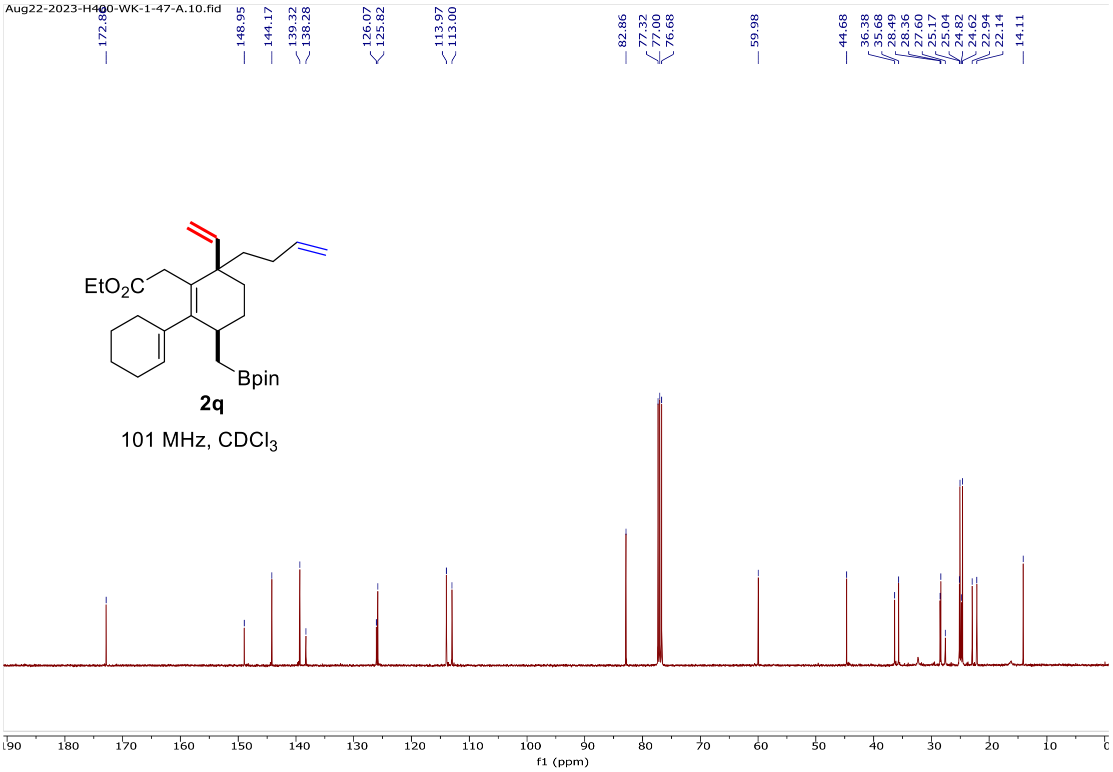
Aug22-2023-H400-WK-1-47-A.10.fid
172.80
148.95
144.17
139.32
138.28
126.07
125.82
113.97
113.00
82.86
77.32
77.00
76.68
59.98
44.68
36.38
35.68
28.49
28.36
27.60
25.17
25.04
24.82
24.62
22.94
22.14
14.11
EtO2C
Bpin
2q
101 MHz, CDCl3
f1 (ppm)

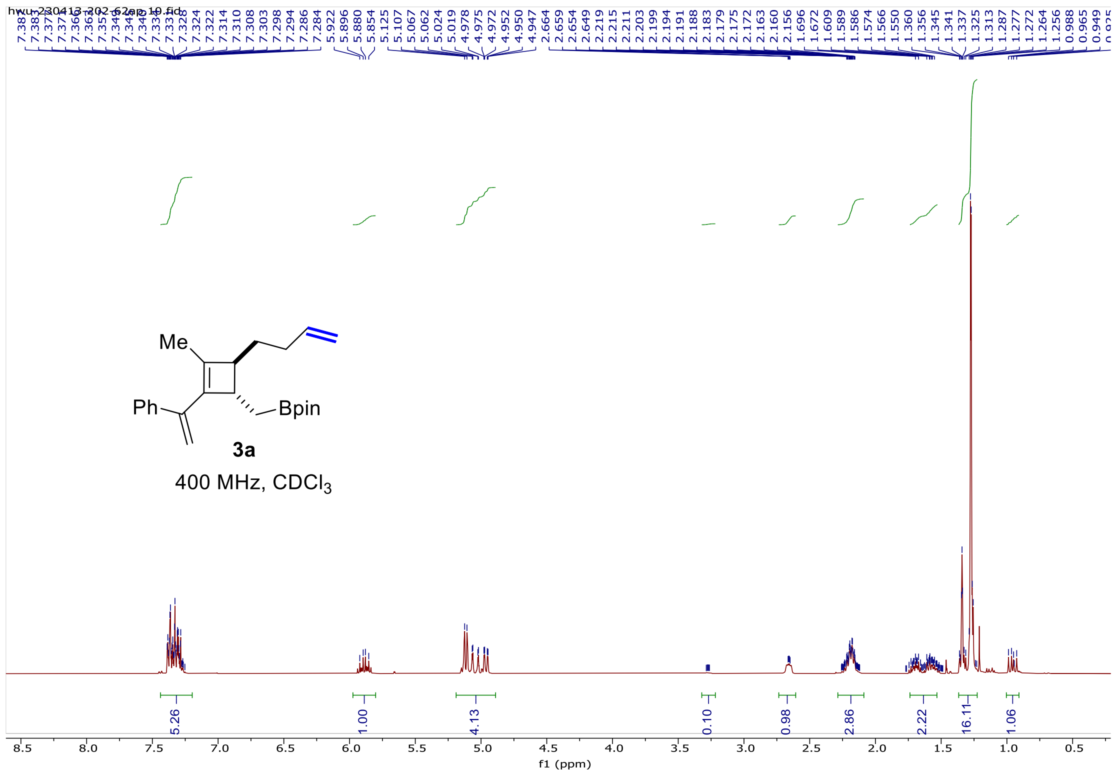
hwu-230413-202-62ap.10.fid
Me
Ph
Bpin
3a
400 MHz, CDCl3
f1 (ppm)

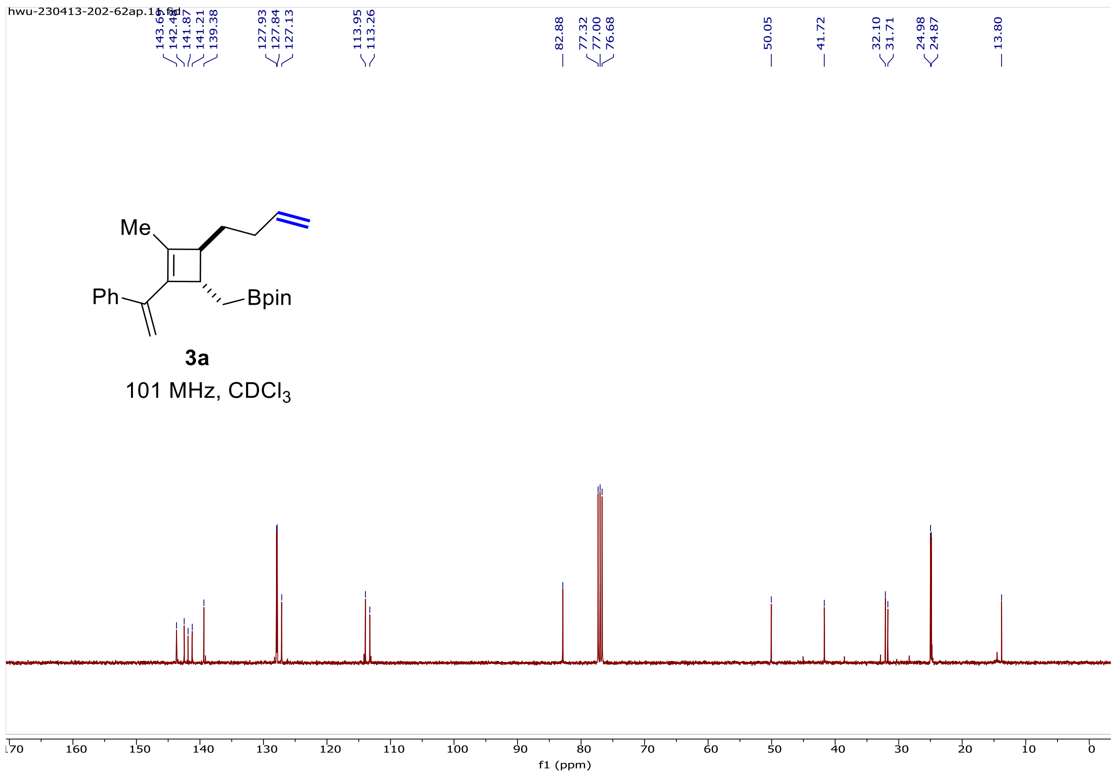
hwu-230413-202-62ap.11.fid
143.65
142.46
141.87
141.21
139.38
127.93
127.84
127.13
113.95
113.26
82.88
77.32
77.00
76.68
50.05
41.72
32.10
31.71
24.98
24.87
13.80
Me
Ph
Bpin
3a
101 MHz, CDCl3
f1 (ppm)

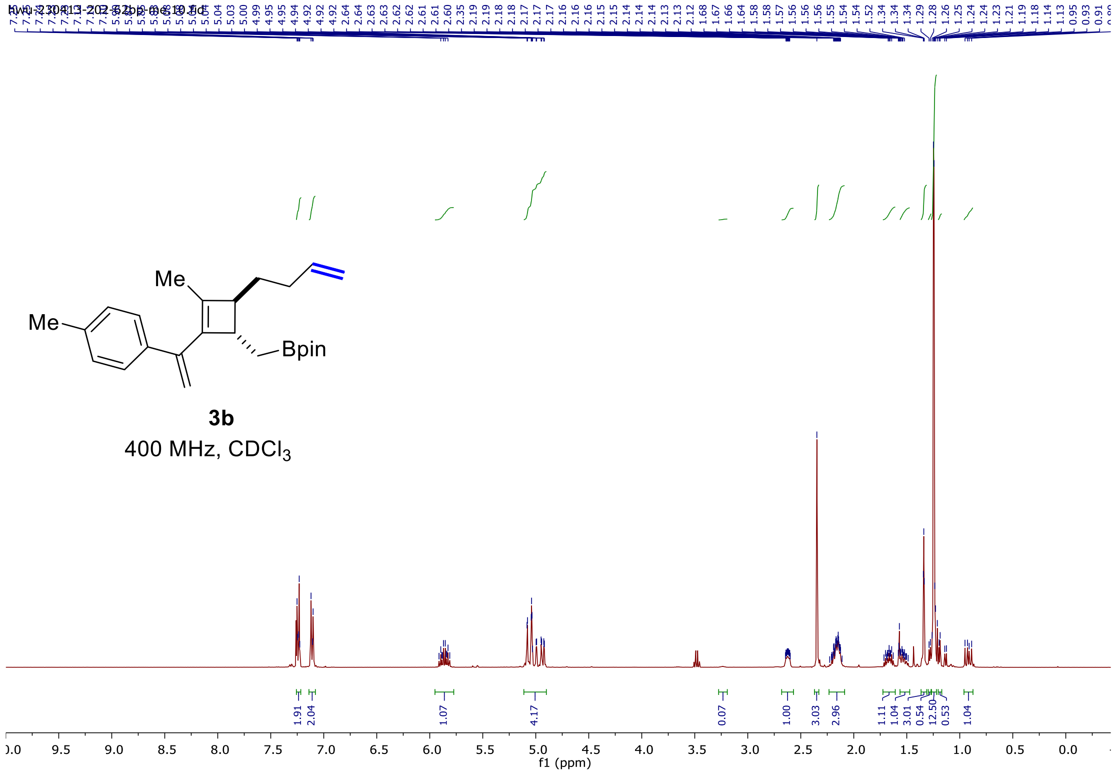
Me
Me
Bpin
3b
400 MHz, CDCl$_3$
1.91
2.04
1.07
4.17
0.07
1.00
3.03
2.96
1.11
1.04
3.01
0.54
12.50
0.53
1.04
f1 (ppm)

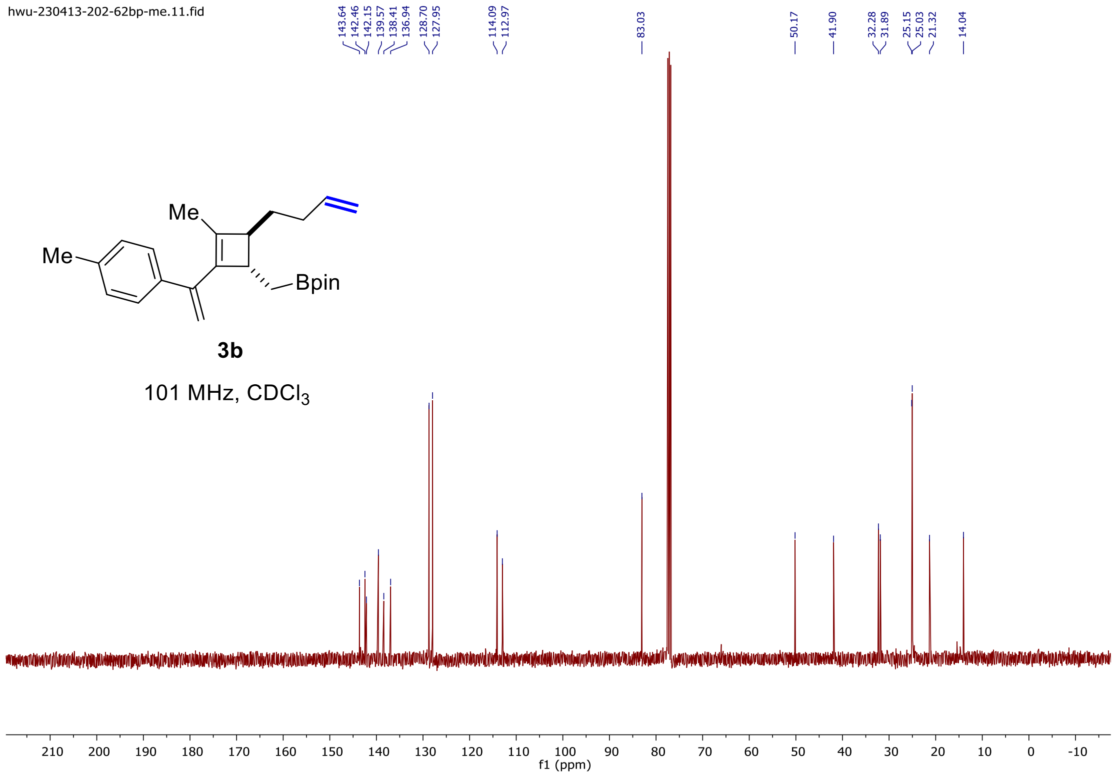
hwu-230413-202-62bp-me.11.fid
143.64
142.46
142.15
139.57
138.41
136.94
128.70
127.95
114.09
112.97
83.03
50.17
41.90
32.28
31.89
25.15
25.03
21.32
14.04
Me
Me
Bpin
3b
101 MHz, CDCl$_3$
f1 (ppm)

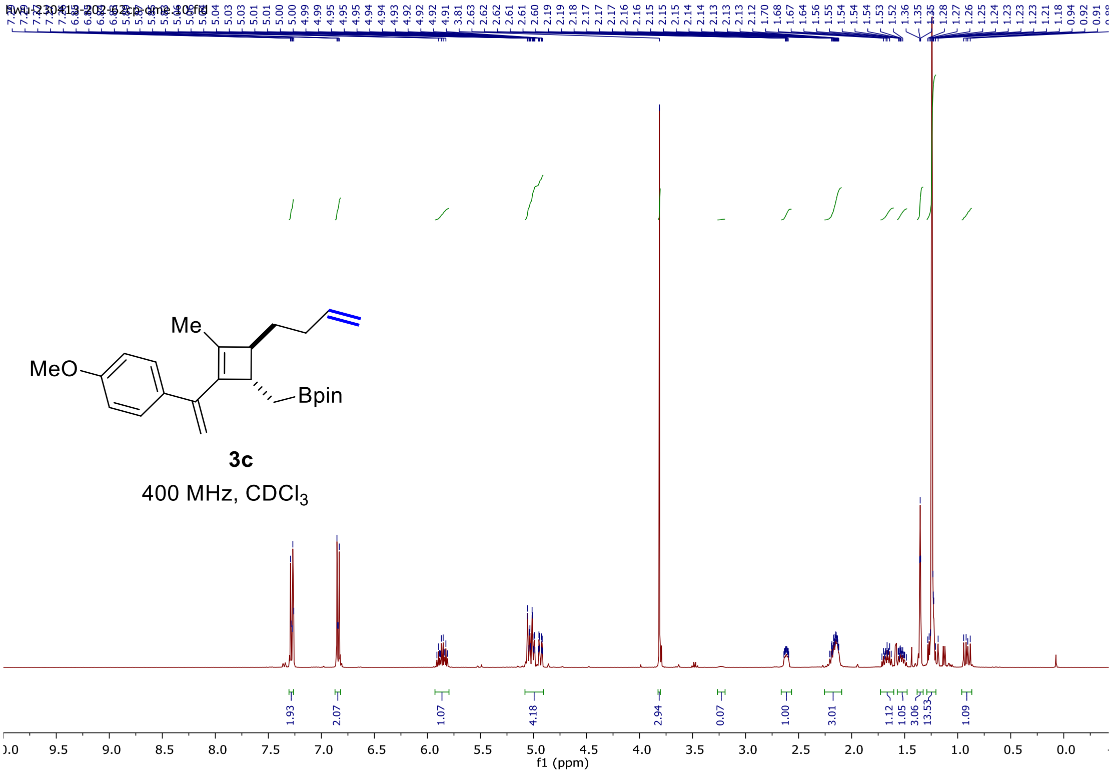
Me
MeO
Bpin
3c
400 MHz, CDCl$_3$
f1 (ppm)

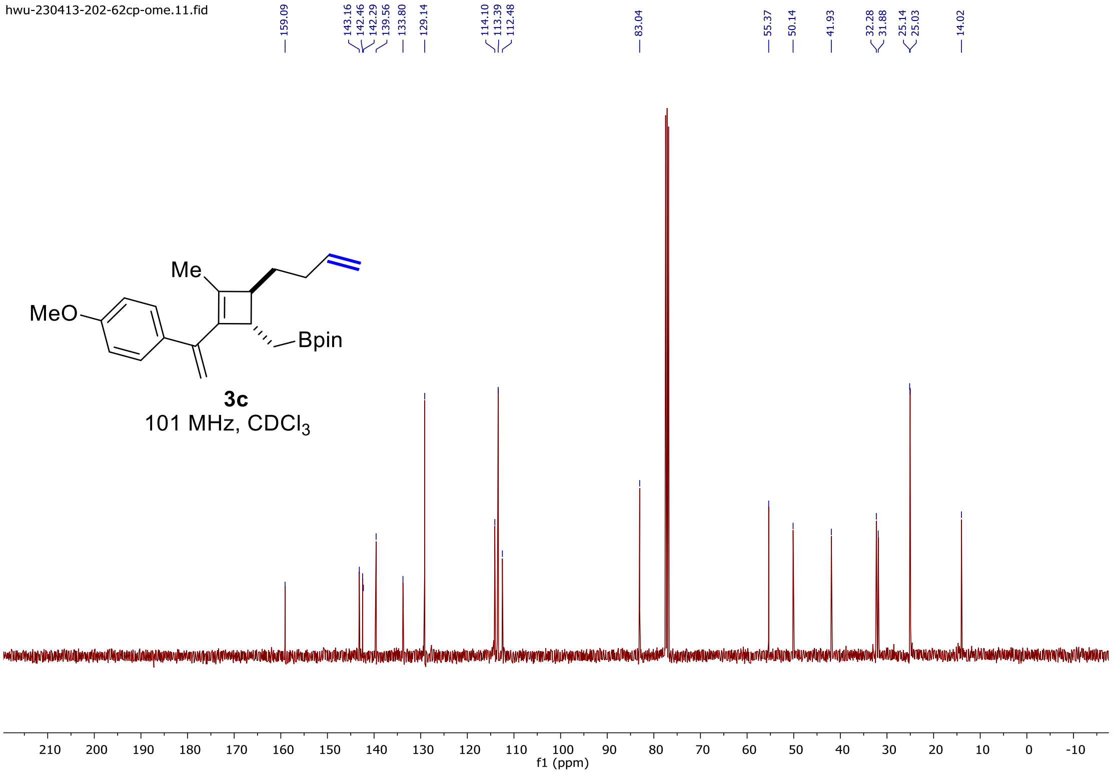
hwu-230413-202-62cp-ome.11.fid
159.09
143.16
142.46
142.29
139.56
133.80
129.14
114.10
113.39
112.48
83.04
55.37
50.14
41.93
32.28
31.88
25.14
25.03
14.02
Me
MeO
Bpin
3c
101 MHz, CDCl$_3$
f1 (ppm)

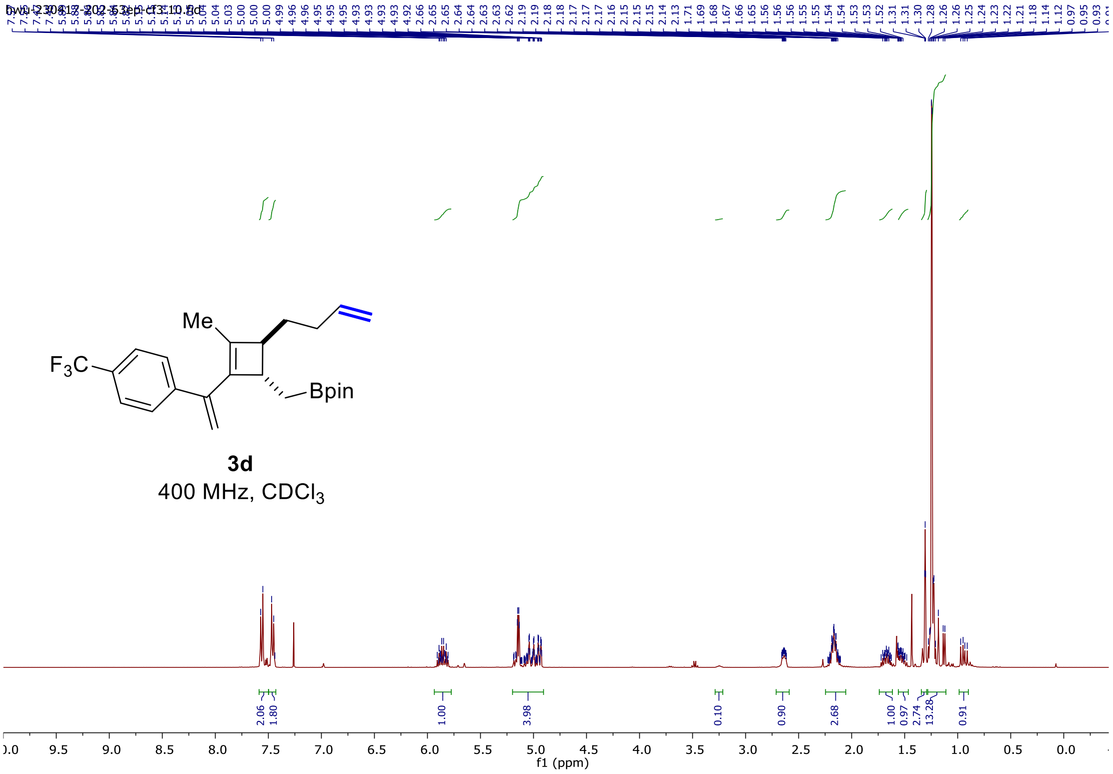

Me
F_3C
Bpin
3d
400 MHz, $CDCl_3$
2.06
1.80
1.00
3.98
0.10
0.90
2.68
1.00
0.97
2.74
13.28
0.91
f1 (ppm)

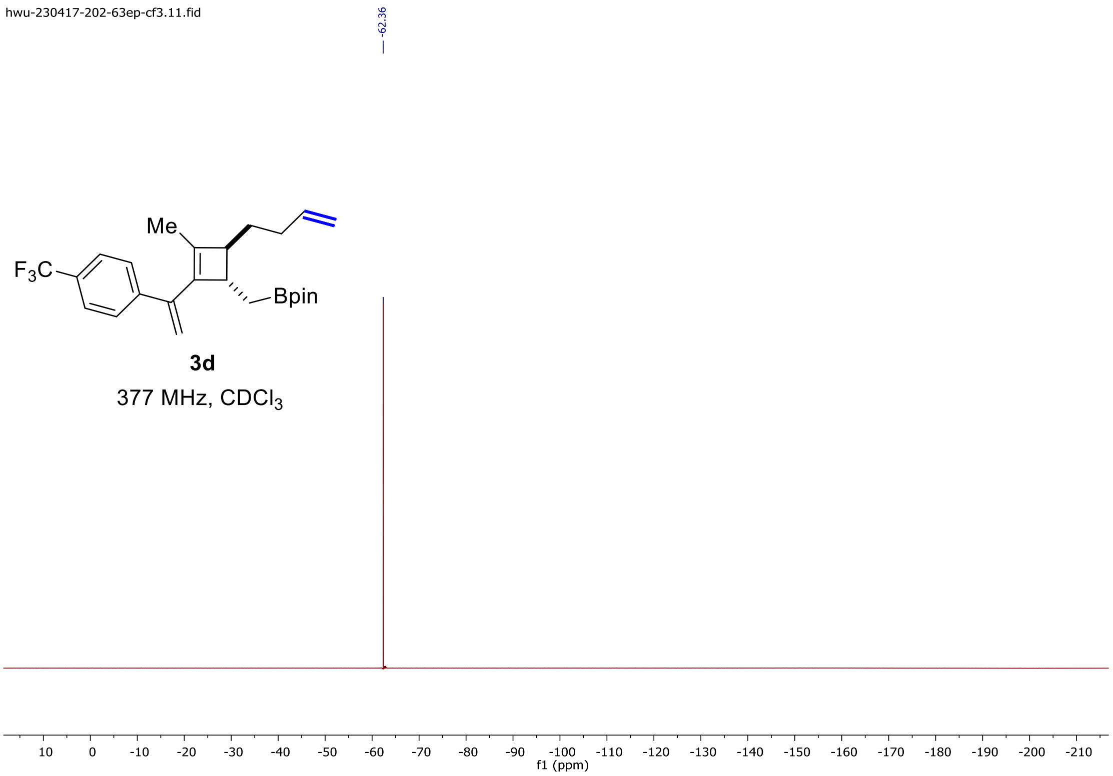

hwu-230417-202-63ep-cf3.11.fid
-62.36
Me
F_3C
Bpin
3d
377 MHz, $CDCl_3$
f1 (ppm)

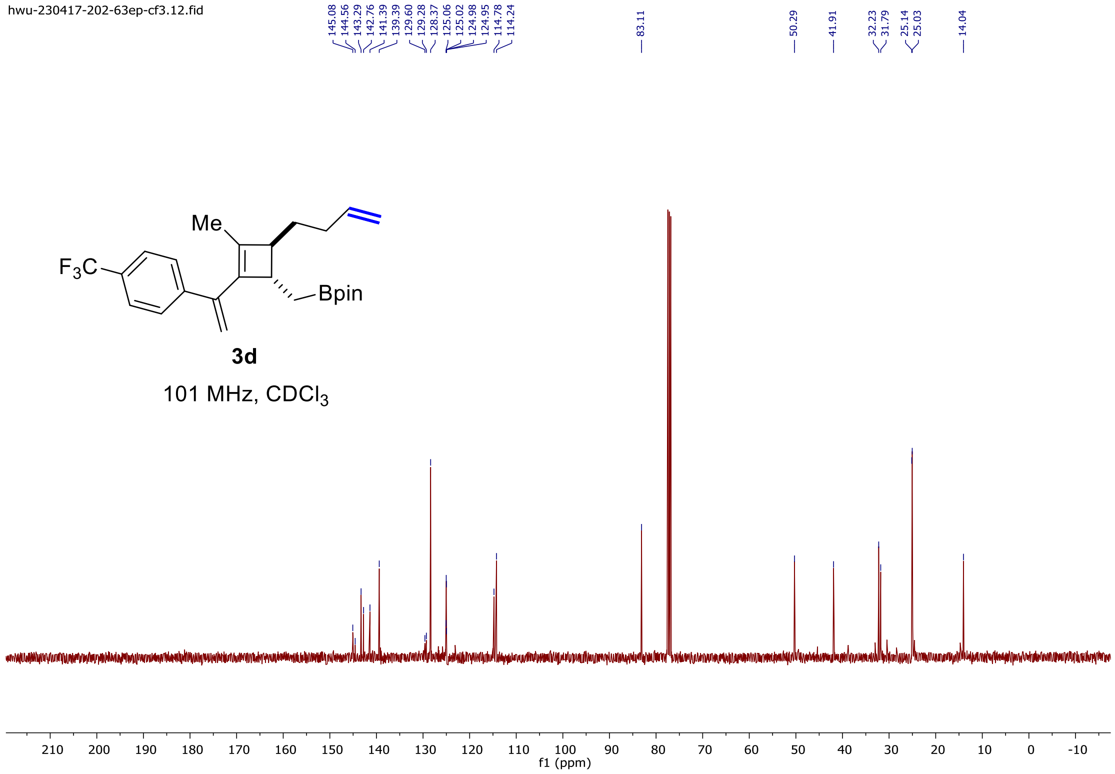
hwu-230417-202-63ep-cf3.12.fid
145.08
144.56
143.29
142.76
141.39
139.39
129.60
129.28
128.37
125.06
125.02
124.98
124.95
114.78
114.24
83.11
50.29
41.91
32.23
31.79
25.14
25.03
14.04
Me
F3C
Bpin
3d
101 MHz, CDCl3
f1 (ppm)

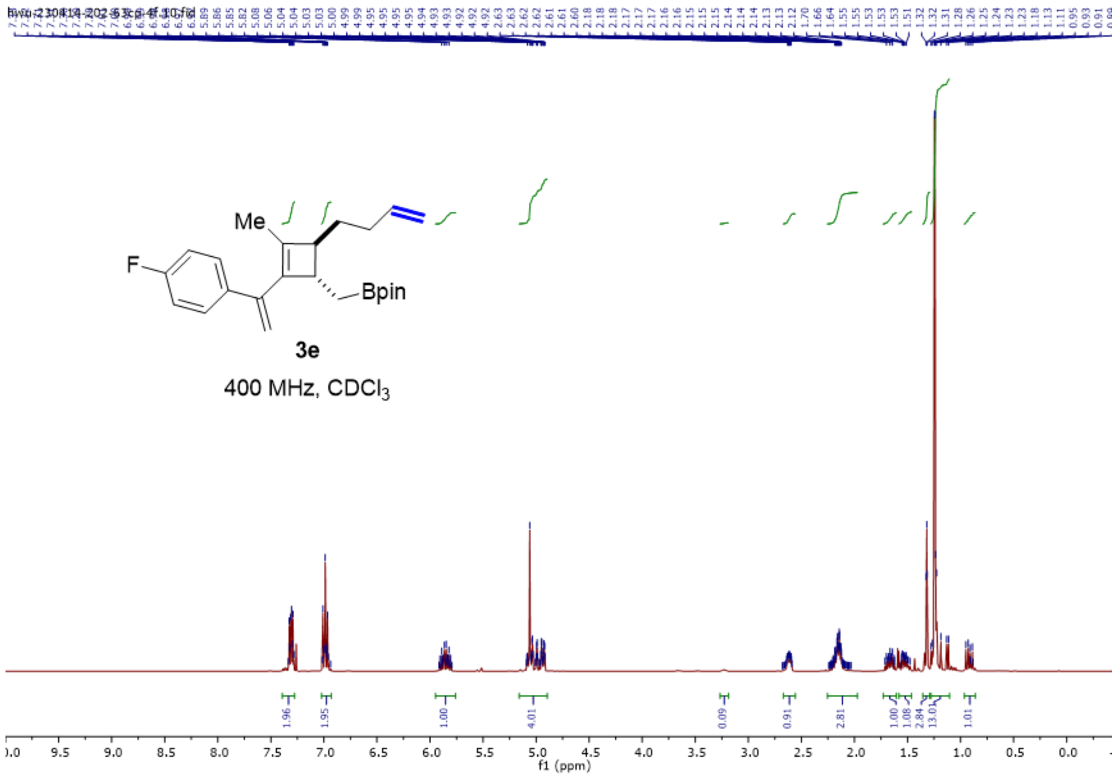
Me
F
Bpin
3e
400 MHz, CDCl3
1.96
1.95
1.00
4.01
0.09
0.91
2.81
1.00
1.08
2.84
13.01
1.01
f1 (ppm)

hwu-230414-202-63cp-4f.11.fid

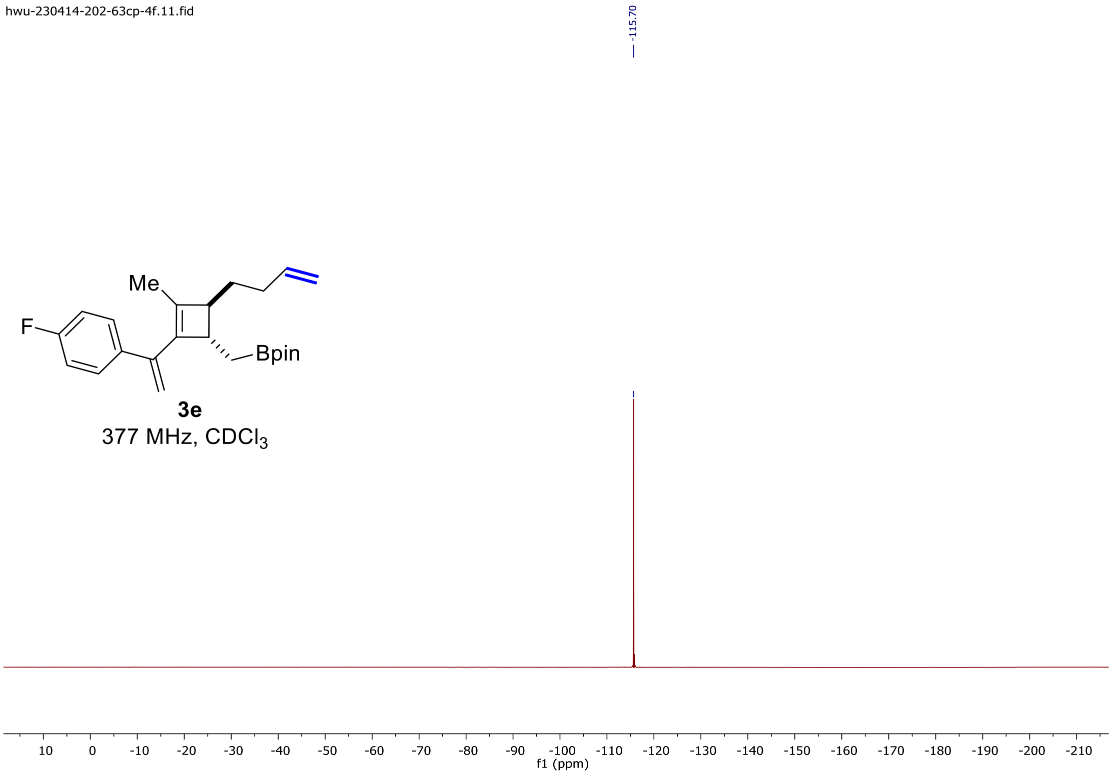

hwu-230414-202-63cp-4f.12.fid

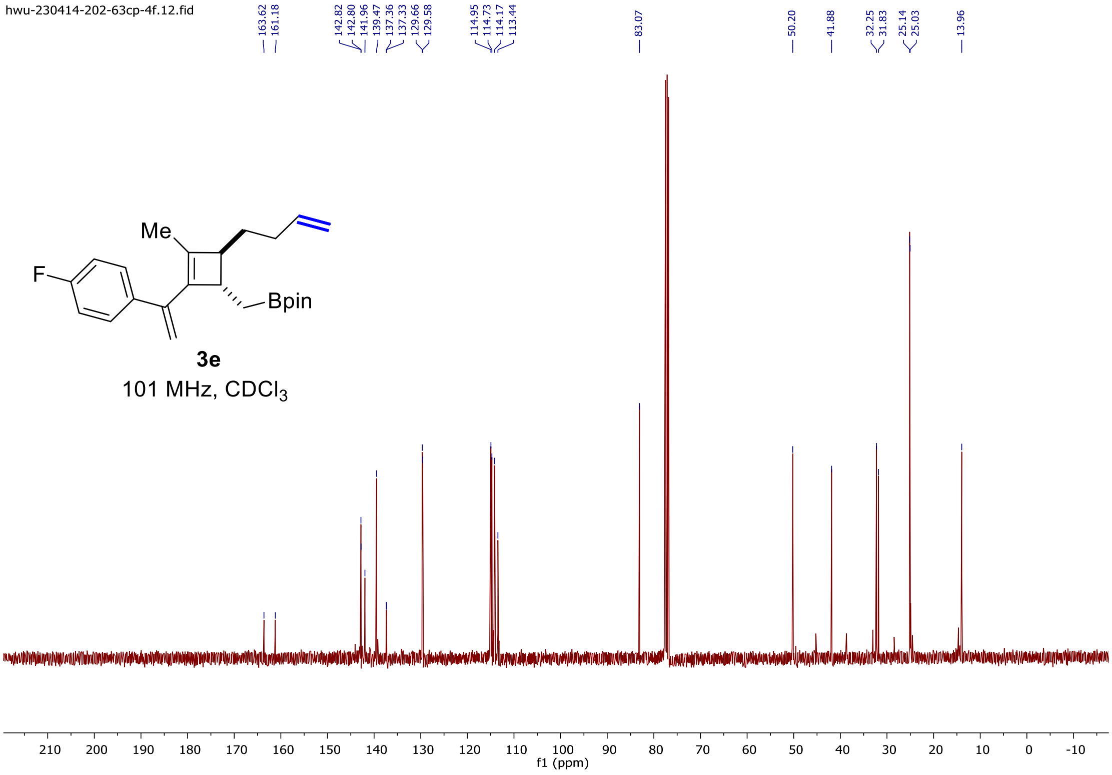

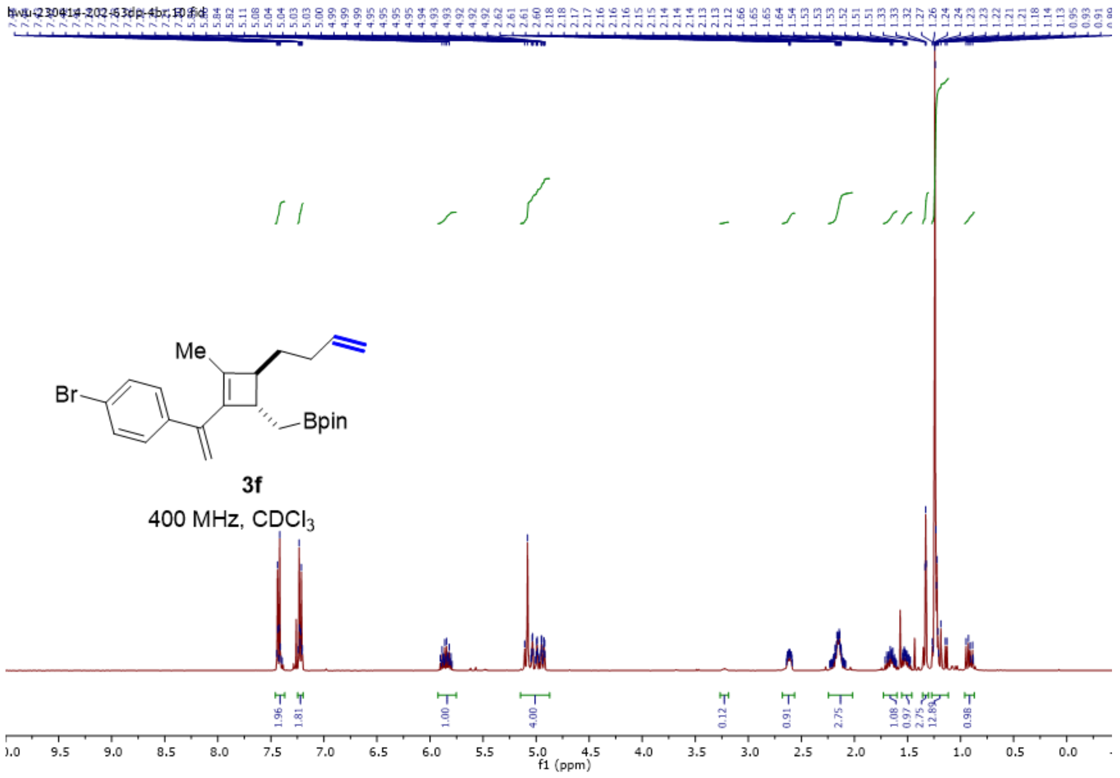
hwu-230414-202-63dp-4br.10.fid
Me
Br
Bpin
3f
400 MHz, CDCl3
1.96
1.81
1.00
4.00
0.12
0.91
2.75
1.08
0.97
2.75
12.89
0.98
f1 (ppm)

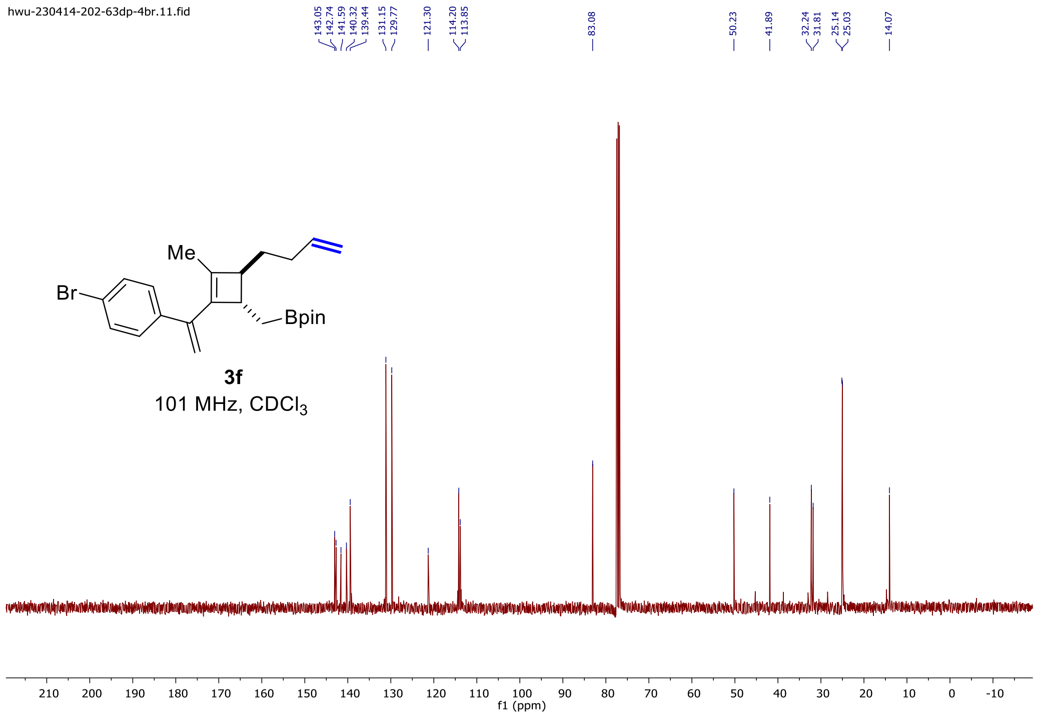
hwu-230414-202-63dp-4br.11.fid
143.05
142.74
141.59
140.32
139.44
131.15
129.77
121.30
114.20
113.85
83.08
50.23
41.89
32.24
31.81
25.14
25.03
14.07
Me
Br
Bpin
3f
101 MHz, CDCl3
f1 (ppm)

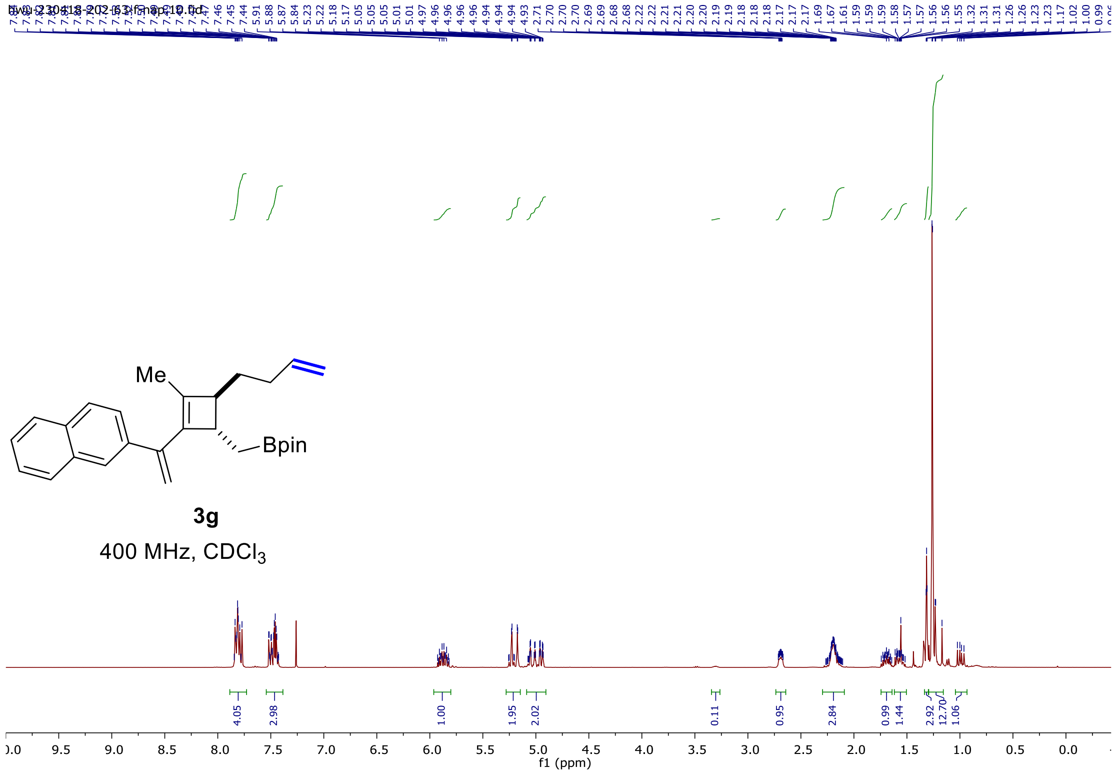
hwu-230418-202-63-f-nap.10.fid
Me
Bpin
3g
400 MHz, CDCl3
f1 (ppm)

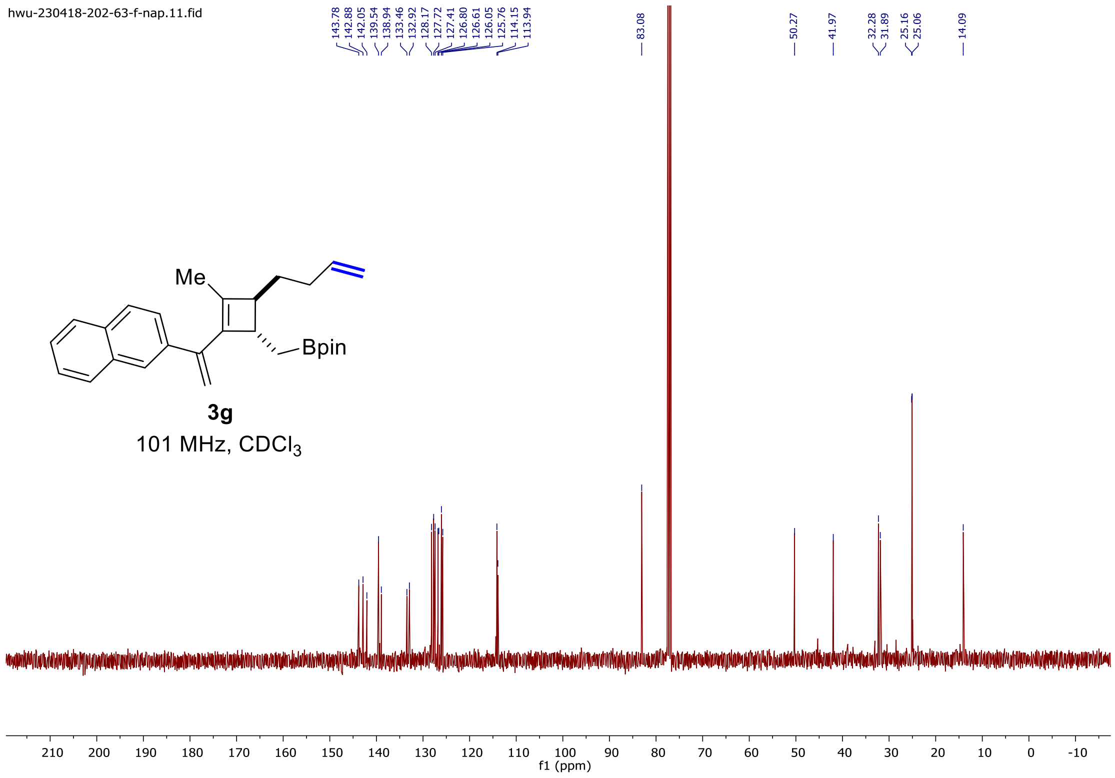
hwu-230418-202-63-f-nap.11.fid
143.78
142.88
142.05
139.54
138.94
133.46
132.92
128.17
127.72
127.41
126.80
126.61
126.05
125.76
114.15
113.94
83.08
50.27
41.97
32.28
31.89
25.16
25.06
14.09
Me
Bpin
3g
101 MHz, CDCl3
f1 (ppm)

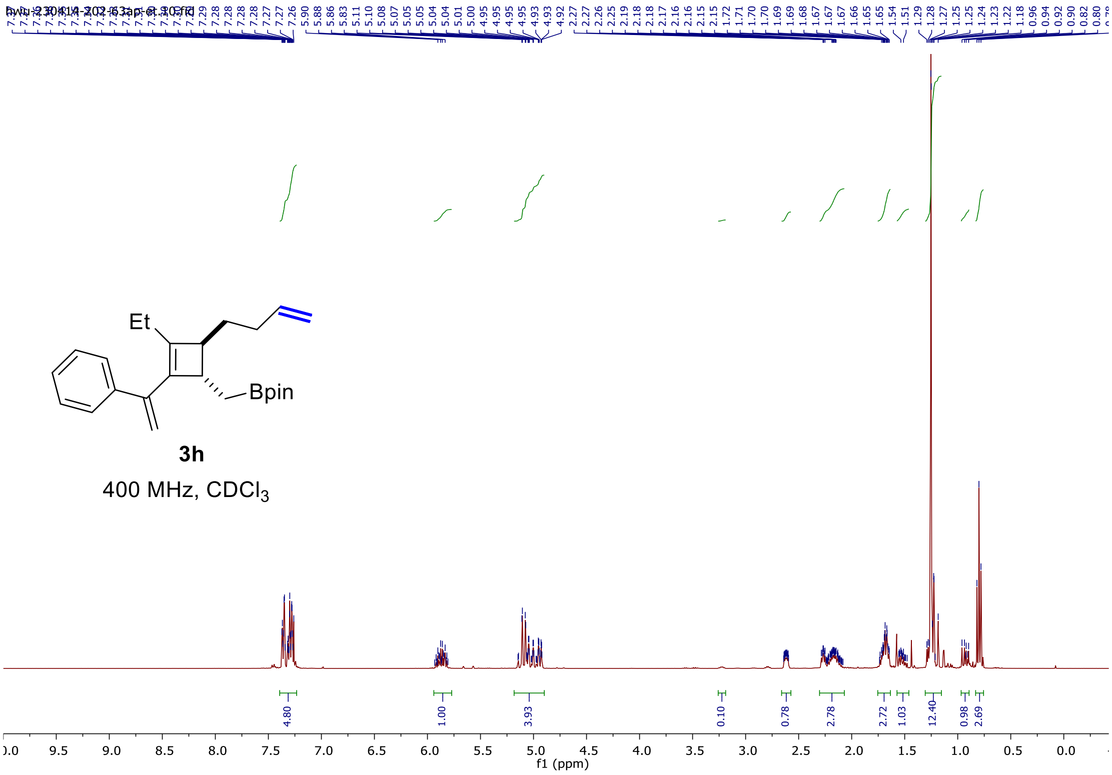
Et
Bpin
3h
400 MHz, CDCl$_3$
f1 (ppm)

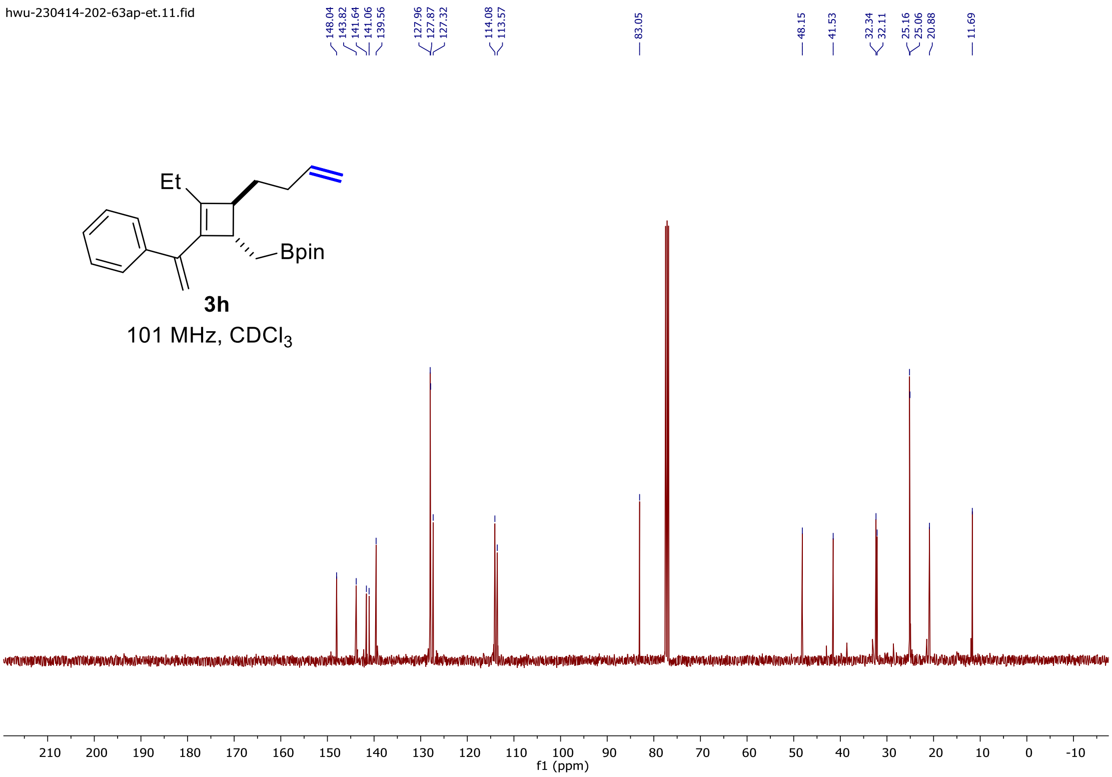
hwu-230414-202-63ap-et.11.fid
Et
Bpin
3h
101 MHz, CDCl$_3$
f1 (ppm)

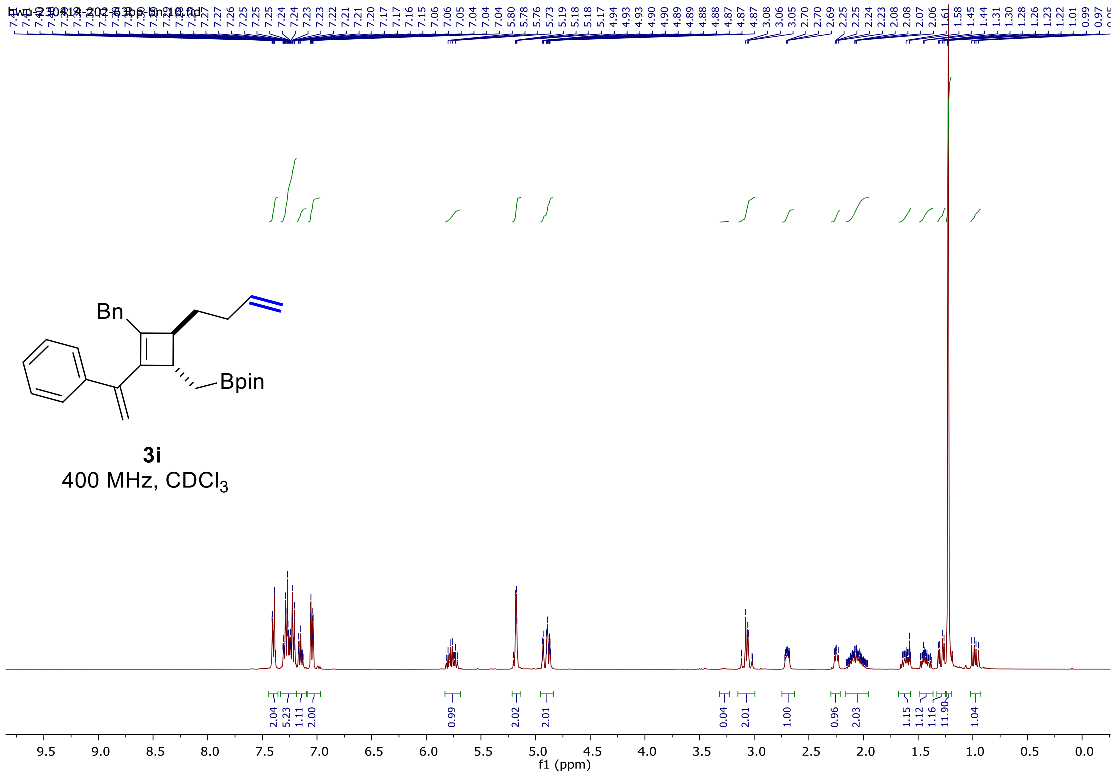
Bn
Bpin
3i
400 MHz, CDCl$_3$
2.04
5.23
1.11
2.00
0.99
2.02
2.01
0.04
2.01
1.00
0.96
2.03
1.15
1.12
1.16
11.90
1.04
f1 (ppm)

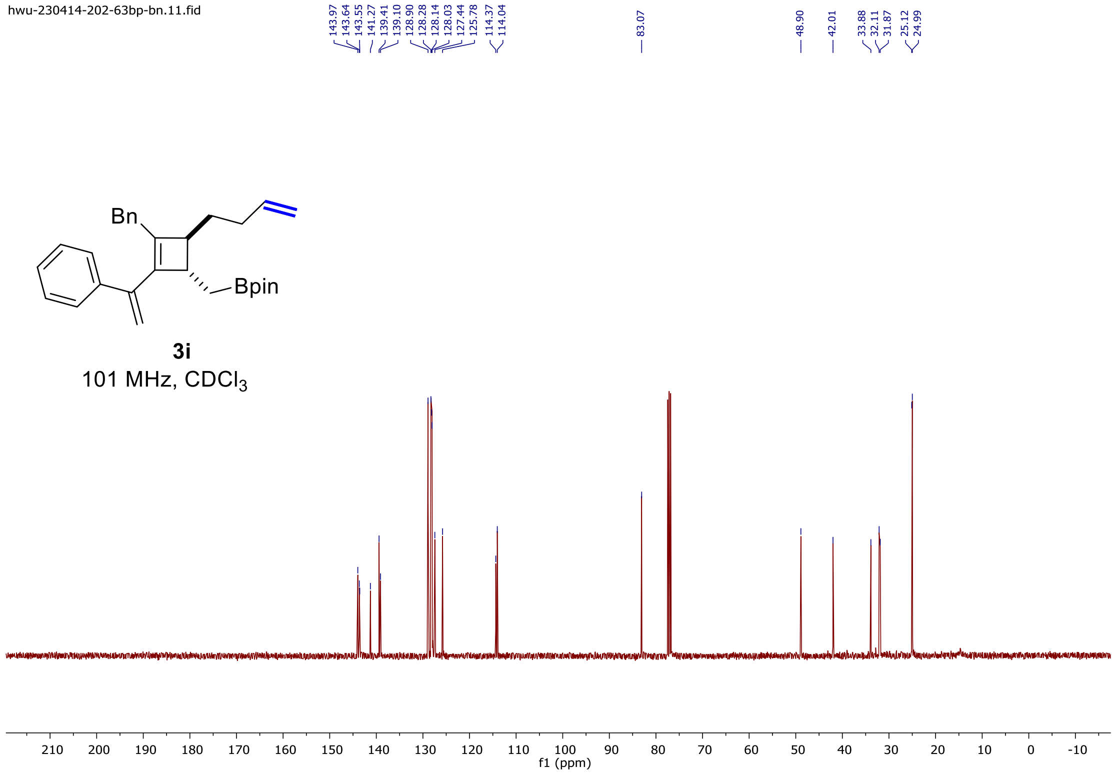
hwu-230414-202-63bp-bn.11.fid
143.97
143.64
143.55
141.27
139.41
139.10
128.90
128.28
128.14
128.03
127.44
125.78
114.37
114.04
83.07
48.90
42.01
33.88
32.11
31.87
25.12
24.99
Bn
Bpin
3i
101 MHz, CDCl$_3$
f1 (ppm)

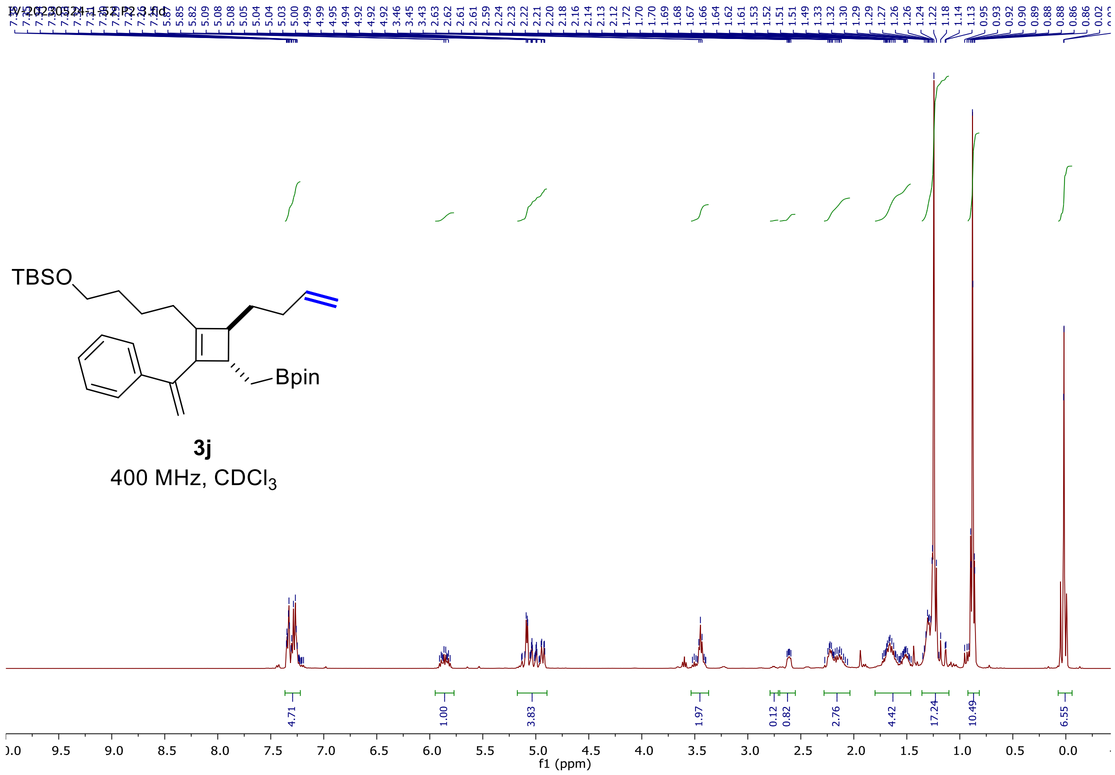
TBSO
Bpin
3j
400 MHz, CDCl3
4.71
1.00
3.83
1.97
0.12
0.82
2.76
4.42
17.24
10.49
6.55
f1 (ppm)

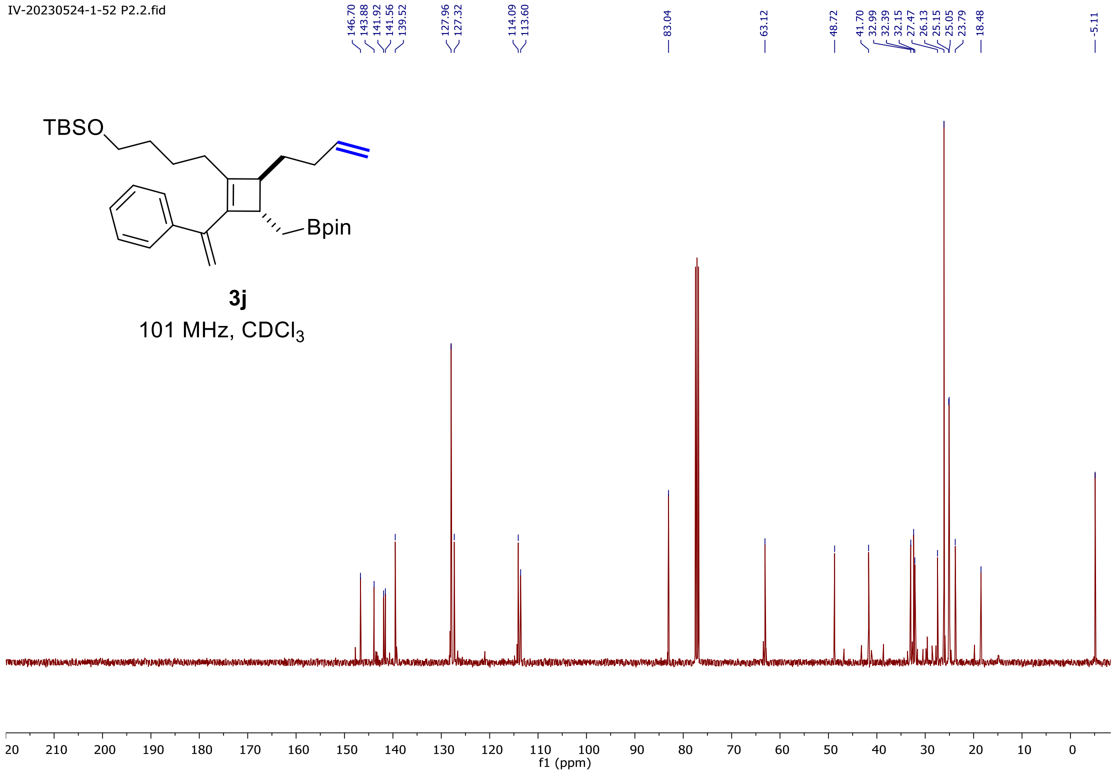
IV-20230524-1-52 P2.2.fid
146.70
143.88
141.92
141.56
139.52
127.96
127.32
114.09
113.60
83.04
63.12
48.72
41.70
32.99
32.39
32.15
27.47
26.13
25.15
25.05
23.79
18.48
-5.11
TBSO
Bpin
3j
101 MHz, CDCl3
f1 (ppm)

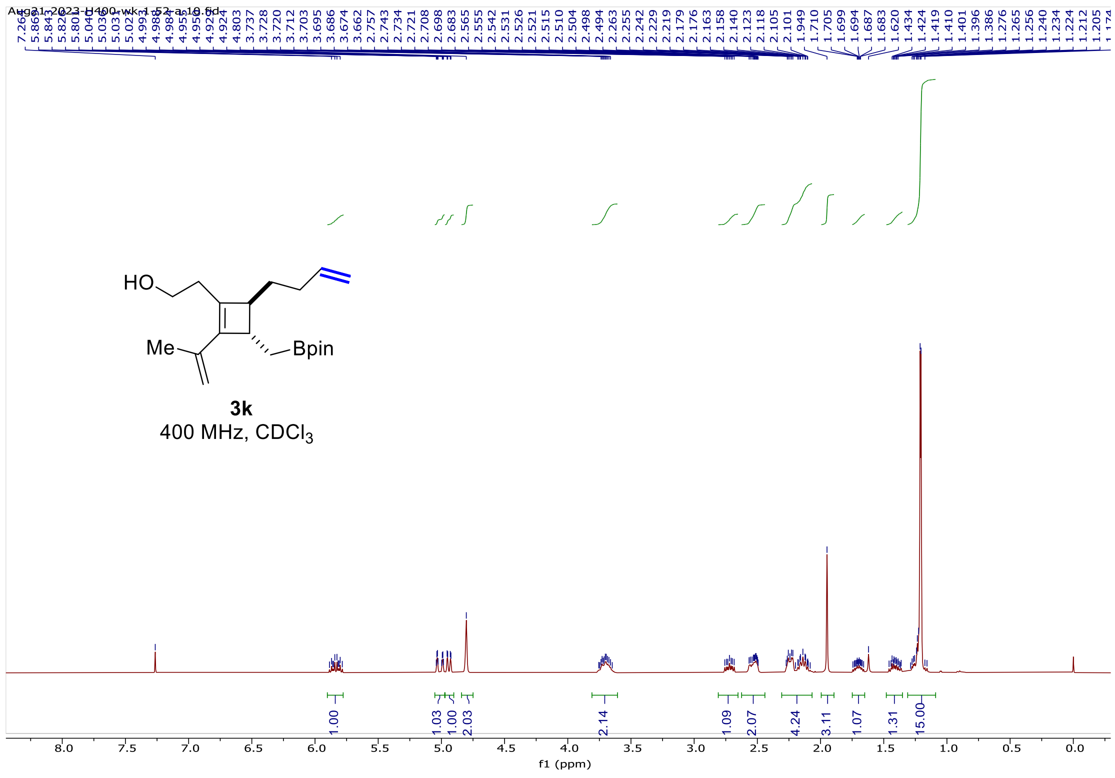
HO
Me
Bpin
3k
400 MHz, CDCl3
f1 (ppm)

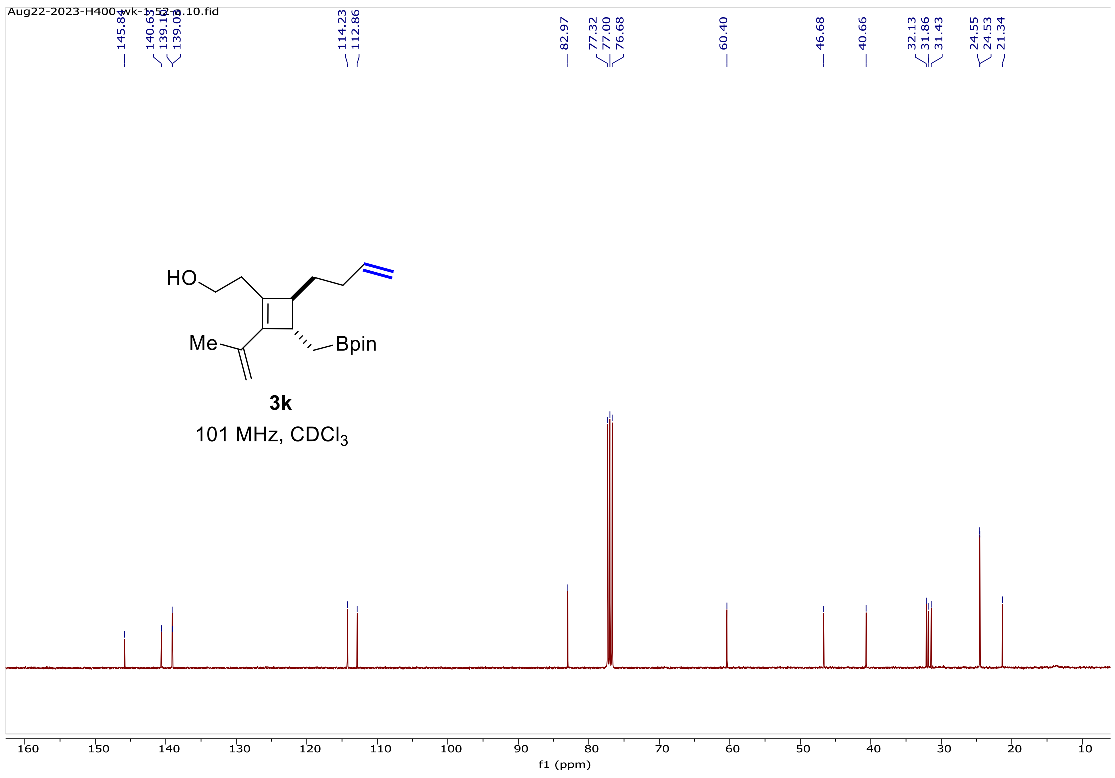
HO
Me
Bpin
3k
101 MHz, CDCl3
f1 (ppm)

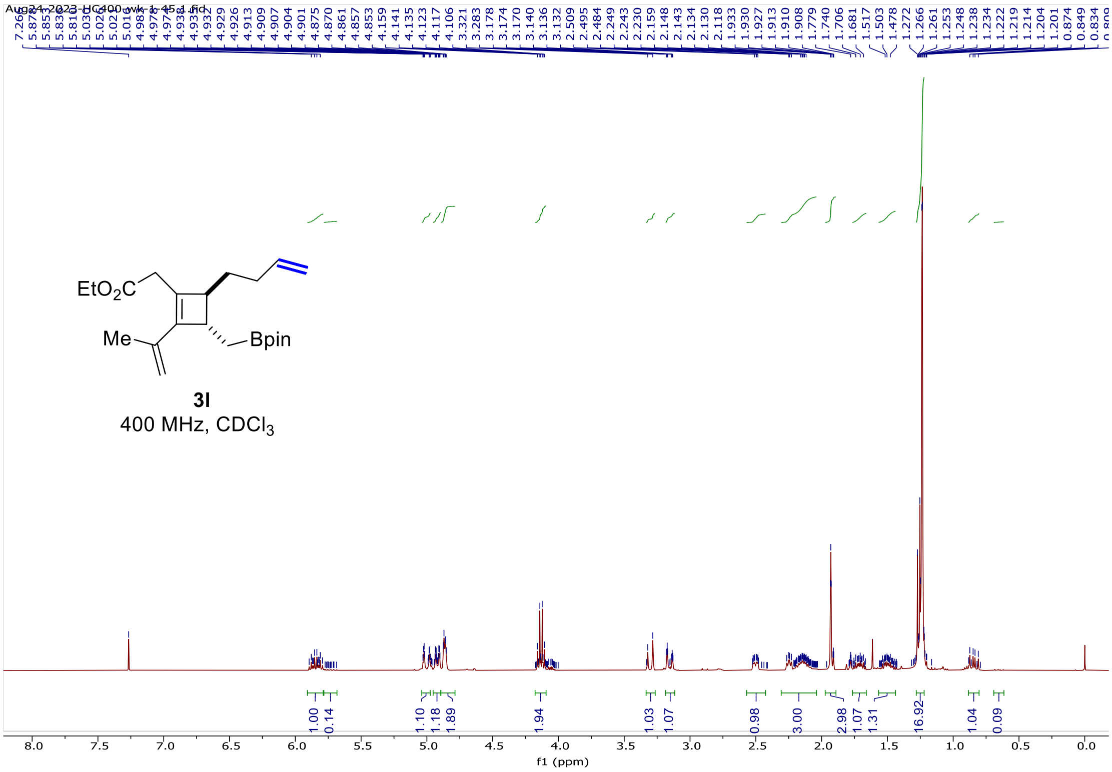
Aug24-2023-HC400-wk-1-45.1.fid
EtO2C
Me
Bpin
3l
400 MHz, CDCl3
f1 (ppm)

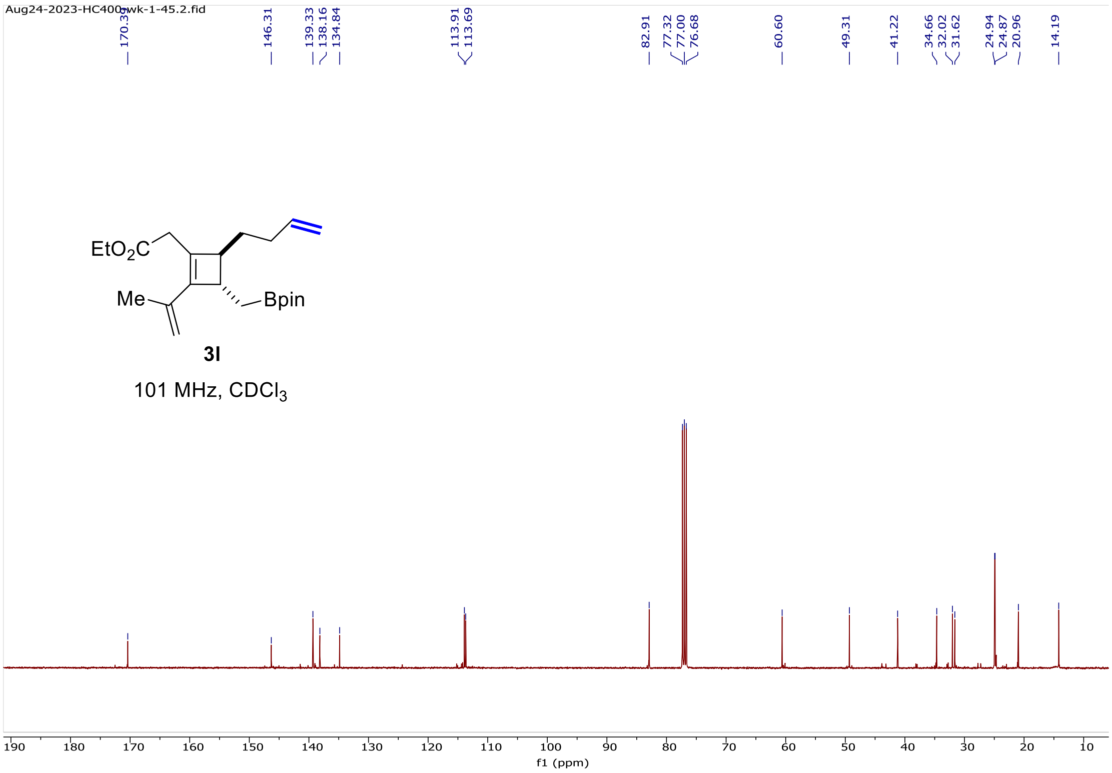
Aug24-2023-HC400-wk-1-45.2.fid
170.3
146.31
139.33
138.16
134.84
113.91
113.69
82.91
77.32
77.00
76.68
60.60
49.31
41.22
34.66
32.02
31.62
24.94
24.87
20.96
14.19
EtO2C
Me
Bpin
3l
101 MHz, CDCl3
f1 (ppm)

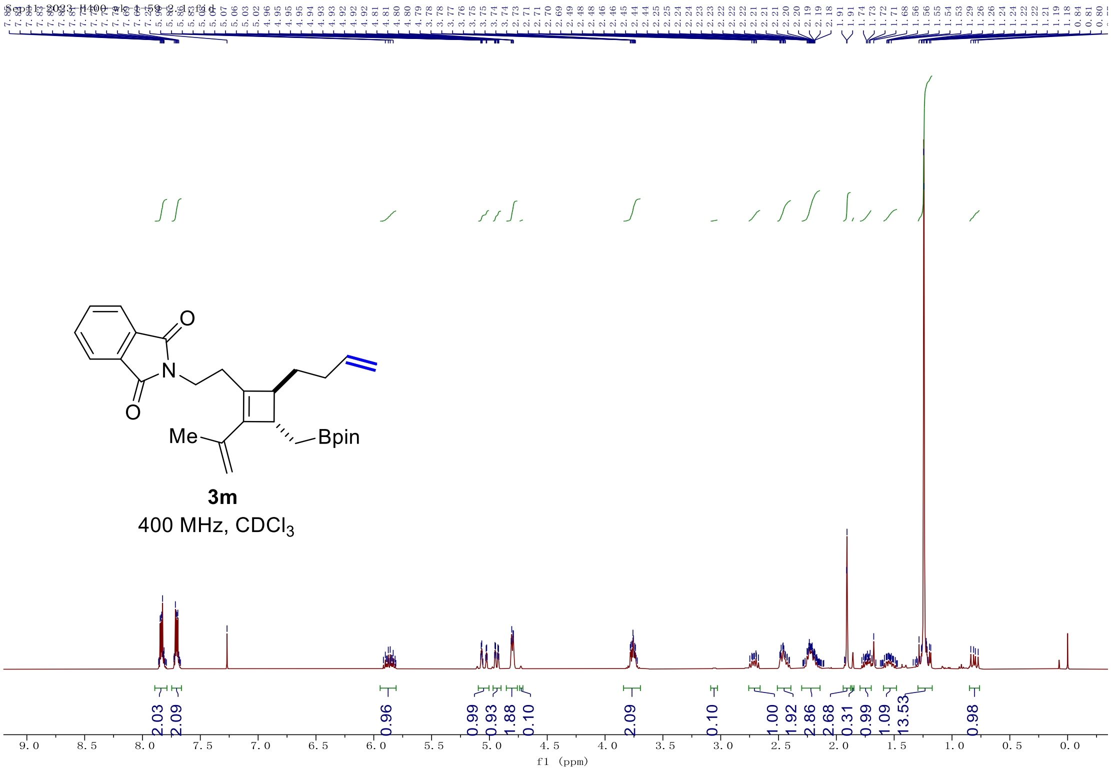
Me
Bpin
N
O
O
3m
400 MHz, CDCl$_3$
2.03
2.09
0.96
0.99
0.93
1.88
0.10
2.09
0.10
1.00
1.92
2.86
2.68
0.31
0.99
1.09
13.53
0.98
f1 (ppm)

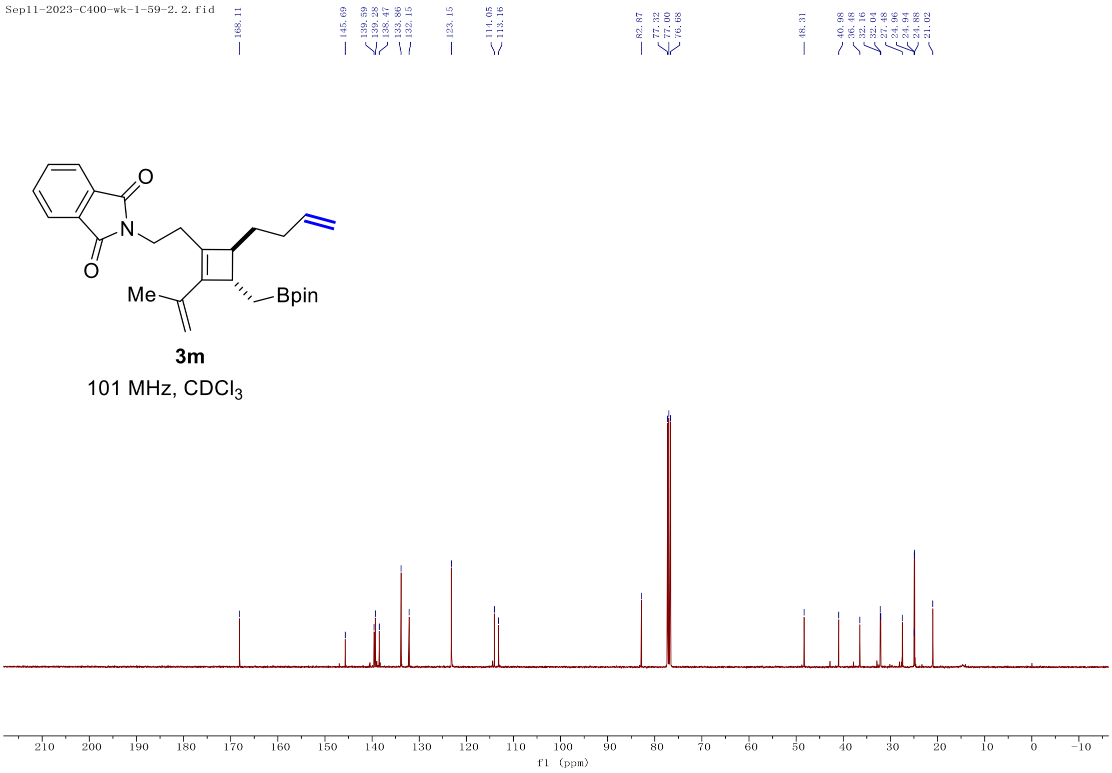
Sep11-2023-C400-wk-1-59-2.2.fid
168.11
145.69
139.59
139.28
138.47
133.86
132.15
123.15
114.05
113.16
82.87
77.32
77.00
76.68
48.31
40.98
36.48
32.16
32.04
27.48
24.96
24.94
24.88
21.02
Me
Bpin
N
O
O
3m
101 MHz, CDCl$_3$
f1 (ppm)

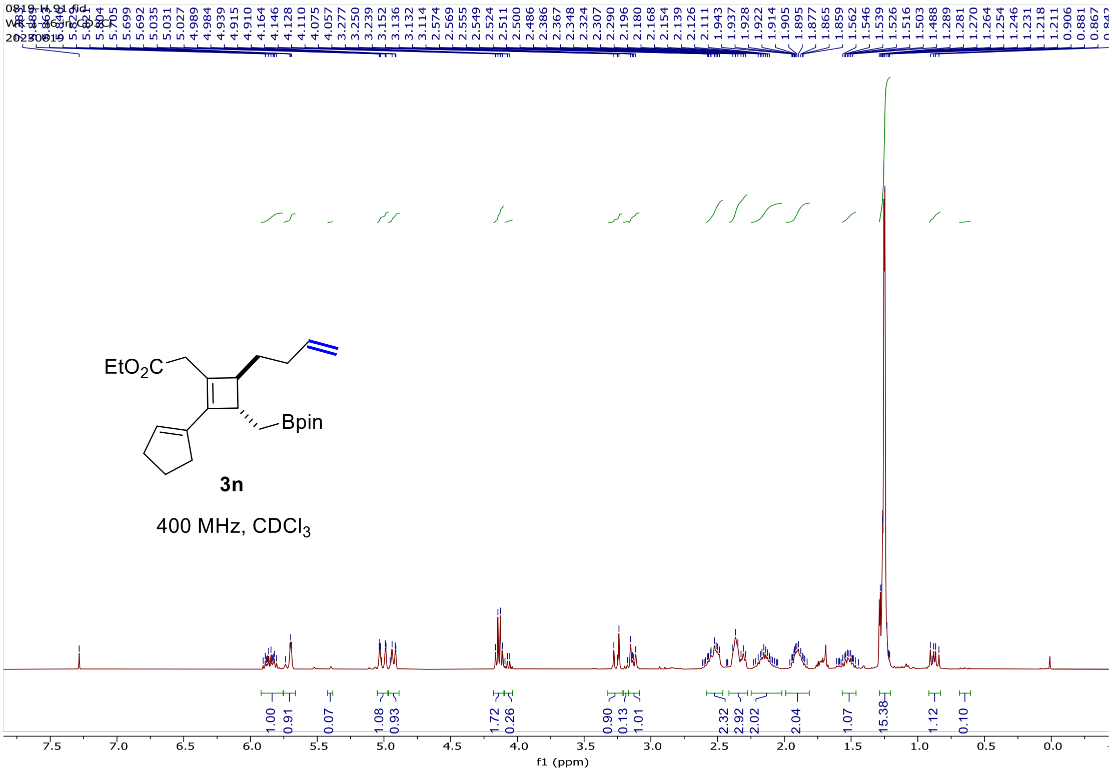
EtO2C
Bpin
3n
400 MHz, CDCl3
1.00
0.91
0.07
1.08
0.93
1.72
0.26
0.90
0.13
1.01
2.32
2.92
2.02
2.04
1.07
15.38
1.12
0.10
f1 (ppm)

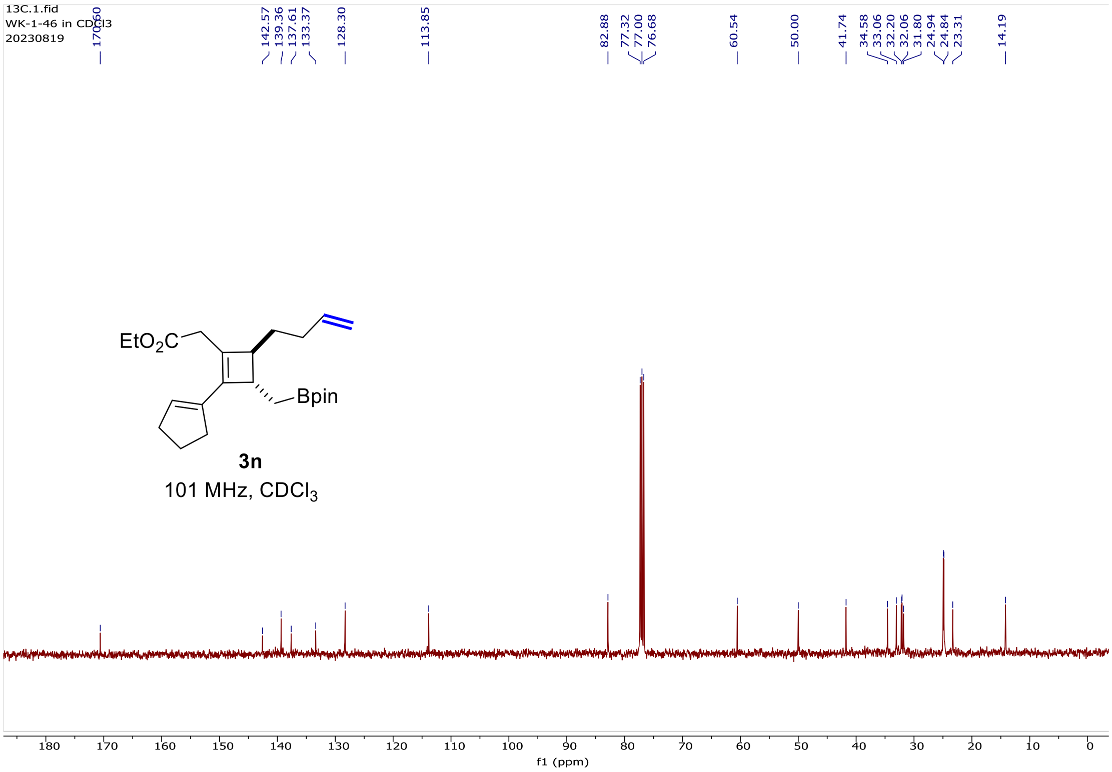
13C.1.fid
WK-1-46 in CDCl3
20230819
142.57
139.36
137.61
133.37
128.30
113.85
82.88
77.32
77.00
76.68
60.54
50.00
41.74
34.58
33.06
32.20
32.06
31.80
24.94
24.84
23.31
14.19
EtO2C
Bpin
3n
101 MHz, CDCl3
f1 (ppm)

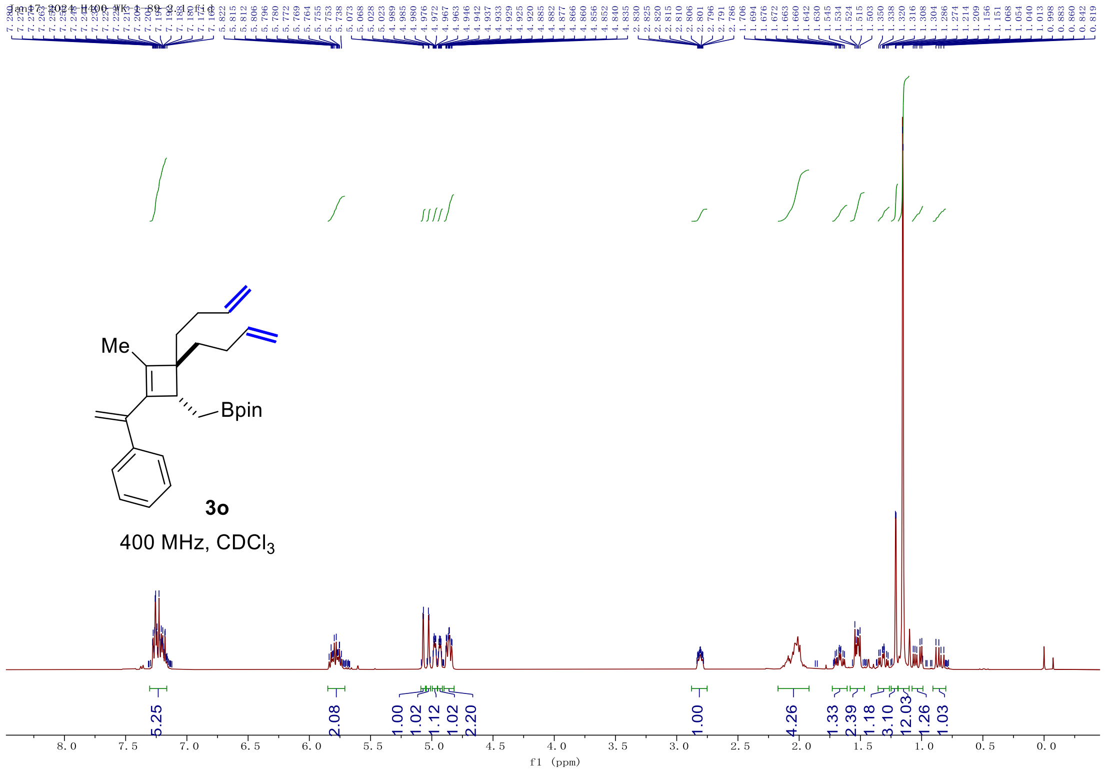
Me
Bpin
3o
400 MHz, CDCl3
5.25
2.08
1.00
1.02
1.12
1.02
2.20
1.00
4.26
1.33
2.39
1.18
3.10
12.03
1.26
1.03
f1 (ppm)

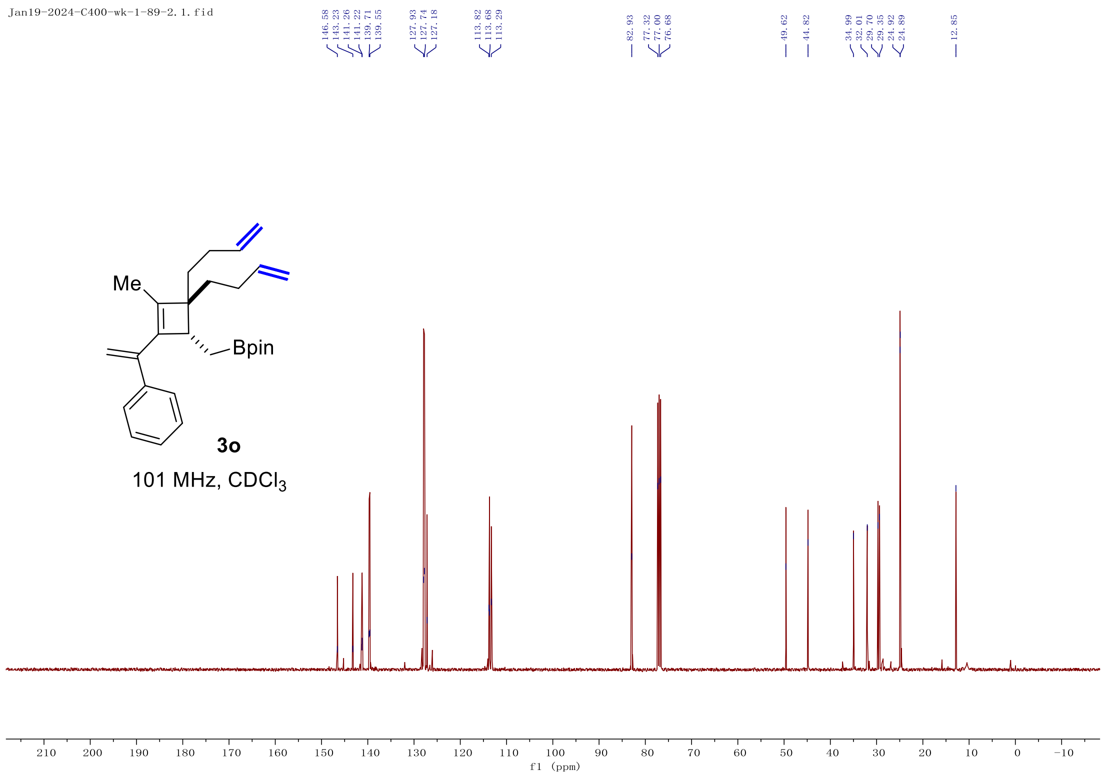
Jan19-2024-C400-wk-1-89-2.1.fid
146.58
143.23
141.26
141.22
139.71
139.55
127.93
127.74
127.18
113.82
113.68
113.29
82.93
77.32
77.00
76.68
49.62
44.82
34.99
32.01
29.70
29.35
24.92
24.89
12.85
Me
Bpin
3o
101 MHz, CDCl3
f1 (ppm)

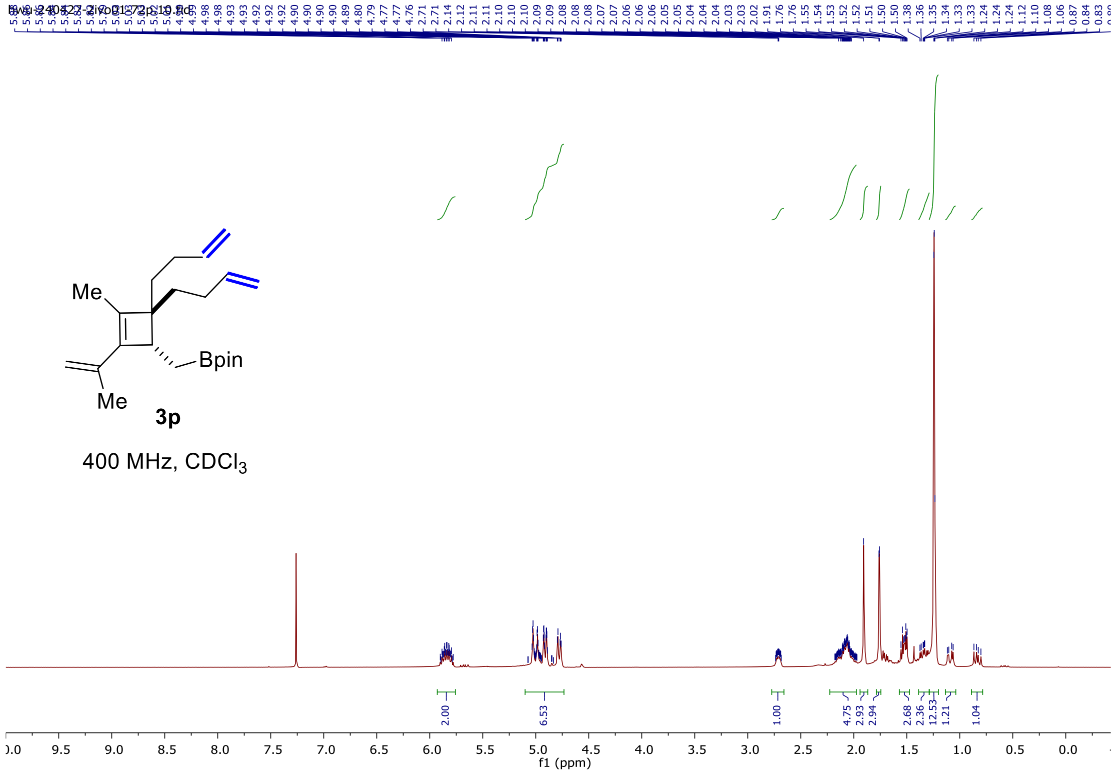

Me
Bpin
Me
3p
400 MHz, CDCl3
f1 (ppm)

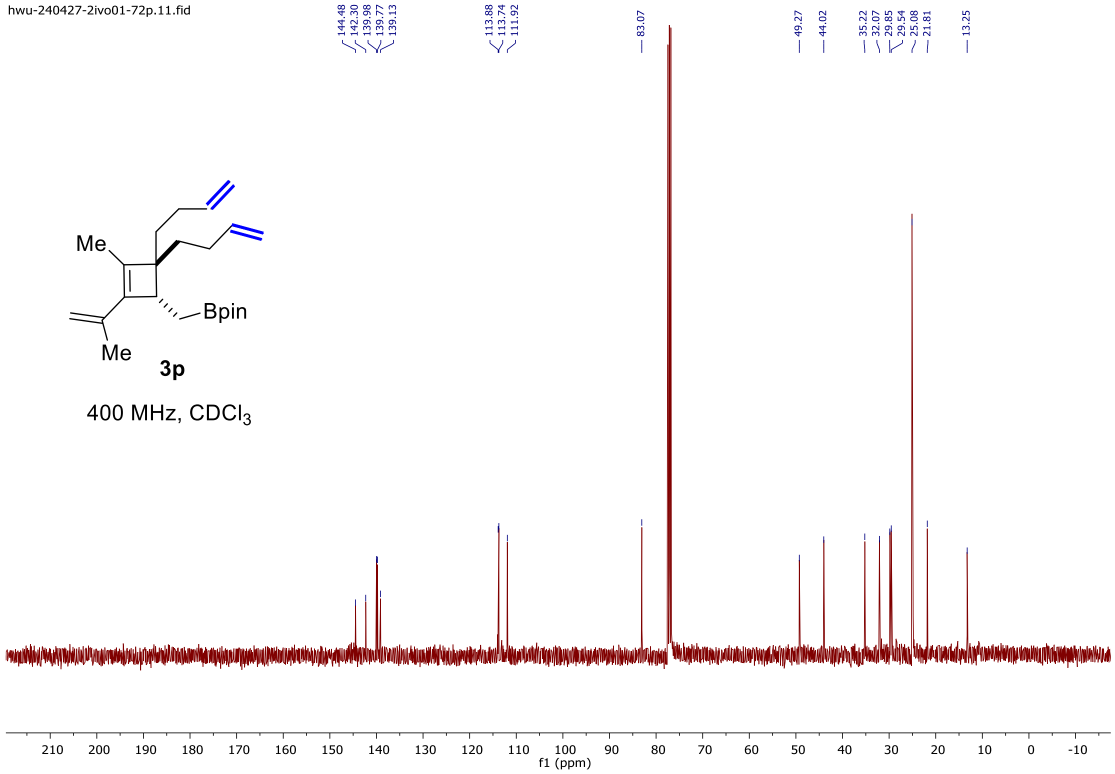

hwu-240427-2ivo01-72p.11.fid
144.48
142.30
139.98
139.77
139.13
113.88
113.74
111.92
83.07
49.27
44.02
35.22
32.07
29.85
29.54
25.08
21.81
13.25
Me
Bpin
Me
3p
400 MHz, CDCl3
f1 (ppm)

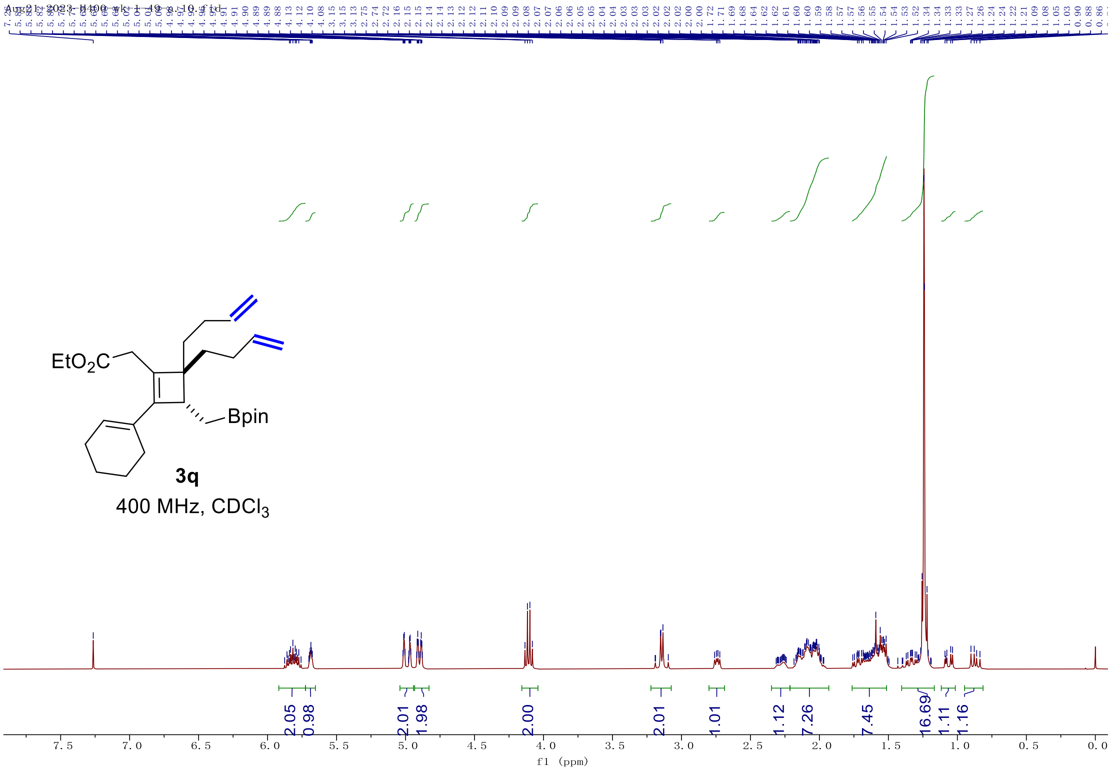
EtO2C
Bpin
3q
400 MHz, CDCl3
f1 (ppm)

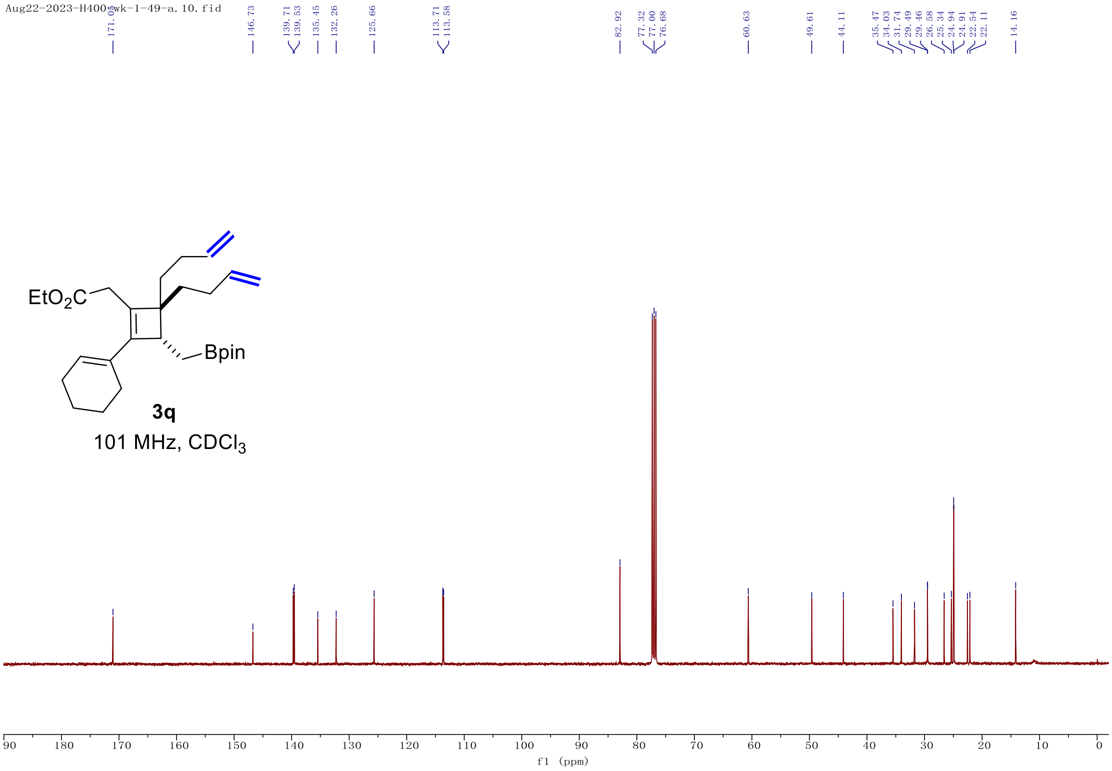
EtO2C
Bpin
3q
101 MHz, CDCl3
f1 (ppm)

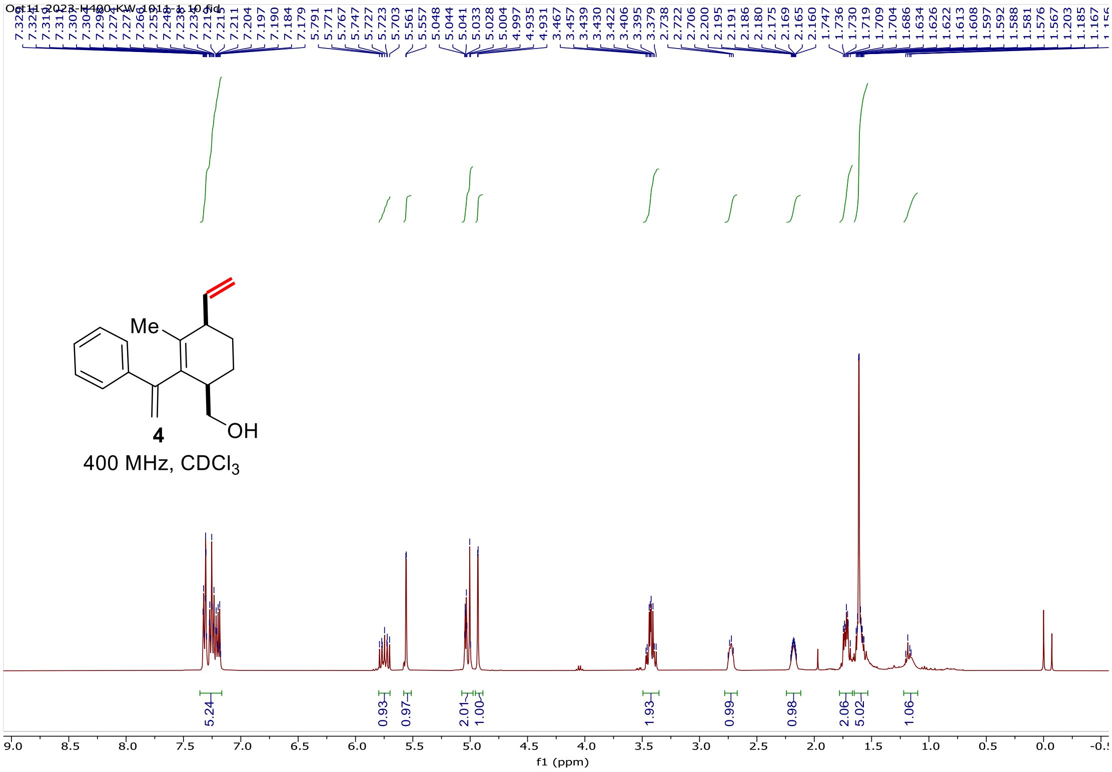
Oct11-2023-H400-KW-1011-1-10.fid
Me
OH
4
400 MHz, CDCl3
5.24
0.93
0.97
2.01
1.00
1.93
0.99
0.98
2.06
5.02
1.06
f1 (ppm)

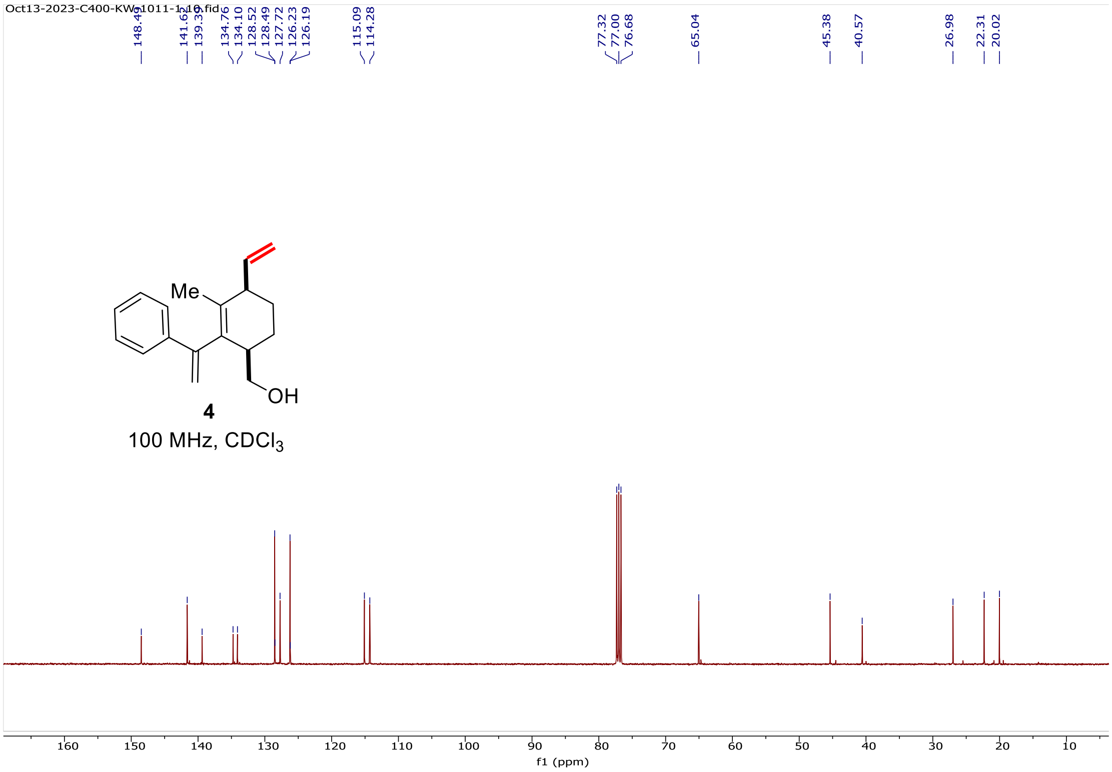
Oct13-2023-C400-KW-1011-1-10.fid
115.09
114.28
77.32
77.00
76.68
65.04
45.38
40.57
26.98
22.31
20.02
Me
OH
4
100 MHz, CDCl3
f1 (ppm)

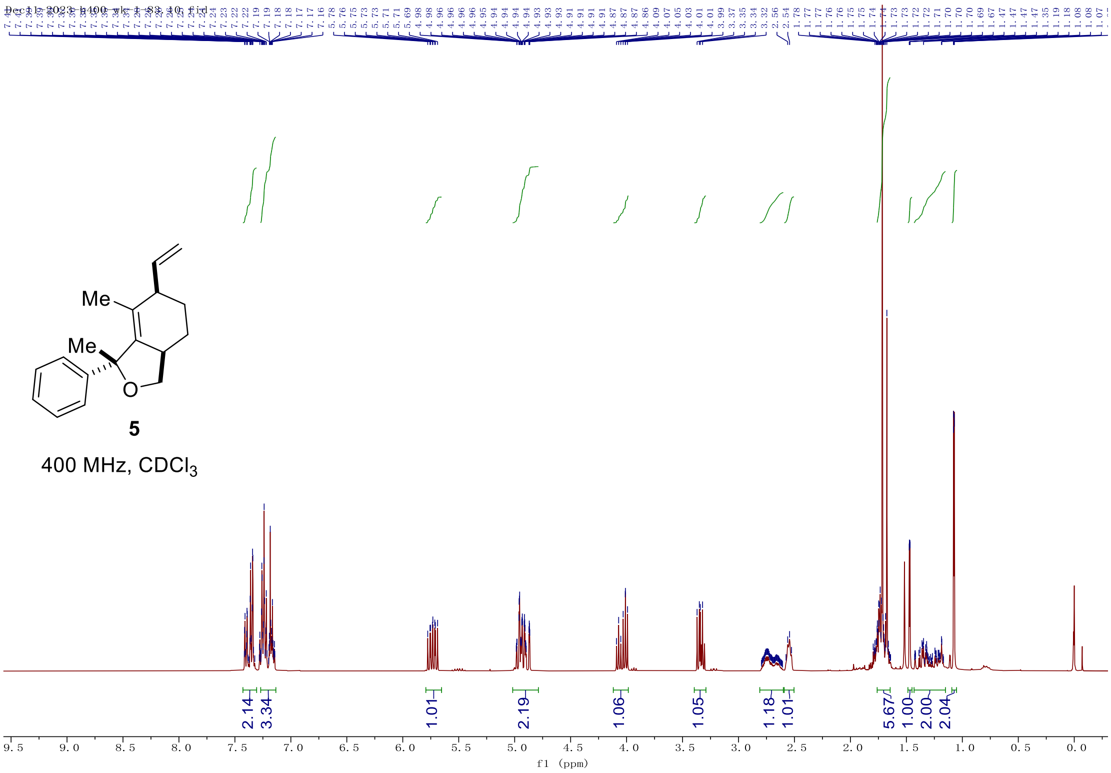
Me
Me
O
5
400 MHz, CDCl3
2.14
3.34
1.01
2.19
1.06
1.05
1.18
1.01
5.67
1.00
2.00
2.04
f1 (ppm)

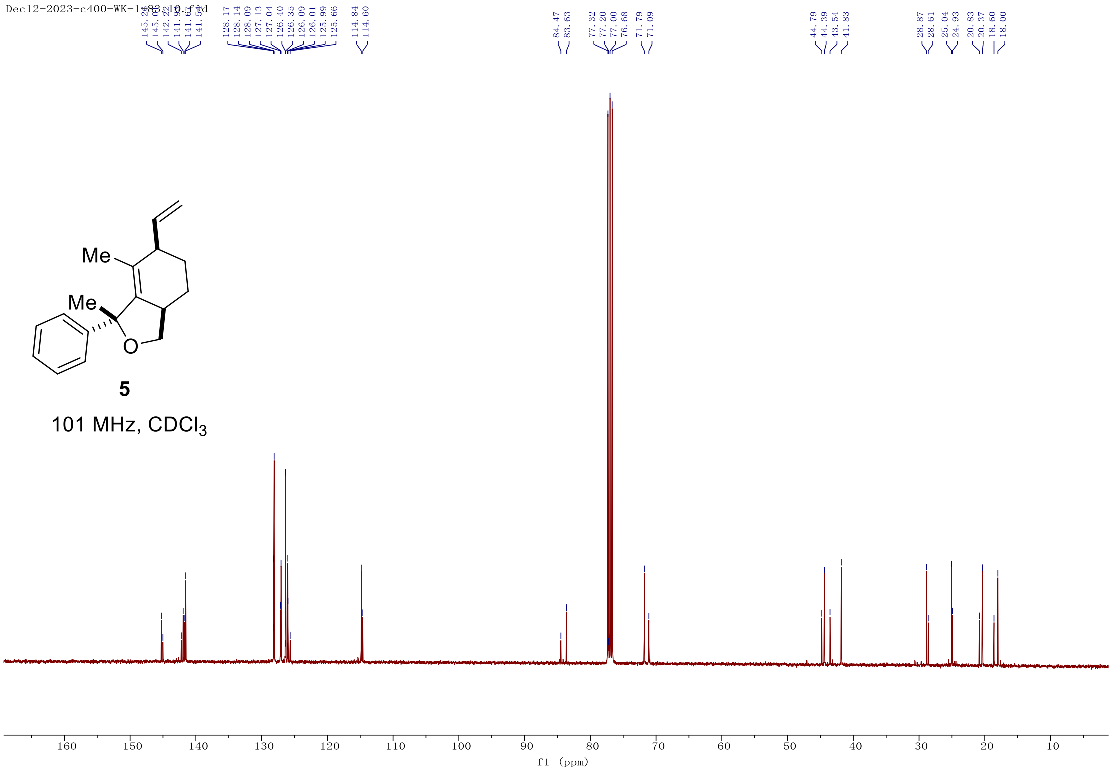
128.17
128.14
128.09
127.13
127.04
126.40
126.35
126.09
126.01
125.99
125.66
114.84
114.60
84.47
83.63
77.32
77.20
77.00
76.68
71.79
71.09
44.79
44.39
43.54
41.83
28.87
28.61
25.04
24.93
20.83
20.37
18.60
18.00
Me
Me
O
5
101 MHz, CDCl3
f1 (ppm)

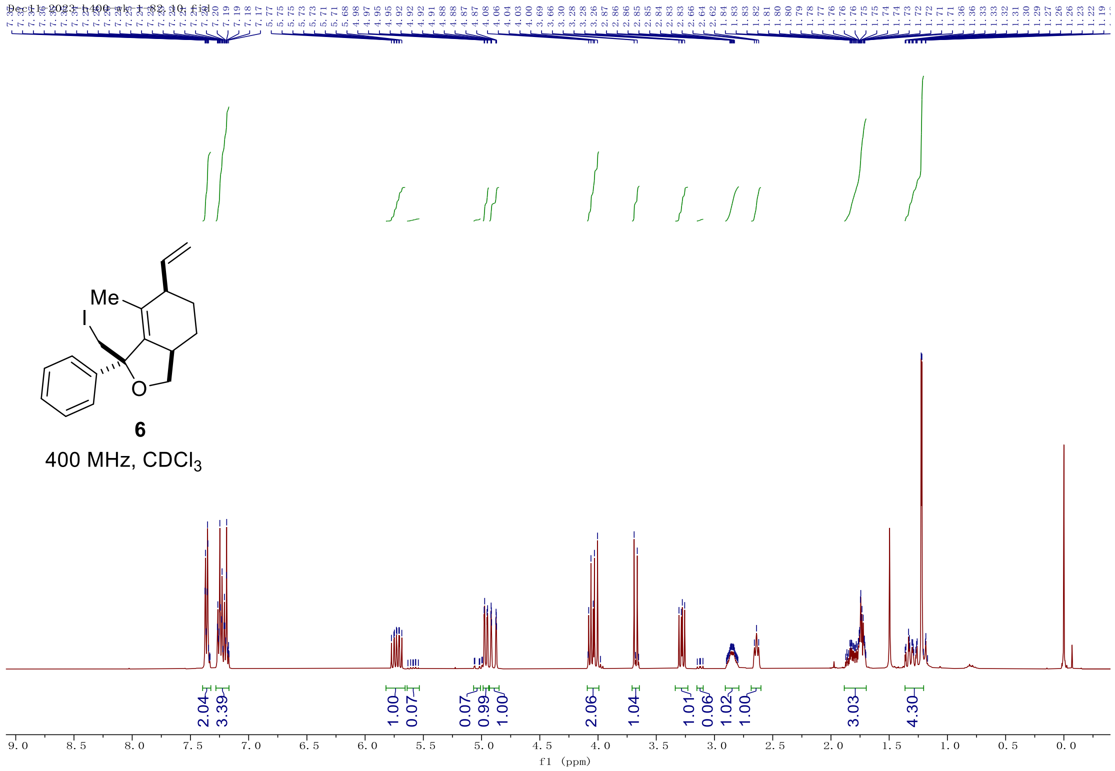

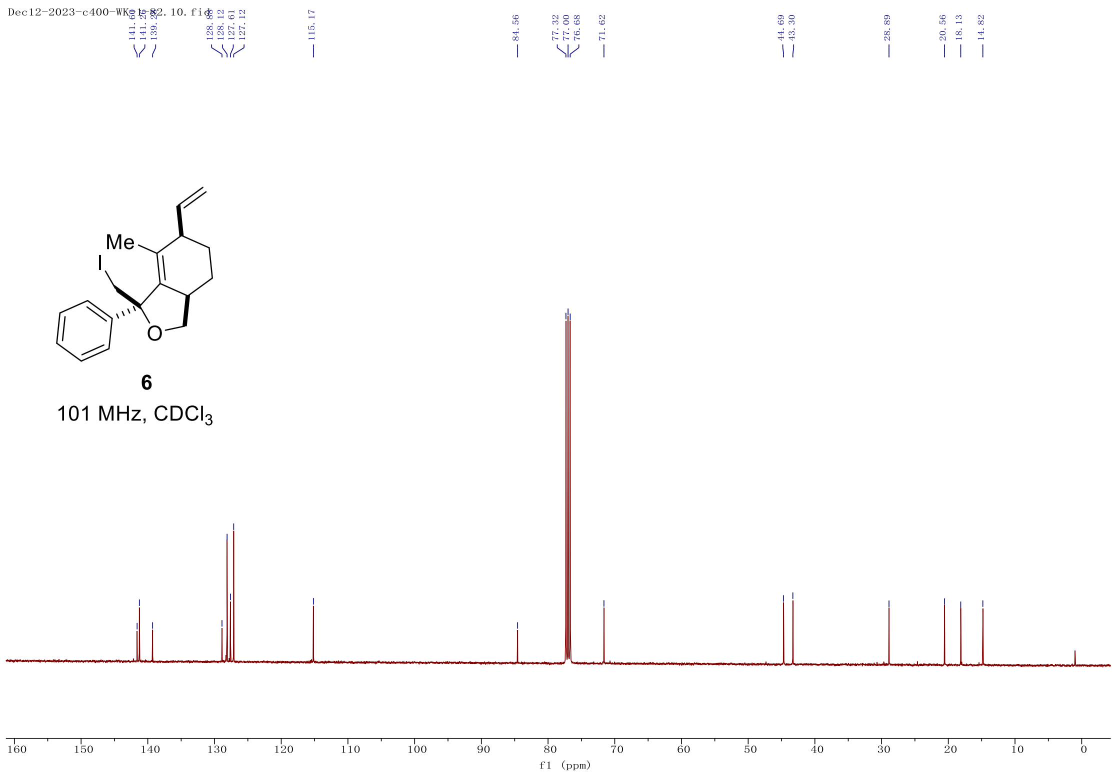

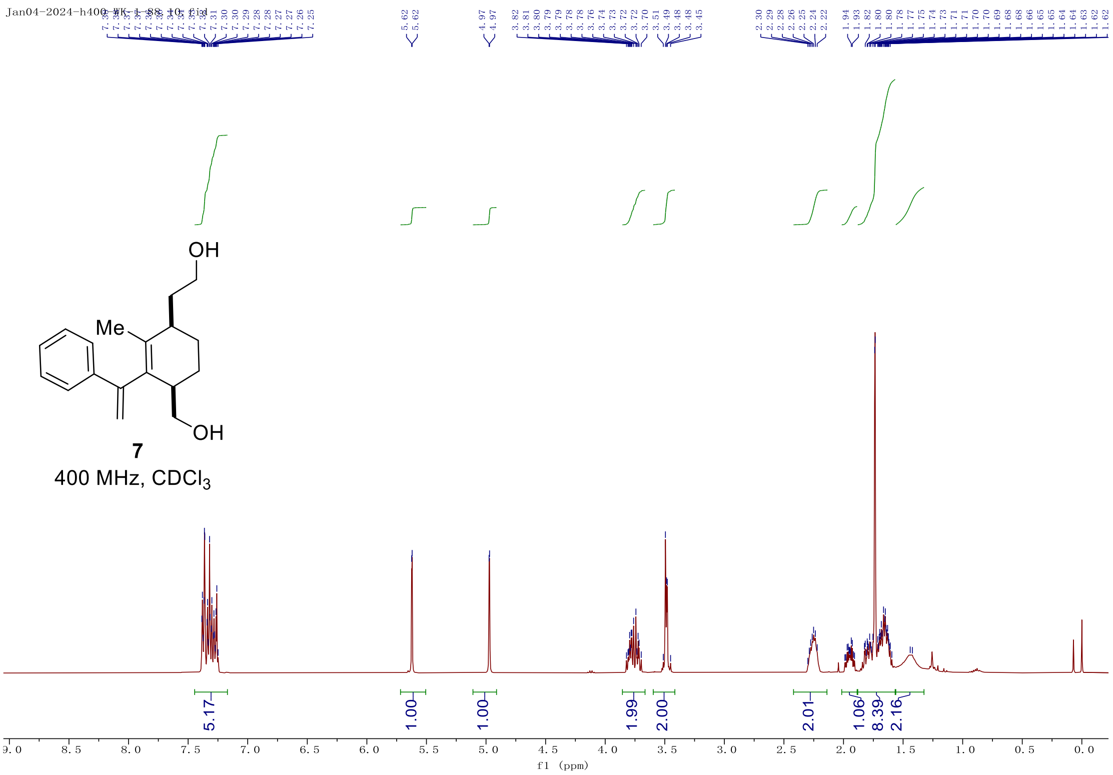
Jan04-2024-h400-WK-1-88.10.fid
OH
Me
OH
7
400 MHz, CDCl3
5.17
1.00
1.00
1.99
2.00
2.01
1.06
8.39
2.16
f1 (ppm)

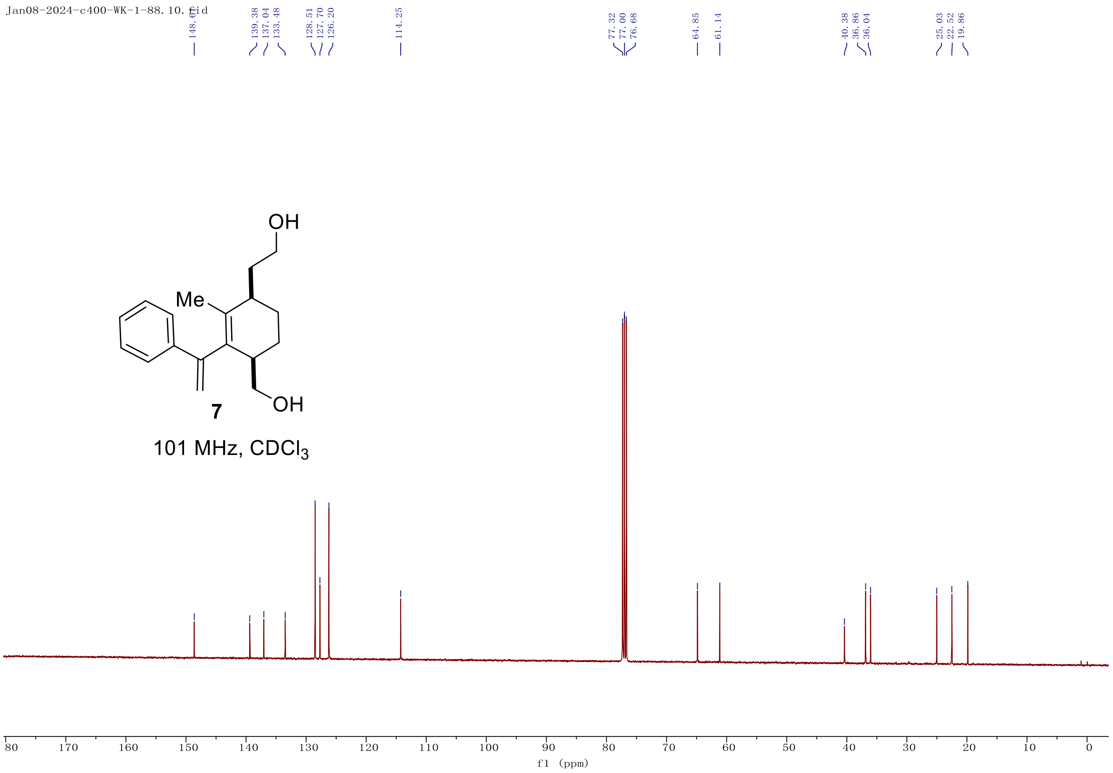
Jan08-2024-c400-WK-1-88.10.fid
148.6
139.38
137.04
133.48
128.51
127.70
126.20
114.25
77.32
77.00
76.68
64.85
61.14
40.38
36.86
36.04
25.03
22.52
19.86
OH
Me
OH
7
101 MHz, CDCl3
f1 (ppm)

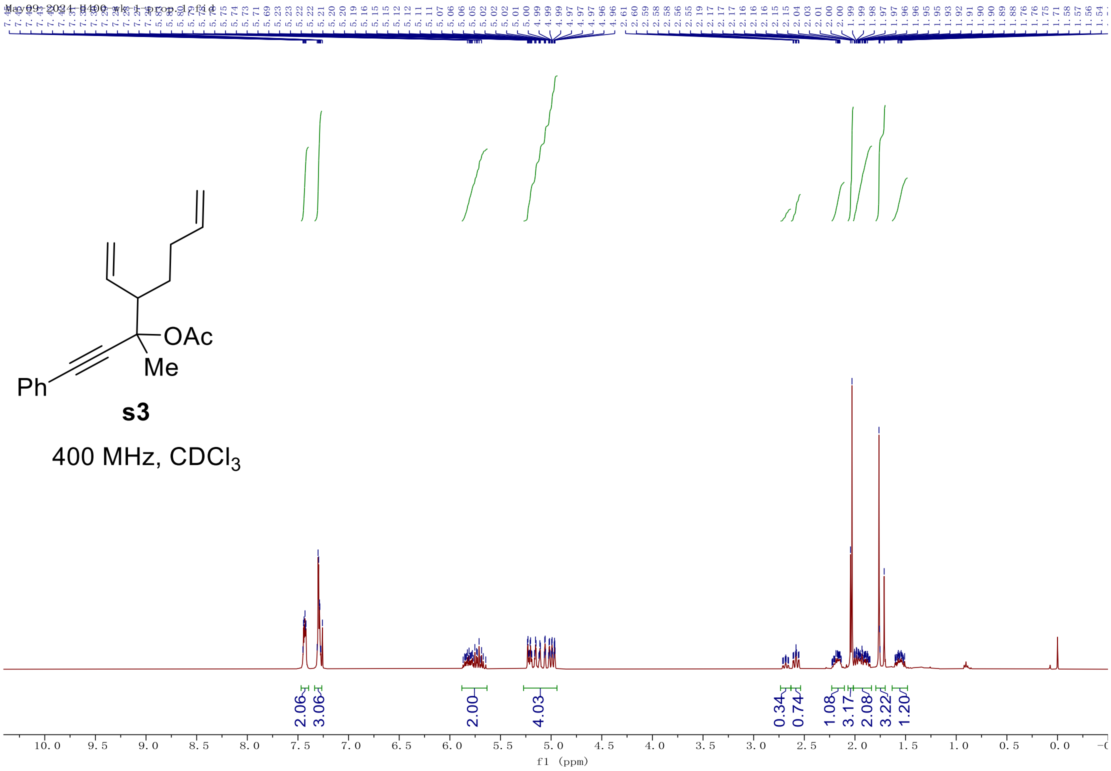

s3
400 MHz, CDCl$_3$
2.06
3.06
2.00
4.03
0.34
0.74
1.08
3.17
2.08
3.22
1.20
f1 (ppm)

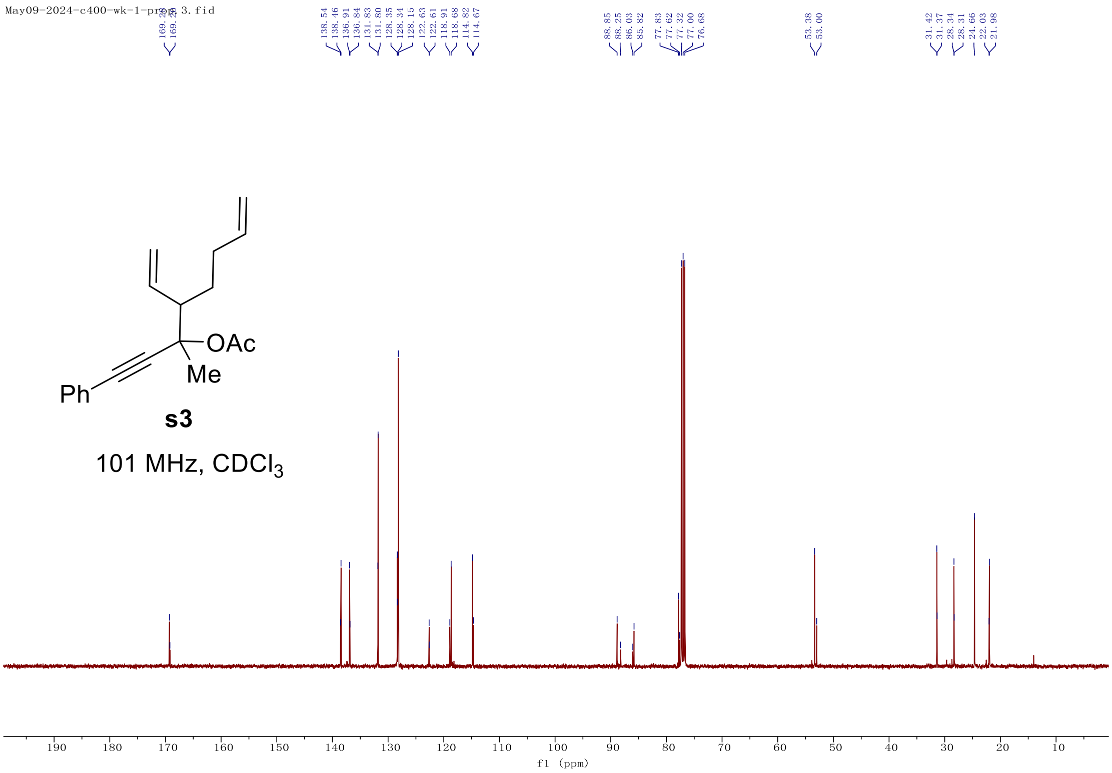

s3
101 MHz, CDCl$_3$
138.54
138.46
136.91
136.84
131.83
131.80
128.35
128.34
128.15
122.63
122.61
118.91
118.68
114.82
114.67
88.85
88.25
86.03
85.82
77.83
77.62
77.32
77.00
76.68
53.38
53.00
31.42
31.37
28.34
28.31
24.66
22.03
21.98
f1 (ppm)

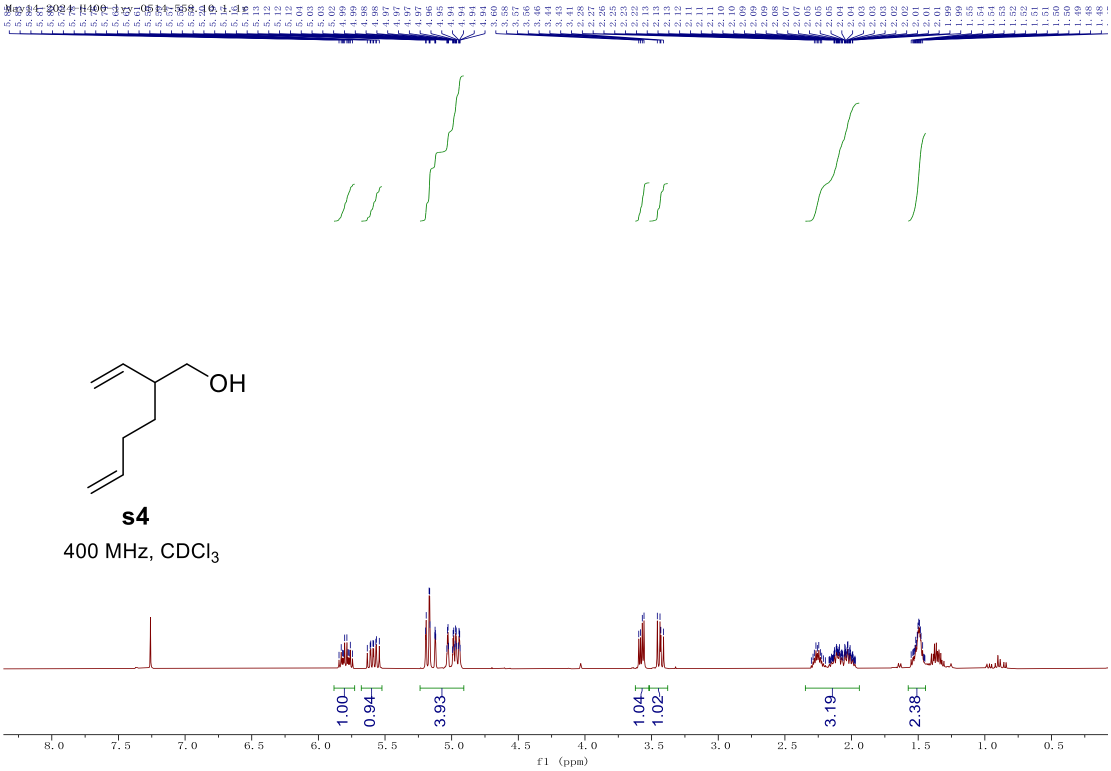
OH
s4
400 MHz, CDCl3
1.00
0.94
3.93
1.04
1.02
3.19
2.38
f1 (ppm)

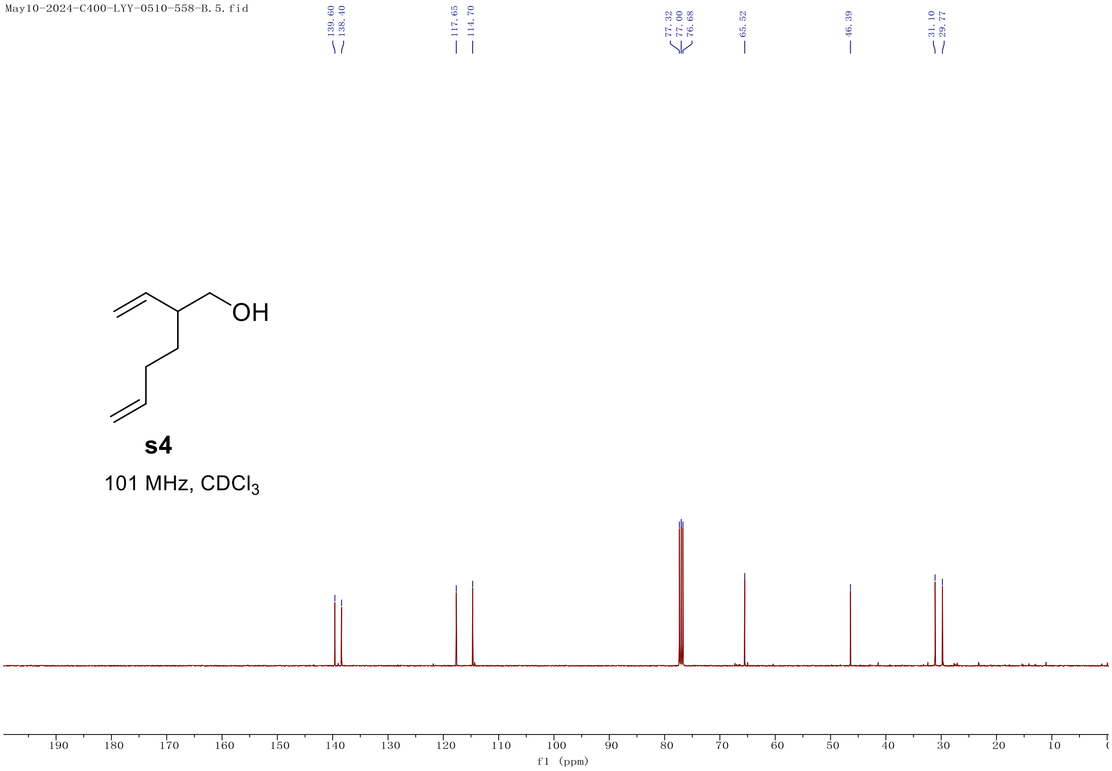
May10-2024-C400-LYY-0510-558-B.5.fid
139.60
138.40
117.65
114.70
77.32
77.00
76.68
65.52
46.39
31.10
29.77
OH
s4
101 MHz, CDCl3
f1 (ppm)

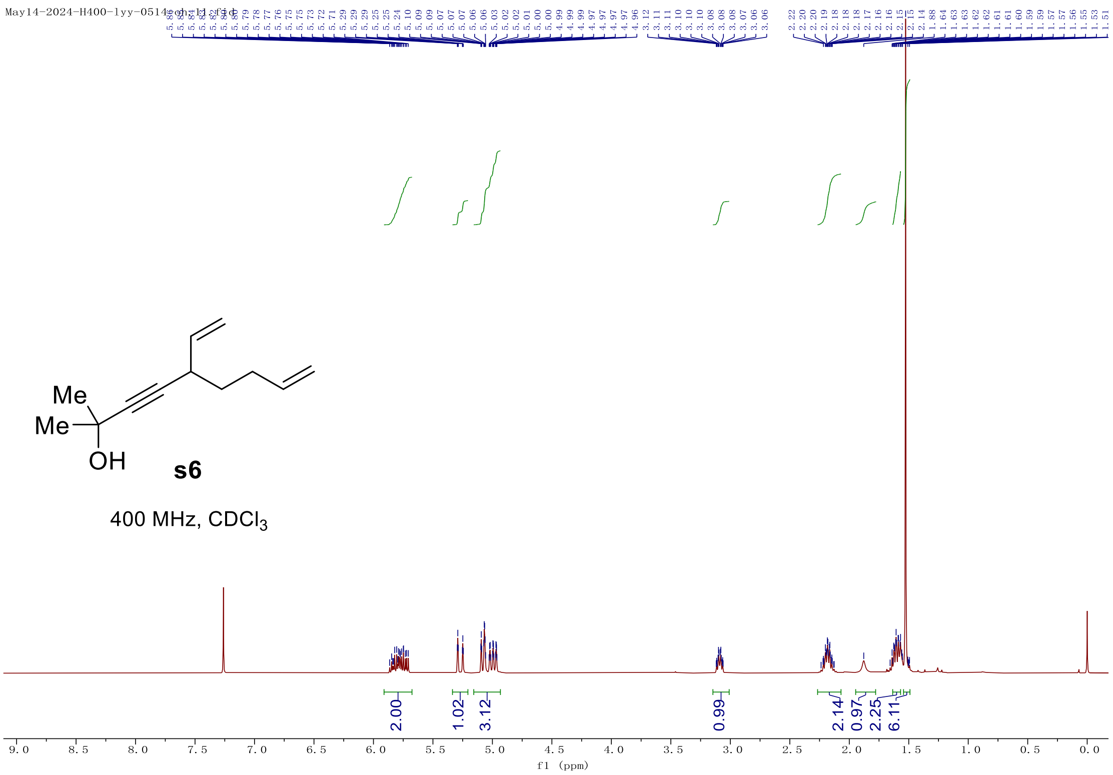
May14-2024-H400-lyy-0514-oh.11.fid
Me
Me
OH
s6
400 MHz, CDCl3
2.00
1.02
3.12
0.99
2.14
0.97
2.25
6.11
f1 (ppm)

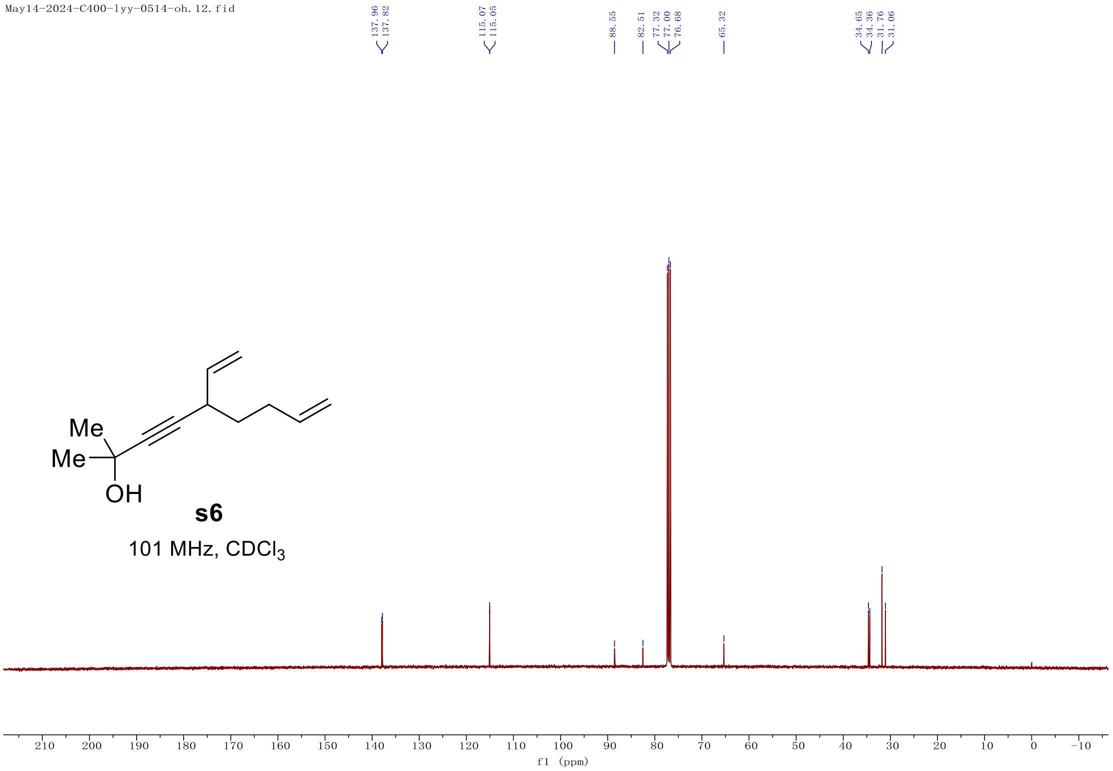
May14-2024-C400-lyy-0514-oh.12.fid
137.96
137.82
115.07
115.05
88.55
82.51
77.32
77.00
76.68
65.32
34.65
34.36
31.76
31.06
Me
Me
OH
s6
101 MHz, CDCl3
f1 (ppm)

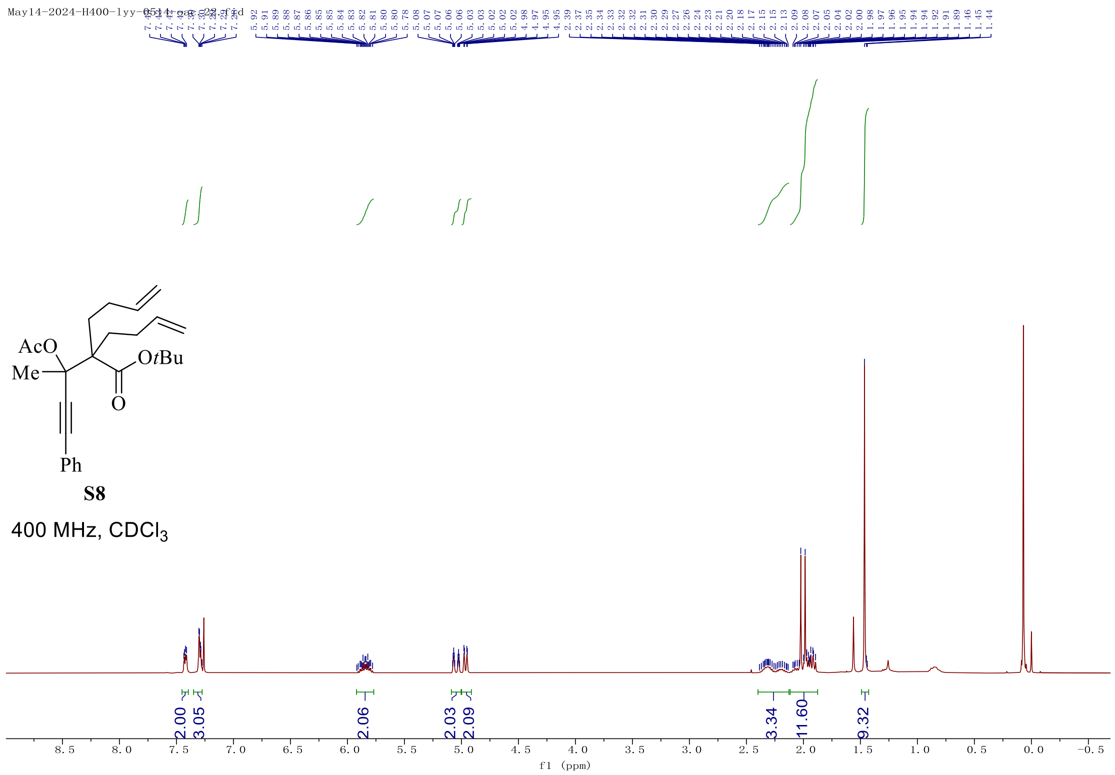
AcO
Me
OtBu
O
Ph
S8
400 MHz, CDCl3
2.00
3.05
2.06
2.03
2.09
3.34
11.60
9.32
f1 (ppm)

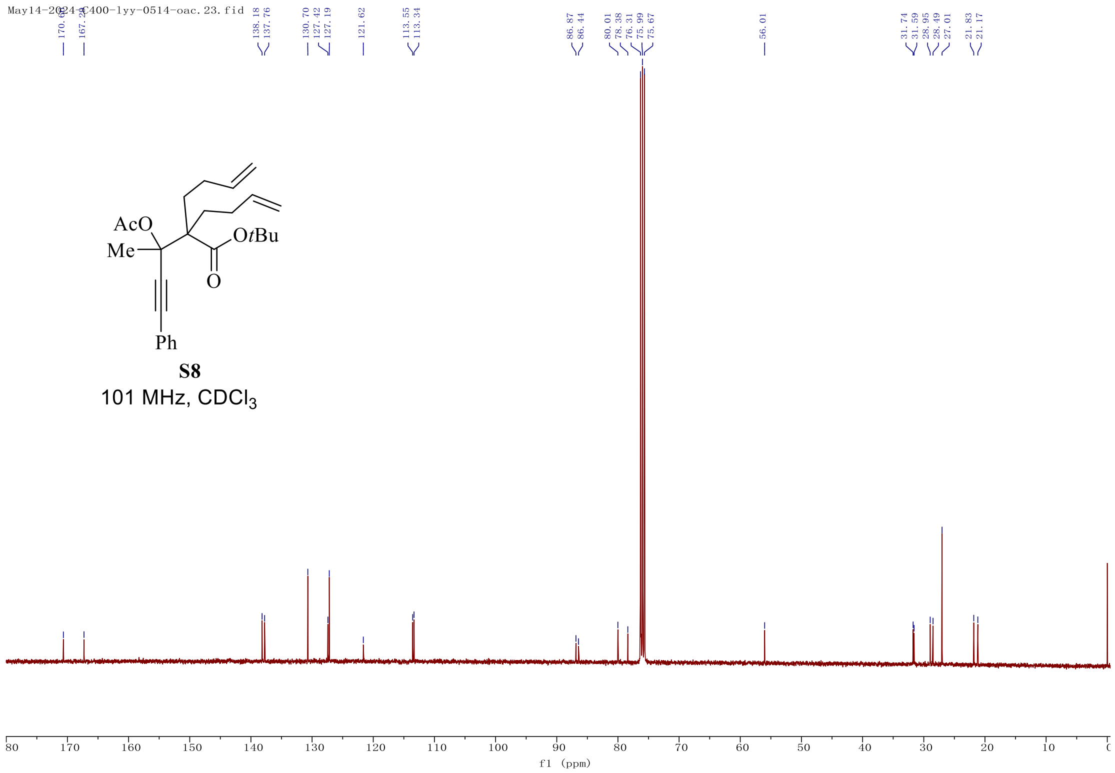
May14-2024-C400-lyy-0514-oac.23.fid
138.18
137.76
130.70
127.42
127.19
121.62
113.55
113.34
86.87
86.44
80.01
78.38
76.31
75.99
75.67
56.01
31.74
31.59
28.95
28.49
27.01
21.83
21.17
AcO
Me
OtBu
O
Ph
S8
101 MHz, CDCl3
f1 (ppm)

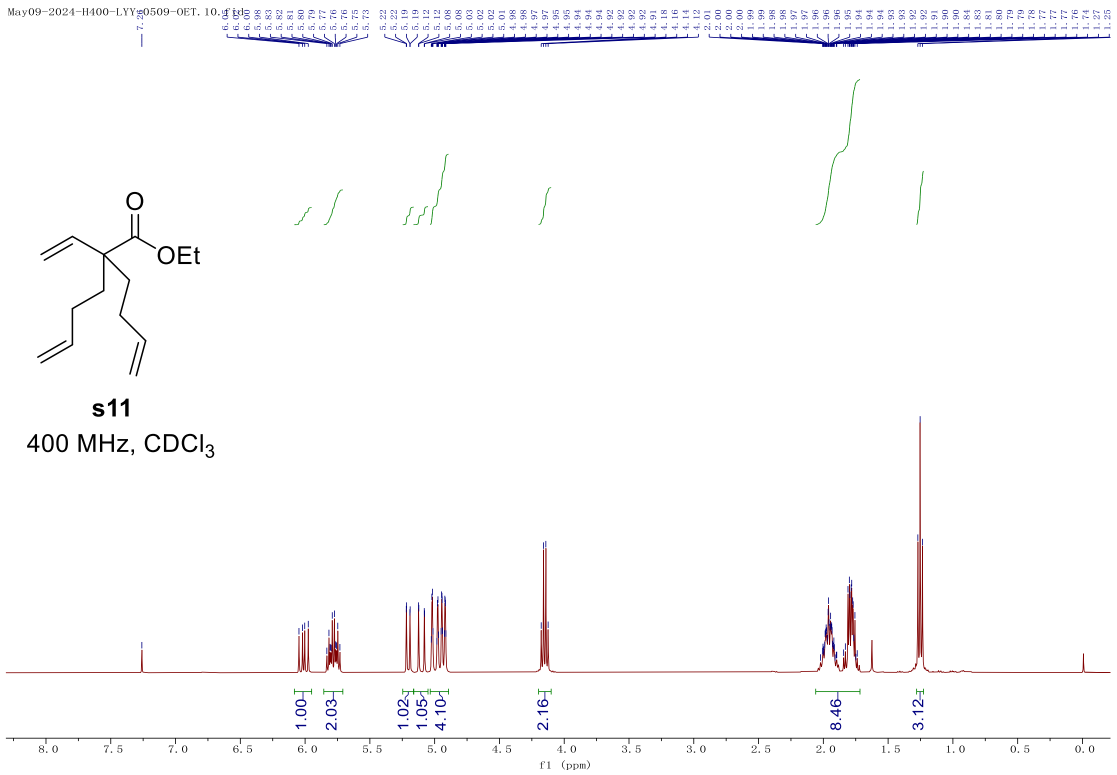

OEt
s11
400 MHz, CDCl3
1.00
2.03
1.02
1.05
4.10
2.16
8.46
3.12
f1 (ppm)

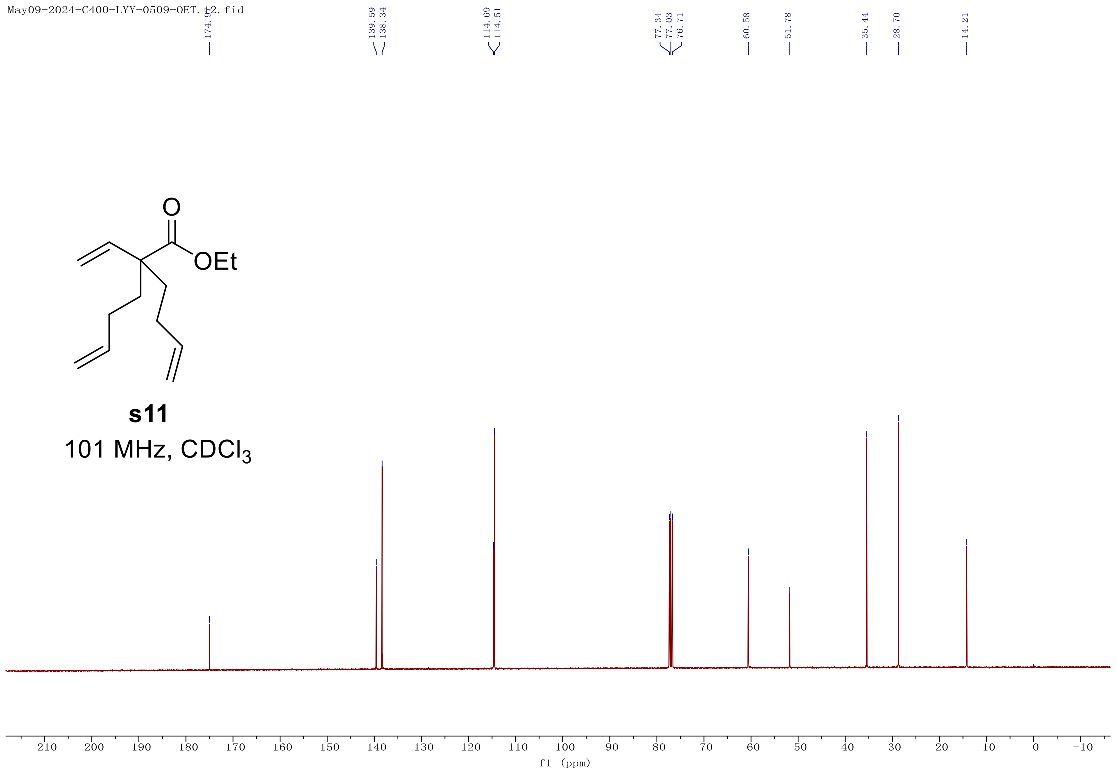

139.59
138.34
114.69
114.51
77.34
77.03
76.71
60.58
51.78
35.44
28.70
14.21
OEt
s11
101 MHz, CDCl3
f1 (ppm)

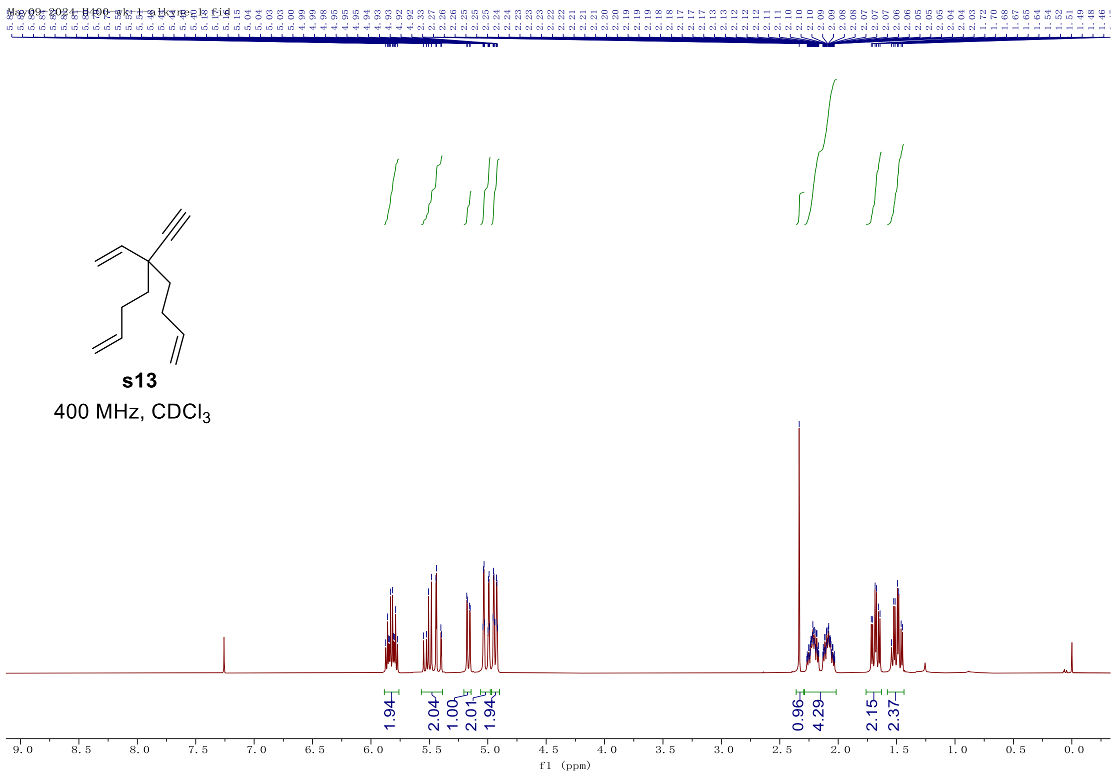
s13
400 MHz, CDCl3
1.94
2.04
1.00
2.01
1.94
0.96
4.29
2.15
2.37
f1 (ppm)

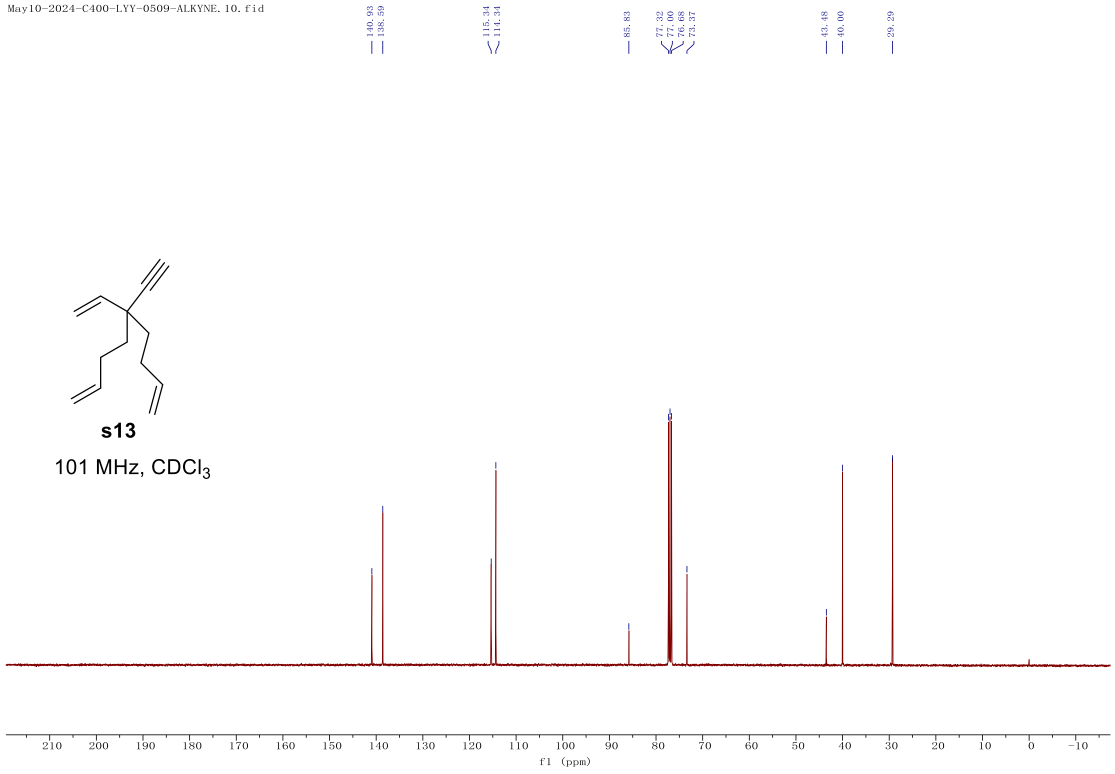
May10-2024-C400-LYY-0509-ALKYNE.10.fid
140.93
138.59
115.34
114.34
85.83
77.32
77.00
76.68
73.37
43.48
40.00
29.29
s13
101 MHz, CDCl3
f1 (ppm)
